# RNA-Seq reveals a xenobiotic stress response in the soybean aphid, *Aphis glycines*, when fed aphid-resistant soybean

**DOI:** 10.1186/1471-2164-15-972

**Published:** 2014-11-16

**Authors:** Raman Bansal, MAR Mian, Omprakash Mittapalli, Andy P Michel

**Affiliations:** Department of Entomology, The Ohio Agricultural Research and Development Center, The Ohio State University, 1680 Madison Ave., Wooster, OH 44691 USA; USDA-ARS Corn and Soybean Research Unit, and the Department of Horticulture and Crop Sciences, OARDC, The Ohio State University, 1680 Madison Ave., Wooster, OH 44691 USA

**Keywords:** *Aphis glycines*, Host-plant resistance, Biotype, RNA-Seq

## Abstract

**Background:**

While much recent research has expanded our understanding of the molecular interactions between aphids and their host plants, it is lacking for the soybean aphid, *Aphis glycines*. Since its North American invasion, *A. glycines* has become one of the most damaging insect pests on this important crop. Five soybean genes for host plant resistance to *A. glycines* have been identified, but populations of *A. glycines* have already adapted to overcome these resistance genes. Understanding the molecular interactions between resistant soybean and *A. glycines* can provide clues to its adaptation mechanisms. Here, we used RNA-Sequencing to compare and contrast *A. glycines* gene expression when fed resistant (*Rag1*) and susceptible soybean.

**Results:**

Combining results from a previous *A. glycines* transcriptome, we generated 64,860 high quality transcripts, totaling 41,151,086 bases. Statistical analysis revealed 914 genes with significant differential expression. Most genes with higher expression in *A. glycines* on resistant plants (N = 352) were related to stress and detoxification such as cytochrome P450s, glutathione-S-transferases, carboxyesterases, and ABC transporters. A total of 562 genes showed lower transcript abundance in *A. glycines* on resistant plants. From our extensive transcriptome data, we also identified genes encoding for putative salivary effector proteins (N = 73). Among these, 6 effector genes have lower transcript abundance in *A. glycines* feeding on resistant soybean.

**Conclusions:**

Overall, *A. glycines* exhibited a pattern typical of xenobiotic challenge, thereby validating antibiosis in *Rag1*, presumably mediated through toxic secondary metabolites. Additionally, this study identified many *A. glycines* genes and gene families at the forefront of its molecular interaction with soybean. Further investigation of these genes in other biotypes may reveal adaptation mechanisms to resistant plants.

**Electronic supplementary material:**

The online version of this article (doi:10.1186/1471-2164-15-972) contains supplementary material, which is available to authorized users.

## Background

The ability of plants to defend against insect feeding has long been a research focus to understand adaptation and co-evolution [1]. Sometimes referred to as a classic evolutionary arms race, these naturally evolved systems are often exploited in crop plants that offer resistance to insect pests as a way to prevent damage and protect yield, *i.e.* host plant resistance (HPR) [2]. Many host-plant resistant cultivars target aphids because they are arguably the most insidious pests of agronomic and horticultural crops worldwide [3, 4]. Yet several aphid species have been able to overcome this resistance in the form of virulent biotypes, which threatens the utility and sustainability of aphid resistant varieties [4]. Research on the molecular interactions between aphids and their host plants will allow comparative approaches to both expand our understanding of co-evolution as well as improve the durability of plant resistance.

Induced plant defenses usually involve the production of plant secondary metabolites (PSMs) that are toxic to insects. In turn, most insects respond to PSMs by inducing an array of stress response proteins including enzymes for metabolic excretion [5]. The metabolic excretion of PSMs and other xenobiotics by insects tends to occur in three phases [5–7]. In phase I, the biological activity of the specific metabolite is reduced, with cytochrome P450s acting as principal enzymes [6]. In phase II, the by-products of phase I are conjugated with hydrophilic substances to increase water-solubility which facilitates their excretion [6]. Phase II enzymes include glutathione S-transferases (GSTs), carboxylesterases (COEs), and UDP-glucuronlytransferases (UGTs). Finally, in phase III, conjugated compounds are exported out of the cell by employing ATP-binding cassette (ABC) and other transmembrane transporters [6].

In addition to inducing xenobiotic metabolism genes, insect stress and defense responses can also involve important proteins such as heat-shock proteins, proteases (to evade plant protease inhibitors), and multicopper oxidases (*e.g*. laccase-1 to oxidize PSMs) [8, 9]. Transcriptional activity of insect xenobiotic stress response machinery is regulated by transcription factors (TFs) [10]. Previous evidence, though limited, suggests that aphids utilize diverse mechanisms, including detoxification and other defense pathways, to cope with PSMs and host-plant resistance [11].

Another important factor in aphid-plant interactions are effectors [12–14]. Primarily, effectors are proteins or small molecules present in aphid saliva which modify the structure and function of a plant cell and can ultimately promote insect virulence and survival and/or trigger plant defense response [12]. Although numerous candidate effector genes from various aphid species have been identified either at the transcriptomic or proteomic level, their expression dynamics during compatible (susceptible plant-virulent aphid) and incompatible (resistant plant-avirulent aphid) interactions remain largely unexplored.

The soybean aphid, *Aphis glycines*, is a major pest of soybean (*Glycine max* L.) in both its native Asia, as well as in North America where it is invasive [15, 16]. *A. glycines* can cause up to 58% yield loss in soybean and is estimated to have an annual economic loss of $3.6-4.9 billion on soybean production in North-America [17]. Additionally, the use of insecticides to manage *A. glycines* has led to a dramatic rise in input cost for soybean production [17, 18].

To minimize damage by *A. glycines*, host plant resistance has been a significant research focus as it can be effective, economical, and environmentally safe [2, 19]. To date, 5 major soybean genes (*Rag1*, *Rag1*c, *Rag2*, *Rag3*, and *rag4*) and 3 provisional genes (*Rag1b*, *rag3*, and *Rag5*) conferring resistance to *A. glycines* have been identified [20–23]. Among these, *Rag1*, known to exhibit both antibiosis (affecting insect biology leading to increased mortality or reduced longevity and reproduction) and antixenosis (affecting the insect behaviour leading to non-preference for feeding and colonization) has been commercialized since 2009 [19, 24]. However, prior to the commercial release of resistant varieties, virulent biotypes of *A. glycines* that can survive on HPR soybean had already been discovered. For *A. glycines*, 4 biotypes (named biotypes 1, 2, 3 and 4) are known so far, each with varying abilities to survive and reproduce on individual or pyramided *Rag* possessing soybean [25–27]. Thus, sustainable management of *A. glycines* using HPR remains a considerable challenge [19, 28].

A comprehensive understanding of the molecular interactions between soybean and *A. glycines*, at both the plant and insect level, can provide insights into the HPR mechanism and potential routes of virulence adaptation. Previous work has focused on the molecular responses of soybean to attack by *A. glycines*[29, 30], but corresponding studies on *A. glycines* are lacking. Here, we compared the molecular response of *A. glycines* when fed resistant (*Rag1*) or susceptible soybean using RNA-Sequencing (RNA-Seq) and determined whether the response was consistent with antibiosis or antixenosis. Previous electrical penetration graphs of *A. glycines* feeding behavior and soybean transcriptomic studies revealed that *Rag1*-mediated resistance is effective within the first few hours of infestation [29, 31]. *A. glycines* stylets reach sieve elements of susceptible and resistant plants in 6 h and 9 h, respectively [31], with phloem intake commencing afterwards. On resistant plants, *A. glycines* can be seen dispersing 16-24 h after infestation, most likely due to stress of plant toxins and/or non-preference. Effects of *Rag1* on *A. glycines* culminate during 24-36 h after infestation when mortality ensues, either due to PSMs, starvation, or both. Therefore, in order to have a comprehensive understanding of effects of *Rag1* resistance and to avoid capturing expression signatures occurring due to potential starvation stress, we focused on an early time point (12 h) in this interaction. Using RNA-Seq, we identified many *A. glycines* genes and gene families which are at molecular interface of its interaction with soybean and may play a critical role in virulence adaptation. Owing to high-throughput sequencing strategy, we also significantly enriched the existing transcriptomic resources for *A. glycines*, a non-model but important invasive aphid species, which will provide a foundation for future molecular studies in this insect.

## Results

### *De novo*assembly and annotation

**Figure 1:**
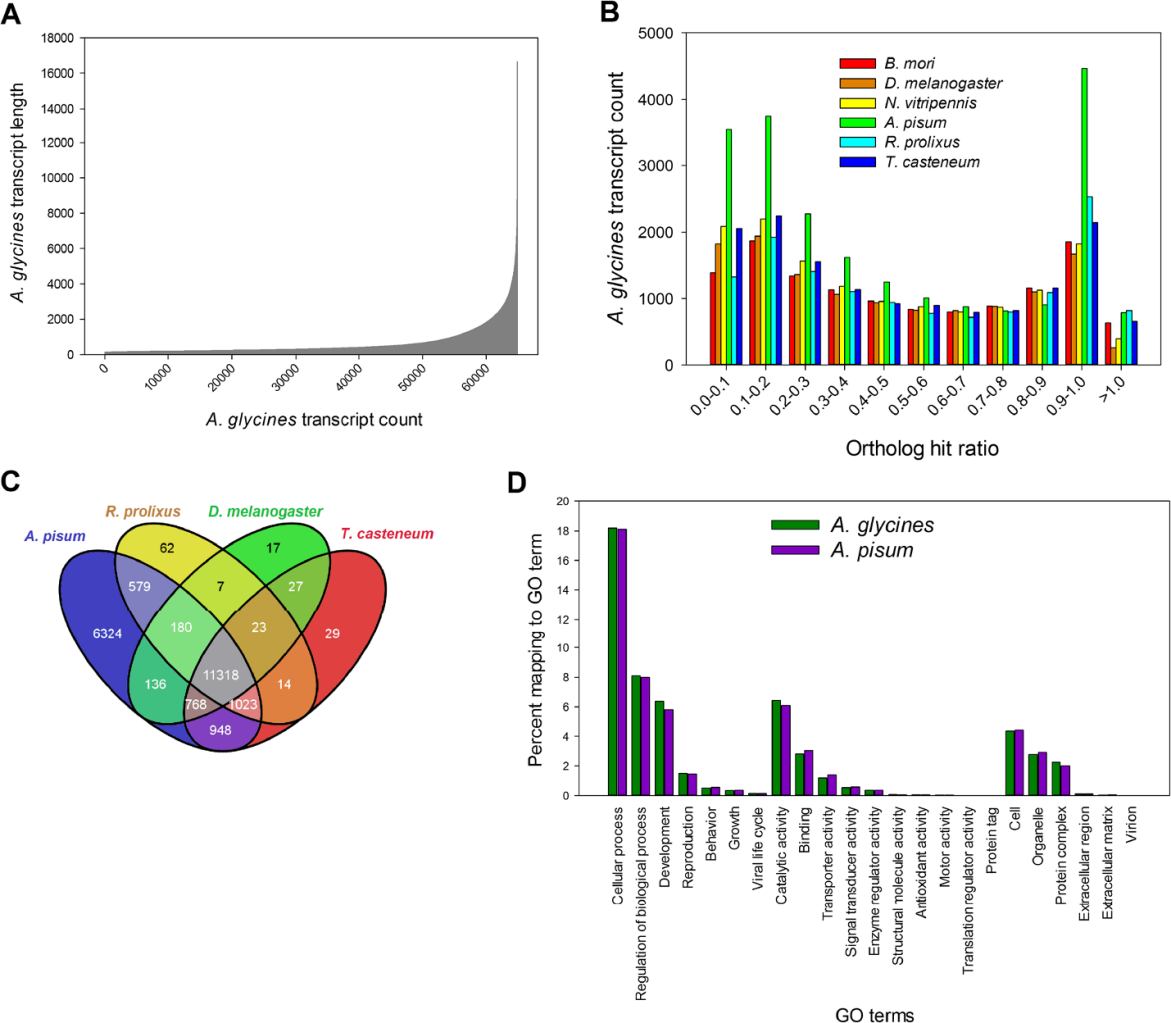
***A. glycines*** **transcriptome annotation and comparative genomics.** **(A)** Length distribution of 64,860 contigs in de novo assembly. Individual contigs are ordered on X-axis based on increasing size. **(B)** Ortholog hit ratio for transcripts calculated after BLASTx searches to genomes of *A. pisum*, *B. mori*, *D. melanogaster*, *N. vitripennis*, *R. prolixus*, and *T. casteneum* **. (C)** Venn diagram showing the number of transcript contigs with significant matches (unique and common) to genomes of *A. pisum*, *D. melanogaster*, *R. prolixus*, and *T. casteneum*. Significant matches (*e* value <1.0E-3) were calculated after pairwise comparisons (BLASTx) to each individual genome. **(D)** Comparison of GO term mappings distributions of *A. glycines* and *A. pisum* that belong to each of the three top-level GO categories (*i.e.* biological process, molecular function, and cellular component).

RNA-Seq for *A. glycines* yielded a total of 122,008,352 high quality, 76-bases paired-end reads. We pooled RNA-Seq reads with a previous transcriptome (comprising of 19,293 transcripts from 454 pyrosequencing, see [32]) to improve coverage and quality of the assembly. Using the combined dataset, *de novo* assembly of *A. glycines* produced 64,860 high quality transcripts, totaling 41,151,086 bases. The length of the transcripts varied from 150-16,670 nucleotides with an average of 634 nucleotides (Figure 1A). The assembly’s N_50_ equaled 1,164 (length N for which 50% of all bases in the assembly are located in a transcript of length L < N), which is relatively high for a non-model organism.

To determine the completeness of *A. glycines* transcriptome assembly, each transcript was compared to its putative ortholog in *Acyrthosiphon pisum*, *Bombyx mori, Drosophila melanogaster, Nasonia vitripennis, Rhodnius prolixus,* and *Tribolium casteneum*. Nearly, 50% of *A. glycines* transcripts (with a match) had an ortholog hit ratio (OHR) >0.5 (Figure 1B). Considering that an OHR value of 0 indicates a poor assembly and a value of 1 indicates a fully assembled transcriptome [33], our assembly for *A. glycines* seems to be fairly comprehensive.

Nearly 30% (19,154/64,860) of the *A. glycines* transcripts had one or more hits to protein sequences in the refseq_protein database at GenBank (complete file available upon request). The majority of the top blast hits for *A. glycines* transcripts were to insects (92.7%), whereas a small proportion showed top hits to bacteria, non-arthropod animals, plants, fungi and viruses (Additional file 1). As expected, 88.4% (of the 19,154 with a match) of top hits for *A. glycines* transcripts were to *A. pisum*, which has a well characterized genome [34]. *A. glycines* transcripts having no match may represent genes either with a novel function or whose function has not yet been designated. Interestingly, an InterProScan search revealed hits to protein signature domains for 18,832 out of the 45,706 transcripts without a match to the ref_seq protein database (41%), suggesting that many have homologs in other species that were undetected. Blastn searches with the unmatched *A. glycines* dataset (45,706 transcripts) revealed hits for 4,576 transcripts, with top hits to *A. pisum* (2,931/4,576) and *Aphis craccivora* (1,381/4,576). Nonetheless, a relatively high percentage of transcripts with ‘no match’ obtained in our study is not surprising as similar values are recorded for transcriptomes of other non-model insects [35–37].

### Comparative genomics

Using pairwise blastx searches to protein databases for four model insects, significant matches for *A. glycines* transcripts (combined = 21,455/64,860) were obtained. A blastx search to the *A. pisum* database showed matches for highest number of *A. glycines* transcripts (n = 21,295) (Figure 1C). A majority of *A. glycines* transcripts (n = 11,318) had matches to all the searched databases. However, there were a substantial number of transcripts which uniquely matched to *A. pisum* (n = 6,324), whereas only a few uniquely matched to *R. prolixus* (n = 62), *T. casteneum* (n = 29), and *D. melanogaster* (n = 17) databases.

### Functional annotation

Using blast2go, a total of 11,311 *A. glycines* transcripts were annotated. Observed gene ontology (GO) terms for each domain (biological process, molecular function and cellular component) were widely distributed into different categories. A comparison of percent mappings to each GO category between *A. glycines* and *A. pisum* revealed nearly identical distributions for both aphid species (Figure 1D). The majority of transcripts assigned to the ‘biological process’ domain were involved in cellular, regulatory, developmental, and reproductive activities, while the largest part of transcripts under ‘molecular function’ domain were predicted to have catalytic, binding and transporter functions. Through KEGG-based pathway analysis, *A. glycines* transcripts were assigned to one or more of 129 total pathways (Additional file 2). The majority of transcripts were assigned to pathways for metabolism of nitrogenous compounds (e.g. purine, pyrimidine, amino acids) and sugars (e.g. glucose). Interestingly, a total of 194 transcripts were assigned to 19 pathways for xenobiotic degradation and metabolism. Among them, transcripts encoding enzymes involved in metabolism (such as P450 enzymes) were the most abundant.

### Differential gene expression in *A. glycines*feeding on *Rag1*-soybean

**Figure 2:**
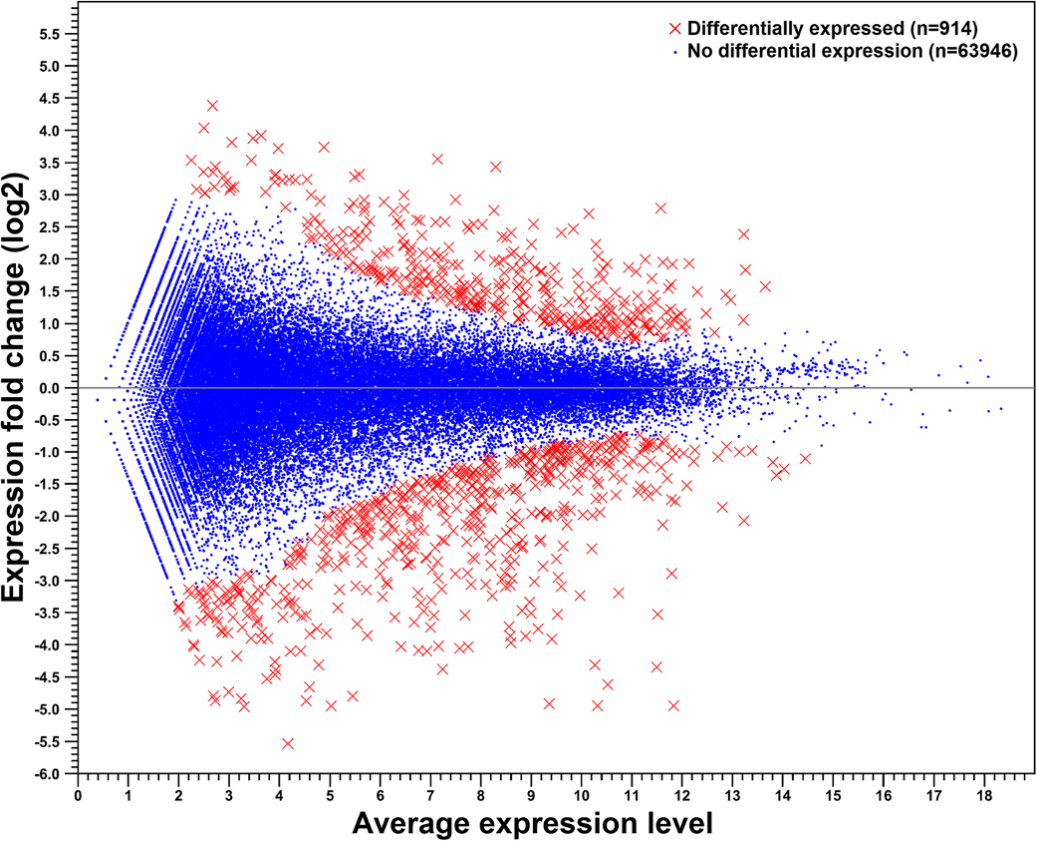
**Gene expression changes in** ***A. glycines*** **due to** ***Rag1*** **-soybean feeding.** The expression (log2 fold change) of each gene between insects fed with resistant *Rag1-*soybean and those fed with susceptible plant is plotted against average expression level of each gene in both treatments. Fold change values for gene expression were considered significant if *P* values were <0.05. See materials and methods for details.

**Figure 3:**
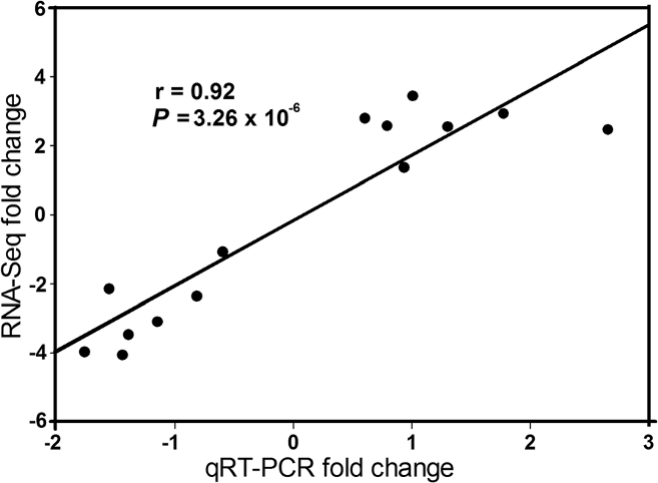
**qRT-PCR validation of RNA-Seq results.** Validation of gene expression (14 genes) using Pearson’s correlation (r) between fold changes (log2 scale) observed in qRT-PCR and RNA-Seq results.

We obtained nearly 68 and 63 million RNA-Seq reads for *A. glycines* fed with resistant (possessing *Rag1*) and susceptible soybean, respectively (Table 1). For expression measurements, 77-87% of total reads mapped to reference database genes, with nearly all reads mapping uniquely. The read depth for reference database genes varied from 0 to 284,127, with an average of 264.9 reads per gene. Statistical analysis revealed 914 (out of 64,860 reference genes) differentially expressed genes (*P* <0.05) (Figure 2). The average expression level and read depth of all differentially expressed genes are provided in Additional file 3. A total of 362 and 552 up- and down-regulated genes, respectively, were found in *A. glycines* fed with *Rag1* compared to those fed with the susceptible plant (Additional files 4 and 5). We chose 14 genes that spanned the range of differential expression and included several functional categories (based on RNA-Seq, see Additional file 6) to validate our statistical analysis with RNA-Seq. This comparison concurred with the expression pattern (either up- or down-regulated) and supported the accuracy and reliability of RNA-Seq in differential gene expression analysis (Figure 3). The GO enrichment analysis (Fisher test, agriGO) revealed 9 enriched ‘molecular function’ categories each among the up- and down-regulated genes (Additional file 7), which are detailed in following sections.

**Table 1:** **Statistics on RNA-Seq yield and read mapping**

Treatment	Replicates	Total reads	High quality reads	Mapped reads (%)	Uniquely mapped reads (%)
*A. glycines* fed with susceptible plant	R1	19,043,918	16,724,663	77.61	77.31
	R2	28,579,810	25,058,503	85.24	84.89
	R3	15,795,962	13,845,221	85.59	85.28
*A. glycines* fed with *Rag1* resistant plant	R1	21,354,822	20,804,280	84.04	83.78
	R2	11,599,306	11,298,071	87.32	87.00
	R3	35,176,984	34,258,321	86.29	85.99

### Up-regulation of genes related to xenobiotic metabolism and other stress responses

RNA-Seq analysis revealed several genes induced in *A. glycines* fed with *Rag1*-soybean potentially involved in all three phases of xenobiotic metabolism. For phase I, 13 genes related to cytochrome P450 (represented by 15 transcripts), were up-regulated. These putative P450 genes exhibited a higher transcript abundance ranging from 0.89-3.43 fold (log2 scale) in *A. glycines* on *Rag1-*soybean compared to those fed with the susceptible plant (Table 2). These genes were featured in 8 enriched gene ontology (GO) terms (Fisher test, FDR corrected *P* <0.05; Additional file 7). Nine out of 13 up-regulated putative P450 genes belonged to the CYP6 family from the CYP3 clan. For phase II, genes similar to GST, γ-glutamyltranspeptidase, COE, and sulfotransferase showed increased transcript abundance (Table 2). For phase III, transcript levels of 10 predicted ABC transporter genes, named *AyABC1* to *AyABC10*, were higher in *A. glycines* fed with *Rag1*. Genes potentially involved in the cellular uptake (scavenger receptors *AySR1*-*AySR4*) and transfer (nose resistant to fluoxetine *AyNRF1*-*AyNRF7*) of xenobiotics were also up-regulated (Table 2). Other putative stress response genes, including 9 heat shock proteins (*hsp*) and 5 takeout (*to*) genes showed higher transcript levels (Additional file 8). Up-regulated *hsp* and *to* genes exhibited fold changes (log2 scale) that ranged from 1.76-3.24 and 1.00-1.92, respectively.

**Table 2:** **Xenobiotic response and metabolism genes up-regulated in** ***A. glycines*** **fed with** ***Rag*** **1** ***-*** **soybean**

Gene	Clan/family	Transcript ID ^1^	Log2 fold Change ^2^
**Phase I- P450**			
*CYP18A1*	CYP2	contig_9694	3.43
*CYP380C10*	CYP4	contig_11996	1.28
*CYP4CJ1*	CYP4	contig_16027	2.54
		contig_16409	2.35
*CYP4CJ2*	CYP4	contig_4861	1.15
*CYP6CY7*	CYP3	contig_6786	0.89
*CYP6CY18*	CYP3	contig_10526	1.47
*CYP6CY12*	CYP3	contig_37882	2.23
*CYP6CY9*	CYP3	contig_14185	2.57
*CYP6DA2*	CYP3	contig_14387	2.02
*CYP6CY12*	CYP3	contig_14831	1.54
*CYP6CY?*	CYP3	contig_2862	1.86
*CYP6DA2*	CYP3	contig_14561	1.25
*CYP6CY9*	CYP3	contig_2861	1.46
		contig_15324	2.12
**Phase II**			
*Glutathione-s-transferase*	D1	contig_3571	1.36
*Carboxyesterase*	3	contig_18013	1.59
*γ-Glutamyltranspeptidase*	1	contig_13012	1.78
*Sulfotransferase*	C4	contig_17526	1.57
**Phase III- ABC transporter**			
*AyABC1*	G	contig_9851	2.70
*AyABC2*	G	contig_1566	0.81
*AyABC3*	G	contig_10797	1.54
*AyABC4*	G	contig_24607	1.74
*AyABC5*	G	contig_21800	1.54
*AyABC6*	G	contig_10798	1.27
*AyABC7*	D	contig_6659	0.93
*AyABC8*	G	contig_38300	1.87
*AyABC9*	G	contig_7807	1.24
*AyABC10*	G	contig_15965	1.50
***Others***			
**Scavenger receptor**			
*AySR1*	B	contig_4343	1.77
*AySR2*	B	contig_11991	1.35
*AySR3*	B	contig_13127	1.41
*AySR4*	B	contig_41556	2.21
**Nose resistant to fluoxetine**			
*AyNRF1*		contig_12910	1.81
*AyNRF2*		contig_8994	2.46
*AyNRF3*		contig_25819	1.54
*AyNRF4*		contig_2364	1.72
*AyNRF5*		contig_5415	1.06
*AyNRF6*		contig_11008	1.58
*AyNRF7*		contig_37766	2.13

^1^Nucleotide sequence for each contig is provided in Additional file 9.

^2^Fold change values for gene expression were considered significant if *P* <0.05.

### Differential expression of proteases, protease-inhibitors, and laccase-1 genes

RNA-Seq analysis revealed 6 protease-related genes having higher transcript abundance and 11 having lower transcript abundance in *A. glycines* on *Rag1*-soybean (Table 3, Additional file 4). All putative protease genes with higher transcript levels were most similar to serine proteases, and were named *AySP1* to *AySP6*. The transcript levels for these genes exhibited an increase ranging from 0.88-1.78 fold (log2 scale). The putative protease genes with lower transcript levels in *A. glycines* feeding on *Rag1*-soybean included 7 genes similar to serine proteases and 4 genes encoding carboxypeptidases with reductions ranging from 1.45-4.06 and 0.93-2.62 fold (log2 scale), respectively. *A. glycines* feeding on *Rag1*-soybean also resulted in differential expression of 4 protease-inhibitor like genes (Table 3, Additional file 9). On sequence-based homology search (blastx), these protease-inhibitor like genes showed strong matches (*e* value ranged from 0.0 to 5.31244E-80; Additional file 4) to **ser**ine **p**rotease **in**hibitors (called serpins) of other insects, and were named as *AySPI1* - *AySPI4*. Amongst these, *AySPI1* and *AySPI2* have higher transcript abundance whereas *AySPI3* and *AySPI4* have lower transcript abundance in *A. glycines* feeding on *Rag1*-soybean (Table 3). Four putative laccase-1 genes were also up-regulated in *A. glycines* on resistant plants (Table 3); transcript levels were in the range of 2.41-2.87 fold (log2 scale) greater in aphids on *Rag1*-soybean.

**Table 3:** **Differentially expressed proteases, protease-inhibitors, and laccase-1 genes in** ***A. glycines*** **fed with** ***Rag1-*** **soybean**

Gene	Transcript ID ^1^	Description	Log2 fold change ^2^
**Up-regulated**			
*Proteases*			
*AySP1*	contig_3884	Serine protease	1.17
*AySP2*	contig_6872	Serine protease snake-like	1.08
*AySP3*	contig_25003	Serine proteinase stubble-like	1.49
*AySP4*	contig_11484	Serine proteinase stubble-like isoform 2	1.78
*AySP5*	contig_4951	Venom protease-like	0.88
*AySP6*	contig_237	Venom protease-like	1.21
*Protease-inhibitors*			
*AySPI1*	contig_3885	Serpin b4-like	1.25
*AySPI2*	contig_37590	Serpin b8 isoform 2	3.32
*Laccases*			
*Aylac1*	contig_45158	Laccase-1-like isoform 1	2.87
*Aylac2*	contig_26518	Laccase-1-like isoform 1	2.74
*Aylac3*	contig_7195	Multicopper oxidase	2.41
*Aylac4*	contig_22703	Multicopper oxidase	2.59
**Down-regulated**			
*Proteases*			
*AySP7*	contig_1678	Serine protease	-1.98
*AySP8*	contig_4515	Serine protease	-4.06
*AySP9*	contig_23830	Serine protease	-3.37
*AySP10*	contig_5272	Serine proteinase stubble	-3.02
*AySP11*	contig_11924	Transmembrane protease serine 9-like isoform 1	-1.45
*AySP12*	contig_3508	Transmembrane protease serine 9-like isoform 2	-1.67
*AySP13*	contig_14346	Hypothetical protein LOC100166829	-2.73
*AyCP1*	contig_5565	Carboxypeptidase b-like	-2.07
*AyCP2*	contig_5566	Carboxypeptidase b-like	-2.62
*AyCP3*	contig_8089	Carboxypeptidase m	-0.93
*AyCP4*	contig_10493	Carboxypeptidase m-like	-1.06
*Protease-inhibitors*			
*AySPI3*	contig_6531	Serine protease serpin	-2.46
*AySPI4*	contig_5436	Serpin b10	-1.23

^1^Nucleotide sequence for each contig is provided in Additional file 9.

^2^Fold change values for gene expression were considered significant if *P* <0.05.

### Suppression of putative salivary effector gene expression

**Figure 4:**
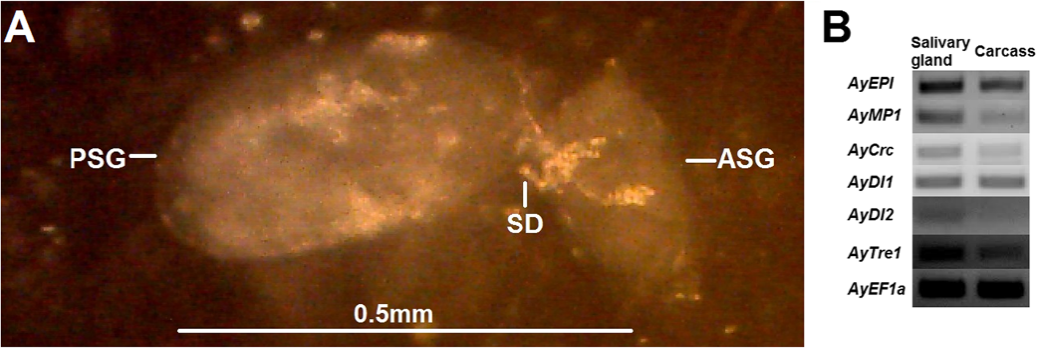
**Gene expression of effectors in salivary glands of** ***A. glycines*** **. (A)** A dissected out salivary gland from an *A. glycines* adult as viewed through a microscope. The principal salivary gland (PSG), the salivary duct (SD), and the accessory salivary gland (ASG) are indicated. **(B)** Results of semi quantitative PCR for expression analysis of effector genes in salivary gland and carcass (adult minus salivary gland and developing embryos) are shown. The PCR reactions were run for 35 cycles for all primer pairs except for *AyDI2*, where it was 40. The effector names and other details are provided in Table 4.

As effectors play a central role in the molecular interaction between aphids and their host plants [14], we focused on genes that could encode for salivary effector proteins. Currently, there is little knowledge regarding *A. glycines* effectors; however using *A. pisum* effectors as queries (see methods) we found significant hits to our transcriptome, totaling 73 putative effectors (Additional file 10). However, only 6 putative effector transcripts were differentially expressed in *A. glycines* fed with *Rag1* plant (Table 4). These 6 putative effectors showed 90-98% amino acid similarity to *A. pisum* effectors (Additional file 11, Table 4), and all were predicted to contain a secretion signal peptide at the N-terminal (Additional file 11). Our semi-quantitative PCR results confirmed effector expression in *A. glycines* salivary glands (Figure 4) as has been observed for their homologs in other aphids (references in Table 4). Interestingly, genes for these six effectors were down-regulated in *A. glycines* on *Rag1*-soybean compared to those feeding on susceptible plants; with reduction in transcript levels ranging between 0.85-4.50 fold (on log2 scale) (Table 4).

**Table 4:** **Salivary effectors genes down-regulated in** ***A. glycines*** **fed with** ***Rag1*** **-soybean**

Gene	Transcript ID ^1^	***A. pisum*** homolog	Protein identity & similarity (%)	References ^2^	Effector description ^3^	Putative function ^4^	Log2 fold change ^5^
*AyEPI1*	contig_6230	ACYPI008001	96, 97	[13]	Armet/endopeptidase inhibitor	Inhibition of plant defence proteases	-0.86
*AyMP1*	contig_7391	ACYPI009427	93, 96	[13, 38, 39]	M1 zinc metalloprotease	Deactivation of plants defence signaling peptides and dietary plant protease inhibitors in insect gut	-1.50
*AyCrc*	contig_351	ACYPI002622	96, 97	[13, 38]	Calreticulin	Inhibition of sieve tube occlusion	-1.16
*AyDI1*	contig_559	ACYPI005594	95, 97	[13]	Disulfide isomerase	Aid in gelling nature of sheath saliva by catalyzing the formation of disulphide bridges in proteins	-1.08
*AyDI2*	contig_2545	ACYPI008926	98, 98	[13]	Disulfide isomerase		-0.92
*AyTre1*	contig_8225	ACYPI002298	82, 90	[13, 40–42]	Trehalase	Suppress the activation of plant defences	-1.09

^1^Nucleotide sequence for each contig is provided in Additional file 9.

^2^Found either at RNA or protein level in salivary glands of aphids.

^3^Based on [13].

^4^Based on [13, 38, 40–47].

^5^Fold change values for gene expression were considered significant if *P* <0.05.

### Differential gene expression in starved *A. glycines*

**Figure 5:**
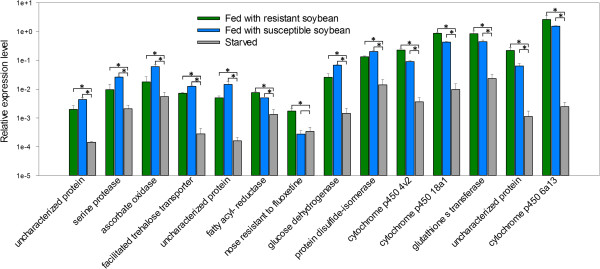
**Gene expression comparison among starved and fed** ***A. glycines.*** Bars represent the relative mRNA levels of different genes in *A. glycines* using qRT-PCR. The mean (± S.E) expression level is represented for three biological replicates for *A. glycines* fed with resistant soybean (green bars), susceptible soybean (blue bars), and starved (grey bars). The elongation factor-la (*AyEF1α*) gene was used as an internal control for cDNA [77]. More details on genes and primer sequences are provided in Additional file 12. (* *P* < 0.05).

*Rag1* leads to both antibiotic and antixenotic responses in *A. glycines*[24, 48], and the response seen in our study may also be related to starvation from the antixenotic response (i.e. non-preference). Due to the lack of a standardized and consistent artificial feeding (non-plant based) assay, we used starved aphids as a proxy to examine the molecular response that might be expected with antixenosis-induced starvation. Using qRT-PCR, we compared gene expression of the same 14 genes that encompassed a range of expression levels (Figure 3, Additional file 6) in aphids starved for 12 hr. We observed an overall pattern of reduced gene expression in starved aphids, and, in 13 out of 14 genes, the decrease was significant and expression was substantially less than what was observed when *A. glycines* was fed either *Rag1* or susceptible soybean (average reduction ranged between 3.21-1,074.52 fold, Figure 5). For example, expression of disulphide isomerase like gene (a putative effector) was reduced by ~9.5 fold after starvation, but only ~1.5 fold after feeding on *Rag1*-soybean. In addition, P450s showed decreased expression in starved aphids, which would be expected in the absence of PSMs or other stress related to plant resistance.

## Discussion

In plant-aphid interactions, initial molecular recognition and signaling events are rapid and transient [49]. To identify the key genes involved, it is vital to focus on early time points, especially in an incompatible interaction (*i.e.* a resistant plant- avirulent insect) [49]. Our gene expression data was consistent with a rapid response by *Rag1*-soybean upon infestation with *A. glycines*[29]. Expression of genes typically involved in xenobiotic (PSMs) metabolism (*e.g.* P450s and GSTs) increased, whereas a few effectors showed decreased expression. These gene expression patterns were not similar to what was observed in starved aphids (Figure 5). Overall, our results indicated an active, rapid, and specific molecular xenobiotic stress response in *A. glycines* when fed resistant soybean and are consistent with earlier studies showing rapid responses in aphid-infested soybean and the presence of, yet unidentified, PSMs.

### Feeding on resistant *Rag1*-soybean induces a xenobiotic stress response

PSMs, the defensive chemicals possessing direct toxicity to insect herbivores, are believed to occur as a complex mixture of inducers, substrates and inhibitors of insect xenobiotic response machinery [50]. We found that feeding *A. glycines* on resistant soybean resulted in the up-regulation of genes encoding for P450s, GSTs, COE, and ABC transporters (Table 2). This response is consistent with a typical xenobiotic challenge, resulting from the probable ingestion of PSMs present in *Rag1*-soybean, and supports the ‘antibiosis’ mode of HPR [48]. This increase was not due to starvation, as all P450’s were down regulated in starved *A. gylcines* (Figure 5). The involvement of some of these P450s and ABC transporters in other biological functions cannot be ruled out as these occur as large gene families known to perform multiple biological functions.

In *Rag1-*soybean, no specific PSM toxic to *A. glycines* has been reported due to a lack of metabolomics studies. However, a microarray-based study on *Rag1*-soybean responses to *A. glycines* feeding identified 17 differentially expressed genes for secondary metabolism [29]. Interestingly, 14 out of the 17 genes were related to the phenylpropanoid pathway (PPP) and encoded for homologs of chalcone synthase, isoflavone synthase, and a flavanone 3-hydroxylase-like protein. In plants, the PPP is a rich source of PSMs for defense against insect herbivores [51]. Soybean PPP produces PSMs like flavones, isoflavones, isoflavanones, and anthocyanins which can be potentially toxic to *A. glycines*[52]. Besides phenylpropanoids, phenolics appear to provide another layer of defense in *Rag1*-soybean, as indicated by the induction of laccase-1 genes in *A. glycines* (Table 3). Insect laccases are copper-containing enzymes which tend to detoxify plant phenolics through oxidation reactions [9].

Among 13 induced P450 genes in *A. glycines* feeding on *Rag1*-soybean, 9 belonged to the CYP6 family of CYP3 clan (Table 2). Members in this family have previously shown similar responses in other insects, and are specifically involved in metabolism of numerous PSMs. For example, CYP6B enzymes detoxify PSMs in *Papilio polyxenes*, *P. multicaudatus*, and *Helicoverpa zea*[50, 53, 54]. Interestingly, the induction of P450 and other xenobiotic metabolism genes revealed in current study occurred in an incompatible interaction (resistant plant-avirulent insect). In fact, the induction of xenobiotic response genes by PSM exposure is thought to be the first step leading to the eventual detoxification and virulence adaptation because mutations responsible for higher enzymatic potency toward a xenobiotic substrate are more likely to be selected if these occur in inducible genes (that overproduces the enzyme) rather than in constitutive genes [55]. Our findings strongly indicate a vital role for P450s in the coevolutionary history and apparent ‘arms race’ between *A. glycines* and soybean, and future investigation into role of xenobiotic response machinery may reveal adaptation mechanism in virulent biotypes.

### Suppression of putative salivary effector gene expression

Among 73 putative *A. glycines* effectors identified in this study, 47 matched to *A. pisum* effectors with known function (Additional file 10). Based on homology, these effectors seem capable of performing diverse biological functions at the interface of aphid-plant interactions. Further, the down-regulation of 6 effectors in *A. glycines* fed with resistant plants (Table 4) seems to be specific, as expression of other putative infection-promoting effectors (*e.g.* peroxidase, cathepsin, serine carboxypeptidase) remain unchanged (Additional files 4 and 10). The mechanism of suppression after feeding on *Rag1-*soybean is unclear and may likely involve different possibilities. First, microRNAs (miRNAs) in *Rag1-*soybean may down-regulate *A. glycines* effector genes directly. Aphid resistant plants show differential expression of many conserved miRNA families upon aphid infestation compared to susceptible plants [56]. Furthermore, aphid tissues contain several plant miRNAs which are ingested during feeding on resistant plants [57]. The capability of microRNAs to perform cross-kingdom regulation (*e.g.* plant miRNAs regulating the expression of mammalian genes [58]) further supports a potential role of miRNAs regulating *A. glycines* gene expression.

Second, suppression of effector genes may be a by-product of the rapid induction of the xenobiotic metabolism machinery. Initially, to reach sieve element cells, aphid stylets follow an extracellular path surrounded by epidermal, mesodermal and parenchyma cells [14]. However, along the way, stylets puncture these cells (to assess their internal chemistry) and secrete saliva. Plants can then recognize aphid infestation and mount immediate defense responses in surface cells, even before stylets reach the sieve element cells. For example, after aphid infestation, *VAT (*for melon resistance to *Aphis gossypii*) and *Mi-1.2* (for tomato resistance to *Macrosiphum euphorbiae*) based resistance is ubiquitous in various cell types [59, 60]. In fact, a broad, ubiquitous resistance expression is a typical feature of NBS-LRR family genes in plants [61]. There is a strong evidence that *Rag1*, also predicted to be a member of NBS-LRR family [29], mounts its defense ubiquitously in surface cellular layers [31]. Since the defense response is so rapid, shutting down salivary effector expression may lead to more efficient xenobiotic metabolism, resulting in a molecular trade-off. This may also explain the difference in magnitude of putative effector expression between aphids on resistant plants and starved aphids. Having no access to plants for a longer time period, starved aphids likely initiated an earlier and stronger suppression of gene expression (Figure 5). However, with a lack of a consistent artificial diet assay, it may be difficult to disentangle the effects of starvation.

Third, the resistance factors encountered in the sieve elements of *Rag1*-soybean may be responsible for decreased effector expression in *A. glycines*. This possibility is supported by the observation that aphid stylets stay for only 2.7 min in sieve elements of *Rag1*-soybean, as opposed to 18.9 min in susceptible plant [31] which ultimately results in reduced salivation. Otherwise, effector secretion is a continuous phenomenon for aphids on susceptible plants [62].

### Protease and protease-inhibitor gene regulation against *Rag1*defence

As a part of defence against herbivores, plants deploy protease inhibitors which target insect digestive proteases and suppress enzymatic activities [63]. To combat this, herbivorous insects exhibit elevated levels of inhibitor-insensitive and/or reduced levels of inhibitor-sensitive proteases [64]. The observed differential expression of proteases in *A. glycines* feeding on the resistant plant (Table 3) occurred in response to the elevated levels of protease inhibitors in *Rag1*-soybean after aphid infestation [30]. However, modified *A. glycines* protease activity may have undesirable effects as it can be harmful to critical gut structures [65], in addition to the potential damage caused by plant proteinases [43, 44]. Thus, in order to protect itself from internal and external proteinases, it is likely that *A. glycines* differentially regulates protease inhibitors, as observed in this study (Table 3).

## Conclusion

Soybean with *Rag1* resistance induced the expression of genes encoding various stress response proteins such as P450s, GST, COE, ABC transporter, and HSP in *A. glycines*. Furthermore, feeding on *Rag1*-soybean resulted in the down-regulation of genes for putative effectors that were found in *A. glycines* salivary glands. The overall response in *A. glycines* due to *Rag1* feeding resembled that of a characteristic xenobiotic challenge, which supported the ‘antibiosis’ mode of *Rag1* HPR being mediated through toxic PSMs. The genes identified here will be prime candidates to investigate *A. glycines* biotype evolution.

## Methods

### Plant and insect source

Two soybean lines, LD05-16060 (carrying *Rag1* resistance to biotype 1 of *A. glycines*) and SD01-76R (the susceptible near-isoline of LD05-16060) were used. LD05-16060 was developed through backcrossing twice the variety Dowling (*Rag1*) [66] into the background of SD01-76R. The pedigree of LD05-16060 is SD01-76R(2) x Dowling x Loda.

To prepare cDNA libraries for RNA-Seq and to perform subsequent qRT-PCR validation, *A. glycines* were obtained from a biotype 1 laboratory colony that originated from insects collected from Urbana, IL (40° 06′ N, 88° 12′ W) in 2000 [67]. These aphids are defined as being avirulent to all known *Rag* genes. At the Ohio Agricultural Research and Development Center (OARDC) Wooster, OH, a laboratory population of these insects is maintained on susceptible soybean seedlings (SD01-76R) in a rearing room at 23-25°C and a photoperiod of 14:10 h (Light:Dark). All *A. glycines* in this OARDC colony are descended from 1 single founding female and represent 1 clonal lineage to limit variation from multiple genetic backgrounds.

### *A. glycines*feeding on resistant (*Rag1*) and susceptible soybean

To obtain newborn nymphs, *A. glycines* adults (apterate females) were transferred (using a camel hair brush) and allowed to feed on detached trifoliate soybean leaves (SD01-76R) in a petri dish [68]. After 2 h, the newly hatched nymphs of *A. glycines* were delicately transferred onto intact first trifoliate leaves of LD05-16060 and SD01-76R whole plants, grown in separate pots. Following the transfer, infested leaves were isolated with a small snap cage to restrict the insect movement. Snap cages contained holes covered with wire mesh to allow for proper ventilation and maintenance of optimum growth conditions. Nymphs were allowed to feed on respective plants for 12 h. Following the feeding, insects were collected in a 1.5 ml eppendorf tube and were immediately frozen at -80°C. Nymphs fed with 3 separate plants of identical soybean line (LD05-16060 or SD01-76R) were pooled to constitute one biological replicate of each treatment. Nearly 60-70 nymphs were collected for each replication and there were three biological replications for each treatment.

### RNA extraction and RNA-Seq libraries preparation

Insect samples were processed for total RNA extraction using PureLink® RNA Mini Kit (Life Technologies Corporation, Carlsbad, CA, US), following the manufacturer’s protocol. To remove DNA contamination, samples were treated with PureLink® DNase (Life Technologies Corporation, Carlsbad, CA, US). RNA quality was checked using a Nanodrop 2000c (Thermo Scientific, Hudson, NH, US) and an Agilent Bioanalyzer 2100 (Agilent Technologies, Palo Alto, CA, US). The cDNA libraries for RNA-Seq were prepared using the TruSeq RNA Sample Preparation Kit (Illumina Inc., San Diego, CA, US), following the manufacturer’s protocol. Briefly, 4 μg of total RNA for each sample was used to purify and fragment mRNA (library insert fragmentation at 94°C for 8 min to give an insert of 155 bp; range 120-210 bp), followed by first and second strand cDNA synthesis. Then, a series of steps including end-repair (to convert the overhangs resulting from fragmentation into blunt ends), adenylation of 3′ ends of the blunt fragments (to prevent them from ligating to one another during the adapter ligation reaction), ligation of adapters to the ends of double stranded cDNA, and PCR amplification to enrich DNA fragments with adapters were performed. Unique adapter sequences were included for each of the three biological replicates from each treatment. The high quality of the libraries was confirmed using a high sensitivity DNA chip on Agilent Bioanalyzer 2100 (Agilent Technologies, Palo Alto, CA, US). The libraries for 3 biological replicates of each treatment were pooled together, and the pooled sample from each treatment was sequenced in two lanes of a Genome Analyzer II flow cell (Illumina Inc., San Diego, CA, US). The paired-end sequencing was performed at Molecular and Cellular Imaging Center (MCIC), OARDC, Wooster, OH, USA.

### Raw sequencing data processing

For sample-wise allocation of the sequencing data, the raw reads from each lane of flow cell were demultiplexed using the respective index sequence. Initial processing of sequencing data was performed using MCIC galaxy tools available at http://www.oardc.ohio-state.edu/mcic/. Briefly, fastq quality scores of reads were converted from ‘Illumina1.5′ type to ‘Sanger’ type using FASTQ Groomer (version 1.0.4) [69]. The adapter sequences were removed from sequencing reads using ‘cutadapt’ (version 0.9.5.a) tool [70]. The quality check on sequencing reads was performed using ‘fastqc’ tool (http://www.bioinformatics.babraham.ac.uk/projects/fastqc/). Further, reads were trimmed using ‘Trim the reads by quality’ (version 1.2.2) tool (Phred quality cutoff of 20 and minimum read length of 40 nucleotides). All sequence data were deposited in the GenBank under the BioProject accession PRJNA231526.

### *De novo*assembly construction and functional annotation

The *de novo* assembly and subsequent gene expression analyses were performed using CLC Genomics Workbench version 6.02 (CLC Bio, Aarhus, Denmark). In addition to the Illumina RNA-Seq data from this study, previously published Roche 454 cDNA transcripts [32] were used as input for assembly. Assembly proceeded using the following parameters: word size of 24, bubble size of 50, and minimum contig size of 150 bases. The *A. glycines* transcriptome was annotated using Blast2GO, which implemented BLASTx searches (*e* value <1.0E-3) between all *A. glycines* contigs and the NCBI Reference Sequence database (Refseq_protein) [71]. Following the mapping step, gene ontology (GO) terms with *e* value <1.0E-6, annotation cut-off >55, and GO weight >5 were used for annotation. To categorize the GO terms into different GO categories, CateGOrizer, was used, along with the ‘GO_slim’ classification [72]. The GO categories for *A. glycines* were compared to those from *A. pisum*, available at http://www.b2gfar.org/showspecies?species=7029. The *A. glycines* contigs that showed no match to the Refseq_protein database were searched using BLASTn (*e* value <1.0E-3) for hits to the non-redundant nucleotide (nt) database at NCBI. Functionally enriched GO terms in the differentially expressed gene dataset (see below) were identified through the singular enrichment analysis (Fisher test; Yekutieli FDR corrected *P* <0.05) in agriGO (http://bioinfo.cau.edu.cn/agriGO/) [73]. To find the pathways in which putative proteins of *A. glycines* transcriptome are involved, analysis of Kyoto Encyclopedia of Genes and Genomes (KEGG) was performed using Blast2GO [74]. For comparative genomics, pairwise BLASTx searches (*E* value <1.0E-3) between *A. glycines* contig sequences and genomes of model insect species (*A. pisum*, *B. mori*, *D. melanogaster*, *N. vitripennis*, *R. prolixus*, *T. casteneum*) were performed. Results of these blastx searches were also used to calculate the ortholog hit ratio (at OARDC MCIC galaxy, http://www.oardc.ohio-state.edu/mcic/).

### Differential gene expression analysis

To obtain the gene expression values, reads from each sample replicate were mapped to the genes (*i.e.* assembly contigs) with the default mapping parameters {minimum similarity fraction = 0.8 and minimum length fraction (long reads) = 0.9} [75]. Statistical comparison of expression values from both treatments was conducted using bootstrapped receiver operating characteristic (bROC) algorithm, available as an integrated plug-in in CLC Bio genomics workbench. To avoid problems with infinite values, the expression values were transformed as log2(E + 1); where E is the original expression value. The expression data was normalized using median of M-values (MMV) method. While calculating fold change for gene expression changes, expression values for *A. glycines* fed with susceptible soybean (SD01-76R) were taken as reference. Fold change values for gene expression were considered significant if *P* values were <0.05.

### qRT-PCR on starved *A. glycines*and for validation of RNA-Seq data

To starve *A. glycines*, newly hatched nymphs were placed in petri dishes containing only moist filter paper for 12 h. For RNA-Seq data validation, insect samples were collected as described above for RNA-Seq library preparation. Samples were processed for RNA extraction and first strand cDNA synthesis as described previously [76]. Specific primers for each gene were designed using Beacon Designer version 7.0 (Premier Biosoft, Palo Alto, CA) (Additional file 12). Due to their consistent expression, *TBP* and *EF1a* were used as internal controls, using previously described conditions [77]. There were 3 biological and 2 technical replications for each gene validated. Relative expression level and fold change were determined using comparative Ct method (2^-∆∆Ct^) [78]. Statistical analysis was performed using t-test through MeV package, version 4.9 available at http://www.tm4.org.

### Identification and validation of salivary effector genes

Initially, to identify *A. glycines* salivary effector transcripts, *A. pisum* effector protein sequences [13] were used as the query in a tblastn search (top hit *e value* cut-off: 1E-20; bit score cut-off: 250) among the differentially expressed *A. glycines* genes. Identity of putative *A. glycines* effector transcripts was confirmed by blastx search at NCBI-GenBank. Subsequently, identified transcripts were filtered out based on 5 criterions to validate their salivary effector nature: 1) minimum 90% similarity of encoded proteins to *A. pisum* effectors; 2) expression in salivary glands as revealed by semi-quantitative PCR (method is described below); 3) presence of secretion signal peptide in encoded proteins as revealed by signalP version 4.1. [79]; 4) absence of transmembrane domain in encoded proteins as revealed by TMHMM server version 2.0 [80]; and 5) presence of signature domains in encoded proteins as revealed by an interProScan search which were inspected manually (e.g. contig_7391’s coding region contained signature motifs for peptidaseM1 (IPR001930) and metalloprotease (PTHR11533) activities). Due to the minuscule size of nymphs, adult *A. glycines* were used to dissect out salivary glands (10 days old) in phosphate-buffered saline (pH 8.0). Both salivary gland and carcass (adult minus salivary glands and developing embryos) samples (50 individuals each) were processed for RNA extraction, DNA-ase treatment and first strand cDNA synthesis as described previously [77]. The primer sequences are given in Additional file 12. Each RT-PCR reaction was performed with 1 *μ*l of cDNA (100 ng /*μ*l), 0.5 *μ*M of each primer, and 10 *μ*l of PCR master mix (from Promega) in a 20-*μ*l total volume. The PCR amplifications were done with the following cycling conditions: one cycle at 95°C (3 min), followed by 35-40 cycles of denaturation at 95°C (30 s), annealing and extension at 55°C for 30 s.

### Availability of supporting data

All sequence data were deposited in the GenBank under BioProject accession PRJNA231526. Additionally, the nucleotide sequence for each contig described in this study is provided in Additional file 9.

## Electronic supplementary material

Additional file 1: **Summarization of top hit organisms in blastx search for** ***A. glycines*** **transcripts.** (ZIP 56 KB)

Additional file 2: **Putative KEGG pathways assignments for** ***A. glycines*** **transcripts.** (XLSX 71 KB)

Additional file 3: **Average expression (normalized) level and read depth for differentially expressed genes.** (XLSX 44 KB)

Additional file 4: **Annotation details for up-regulated and down-regulated genes in** ***A. glycines*** **fed with** ***Rag1*** **-soybean.** This file has two spreadsheets; one each for up-regulated and down-regulated gene dataset. (XLSX 96 KB)

Additional file 5: **Summary of annotation statistics for up- and down-regulated gene dataset.** (ZIP 126 KB)

Additional file 6: **Visual representation of genes (indicated in red) chosen for qRT-PCR validation.** Based on the expression level distribution, we chose 14 genes that supposedly represented the majority of differentially expressed genes. (ZIP 63 KB)

Additional file 7: **Enriched GO terms among up- and down-regulated genes.** (XLSX 12 KB)

Additional file 8: **Up-regulated stress response genes in** ***A. glycines*** **fed with** ***Rag1-*** **soybean.** (DOCX 26 KB)

Additional file 9: **Fasta file for contig sequences of genes described in this study.** (DOCX 157 KB)

Additional file 10: **Putative effector genes identified from** ***A. glycines*** **transcriptome.** (XLSX 16 KB)

Additional file 11: **Pairwise sequence alignment for salivary effector proteins of** ***A. glycines*** **and** ***A. pisum.*** In the alignment of each effector protein, upper and lower lanes represent the *A. glycines* and *A. pisum* sequences respectively. At the N-terminal, putative secretion signal peptide regions are underlined. If the corresponding amino acid residues are identical, it is indicated by dots for *A. pisum.* (ZIP 912 KB)

Additional file 12: **Gene names and primer sequences used in this study.** (DOCX 35 KB)

## Abbreviations

HPR: Host plant resistance
PSM: Plant secondary metabolite.

## References

[1] Tilmon KJ (2008). Specialization, Speciation, and Radiation: the Evolutionary Biology of Herbivorous Insects.

[2] Smith CM (2005). Plant Resistance to Arthropods: Molecular and Conventional Approaches.

[3] Van Emden HF, Harrington R (2007). Aphids as Crop Pests/edited by Helmut F. van Emden and Richard Harrington.

[4] Smith CM, Chuang W (2014). Plant resistance to aphid feeding: behavioral, physiological, genetic and molecular cues regulate aphid host selection and feeding. Pest Manag Sci.

[5] Li X, Schuler MA, Berenbaum MR (2007). Molecular mechanisms of metabolic resistance to synthetic and natural xenobiotics. Annu Rev Entomol.

[6] Xu C, Li CY, Kong AT (2005). Induction of phase I, II and III drug metabolism/transport by xenobiotics. Arch Pharm Res.

[7] Misra JR, Horner MA, Lam G, Thummel CS (2011). Transcriptional regulation of xenobiotic detoxification in *Drosophila*. Genes Dev.

[8] Jongsma M, Beekwilder J (2011). Co-evolution of insect proteases and plant protease inhibitors. Curr Protein Pept Sci.

[9] Prasain K, Nguyen TD, Gorman MJ, Barrigan LM, Peng Z, Kanost MR, Syed LU, Li J, Zhu KY, Hua DH (2012). Redox potentials, laccase oxidation, and antilarval activities of substituted phenols. Bioorg Med Chem.

[10] King-Jones K, Horner MA, Lam G, Thummel CS (2006). The DHR96 nuclear receptor regulates xenobiotic responses in *Drosophila*. Cell Metab.

[11] Cai Q, Han Y, Cao Y, Hu Y, Zhao X, Bi J (2009). Detoxification of gramine by the cereal aphid *Sitobion avenae*. J Chem Ecol.

[12] Hogenhout SA, Bos JI (2011). Effector proteins that modulate plant–insect interactions. Curr Opin Plant Biol.

[13] Carolan JC, Caragea D, Reardon KT, Mutti NS, Dittmer N, Pappan K, Cui F, Castaneto M, Poulain J, Dossat C (2011). Predicted effector molecules in the salivary secretome of the pea aphid (*Acyrthosiphon pisum*): a dual transcriptomic/proteomic approach. J Proteome Res.

[14] Elzinga DA, Jander G (2013). The role of protein effectors in plant–aphid interactions. Curr Opin Plant Biol.

[15] Tilmon K, Hodgson E, O’Neal M, Ragsdale D (2011). Biology of the soybean aphid, *Aphis glycines* (Hemiptera: Aphididae) in the United States. J Int Pest Manag.

[16] Ragsdale DW, Landis DA, Brodeur J, Heimpel GE, Desneux N (2011). Ecology and management of the soybean aphid in North America. Annu Rev Entomol.

[17] Hodgson E, McCornack B, Tilmon K, Knodel J (2012). Management recommendations for soybean aphid (Hemiptera: Aphididae) in the United States. J Int Pest Manag.

[18] Ragsdale DW, Voegtlin DJ, O’Neil RJ (2004). Soybean aphid biology in North America. Ann Entomol Soc Am.

[19] Hesler LS, Chiozza MV, O’Neal ME, MacIntosh GC, Tilmon KJ, Chandrasena DI, Tinsley NA, Cianzio SR, Costamagna AC, Cullen EM (2013). Performance and prospects of *Rag* genes for management of soybean aphid. Entomol Exp Appl.

[20] Hill CB, Li Y, Hartman GL (2006). A single dominant gene for resistance to the soybean aphid in the soybean cultivar Dowling. Crop Sci.

[21] Hill CB, Li Y, Hartman GL (2006). Soybean aphid resistance in soybean Jackson is controlled by a single dominant gene. Crop Sci.

[22] Mian MR, Kang S, Beil SE, Hammond RB (2008). Genetic linkage mapping of the soybean aphid resistance gene in PI 243540. Theor Appl Genet.

[23] Zhang G, Gu C, Wang D (2010). A novel locus for soybean aphid resistance. Theor Appl Genet.

[24] Diaz-Montano J, Reese JC, Schapaugh WT, Campbell LR (2006). Characterization of antibiosis and antixenosis to the soybean aphid (Hemiptera: Aphididae) in several soybean genotypes. J Econ Entomol.

[25] Kim K, Hill CB, Hartman GL, Mian M, Diers BW (2008). Discovery of soybean aphid biotypes. Crop Sci.

[26] Hill CB, Crull L, Herman TK, Voegtlin DJ, Hartman GL (2010). A new soybean aphid (Hemiptera: Aphididae) biotype identified. J Econ Entomol.

[27] Alt J, Ryan-Mahmutagic M (2013). Soybean aphid biotype 4 identified. Crop Sci.

[28] Bansal R, Jun T, Mian M, Michel AP, El-Shemy HA (2013). Developing Host-Plant Resistance for Hemipteran Soybean Pests: Lessons from Soybean Aphid and Stink Bugs. Soybean - Pest Resistance.

[29] Li Y, Zou J, Li M, Bilgin DD, Vodkin LO, Hartman GL, Clough SJ (2008). Soybean defense responses to the soybean aphid. New Phytol.

[30] Studham ME, MacIntosh GC (2013). Multiple phytohormone signals control the transcriptional response to soybean aphid infestation in susceptible and resistant soybean plants. Mol Plant-Microbe Interact.

[31] Diaz-Montano J, Reese JC, Louis J, Campbell LR, Schapaugh WT (2007). Feeding behavior by the soybean aphid (Hemiptera: Aphididae) on resistant and susceptible soybean genotypes. J Econ Entomol.

[32] Bai X, Zhang W, Orantes L, Jun T, Mittapalli O, Mian MR, Michel AP (2010). Combining next-generation sequencing strategies for rapid molecular resource development from an invasive aphid species. Aphis glycines PLoS One.

[33] O’Neil ST, Dzurisin JD, Carmichael RD, Lobo NF, Emrich SJ, Hellmann JJ (2010). Population-level transcriptome sequencing of nonmodel organisms *Erynnis propertius* and *Papilio zelicaon*. BMC Genomics.

[34] Consortium IAG (2010). Genome sequence of the pea aphid *Acyrthosiphon pisum*. PLoS Biol.

[35] Mittapalli O, Bai X, Mamidala P, Rajarapu SP, Bonello P, Herms DA (2010). Tissue-specific transcriptomics of the exotic invasive insect pest emerald ash borer (*Agrilus planipennis*). PLoS One.

[36] Chen Y, Cassone BJ, Bai X, Redinbaugh MG, Michel AP (2012). Transcriptome of the plant virus vector *Graminella nigrifrons*, and the molecular interactions of maize fine streak rhabdovirus transmission. PLoS One.

[37] Bai X, Mamidala P, Rajarapu SP, Jones SC, Mittapalli O (2011). Transcriptomics of the bed bug (*Cimex lectularius*). PLoS One.

[38] Nicholson SJ, Hartson SD, Puterka GJ (2012). Proteomic analysis of secreted saliva from Russian Wheat Aphid (*Diuraphis noxia* Kurd.) biotypes that differ in virulence to wheat. J Proteome.

[39] Carolan JC, Fitzroy CI, Ashton PD, Douglas AE, Wilkinson TL (2009). The secreted salivary proteome of the pea aphid *Acyrthosiphon pisum* characterised by mass spectrometry. Proteomics.

[40] Atamian HS, Chaudhary R, Cin VD, Bao E, Girke T, Kaloshian I (2013). In planta expression or delivery of potato aphid *Macrosiphum euphorbiae* effectors Me10 and Me23 enhances aphid fecundity. Mol Plant-Microbe Interact.

[41] Rao SA, Carolan JC, Wilkinson TL (2013). Proteomic profiling of cereal aphid saliva reveals both ubiquitous and adaptive secreted proteins. PLoS One.

[42] Cui F, Michael Smith C, Reese J, Edwards O, Reeck G (2012). Polymorphisms in salivary gland transcripts of Russian wheat aphid biotypes 1 and 2. Insect Science.

[43] Pechan T, Ye L, Chang Y, Mitra A, Lin L, Davis FM, Williams WP, Luthe DS (2000). A unique 33-kD cysteine proteinase accumulates in response to larval feeding in maize genotypes resistant to fall armyworm and other Lepidoptera. The Plant Cell Online.

[44] Mohan S, Ma PW, Williams WP, Luthe DS (2008). A naturally occurring plant cysteine protease possesses remarkable toxicity against insect pests and synergizes *Bacillus thuringiensis* toxin. PLoS One.

[45] Will T, Tjallingii WF, Thönnessen A, van Bel AJ (2007). Molecular sabotage of plant defense by aphid saliva. Proc Natl Acad Sci.

[46] Will T, Kornemann SR, Furch AC, Tjallingii WF, van Bel AJ (2009). Aphid watery saliva counteracts sieve-tube occlusion: a universal phenomenon?. J Exp Biol.

[47] Darby NJ, Penka E, Vincentelli R (1998). The multi-domain structure of protein disulfide isomerase is essential for high catalytic efficiency. J Mol Biol.

[48] Li Y, Hill CB, Hartman GL (2004). Effect of three resistant soybean genotypes on the fecundity, mortality, and maturation of soybean aphid (Homoptera: Aphididae). J Econ Entomol.

[49] Thompson GA, Goggin FL (2006). Transcriptomics and functional genomics of plant defence induction by phloem-feeding insects. J Exp Bot.

[50] Feyereisen R, Feyereisen R, Gilbert LH (2012). Insect CYP Genes and P450 Enzymes. Insect Molecular Biology And Biochemistry.

[51] Dixon RA, Achnine L, Kota P, Liu C, Reddy M, Wang L (2002). The phenylpropanoid pathway and plant defence—a genomics perspective. Mol Plant Pathol.

[52] Zabala G, Zou J, Tuteja J, Gonzalez D, Clough S, Vodkin L (2006). Transcriptome changes in the phenylpropanoid pathway of Glycine max in response to pseudomonas syringae infection. BMC Plant Biol.

[53] Mao W, Berhow MA, Zangerl AR, Mcgovern J, Berenbaum MR (2006). Cytochrome P450-mediated metabolism of xanthotoxin by Papilio multicaudatus. J Chem Ecol.

[54] Puinean AM, Foster SP, Oliphant L, Denholm I, Field LM, Millar NS, Williamson MS, Bass C (2010). Amplification of a cytochrome P450 gene is associated with resistance to neonicotinoid insecticides in the aphid Myzus persicae. PLoS Genet.

[55] Despres L, David J, Gallet C (2007). The evolutionary ecology of insect resistance to plant chemicals. Trends Ecol Evol.

[56] Sattar S, Song Y, Anstead JA, Sunkar R, Thompson GA (2012). Cucumis melo microRNA expression profile during aphid herbivory in a resistant and susceptible interaction. Mol Plant-Microbe Interact.

[57] Sattar S, Addo-Quaye C, Song Y, Anstead JA, Sunkar R, Thompson GA (2012). Expression of small RNA in Aphis gossypii and Its potential role in the resistance interaction with melon. PLoS One.

[58] Zhang L, Hou D, Chen X, Li D, Zhu L, Zhang Y, Li J, Bian Z, Liang X, Cai X (2011). Exogenous plant MIR168a specifically targets mammalian LDLRAP1: evidence of cross-kingdom regulation by microRNA. Cell Res.

[59] Dogimont C, Bendahmane A, Chovelon V, Boissot N (2010). Host plant resistance to aphids in cultivated crops: genetic and molecular bases, and interactions with aphid populations. C R Biol.

[60] Goggin FL, Jia L, Shah G, Hebert S, Williamson VM, Ullman DE (2006). Heterologous expression of the Mi-1.2 gene from tomato confers resistance against nematodes but not aphids in eggplant. Mol Plant-Microbe Interact.

[61] McHale L, Tan X, Koehl P, Michelmore RW (2006). Plant NBS-LRR proteins: adaptable guards. Genome Biol.

[62] Giordanengo P, Brunissen L, Rusterucci C, Vincent C, Van Bel A, Dinant S, Girousse C, Faucher M, Bonnemain J (2010). Compatible plant-aphid interactions: how aphids manipulate plant responses. C R Biol.

[63] Habib H, Fazili KM (2007). Plant protease inhibitors: a defense strategy in plants. Biotechnol Mol Biol Rev.

[64] Bown DP, Wilkinson HS, Gatehouse JA (1997). Differentially regulated inhibitor-sensitive and insensitive protease genes from the phytophagous insect pest, Helicoverpa armigera, are members of complex multigene families. Insect Biochem Mol Biol.

[65] Ge Z, Wan P, Cheng X, Zhang Y, Li G, Han Z, Bell J (2013). Cloning and characterization of serpin-like genes from the striped rice stem borer. Chilo suppressalis Genome.

[66] Li Y, Hill CB, Carlson SR, Diers BW, Hartman GL (2007). Soybean aphid resistance genes in the soybean cultivars Dowling and Jackson map to linkage group M. Mol Breed.

[67] Hill CB, Li Y, Hartman GL (2004). Resistance to the soybean aphid in soybean germplasm. Crop Sci.

[68] Michel AP, Mian MR, Davila-Olivas NH, Cañas LA (2010). Detached leaf and whole plant assays for soybean aphid resistance: differential responses among resistance sources and biotypes. J Econ Entomol.

[69] Blankenberg D, Gordon A, Von Kuster G, Coraor N, Taylor J, Nekrutenko A, Galaxy Team (2010). Manipulation of FASTQ data with Galaxy. Bioinformatics.

[70] Martin M (2011). Cutadapt removes adapter sequences from high-throughput sequencing reads. EMBnet journal.

[71] Conesa A, Götz S, García-Gómez JM, Terol J, Talón M, Robles M (2005). Blast2GO: a universal tool for annotation, visualization and analysis in functional genomics research. Bioinformatics.

[72] Zhi-Liang H, Bao J, Reecy J (2008). CateGOrizer: a web-based program to batch analyze gene ontology classification categories. Online J Bioinformatics.

[73] Du Z, Zhou X, Ling Y, Zhang Z, Su Z (2010). agriGO: a GO analysis toolkit for the agricultural community. Nucleic Acids Res.

[74] Ogata H, Goto S, Sato K, Fujibuchi W, Bono H, Kanehisa M (1999). KEGG: Kyoto encyclopedia of genes and genomes. Nucleic Acids Res.

[75] Mortazavi A, Williams BA, McCue K, Schaeffer L, Wold B (2008). Mapping and quantifying mammalian transcriptomes by RNA-Seq. Nat Meth.

[76] Bansal R, Mian M, Mittapalli O, Michel AP (2013). Molecular characterization and expression analysis of soluble trehalase gene in Aphis glycines, a migratory pest of soybean. Bull Entomol Res.

[77] Bansal R, Mamidala P, Mian MR, Mittapalli O, Michel AP (2012). Validation of reference genes for gene expression studies in Aphis glycines (Hemiptera: Aphididae). J Econ Entomol.

[78] Schmittgen TD, Livak KJ (2008). Analyzing real-time PCR data by the comparative CT method. Nat Protoc.

[79] Petersen TN, Brunak S, von Heijne G, Nielsen H (2011). SignalP 4.0: discriminating signal peptides from transmembrane regions. Nat Methods.

[80] Krogh A, Larsson B, Von Heijne G, Sonnhammer EL (2001). Predicting transmembrane protein topology with a hidden Markov model: application to complete genomes. J Mol Biol.

