# Supplementary figures and images for: RNA-Seq reveals a xenobiotic stress response in the soybean aphid, Aphis glycines, when fed aphid-resistant soybean
Source: BMC Genomics. 2014 Nov 16;15(1):972. doi: 10.1186/1471-2164-15-972 (PMC4289043; doi:10.1186/1471-2164-15-972)

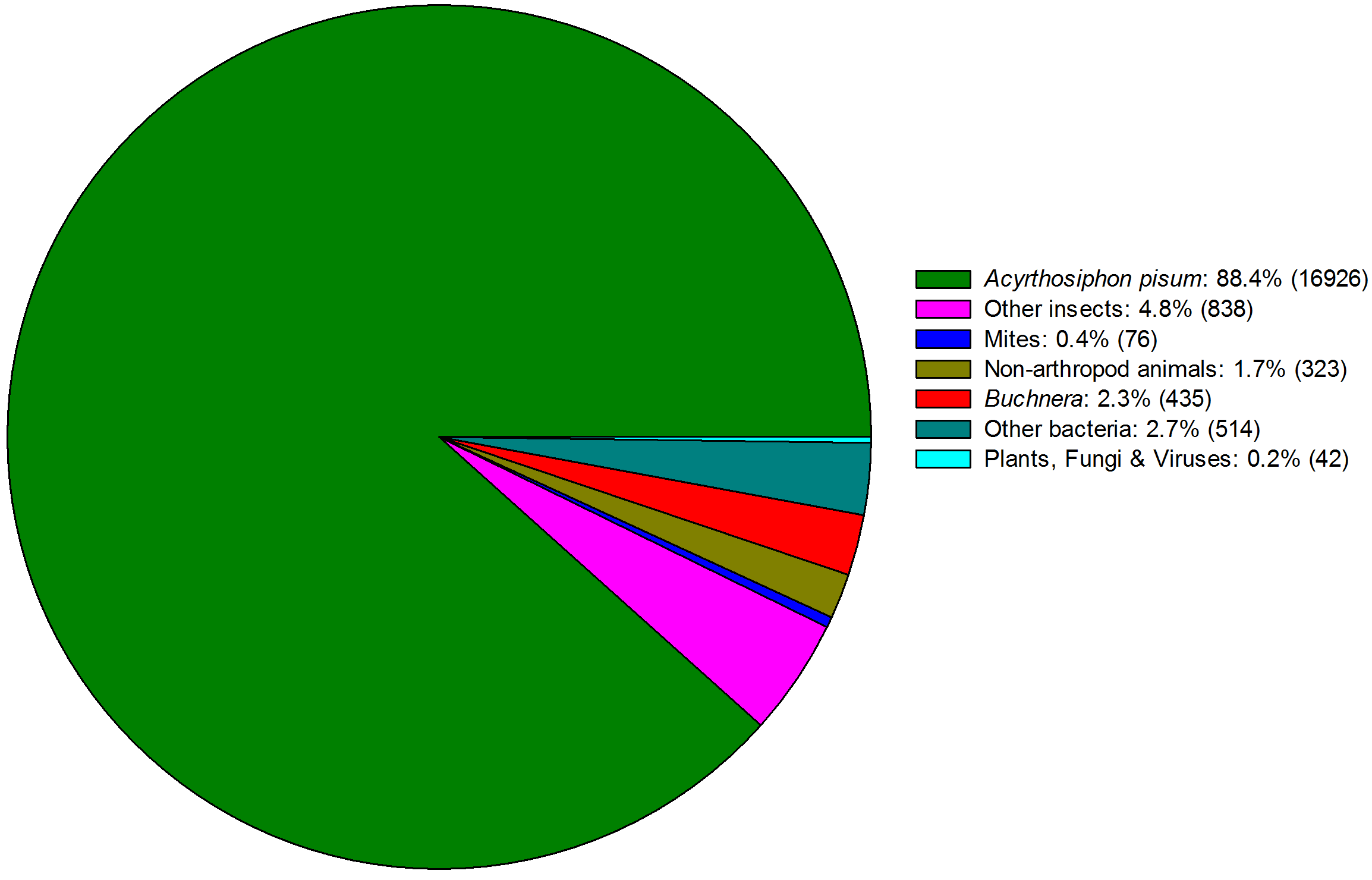

Supplement: Supplementary file 1 — Additional file 1: Summarization of top hit organisms in blastx search for A. glycines transcripts. (ZIP 56 KB) [file 12864_2014_6855_MOESM1_ESM.zip › 1838969456139057_MOESM1_ESM.bmp]

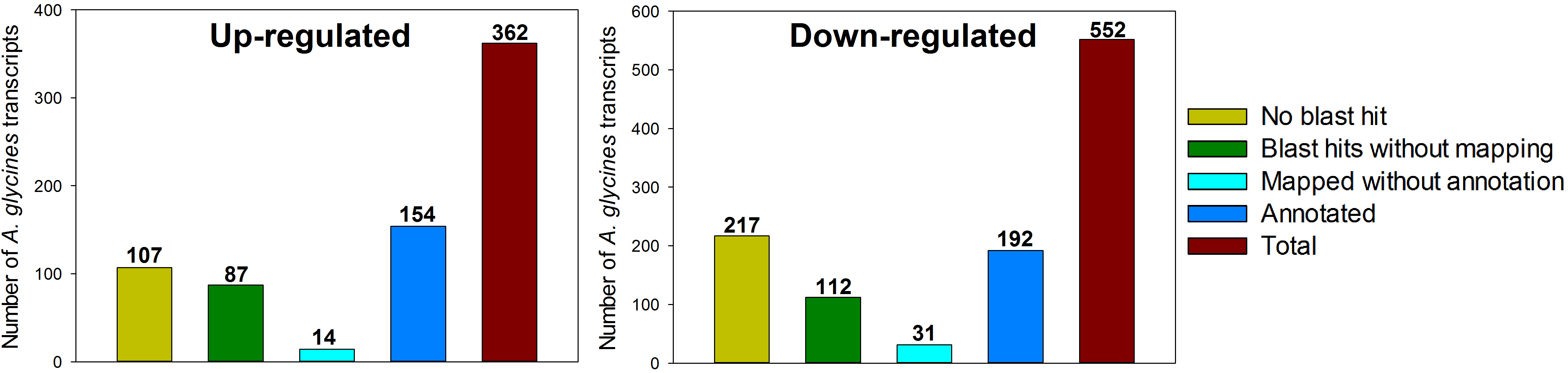

Supplement: Supplementary file 5 — Additional file 5: Summary of annotation statistics for up- and down-regulated gene dataset. (ZIP 126 KB) [file 12864_2014_6855_MOESM5_ESM.zip › 1838969456139057_MOESM5_ESM.bmp]

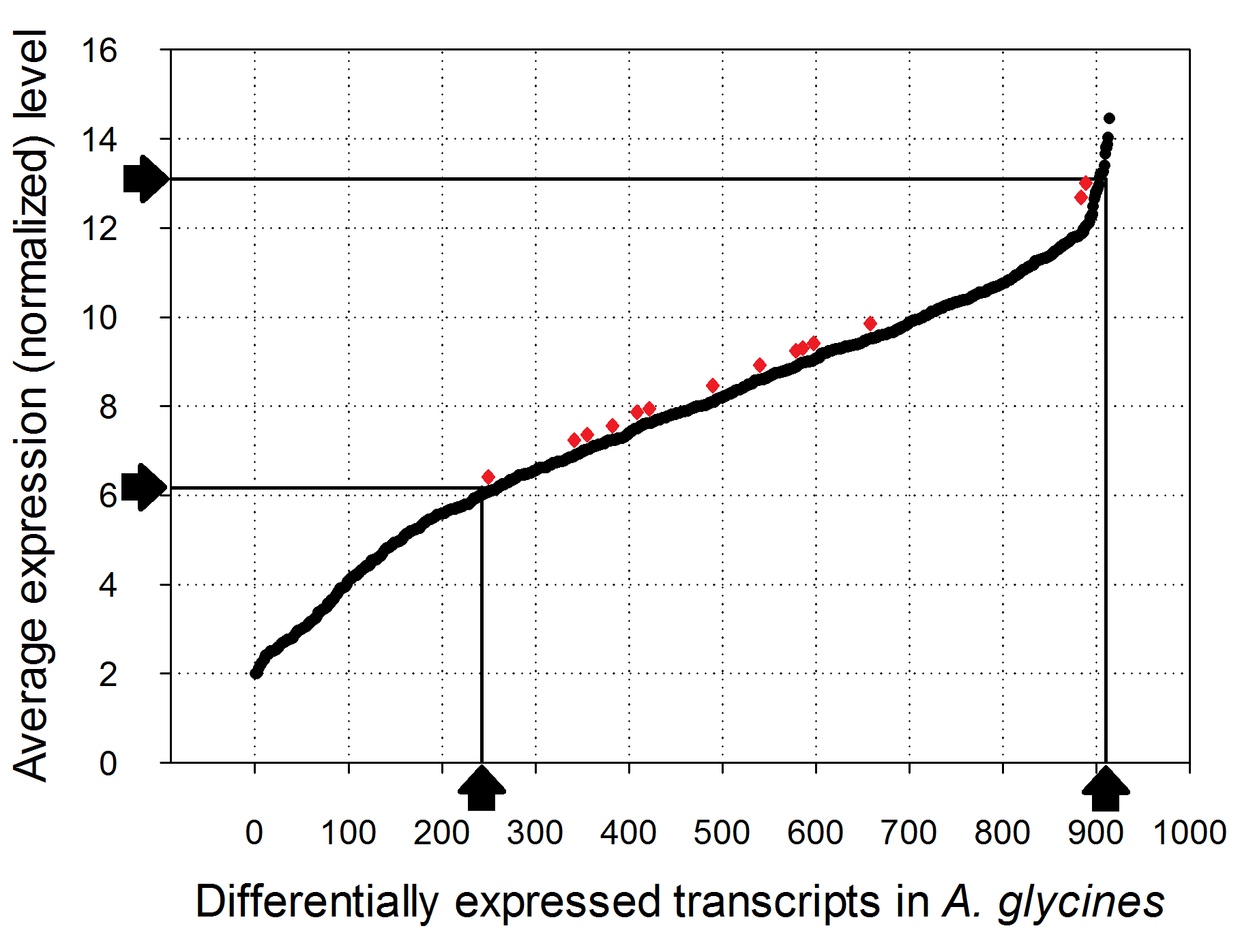

Supplement: Supplementary file 6 — Additional file 6: Visual representation of genes (indicated in red) chosen for qRT-PCR validation. Based on the expression level distribution, we chose 14 genes that supposedly represented the majority of differentially expressed genes. (ZIP 63 KB) [file 12864_2014_6855_MOESM6_ESM.zip › 1838969456139057_MOESM6_ESM.bmp]

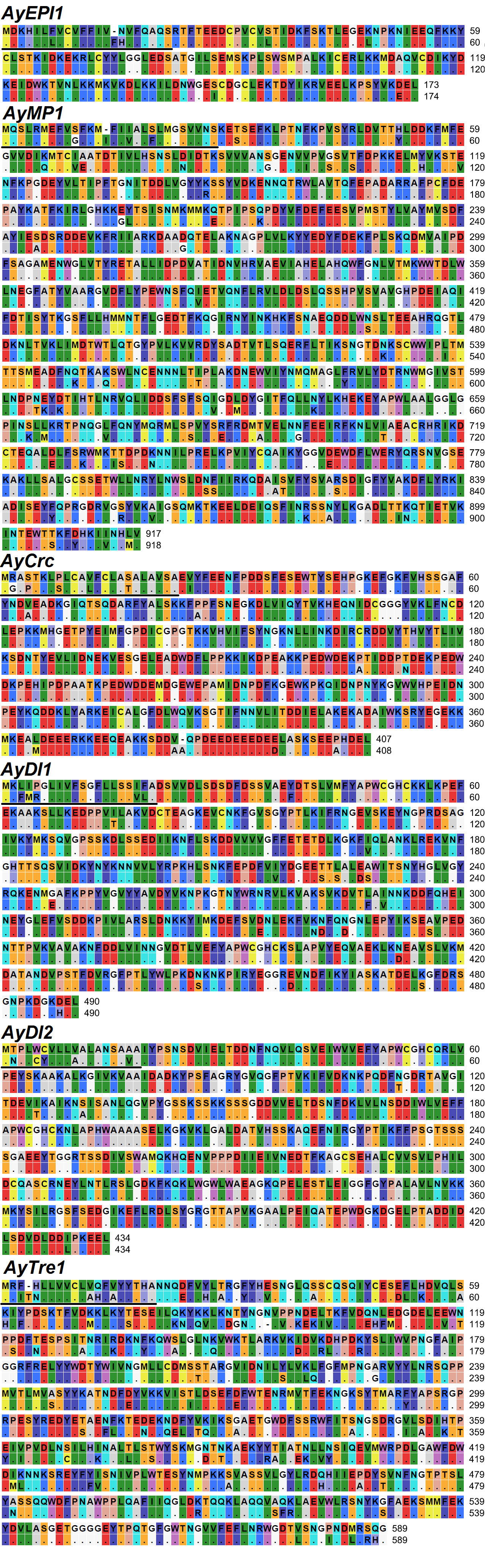

Supplement: Supplementary file 11 — Additional file 11: Pairwise sequence alignment for salivary effector proteins of A. glycines and A. pisum. In the alignment of each effector protein, upper and lower lanes represent the A. glycines and A. pisum sequences respectively. At the N-terminal, putative secretion signal peptide regions are underlined. If the corresponding amino acid residues are identical, it is indicated by dots for A. pisum. (ZIP 912 KB) [file 12864_2014_6855_MOESM11_ESM.zip › 1838969456139057_MOESM11_ESM.bmp]
