# Supplementary material for: RNA-Seq reveals a xenobiotic stress response in the soybean aphid, Aphis glycines, when fed aphid-resistant soybean
Source: BMC Genomics. 2014 Nov 16;15(1):972. doi: 10.1186/1471-2164-15-972 (PMC4289043; doi:10.1186/1471-2164-15-972)
Supplement: Supplementary file 8 — Additional file 8: Up-regulated stress response genes in A. glycines fed with Rag1- soybean. (DOCX 26 KB) [file 12864_2014_6855_MOESM8_ESM.docx]

**Up-regulated stress response genes in *Aphis glycines* fed with *Rag1*-soybean**

| **Transcript ID^1^** | **Description** | **Log2 fold change^2^** |
| --- | --- | --- |
| contig_49128 | heat shock 70 kda protein 1b-like | 2.39 |
| contig_19037 | heat shock cognate 71 kda | 3.24 |
| contig_8514 | heat shock protein 70 | 1.98 |
| contig_7196 | heat shock protein 70 | 1.76 |
| contig_29020 | heat shock protein 70 | 2.59 |
| contig_12137 | heat shock protein 70 b2 | 2.54 |
| contig_7197 | heat shock protein 70 b2 | 2.33 |
| contig_11634 | heat shock protein 70 b2-like | 2.04 |
| contig_57301 | heat shock protein 70 b2-like | 2.81 |
| contig_6123 | protein takeout | 1.92 |
| contig_27856 | protein takeout-like | 1.72 |
| contig_5822 | protein takeout-like | 1.00 |
| contig_12791 | protein takeout-like | 1.31 |
| contig_27288 | protein takeout-like | 1.25 |
| contig_8346 | c-type lectin 5 precursor | 1.78 |

^1^ Nucleotide sequence for each contig is provided in Additional File 12.

^2^ Fold change values for gene expression were considered significant if *P* < 0.05.
