# Supplementary material for: RNA-Seq reveals a xenobiotic stress response in the soybean aphid, Aphis glycines, when fed aphid-resistant soybean
Source: BMC Genomics. 2014 Nov 16;15(1):972. doi: 10.1186/1471-2164-15-972 (PMC4289043; doi:10.1186/1471-2164-15-972)
Supplement: Supplementary file 9 — Additional file 9: Fasta file for contig sequences of genes described in this study. (DOCX 157 KB) [file 12864_2014_6855_MOESM9_ESM.docx]

>contig_1007

CGACAGTCGGCGGGGACATTGGACGTCAACCATTGGCCATCGTAGCGATACGTCGTATCACCATCAATATAATTTATAGT

GTGTACGTTTACACCTGGTAGTTTTTCTTTTTATCGTTGAGAGAATTACGTGACGTGTTACAAACGTTAAGGCGATATTA

TATTGCTATCTTAGGGTTCCGTTAGGTCGACGTCGTAGGCCAATATTTTGATTATTCAAATTATGTAAATTAACCCATAA

CCGCACCATGCTCCCAGAGTAAATGTTCGATTTATGTACTACCCGAAACAACAATAATGGCCGGCCAAAAGTTTCAGTAC

GATGAAAGCGGAGCTACGTTTTTCTATTTTTTATTGTCGTTTCTGGCTCTTCTGCTGATACCGGGCACGTATTACTGGTG

GCCGAGAAAACAAAAGGAAGATCCAAAAGCGTTGGCATCTGAATGTTATTGTCCAGGATGTCGAAAAAAAAAGCTTGTCC

TGCAGTCCACTAAACCGTGGGAACTGATGAAAAGAACATGCATAAAAGTCATCATTATTGGTGGATGGGTGTTATTGGCT

TTTCTTGCCTACAAAGTATCCCAGTTTGATTATGAGAGTGCAAGTTTTGATCCATATGACATTCTCAATGTTCCAATTGG

AACCAGTGAAAAAGTAATTAAAAAAGCTTATAGAAGGTTGTCATTAATTTACCATCCAGATAAGGAAACTGGAGATGAAA

AAAAATTTATGAAGCTTACTAAAGCTTATCAAGCCTTAACTGATGATGAGTCCAGAAAAAATTGGGAAAAATATGGAAAT

CCAGATGGACCTGGTGCAATGAGTTTTGGAATAGCATTACCATCATGGATTGTAGAAAAAGAAAATTCAGTTTGGGTCTT

AGGTCTATATGCATTAGTTTTTATGATTGCACTTCCTACTACAGTTGGTATGTGGTGGTATAAATCTATCAGATATTCTG

GTGATAAGGTTTTATTAGAGACCTCTAGAATGTATTATTATTTTTTACATAAGTCAAATTCTATACCTTTGAAACGAGTT

ATAATGATTTTAAGTGCTTCATTAGAGTTTGAAAAAAAGTATAATAGTGAAATAGTTGAACGATCTTCAGATGAATATGA

AATGCCTCAACTTATAAAACAACTTTATAATTTTGGAGAAAAGGCACAAAAAGAACCACCACTGTCTTATAATTATTCAA

TTAAAGCTAGAGCTTTGATACATGCACATCTGTCTCGCCTGGTACTTAAGGCAGATACATTAGATTTAGATCGTATGTAC

ATAGTTAAGAAATGTCCATATTTAATACAAGAAATGGTTGGTGTTGTTTCTCAACTAATTTTACTAGCATATGCTCAAAG

AGTTCCTCGTTTGCTTAATATTGAAACTATAGAAAACTGTATGAAACTTTGTCCCATGGTGGTCCAAGCATTATGGGAGT

ATAAAAGTCCATTTATGCAATTACCATACATTACTGAGGATCACATAAAACATTTTGACAATAAAAAGAAACGTGTTAGG

AGTATTCAGCATCTTGCTCAATTAAAAAATGAAGACAGGCGAAGCACGTTAAAATTTTTATCTGATAGTCAGTATGAAGA

TTTAGTTAAAGTATTGGGCAGAATGCCCTATATAGACTTTAAAGTTCGTTGTGAAGTTATTGATGATGAAGCTACAACAG

TCTATACAGCTGGAGCTATAGTAACCGTAACTGTGCAGTTAAAAAGACGAGATATGAGGGTATTGTTTGGAGATGATTCA

TATGCAGATAAACATCATATTGAGCTAGAAAAAAATGCTGCTAAAGAAACAGAAAAACCAGATAAGTTAGAGAAACCTAT

GTCTAATGAAGATGTTATAGAAAATAGTGAAAATGTTGAAAATAAAGAATTAAAAGATGATGAAAAAGAAAAGAAAGCAA

CTAAGGGTATTTGGCAGCAAAAGCGCCAATCAGGAAAAGGTAAAAAAAGTGGAAATGTCAAAAAACAAAAAGCTACTTTA

TCAGCATCAGCTAAGAAAAAAATTAAAAAGAAAGAAAAATTAGAAAAAGCAAAATTAGGAGATTCAGAAATTAAAGAAAC

TGAAGATAAAGAACAATTAAAAGATAATAATGTTTCATCAGATGATGACTCTGATTCAGATAATGAAAGTTCTAGTGATG

CTTCTGGTGACGAATCAGAGAAAACAGATGCTGAAGAAACAACAGATGATAAAAAAAAGGTTTCAGCAAATAATGATGAC

GATGATGATGATGATTGGGATAAATTTCAATCTGGAGCCAATAAAAAAGATAGAGTACTTGAAGGCAGAACTAAACAATC

ACATTTGGTCCACTCTCCATATTTTCCTGAAGAAAAACACGAATATTGGTGGGTTTATTTAAGTGATCGGAAAAGTCGTA

CATTATTGACTGTTCCATATCATGTCACTGAACTTGTTGATGAAGAAGAAATTCAATTAAAATTTACAGCTCCTAGGTGG

CCTGGAGTTTATGTTTTTGCCGTTTGTCTGAGATCGGATTCATACTTTGGTTTTGACCAAATGCATGATATCAAATTGGA

TGTAAAAGAAGCACCAGAACCTTTAACAGAACATCCTCAGTGGGATATTTCTGATGAAGAAGATGACACTAAAGAAGAAG

ATAAGCAAAGTGATATTTCAGAATTTACTACAGATGAAGATGTCGAAGATTAAATAATACTAACATATAAAACCTTTGGG

TGACCTGAAAAACAAAGAAGTTCAAAACAATAACTAGAGTTTAAAGAGTCAAATATCACATTTCAATAATAATCATAATC

ATATATGTGAAATAAGTCAGTTATAGCAGAAGAAAATTATTATTTAATACACAGAAATGAGAAATGAATTAAAAAAATTA

TTTGGAAACATACCTCAGATTCATCACCTTTTTCGATTTTCTGTACAGAATCATCAGTAACAATTAAATGATTGTAAGCT

GGCAAGAAACTTAATTTTGATGTACAATCAGAACATATCATTTCAGAATAATCATCTGGATTCATATCTTTGGTGCCCAA

ATGCTTGCTATGAAACCAATCTTCACAAACAATGCATTGTATCATCTCATCAGTATTTTCAGGATCAGGGTATGGACGTT

GACAAATGCAGTATAAACCCTTAAAATTTTGATTGTAAATGTTACGGTCATTTGCTTCAGGTTTTAACGGTTCCAATTGA

CACTCTGATGTTAGCTTATCACTTCCACAGTCACATCGATAATTCCTTTTAGTCCATAACTCAACCAAATCATGGTCTTC

ATGGCATTGGTACATACAGGGCAAACATACTGCACCAGGTAACTTATCTTTTTCAGTGCAAGTTAAACATGCATACAACG

CTTGTCGATTCATATATCCCTGAGAATAAGTGCAACTATCACTTTCGGACGCCTCAAGTATCAGATTGGCTTGTTCGATC

TCAGCATTTTGATCGTTGATGTACTCGACAAGCCCGACAACCTGATCCTCTTCGTTTTCGTTGAAAGAATTACCCTCAGA

CTCGGACATGATTGACAAGCGATCGAGATTACGACGCAACACACACGACACACGTCGATTACAATAATAAGCAACCAACA

GCAACTAACGGGTATCGACTGTAAAAGTCTTTGCTTAGGATCAGCAATCCAGCGTTCAGCGCTTTCAGTTTCACAAACCG

CCACTAGTACGTCGATTATAGCTTACAGTTCAGTGTTCACTGTTGGTCTGTTGAAACACTTGAAACTTTTAACTTCTAAT

AGTCGTTACCGCTTACCACAGAATCGTATTTAATGCGCATGCGTGCGAATAGCTGTGTGTGTATTTATTTTGTATACTGT

TTTTTGACTTTTTTCTGTACATGGTTATTTTTATGTGGTTATTTATTGTTTATGTCGCTCAGCAGGATGTTTACCTAAAT

GTTG

>contig_10493

GACTAATCGCGAACGATGGCGCTTTCCACCGCGACGACGTGCCGGGCGAGAGCTGACGGTCGTCGCTCGCATTTGCTGCT

ATCTGTTCGGACATTGGTCGTGGTTACGATCGCGGTCACACTTTTCGCGCTGCCGATTCATTGTCAATCTGAAAAAAGGC

TATCGTCCATCCAACAGAAACAGCAACAACACCAATACTACGATTCGGAGGAGCTGAATGGCGGCGGCGAGGTGATCAAG

AGCAAAATTCGACCAGATTCGTATTCCCAAGATCCATCATCCTCACAACAATACGAAAAGCATTTGCAACACGGCAAGCA

ATCGGACTCGGTCGGTTCAGCATCCGCCGTGTACGGTGAACAGTATGGTGATGGAAGCGCTCTGATGCAGCAGGATCAAC

CACTGTTGGATGAAGAGGCCCAGGAGTCGATCAGCTCGGGTTCGATAAACAATCACAGGATACCGCAGTATACGCGCAAC

GGATCTCCTGTGTCTGGTGATGTGATCGAGGGCAAGAATGTGGGCAACCGAGCCGTTGGAAACAACTACCATCATCCTGG

TAACCCATCACCAGCCTCCGGTGGTTATGCGGTTGACTCAGCATATGGCCCGCACTACAGCTCCCAGGACCTGGTCCAAG

ACCAAAACTATCAGCTTGAATTTAAATACCACGACTACGATAAGATGACCAAGTTCCTGAGGACCACATCATCTCGGTTC

CCAAACCTCACAGCTCTCTATTCCATCGGAAAATCCGTCCAAGGTCGTGACTTGTGGGTCATGGTGGTGTCTTCCAGTCC

GTACGAACACATGATTGGCAAGCCAGATGTCAAATACGTAGCCAATATGCACGGAAACGAAGCGGTTGGCAGAGAGCTCA

TGTTACATCTTATTCAGTATTTGGTGAACAGTTATTCGGTGGACCCATACATCCGATGGCTTCTGGACAACACTCGCATT

CACGTGTTGCCGTCAATGAACCCAGACGGTTTCGAGGTAGCCCGTGAAGGTCAGTGCGACGGTGGTCAAGGACGGTACAA

CGCACGCGGTTTTGACTTGAACCGCAACTTCCCAGACTACTTCAAGCAAAACAACAAGAGAGGACAACCGGAAACTGATG

CCGTCAAGGAATGGACGTCTAAGATACAGTTCGTTCTGTCCGGTGGACTTCACGGGGGTGCCTTAGTGGCAAGCTATCCG

TTCGATAATACACCCAACTCAA

>contig_10494

GCTGCCTCCATCGTCTGTTGATCTTTGCCAACCATCAGGATTCATCGACGGCATTAGGTGAATCCTAGTTTTAGTAATTA

ATTTTACAATATCCTCGTTTCCGGCTTTATACTCCTCACAAAAGTAATCCGCTAATTTCAATAGTAACTCCCTTCCAAGT

ACTTCATTACCGTGCATGTTAGCTATATATTTAAACTCTGGATCCATTGGCTTATGCTCAGTTGGATGAACTGAAAATAC

GAGGACTAAAAGTGGATGGCCTTCTACAGAGTCCTCCGATAGCCTATACAGACTAGTGATATTTGGGCATTTGTCGCGCA

CTTGCAAAATGGCATCGTACATTTCTTCATTATTGTGATGTTTGAATTGAAATTCCTTTCGTTCAGATGAATTCACATTG

ATCGCAAAAGCGGCTAACCACAATAAAGCCCACATTGACGTTGCGGGTGATCCGAACAGGATTTGTTTATTGATCATTGT

AGTTCGTCCATAAGACATTGCAGCATTTAATTTCTCTGGTGTGCTTACTGAATTATTATGATTTTATTTAACAGTTTTCT

GGAATGGAGTGTAACTGCGGATAACTTATACAAAGCGGTATAACGTCCAGCTGGCTATAACAGAATGGCAATGCAAGCTC

TCGGTCTGAGCATGCTCAAAACGTGTATAGAGCAAGTCCGTCCCACCCACCGGTGCTTCTACAATAATATATATTGATTT

CACACCAAGTGTATATTATTACGTCAAGACATTCAAA

>contig_10526

GAATACAGTTTTAACTTTCGCGTGTATTGAGTTAGTCGTATTTTGTATTTTTCATACAATGTTTGCGTCGATCTCCATTT

GGTGGATAAGTATAATAACACTATGTCTACTAATTTTTACAATTATTCACTATTTTTGTACTTATACATTTAATAAATGG

GACAAAATCAACGTGCCTTATGTTCAGCCAATTCCACTATTTGGTAACTTCTTCTATGTAGCTTTTGGAATGCAACATCC

CATAGATTTCTACAGGAAAATTTATTATGACCTGGCTGGTCATAAATATGGAGGTTTATTTCAGATGAGAACCCCATATT

TGATGATCCGTGACCCGGAAATAATAAACAATGTTCTTATCAAAGACTTTTCATATTTCCCTAATCGTGGTATTTACTCA

GATTTCTCAGCGAATCCCCTGTCGAATCAACTATTTTTCATGGAAAATCCCCAATGGAAGTTAATCAGAAAAGCGTTGAG

CCCAGCGTTCACTTCAGGCAAACTCAAGCTAATGTACGATCAAATCAAAGAGTGTAGTGATGAACTAATGAAAAATATCC

AAAAAAACTTCATGAAAATCGACGATAAAATAGAAGTGCGAGATATGTTGGGCAAATATTCAACCGACGTTATCGGCACT

TGCATTTTTGGTTTAAAATTAAATGCCGTGAGCGATGATAATTCTACGTTTCGAAAATATGGCAAGTCTATATTTGTCCC

GTCACTGAGAACACATTTAAGAGAATTGAGTCTTATGATCAGTCCTACTCTTTTGAATATCCTTAAATTTAAGGATTTCC

CAGCGGACGCCACTGAGTTTTTCCATTCAGCATTTCATGAAACGATAACGTACAGGGAAAAAAACAACATAGTCAGAAAT

GATTTTGTTCAAACTTTGATACAAGCGAGAAACGATTTGGTTTTAAACAAAAGTATACCTCAAGGAGAAAGATTTTTAGA

GTCACAAATTATTGCCAATGCATTTGTTATGTTTGCTGCTGGATTTGAAACCGTATCTACTGCTATGAGTTTTTGTTTAT

ACGAGCTAGCGTTGAAGAAACATATTCAAGACAGAGTACGCCAAGAAATTAATTTGAAATTATCGAAAAACAATGGACTG

ATTAATAACGAATTTTTGATCGAATTAAATTACTTAGATATGATTTTAGCAGAAACACTTCGTAAATATCCACCAACCTT

TGCTTTATTCAGAAAAGCATCACAAACATATCACGTACCAAATGATTCATTAACTATTGATAAGGACCAAAAAATTATAA

TTCCTATTTATTCACTGCATTACGATCCAAAGTATTTTACGGATCCTGAAGTATTTGATCCGGAAAGATTTTCACCCGAA

GAAAAAGCTAAACGTATAAGTGGTACTTATCTTCCATTTGGCGATGGACCTCGAATTTGTATAGGAAAACGTTTTGCTGA

GTTGGAAATGAAATTGGCTTTAGTTGAAATTCTGACTAAATTTGAAGTAGAACCATGTGAAAAAACTGAAATTCCTATAC

GATTAAGTAAAAAGGCTATAGTTGCAATGCCAGAAAATGGTATTTGGCTAACATTTAAAAAAATTACTTGCTAATGATAA

TAGATAAGTAAGTGATTTTGTTAATAGAATGCATTATCAATTTGTTAATTTTACTTATTAATATTTTAAAATATTTTTCA

AAATATGGATGTATATAAGTAGTGTTAACATACGTAAATTGTGTGCTTTATTTAATAATTTTAATATAG

>contig_10543

AATAAAATTAATTAAACAATTAGTTATTAGATAACAAGAAAAAGGATTATTAAAATCATAATATTTTAAGTGCCTGTAGT

CTGTCTATATTGCAATAATTCGTACTCGTTTAAAATTAAGTCGTACATTTTATTTCAAACAACATCGTCCATGCTGACAT

CGTTCAAATGACGCGGATAATAAAAACGTCGGTATGGAATAATCATAACGTGTTTTCTGTAATATTGTGTCGATAAACGT

GTACAGCAATATCACTTCAATTAATAATTATTATTATAACAACTATAGGTACCAAACAAATATGATATGTGATTGTCGAT

TGATAAAATAAAATAGATTGATTATATTATTAATAGTATATTACTCAAAACTAAACATTAATCACGAATAAATTAATATA

AATTACGTAGCATAAATAATACGATGAAAATGATAACGTAATAATAGTATGGTGATAATAACTTATTACTATACCATCTC

ACGAAATATCATAACGACGTGGCGCGTTAGGTTAGGAGTGTTATAAATATTATAAAAAACTGAAAATCGTGCGTTTTCAA

ACGTTAAATTATACGAAAACGCCGCCTCGACGTTATAATACTATAACGATATCGATAGTAGGTATAATGTGACGACGATT

AATCAGCCGAGCCGTTCCAGGATCGTCGAGTACATCTTTTCGACGGAAGACAAGTATTTTTCGACGTCGCTTTTGTTCAA

CGTCACCGTATTTCCGTTGTTGGTCAGATCGTCGGACGTACCGTAGTTGACCGGCCCGTTGTTGACCGTGCCCACGTTTA

TGATACCTTTGTCCGCGCTGCTCCTTTCGATCCTGCCTTCGTTGATCGTCCCGTTGTTGATGTCGTCCTCCTCCATGTTA

CCGTTGTTGAACGTACCGTTCCCCACGAAACCTTTCCACACGTTCGCGTGGTTGTGCGAACCTGCGGTCACGACGTTCGA

GTCGGAATCCGTTTTGCCGCCGGCCCCGGTATTTTCGTTTTTCACGATCGCGCTATTGTTTTGCACGACGTCCGCACGAC

GGTTCTGGTCGTCTTTACCCAAGCGGTTGACGACTTCGGGACGTGGCCAACGAGTCTTTCGGCTGCCGTCTGCTTTTGGC

GTCCCCTCGTCGCCGGAACCGCTTCCGCCGGACGTCGAAACGCGCTCATGTGATCGGTCGGACGTACCGTTGAGGTCTCG

TGCGCGGTATTGTCCGCGAGCGCGTTCTTGTCGAGCACCGTTTTCGGGAGCTTTGACTTCGTTTCCGTTCGGGCCGTCGG

TAACGATGGCGGGCGTATCGGTACTTTCGGACAGGTAGTCTGGAGACCGATTTTTGGTATTTCCGTCACTGTCGGCGGTT

TTGGCTATCGGGGCGATGACGATTACCATATCGGGGTCGTCCGGGTGGTCGTCAGTACGACGAATTTTGGATTCAGGTGA

CGATTCGTAGATGGTGATTGTGTCGGAAGGCTCTTTTTCGATGGTCACACTGTTGACGGTACCGTCATCGACACTTAGGT

TTTTGATCGTGTTATAGTTTGCGGAAGTGTTGTTATTGACGACCGCCCCGCCAACGGCTTCGCCATCGACGTTTTTCGGC

AGGCTTTTATCGTCCACGATGGCGGACGCGTCGTAATCGATCTGACACAGTTCCTTGTTGTCCTCCACTATGTTGGTCAC

AGACTTGTACACTTTGGACGGATCATCGGATTGAGTGACCGCCTCGTACTGGTAAACGTCTTTGTGGTACTCTTTTGTGT

ACCGGGTTATTACCTGGGTGATCTTGGTGATGACCGTGCGTACCGACAATTTTCCGTCGGCCTGTGGTTCCCTCACCTGT

TTGAGCACCAAAAGCTGGTCGACGGCCACGCACTTAAGCCCGTCATGGCACGCTAACTTCAGTCGCGCCAGTTCTCCGTC

GGAAAGGTCGACCGGCGATTTTTCAATGAGCTCGTCGTCCAAACGCTGCCCCGTTTTTATCGTCAACGATCGTTTCTTGA

TGTCCGTCCAAATGCTCGAGCTCTGGATCGCCATCACATATTTTAGGTTTGACGATCGGCTGTCGACGTCCACGATCTGC

GCTGTTTCGTCCGAATGGCCTCGGATTTTTTCTTCTCTTACGAACGTCAGCTGTGTTGGTATCGGGCCGGACGAACGGCC

GAGCACCGCAGACACGGCCAATGCCGATGTCAACACGAACGCAAACAACATTTTCACCGTTGGCGTATCACAACTAAATA

TAGATATATATATATATATATTATATAATGTACATTTATAATATATATATATAT

>contig_10647

ATATAAACATGATCTACTTATTATACAAATATACACATATAACAAATATTATATTAAACCTATATAATCTTATAACAGCA

GTAAAGTCCATTATTTTTGTATTTGCCGATAGTAGCATAATAGAGTATTTGGTATTTTCTCTTCGTTATTTGTTACCTTA

TCGCGAATTGCCTGAAAATTTAAGAAGTTGAAGGAGTTCATATAGAAGTTTAAATTGTTCCTTCATTCTACAGTCAATTT

TACAGTTCATCGCAGAGTTCTATCAGGATGTTAAATTTGGTTGTATGGTTATGTGTCATATCAATATTGGGAACTTCCAA

AAGTTATATCACTGAAACTAATGACCTTATGGTTTATTATGGAGATGGTCCTCATTCAAATAGCATATATTATAACCAGT

GTGCACCTGAAGTGACCTGTGATCGAAATGCTATTTATAGGACAATAAATGGCGAATGTAATAACTTACGAAACCCGTTG

TGGGGGAGTTCGGATACTCCATACATCAGACTACTTGAAGCTGCTTATAATGACGGTGATCACGAAGTTCGAAGACAGAT

GGATGGATCAATGTTGCCCAGACCACGAAAAATTCAGTTGTTATTATTTTTGGAAAAATCCCGTGATTTTCTAGACTCCA

ACAATTATCATTTAAGTCAATTTGGTCAATGGCTCACTCACGATATGAGTCTCCTGTCACCTGATTTTGGTGGGCCAAAA

AAATGTTGTGATATTCCAATTGAAGAGATAAACAATAATAGTCCATACCAATGTCAATTAGTTATTGAGGTACCAACGAA

TGACCCAGTTCATGGTTGTAATGGACAAACATGTATGGAATTTAGACGTGCAATGACTGCTGCCCATAATTTTAGCTGTC

CCGTAATTCCTCAAACTCCAATGAATCAAGCGACATCGTTCATTGACTCATCTCAATTGTACGGGCACAAATTAGCTAAG

GCGAATTCGATTAGAACATTTGTCGGTGGGAGATTGATAACAGATATCATAGATGAAAATGAGTTTTGTCCTCTAAAGAA

AAGAAACGGTTCATTATTATGCAATGGTCGCGATAACGTGGGCATTTGTTTTGAAGCCGGAGACCCAAGAATAAATCAAC

ACTTTGGGATTACATCATACACTATTATGTTTACCCGGTTTCATAATATTGTGACTGAGATGTTAGAACAAATAAATCCT

CATTGGAGTGATGAAGTGTTGTATCAAGAAACTCGAAAGTTTGTTGGTGCACTAAACCAGATAATTGCTTATCAACGTTA

TTTGCCTATTTTACTTGGTAAATCATATGCAAAACGCTATGGTTTATATCTTAGTGAATTTAAAAAGACAAAATATAATC

CATTACTCATACCGCAATTGTCCGTGGAATTTGCAGGAGGTGCTTTCCGAGTGCCACATAATGTAGTGGCTTCGTTATAC

AATTATATAGGCAAAAATTATGAGATATTAGATTCAGCAAGACTTAACGAATTTATGTCATTTTCTGATCCCTTAGTAAA

GTGCTCAAATCTTGATAATATAGTCAGAGGAATGACGGTAACACCAGGTCGATATTTTGCTCCATCGTATAACTATCTAA

TATCAAATTTCATGTTCCATGGTCATAACACTGGCGATCAAGATTTGCTCGCAGTGGACATTCAAAGAGGGCGTGATGTG

GGCGTACCGCCTTATATTAAGATAAGAGAATGGTGTGGTTTACCGCCAATCGATTCGTTTGAAGATTTATTAAATTTCTT

ACCGTTTGAGGACGTCGAAACACTGAGAGAACTGTACGCGACTGTCCACGACATTGATCTACTCGTGGGAGCTTTACTTG

AACCACCAGTCGATGGTGGAACGGTCGGTCCAACTGCACAGTGCATCTTAGCTGATGTATTTTATCGTACTCGCTTTGGT

GATCGTTTCTTTTGTGATGTAGAAGATCAACCGGGAAGTTTTTCACAAGAACAATTGGACACTTTGAGAAACCTCGACCT

GGGTCATGTAATATGTGCTACAACTGAAATGGATGAAGTGCCATTTGATATTTTTGATCCAACAATGTATTCACAAATGG

TGAAATGCCAAGATATTTTAGTGAAGTTGGATCTTAGTGCTTGGAAAGAATTTCCATAGTTCGACGAATACATCAATTAT

AAATTAACCATAATAATAGTACATGCGAGAAATAAATTGTATACGAATCAGCTGTAATTTTTAATTTAAAATTATAAATA

AATGCAATAAATACTTAAAAAAGG

>contig_10797

GTTATTGTTTACCTTGATGGTTCTTCCATTCTTTCAAACATGCATTTTTTCTTCTGGCGTCGGTGTAAATTTTAAAGGAA

TGGAAATAGCAATACAAAATGATGAAATTAGTTTAAATGATTGCAAACACATTTCTAATTTCAATGGATGTATTTTTAAT

AATGATACAACTCAATTAATGAGTTGTATTGCTGTACAATATCTTACATCACTCGATTATACACTGGTTGAAGTGAAAAA

TCGTCATGCTGGAGAAATGTTTGTGGACAATTACAAGTTTTTAGCGTTTATACATTTTCCAAAGAGCTTTACGCAAGAAT

TAACCAAATTTATTGACACTCCTAATGAATATGATACACAAGCGCTGTCTTATATGCACTTTGCCAAACATAATTTATTG

TTTAAAAATCAAATAATTTTGGATGTAACAAATGCAATGCGCTCTCTGATATACAACACTTTGAATAGTTGCTCCAATAA

TCCAAAAGTAGCTGGTTTACCTATGGAATTCAATGCATTATATGGAAAAGAAGTGAAAGTTCACGTCCACAGTACAGCAG

CAGTTTTTCTAGCAATGGGAGCATTTTATTTTAGTAGTATTTATTCAGTGAGCACTATGCTTTCAGAAAGAATGGATGGA

ATCTTAAGTCGATCGATGTTTGCAGGCGTTACAATATTGGAATTGCTTATTTCAATGTTTTGTATTTCGAATTTTTTAAT

TTTGATTCATTCGGGTATAGCTGTTATTATCGCATACGTATTTTTTTCGAATCCTATTCTCATATCCAGTGGCCTATTAA

TGTATGCTATGTTGATAATAATTATGTCATGGATAGGATTTCTATTTGGTTTACTTGCCGCTGGAATAAC

>contig_10798

AGTAAATAATGAATACCTTGAGAACAAATATGACAAATATTTGCTGAAGTCTAAAGCTCTTATTTGTGTATAACCCACAT

ATTCTTTTTTATTTTACTTAGAACAATTAAAACCAATATTAATAATATGGCATGTCCAAATGTCGCCAAGGTACCCTTTA

TGACCGATGGATGACCCAACGTCCAACCTTTCAATGTTATGTCGTTCATTGTCTTTCCGGCTAACCGCATAGGAAGTAAC

TCAGATACTGATCTTAATAAGGGTAACTGTCCATCAATTGGCCATACTCCACC

>contig_10955

CTCAGAATACACAGCATTGAAACAATAATACAATAGACAGAATTATACTATACGCGAATATTTAGTCAACTTTTTAAGCG

AATAAAACAATATTTATGTATTTTTTACTAATAATATAACAATTTATAAAACAATAAAAATAATAGAAAAGATAATCCAA

TTAACAATGACATAATAAATTAATAGTTAAAAAATTTATATTTTATAGCTTATTACTAACTTATATGAACACAATTATAA

TTATAATAAGGCTTATTTGTATCAATGCTAAATTTTTACAAGATCAAGGACGGATATACACCATTGTTGCTCCAAGTTAA

ATTTGTTTGGGTTCATACATCAATATTCTATAATTTATAATTAAATGAGATATTAATACAATAGGTCAGTTTGGGAATCA

AATTGGTACGCAAAAGGTCACATGATACTACGTGAAAAACCACATAAAAGTATGAAAAATAATTCAAATTTTTAGTTTTA

TTTATTAATATTTGTAATAACACGAATAGTATAATTATAATATATATTTCTATTACATATCATATTTTATAATCATGTTT

CCCATATTTTTTGATATTAATAGGTATAAAATGTTGAATAGTTCTATTTATGTAACTGATGTGTAATATCCATAGATGGT

TAACAGAAAAAACTTTGGAAGCCTCTGCAATAGATATCTAGAGAATAATCATGTTTTAGATAAACCTGTTGTTAAATTGA

TATAAATGAGAAATATATAAAATACAATTTTAATACAAAATAGCCGGCCCTCTAAAAATGTAAATCCTAGATTGATTATT

TTGCGACACTGGATCTGTTACTGTACTAGAGTTTATTCTTAATAATTAGTTAAGTCAATTTTATATATACACATATTTAT

TTACTAATACTTAGTTTGAACACCATTACTGTGTTAAAAATGTATTGTTATGGGGTATAATAAACATATACAATGGAACT

AAATACACTCACTCAACGTATTGAGTATCTATTTGAATGTACTACCTCATCATTAAATAGAAATCAAGTTCAGTGTGTAC

TAAATGTGTTAAGTTAAAGGAAATTAAATATATTTAACTTCATCAAATGGATAATAAATATAGTAGAAAATGTCCTAATG

TATCCCATTATAATATATTAGGTTTTATTTTTATATGCAAGTTTATGCCTAACTCAGATTCAAAGTTGTTTTTCATTTTC

AAAATAATAAATGTTAAAAAGTTTTAGTGATAAATTTGTGAAATTTGGTATTTAAAATTATAAAATAAAAATTTGTACTA

TCTGAGCAATATTAACATCTTAATAAAATTCATACCTATAAAATGTAATTTTAAACTCAACCTAAATCATTTTACTTCCC

TTTTTTTAGATTTTTAGTGTTTTTTACACTATATTCTGAACATAAGCACCAAGGAGTTAACGTCTTATTACATTTTAATT

CTATATCTAACACTAGTGTAGGTACACCATCTATGAGCAATTCATGTTTTTTTTTTACAAAATAGTTAATATTATGTAAT

CCAGTGTGTCTGTCACGCTTTCTTGTTACTTCACGTTGGTTATAACATTTTGTCATATCACGTTTTCTAACAGTTCGATC

ATGATTGTGTCTGAATGTATTTGATATACCCGTTGTTAAATTTCCTGGTCTATGAATACCTATCTTGGCTTCTTTAAGGC

GTAAATAAAACTCATCATCTTCAAGACCCCAACCCCAATAATTGTTGCTTAAACCATCGATTTGTATAAAATCTTCTTTG

GTAATAAGTAGAATGCCACCCACAAATGAAGCATAATGGTAACGTGGATGCAAATGAGGAGCAGCAATGTGGTTAACATG

ACCAGTTGGAGGATAATGATAATCTAGTGCAGGGTTAAGAGGTAATAAGTCAACATCATGCATAGCTATATAATCAACCA

TGCTATTTGAGGACATTATTTCTTTAAACCCGGCATTAATCAATGAACCTCTGTTAAAACGGTACTGATCAACTTGGTTG

ATAACAAATATTTCATGGAGTACATCTTGGTTTTTCAGAAACGAATTAATATGTGGCACAAATTCTAAAAGCTCATCAAA

TCTTTCTTTAAAAGGTATTATGATGCCAAGTTTATTACCTTTGAACACAGAAGAAGCCTGTTCTTTTAACCGTAAATTTC

CACCATTCTCAAGTTCTCGTACATATAACAGTGACAATAATAGAGTGATTACTAGAGTGAGTATGATTCCAATTAATAAA

ATTTTTGCTTTAAACATCATGATTGTTAAATGTGTGTTGATTTTCTAAAGATTATATTTTAATGGTCATAATTTAATTCA

GAGGAAAACATTGAAATTGATGAACCACTAACAAAATCTTAACATCCTTAAGAGATTGAAAGATATTTTACCTATTTGAT

TGCAATTACTGTTTTGTATGTATATTATAGTATTTATTTAAATCAGTCCAATTTCAATTCTACTGAAATTAAATTTAATC

CGATCGAAACGTTCGGAAATAAATCCCTCGGTCATTGTAAATTAAAAATGTTTTTTTTTTTAACATACTTAGAGAACAAA

TTTCTTTATTTTATTTAGAGAGAATTTATCCATGACAAAAAACATTTAAATACTGGCATTCGGCAGTCGGCACCAGTAAG

TAGTAGCTTTTAATCATTATCAATTTATAATATCTACCGAAG

>contig_11008

GGTACTTCAAAGCAGCCGCAGTTATTTTAGTTTTTTGTACGGTCGCCTCGTGGTTCACTACCGGATACATAGCATACACT

AACAAGCACATGCCAAATATTGACGACCCGTTGGCGCTATTCGACAAAATCTACGACAAACCGTGGACGAGGCTCGGACC

GTATTTCGTGGGCATGTCTATCGGATGGTTGCTGTTCAAAACCGAATGCAAAATCAAGATGAACACGCTAGTCGTGATTA

CCGGATGGACCGTTTCGTCGGCGGTGCTTTGCAGTCTGATATTCGGTCTTCATCAAATGGATTTACATCCGGTCGCGGCA

GCCGCGTACTCTTCGTTGAGTCACACCCTGTGGGCCCTGTGCTTATCGTGGACTGTGATCGCTTGCGCTACCGGACACGG

AGGTTACATAAATAAATTGTTGTCCTGTAAAATCTTAATACCGTTCAGTCGGACAACCTATTGCGCCTACCTAGTTCATC

CGATCATCATCAGGTATGTGGTAATGAAGAGAGACACTCCACTTCATCTGACCGTGGAGACTGTGGCAATTTTGTTTTTG

GGACAAA

>contig_11144

TATTTAACAGTACAAATACCCAAAAATTCACGCACTAGTCGTCAGACGATTGCCCAAACACTATGATGTACCTTTGTTAC

ATTTTCGTTTTAATAACAATAGCATTAAGTAAAAATGCTGAACCTGCAAATTTGGAATGGTGGCAGACCAGTGTCATTTA

CCAAGTGTACCCAAGATCGTTTAAAGACAGCAATGGTGATGGAGTTGGAGACCTGAAAGGTATAGAAGAAATGGCTGAAC

ATTTCTATGAAACAGGTATTGGAGCTATATGGTTGTCCCCAATATTCAAATCTCCCCTGGCTGATTTCGGTTACGACATT

TCCGATTTTGTAAGCATCGACAGTACTTATGGAACAATGGAAGACTTTTTATCACTTCAGATGAAACTTAAATCGTTTGG

CGTTCGTATTCTATTGGATTTTGTGCCCAACCATTCAAGTGACGAACATGAGTGGTTTCAAAAATCAGTCAAAAGAATCG

ATCCATACACCGAATACTACGTGTGGTTAGACGGAAAATTAGACGAAAATGGTAACAGAATACCTCCAAATAATTGGCGA

AACTATTTTGACGGAAGTGCTTGGGAGTGGTCGCCTGAAAGAGGACAATATTATTTGCATCAGTTTACGGTTAAACAGCC

AGATTTAAATTACAACTCTCCTGCTGTATTAGAAGAAATGAAGAACGTACTTAGATTTTGGCTGGATATCGGCATCGATG

GATTCCGTATAGACGCTTTGCCATTCATAGTAGAAGACATCACATTTAAAGACGAACCAATGATTAATCCAAATGTCAAG

GATGATAATTACACATATTTCTTGCTAGATCATACGTTATCTAGGGATCAACTAGGTACATATAAAGTTGTCGAAGAATT

TAGAGCAGTCTTAGACGAATATTCTAACCGTGACGGAAACACAAGACTGATGTTAGTGGAAGCTTACGCGTCCATAAATT

ACACGATGATGTACTACAGCCAGCAGACACCCAGGGCTCATATGCCGTTCAATTTCAACTTTATCACATACTTGAATAAA

ACTTCGTCGGCCGTTGATATAAAGAATACGATCAATCTGTGGTTGGATAACATGCCTCCTGGTCAATGGGCCAACTGGGT

GATAGGGAACCATGATAACAAACGAGTGGCTTCAAGATTCGGTCAGGACATGGTCGACCCGATGAACACGCTGGCGACGA

TGTTACCGGGCACGGCGATCACTTACAACGGAGAGGAAATCGGTATGGCTGACGGTACCATAAGGTGGGATCAGACGGTT

GACCCGTTCGGTAAGAACGGCGGTAAGGACAAGTACGAGCTGAACTCGAGAGATCCGTTTAGGACACCGTTCCATTGGAA

CGATTGGCAGAACGCCGGGTTTTCGACGTCTCAACGCACATGGCTTCCCGTGAACAGCAACCATTGGTATCTCAATTTAG

CCTTTCAAAAGGTGTGCTTAAGAAGCCACTATCAGACGTACAAAACGCTGTTGAAACTCCGTTCGTATCCGACGATAGCA

CAAGGAAATCTCACCATGTACACGCCATCAGATTGGATTTTGATTTTAACTAGACAATTGGAAGGCCACGAGACCTTCTA

CATTGTTTTGAACGTAGGGACGGAACAAGAAGCAATTATTTTAAATGAACACTTTAAGGACATACCGCTTCATATGATTG

TCCGTGCTTCGAGCATAAATGCATGGCACTCAGAAAATGATTACATTGATACAACAAAACCATTACCAATGCGACCCAAA

TCATCACTAGTGTTATCATATTAATAAAACGTTAGACCCAAAGTATATATTGTATACACTTACATATTGTATTATGATGT

AATCAAATTACGAATGTTGCTTATGATCCCACTTAAAGATAAAATATTATTACCTCTTAATAGAGTCGTTTCCATATTTT

AATTATTATTATAAATATTTTTGTATTTTTGAACTTTAAATTAAATATAAATTTAAAAAAA

>contig_1144

AGTAGGTATTAAATATATCATTATCTACTCTAACATAATACAAATTTTTAAAATAAATAACATATTTATATATTTTATTT

CATTTCATTATATAATTATTTAATATAGGTACTTAATATTATATCAAACATAACTATAAAATAGAAGGCCTTTATCTAAA

GCAATTTAAAACCGTAAAACTCCTAAATACCTTTAAAAAATCATAAAATTATGATGTCAAGTTGAGCAGCCAGTCGTGGC

CTTAAGAGTGAGTGGGTAAAGATAGACAATGATAATATTTAATGATGATAATCATCGTGGCACTTGTGTAGGTAATGGGA

ATTCAGTGGGGTCTTATATGATTTTATCGGTTCTATTAAACTTTTTTTATTTGGTGAAATGGTTCATCGATTTTGCGTCA

TAGAGTAACTCTTCAGAAACTAATTATTAACCCATAGATTCTACCGACTTGAAGTATTCATTTTAAGTAAGTAACCCAAG

TTGAACTTTTGATTTTAATATCGAAGAAGTTTCCATCTAGTCCATCCAGGAAATGTAGAGAAAATAACTTCACGAACACA

AATTATAAATTGTTATTTTATATAATATAATATTAATCATTACATTCCAATTACCTATTTCAATATTTATATAATAGGTT

AGGTTGGTATCAGGGATGCAACACGTTTGAGCACACATATTTTAGGCTTATATAGTAAAGGTACAACTTGTTTACGCACA

TTTTTCCTAAAATATAATCGGAAAATATAGAAATAGTACTATACTTAGTATTTGATGCTATCGACATAAGATCAGAAAAT

CCATCAGAAACTTTGTCAGGGAGTAAATAACTTAATCCAAAAAAACATTTCATCCATTCGCCAATTTCAGAGTCGTTGTT

TAAGTACTCGTTTGGTAATATTTTGTGTTATTGAATATGCCAAAACCAATTTTTCCCTAAATGAAAGTTACAATACTCAA

TTTTGCAGTTGGGAAAATTTGCAGTACTGCAGATTTTTCAAAGTCAACATGAAAATTAGTTAAATTTATAAATTTATTAT

ATTTCTAGTAATTTGAATACATATATTTTGTAATGTTGTCTACATAGTGATGTAAGTATCAGTATGTTTGTTACTTAAAA

AACAAAACGCCACAGGTTACTTTAGGTAAGTTCAAATTTTAAAAATATAAAATATACCTTACTCAGTGTTTGTGTTATGC

GCAAACGAGTTGCACCGGGTTTCAGTAATCTTAGAAAACTGTCATCTTTGTAAGATTCACAGTCTAAACTGCATAATGAG

GACACTTTATACGACGAGGTAAAAATGTAAATTTCGGAACTCAGACACTTTGCAATACTATTATATAATATAAAAATAAA

GTATGTACCCATGTATAGTAAATATAAGTTATATACCTAAGTAGTAAGTATATATTTTGTCAATTATTAAAATGTGAAAA

TAAAATTCAATTACTAATGCTATAAAATATAGTATGTTAACAAGTTAAAATAATCAAAAACTCCTAAAAATAAAAAAAAA

ACTATTTACAAAATCTGAAATTATTTCAGATGTACAGCGTTCTAATTTAAAATATTAGATTTTTAAAGTGTGCAATATTA

AATACTATAAGTAAACGTACAACAGGTAGTGTAACAAATACCAGTGTATGTATATTTTCATTTTACACAAGTATGTAATA

TTTTATAGTTTCTTAAGGAAAAATTAGTATGATTAATTGATTGTGTTACACAAACATATCTAAATCAAATATTTAATATG

CTTTAGTTTATTTTTATAAATAATTATTATATTGCAATAAATAACCTATAGGTTTCATTCATACATTTATTATGAGACAA

TATTTATTCCAATAAACTTAATTATTAGGTAGATGTTTATGAAAATCTTGAACACTTTTGAATTTTTGTTCCGTACCAAA

TCGATTTGAAAAAAAAACCATTATTGTTGTACGTTTGGTATTCAATTAATAAATCATGTACACAAATATAGAATATATTA

AAAAAAAAACTCTGCTTAAGTTTCAAACCAATTTTAATTTAATTTGTACTTAAACTTAAATACTTGCTTCAATAGTTTAA

TGAGTGATTATTAATGATTTTCAGAAAACGTAATGACATCTTCATTTGGTTTTGCTGTTGAGAGGACCATAGATGCTTTT

GGTCTCAAAATTATTTCTTCCGCGTTAACTAAATTTCCAGTTACATAACCTGAGTTTATACTGGAAACTTTGACTTTTAA

TATTTCTGGTAATGTTGGTCTAGCTGTTCTTATATTGATTCGCTCTATTTCACTGCCGAAATTAATGACGACGAAGAAAG

TTGGATGATCGTAAAAACTCCTCGAAAAGCCTAACACCCATTGAGACAGTACGAATACGTGCAAGTCTCCTTTGAGGATT

GAGGGTATCTGTCTCAAGCGCGCCAACTGTCTGTACGTCCTAAGATGACTCTTAAACTTGGACAACTGAACCAAGTTTTC

ACGCCAATATTCCGGGTTGATGGGAAGCCACGTTTGCGTTCCGTTGGTAAAACCGGCATTTTCAGAGTCGTCCCAGGGAA

ACGGGCTTCTGGCGGGATCCCGACTGAATTTCGTGTACCGCAACACGCCGACGTTAAGTCCGGCCGGGTCCAAGGTCTGG

TTCCATCGGACATACGTGTCTTGTACACCCAACTCGTCGGCGTAGTATGTGATCGCAGTTCCCGGCAGTAACATTTGCAA

CATGTGCATGCCATCCAACAACAACGGGTTCATCCTAGACGCTACTCGCGAATTGTCATGATTGCCGATCACCCAGTTGG

GCCACATTTTTTCAGGCATGTTCAGCATCCAGGTTTTAATCATATCTCTGACATCGTAGGCATCGGATTGCTGATTGATT

GAATTGATCAACAAAAAGTTGAATGGGAAATGAGCTCCGGGAGATGTTTTGTTGCCGTAATATAGCATAGTGTTGTTTAT

TGGTGAGTAACATTCCACCATAAAGAAGTTGGTTTTTTTTTCGGATGATGAATAATCGTCTAACAATTCTCTCCAGACGC

GTACCATTTCATAAGTTTCTGGTTGATCAAGCGTGTAGTTGTGCACTAGCGAGTCGTAATCAGTGTCTAATATGCCGACT

TTATAAGATTTACCTTCATCGGCTAAATCTTCCCTTTCGTATAGATAATTGACAGCGTCAAATCTGAATCCGTCTATACC

ACGTCCCAGCCAAAATAATATGATATTCTTGATTTCTTCTACGACCATTGGACATCTGTAGTTCAAATCTGGTTGTTTTT

TTTGGAAAGCGTGGAAATAGTATTGTTGACGTTTTTCGTTCCACTCCCACGCAGACCCGGTATTGAAAACTCCCAACCAG

TTATTCGGAGGTGATCTAGTACCATTTTCATTGATAATTGGGTCTTTCCACACGTAGTAATTTGTGTAAGGTTCTTTTCG

CTCTACAGATTTCAGGAACCACGGATTTTCGTCACTTGTGTGGTTCGGCACAAAGTCCAAAATTACTTTTATACCACGTT

CGTGAAATGCATCCCTCAACCGATCAAAATCCGCCATAGTACCGAATAATGGATCAACCATCTTAAAGTTCGAAACGTCA

TACCCAAAATCGTCTTGCGGAGACTGAAATATTGGAGATAACCAAACCGCTCCAACATTTATACTTTTAAAATAATCCAC

TTTTTCCGTAATACCATTGAAATCTCCGACTCCATCACCGTTACTATCTTTAAAAGAACGAACGTAAATCTGGTAAATGA

TTTCCGATTGCCACCAGTCTGGTTTGAATCTATCATCAAATCCTTTGAATATGTACTCAGAGGACACAAATGTATTGGCG

ACAAATAACAAAACAAAAATAATTATTTTCAACATTTTGATGTTAAATTAATTTAACGGTATGTTGACGACGTGCGTTTA

GCCGCCAGTGTAATAGCTAATTCAAATAGGGTACGTGTATTTATATGAATATATAAAGTAAGTTCAGGCTTAAAATTATT

AATATAGATGATAA

>contig_11484

TTTTTTTCTTCTCCGTCGAGGCGATCGTAATAATATCGTTGTCGGTGGGCAGGGCACCGGTGCAGCACCAGCGACAGCCG

ATAAACACCGCGACCGGGCAGTGCAAGCGTGCATGCGCGTCGCGACCGTCCACACGGTTTTCCCGTCCTTTTGGCCCTCG

TTTTTCTCGACCGATCGTCGTTTTTCCCGCCGCGCCGCCGTCTTCGGGTTCGTAAATAATAACGATAATAGTTATTATTA

CTGTTGTTCTATCGCCCTTCGTAGCCGCCATGTCGTCGTCGCCGCCGCACTGTCACCGCCGCCGTACGCCGCCCGTGCAG

ACGGCCGTCGCGGCGTTGGTAGCCGCCGCCCTGGCCATCGCGGTCCGCTCGACGGTCGCCGCCCCGCAGTCCGACGGCGA

CGCCGTGATCACGATCGCCGGCAATCAGTGCAAGTGCGTGCCGTTCTATTTGTGCGAGTCGAGCAATACCGTCTACGTAC

CCTCTGGATCTACAGAATTAGGTTGTATA

>contig_11634

GGTTCGATATTCGAAGTGAAGTCGACGGCGGGCGACACGCACTTGGGCGGCGAAGACTTCGACAACCGGCTGGTGTCTCA

TTTGGCCGAGGAGTTCAAGCGGAAATTCAAGAAGAACGTGCACGCCAACCCGAGATCGTTGAGAAGGCTTAGGACGGCCG

CCGAACGGGCCAAGAGAACGCTGTCGTCCAGCTCCGAGGCGGTCATCGAGATCGACGCCCTGATGGACGGCATCGATTTC

TACACCCGGGTGTCCCGGGCGCGGTTCGAGGAGCTGTGCGCCGATCTGTTCAGGTCGACCCTGATCCCGGTGGAGAAGGC

GTTGGCCGACGCCAAGATGGACAAGGGAGACATACACGACGTGGTACTGGTGGGAGGTTCGACGAGGATCCCGAAGATTC

AGAGTCTCCTGCAAAACTATTTCTGCGGCAAACCGCTCAACCTGTCCATCAACCCCGACGAGGCGGTGGCGTACGGCGCG

GCGGTACAGGCGGCCATCCTCAGCGGCGACACGAGCTCCGCGATCCAAGACGTGTTGCTCGTGGACGTCACCCCCCTATC

GCTGGGCATCGAGACCGCGGGCGGCGTGATGACCAAAATCGTCGACCGCAATTCCACCGTCCCGTGCAAACAGACCCAGA

CGTTCACCACGTACGCGGACAACCAACCGGCCGTCACCATCCAGGTGTTCGAAGGGGAGAGGGCCATGACCAAGGACAAC

AATCTGTTGGGAACGTTCGACCTGACCGGCATACCTCCGGCGCCCAGAGGCGTGCCCAAAATCGAAGTGACTTTCGATTT

GGACGCCAACGGGATTTTGAACGTGTCGGCCAAGGACAACAGCTCCGGACGTTCCAAGAACATCGTCATCAAAAACGACA

AGGGTCGCCTGTCTCAGGCCGAAATCGATCGCATGCTCAGCGAGGCCGAACGGTACAGAGACGAGGACGAACGTCAAAAG

GCCAAGATAGCGGCCAAGAACCAGCTGGAGAGCTACGTGTTCGGTGTCAAACAAGCCTTGGACGAGTCCGGCGACAAGTT

GACCGAAACCGAGCGGAACGCGGGAAAACAGGAATGCGATGCCGTCATCCAGTGGTTGGATAACAATCAGTTGGCAGACA

AAGAGGAGTACGAGCACAAACTTAAGGAGATCCAAAAGAGCTGTTCGGCTCTAATGATGAAAATACACGGTGGTGCTGGA

CAAGGCGGCGGTGACGCGCCTCCGGCCGGCGCTCATGGTTTCCCCGGTTCTCGTTCCGGAGCCGGAGGACCTACGATCGA

AGAGGTCGATTAAATTGCTTCCATAATTTCATTTTATTGTATATATTTTGTAAATATGGAAATACGTTGTGAACAGCATA

CTAATTTTATTTGTTATGTTAAGTATCCACTAGACTGATTTTTCTATTGTAATATTTAATTATTTTGCAAAATAAATATT

TTATACTTGATAACTGTAAAAA

>contig_11662

TTTTATTATATTCTGTGATAATGATTATTATTATTATAGTTCTGATAAGAAAATACATATTGTCATATTATTATTTATTG

AAATTTGGATAGGTTATAAACTTATAACTTGTAACTTTAAACTTATAGGTATTCATCAGTCAACACCAATAGTCGATATA

CTATTAAGGTTCAGGAAATCTTGCTGTTTTAGAACCCCTCGGGTATTGACAATTAAAAAATCGATTTTTTAAATAATAAC

ACTTTTAGTGTTCGATATAACTTATTAGTAAATTAATCGACAATGTTTAGTATTGTATAACTATACATTGATAAGTAAAA

ATCAAGTCAAATCAACAAAATGATTTTGTTAATAAAGTCATTAGTTTTTATAATGTTTATAGCTAAATCATTGAGTACTA

CGTCAGAGGATGGTGTTAAATATGCCAACAAATGTGAAGTTTGTAAAATATTGGCAGAAGAATTAGAGGGTCGTTTACAA

GAGACTGGTAAGACTAGTGAATATTTAGAGCTTGGTTACTCAATAGATCAACCAAAAAAGAAAAAAGAGTACAAAAAATC

GGAATTGCGTTTAGTTGAATCACTAGATGGCCTTTGTGAAAGAATACTGAAGTATAATATTCACAAAGAAAGAACTGATA

GTACACGATTTGCTAAAGGAATGAGTCAAACTTTTCAAACATTACATGGCCTTGTGAATAAAGGTGTAAAAGTTGAACTT

GGAATACCGTATGAACTTTGGGACAAACCTTCAGTTGAAATTACAAATATGAAAACTCAGTGTGAAGATTTGTTGGAAAC

CTATGAATCAGATATAGAGCAATGGTATTTTAATGATCAAGTTGAAAGCTTAGGAAATTATTTATGCAAAGACCGCGCAT

TGAAGGATTCAGATAAAAAATGCTTAGATGAGGCTCCACCTAAAGGAGATACAAGACAGATAAATCAAGAATTGTAAAGG

TTAGATTAGTATAGTTAGGTATATTTTGTTAATATTTCATAAACATTATCATTGAAAATATGTATAGGATTTTAAATATA

ATAAATAATTGTGTAATCTTATGACATCTAAAATTATTTATTTATAATAATGAATCAAGTAAATACGGAAATCGGTTAAT

TTTTATCTTATTATATTCTTTATGTTATTGAATATAAAACAATAAATTTAATATAGTATTTTATATCTTATGAATATAAT

AGTAAACATGTCCAAGTTTTATTGTAAATAATTGTTACATATTTACTTCATTCGTTAAAATTAAATATATTTTGATAACT

TATCCATAATATCATTGATCTACTTTATAATCAATATAATAATAAATGATAGTAATCAACTAATTATTTTAAGTAAATAT

TTTTTTGTAATTTGTGTGCCTTAAAAATGAAATATTTGTTTTATATTTAATTATGATTAAATTAACACATTTTATAACTC

AACTATAAGTAATTTTTGTAAATATCAAATTTGCTTATTGTTTTAATTATATACAGATTGTGAATTTTCTTTAAAGAAAA

AAATAAC

>contig_11810

TTTTAATTATCTAAGTACACAATTTATTGCAACTGTTCAAACCACGCACAATTTAGTTATTAGAAGTTCGAGATCATGAT

TATATTATAGTATATTATAATATGGATACATTTTTGTTTCGTATAAAGCGAATTACCAAATAATTTCCGTTAAAAAAAAA

TCAAACAATGTTGAGTCAGTTTTCGTTATTTTCGGTGATTTTAATTTTATTGGGAGGACGATGTTTTTATGGCATTAACG

GAGAGGAGCAGCAACAACAACAGCAGCAACCCGAGCAATCAAATACAGGAGAAATTACAGATATCGCTGGCAGATGGTAT

GCAGTTATGGCAACTCCATGCTCCGACGGCGGAGAGTGTAATCTTCTCACCAATAACAAAGTGCCATGCCAATGTATATC

AGCAGACTTTCTGGCTCTCCAGAGCGCTGGCTTTCAAGTGTACATGTATGCAATAAACAACCAGACTTCTAAAATAACAG

TCGATATGGGAGGTGCATCGTACGTGCCACCCGATAATAAAAAAGAAGTCCAAGGTTTCATTTACAGCCGTGCTACAATG

GATTTGAGCGTAATACAAAAAACACCATATGGTTTATCTGAAAATGTTTACGGAAGTGAATTTTTTTATAAGTTTAAAAT

GACCATTATCGGCTCTAATCTCGAATTTAAGTATATGATCACCACAATGTCCTCAGATAAAGTTTCTGAAGCAACGGTTG

TATGGTGCAGAGTTAAAGCACCAGGTAAAGCTACTATCTGGACCCAAATAGTACAACACTTTAAAAGACTAAATATGGAT

ATCCACAAATTAAATTTCATTAATCAGCTAAATTGCGTGTATCCAACGATACCTCAATGCAACGAAATATTTTTCTGTTA

ATAATCAAAAATGCTATTCGATATTGTACGTTAATTGTATAGGTAGTAATAAAATACATAGTATAATAAATGTATTATTC

TATTCAACAATTAAAATTATTAAAAATATTTATATCAAATATTTTTTTAAATTATAATAATACTTTAACGATGTAATATT

TTTTGAAATGTTTAAAAAAGTTATAAGGAATATATACACACACACATATGTATATATATATATATATAATATATGCTATG

TATATAAACACACACACATATACATTATATACGTATATATATATAATATATATTTTTTTTTTTGAGTAGGTAATAAACAT

TATAATGAAATAAAATGTTCGATTAATTCATAATAATTTAATGATAACGATTGTACTAAATTGAAAACATAACCGGGTGG

TTTTTTTTTAAATTTAAAAACTTATTCTAAATTTCTAAGGTACCTACATGAGCACGACATTTTTCGGTATTTTAATAATA

ATGATTATAAGATAACCTAATAGTACTTAGAAATATATTTATCATTGTTGCATATAATTGTGATTTATTTTCAAACATAT

TTTACGATTTATGTTAGGTACTTGTGTGGACCACAAGATCACATAGTTTACGGTTGTCAATTTTAATTTTTCCAAAAGAA

CTAAAACTATTTTAATGATACTAAAATAGGTTGTTTATCGTTAATATTAAATATTATTTTCAACAATACTTTCAATGATT

TCATTAGGGAATTTCGAATATGAACAAATACAAGATTAAATTTTTGGGGAACATATTATTTTTAATGTAAATTTTCAAAT

>contig_11924

CTGCCCGAGTTGAATATAGCTACGATCCACGGCCATTCTCCGGGATCCGCGTTTTGACCGCCGACGATCCTCTCTTGATC

TTGTGGACCGTTTTTCAGACCGCACTGATAATCGAAGCTGCCGCTATCGCCGTCCACATAAGACGGTGTGGTGGTAGGTT

TATTTTGCGGGGTAGTATTAGTAGTGAAGTTTTGATGTTGGAACGGCGGCCATGGCGGAATAGTCGGATGCTGCGCAGGC

GTTGGCTTGGTCGGATGCGTCGGCTCGGTCGGATGCGTCGGCTCGGTCGGATGTGTTGGCTTAGTCGGATGCGTTGGCTT

GGTCGG

>contig_1199

TTTGGATCCGTGTTGAGCTCTCTGCTTGCGCTGGAACTCGTTATTTATGTAGAAATCTTTTGGTGACGGGGCAAGTACAA

CAGGGCGCTCCTCCCAAACTTCTTGAATATCATCTTCGTCGTCTTCTTCTGGTATTTCATTTTCTTCGAACGGAATTGTT

TCATGTCCATAGTTTTGTCCATAGTTTTGTCCATAATTTTGGCCATAATTTTGACCATAACTTTGTGCGGGACGTCTGGG

TTTGTCTCGAGCCTTATTTGCTTCCAGGAGGGACATCAACGCCAGCAATTCTTCGGGATCGAAAGGCAAGTCACGTTTGG

CACGTCCGCTGTCCAAATAAGAACCATATACTCTAGAACCACTCCTCTTAACCGGGGTGCCGAAATGGTAAGCGTTCCTT

TTATAACGGTCATCAGCGTCTAGTGATTGAAGTCCGTAGGAATCTGGGTTGTAAAAGAGATCGGCGATCTTTTCGTGTCT

GGCCGCCTGGGGATAGTGTTCAAGGGCGTTGTTCACTTCAGGTACCGGTTCTGGGTCGTGGTGATCAGGTGCCTGTCTCT

TTTCAGCTCTAGCATAGTTTCCGATGTCTGCCTGTCTGTTTTGCTGGTTACCGAACTGGAGTACTTCCTGAGTCTTCTTG

GCTTTATTCTTAGCCATGTTTTTTTGGATCTTAGCCGCGTCCGCTTTACCGGGGGTGGGTGAGCCGTACGCCATATGCGT

GACGGCCACCGCCACCGTGATCGACAACACGAACGAAGTTAACAACTTCATGACGTGACGTAAGTTCGACCGTGTTTATT

TGCAGTGCAGTAACGCAGTGTGCTTACTTTTCGGTGTGTAGACAGTCGCGTTCTGTCCGTGGTTTCGGCGGCGCGATGAC

AACACACACACACAGACTACTACTGGCGGCGTCGGTGGTGGCGGCGCGTTCCTAGACGGTGGTGGCGACGATATCCGCGG

CTTCCACGGACATTGCGGACGACGAAGAGGAGCAGTTTTTTTTCAACTGTCGAGGACATGGGGAGTAGGTGTTTTCCGCC

CGATCGATATTTCCGAGTGTGCG

>contig_11991

CAGAGAATTATTACGACACAGCGATGTTAGATTCAACGACAATGGTACAATGACTTATGTGGCCACGAGAACGGCCGTGT

TCTTGCCAGAAATGACAAATATCAGCTTGGACGCTAATTTAATACTACCCAACTTTGCCGCGTTGGGAATGGCATCATAT

TTATGGGATGCTTCTTATTTTACTAAGTATGGTTTCAAATTAATTATGGACATGATGGATACCAAAATGTTTATCAAGAC

ATCTATTAATGATTGTTTATGGAATTTAACTGATCCATTAGTCCAAAAAGCAAAAACCATGATGCCTGGTTTAGTTCCTG

AAGAGAATATGGGTATATTATACCAGATTTATAACAAATTTACGGATGAAGTTACCGTCTTCATGGGTCCGGAAAACGGG

CGAAGGTTTTTCACAGTAGATAAGTACCATGGTAAATCCAACCTTGGAATTTGGGAGGATCCAAAGTGTGATAGTGTACA

GGGTTCTTCTGAAGGCGTTACGTACCATCAATTTGTATCAAAAAATGACACATTGAAGTACTTGAGAAAAACCATGTGTC

GAGTGACACCGTTAAAATATAAGAATGATGTGGCCAAATCGGGCATGACGATGTACAAGTTTATTCTCCCAAATAATGTC

TTTTCTCATCCACAAACCGATCCAAGCCTAGAAGACTGTTTTCATAATCCAAAATCAACTCCTCTTCTTTCTGGGCTCTC

AGACGTATCACCGTGTTATTATGATTTCCCTATTGCAGCTTCATTTCCTCATTTCCTCAATGGTGATAAAGCACTACTGA

AATCAATAACGGGACTAAAACCTAATGAAGAAAACCATGGCTCGTATTTAATTGTCGAACCATTAACAGGAGTTCCAGTC

GAAAGTAGAGCTCGTAGCCAAAGTAATCTAGTCATGCATCCCACTAGTGGATTTTCAAACATCGATAAATTCAGCAATAT

GACAATACCAATGTTTTGGGCTGAATATAATCAAGTGGGTTTACCTTGGTACATTACTACTCTGATGTATTTTACTGTTA

TCGTTTTGCCGTACACGCAATCGATCGGGAGCATCCTCATGATGATAGCCGGTTTGGCAATGATCGGAAAATCAATATTT

GATGCAATATTTGGTTATAAAAAACCACTCTACTCATATAGTTCTTTGGATCTCTTGCCGTCAATTAGTTAATACGTTTT

GTAGACACCTGTCCTATCAACAAAGACAATATCAAAATTTTATTTTTATATAATTTATTTATACACATAGCTTTAAGTAT

TTAAACTTTATTTCTTATCATTTTATGTTCATCGTATTTTGTCAACCGTGTTTACTCATAAATATGTTCTGACAATTTGT

TATACCTAATAAAAATCATCTTTTTTCACCACATAAATAATATCGTAGTGTTAAATA

>contig_11996

AATGACAAGTACCGAGTTGACTGCTTACACATCAATTTCTTTTATTGTGGTTTTATGGTGTCTTCACAAATGGAATCGTA

GACACTTCGAAAGAGTAGCATTGAAAATGACGGGACTATCGTCATACCCATTTATTGGCTCAGGTTTTGAGTTTATTGGT

ACACCCCAACAGGTCATGGATAGTGTATTTAAATTGTTCAATGGTTTTGAACCACGCAAATTGTGGTTGAGTTCATATTT

CTTCGTAACCATAAGCAAGCCAGAAGACTTACAAATAATATTAAACAGTTCCAAAGCCTTGGATAAATCCAAGTTTTACA

ATTTCTTTAAGAATACTGTAGGTGAAGGCTTGTTTTCAGCTCCAGTTCATAAATGGCGACAACATCGTCGTCTTATTGCT

CCCGTCTTCAACGCCAATCTTCTTGATCAGTTTTTTCCTGTTTTTAACGAGAAAAGCCGAATTCTCATTGAAAATTTAAA

GAAGGAATTAGGTAAAACACAACCATTTGATCTTTCGGACTATATTATGAAAACTAATCTTGATATAATTTGTCAAACCG

CAATGGGTTACAATCTTGACTCTCAATCAAATAAAAAGTCTGAATTTGCTGAAGCATTAATAAAAGCATCAGAATTAGAT

TCCATGAGAATTTACAAACCATGGTTGTATCCAGATATAATTTTTTCGATTTATAGAAAAATTATGGGGCTACAAAATAT

CTACAATAGGTTGCACAAACTTCCAAATCAGGTGATCAGAGAAATGAAAAAAAAACATGCACAAAGAAAAATGCAAAATA

ACAATAACGATATAGATGCCAACAATGGAAAGCGCTTAAAAGTTTTTTTGGACACATTATTGGATTTGAATGAATCTGGT

GCAAACTTTTCTGATGAAGAAATTAGAGATGAAGTTGTGACTATGATGATTGGTGGTAGTGAAACTAGTACTATCACAGA

TTGTTTTTGTATATTGATGTTAGCTATCCATCCAGAAATTCAAGACAAGGTTTACGATGAAATTTTTGAAGTATTTGGTG

ATGGCGACCAAACAATTACTATTGAAGATACTACTAAATTAGTTTATCTCGAGCAATGTATACGGGAAACACTTCGATTA

TACCCTACAGCACCATTGTTACTCAGACAACTCCAGGATAATGTTAAAATTCATTCTGATAATCAAACACTGCCAAAAGG

AACAATATGTATTATATCTCCAATATGTACTCACCATATTCCTGAGTTGTATCCAAATCCATGGTCGTATAACCCGGATA

ATTTTGACGCTGAAAATGTTAAAAAACGCCATAAATATAGTTTTATAGCGTTCAGTGGTGGTCCGAGAGGTTGTTTAGGA

TACAAACATGCTATAGTGTCAATGAAGGTTTTAATATCAACATTTTTGCGAAACTACAGTGTGCACACAGATATAAAATT

AAGTGACATTAAACTAAAATTAGATTTATTAATGAGAAGTTCTAATGGTTATCCTGTGACTATTCGACAGAGAGATAGAA

GACCTACATATAAAAAAAAATAAAAATATTGGAAGGAAAATTTGCGATAGATAATATTAATTAATATTATCAAAAAATAA

AAAAAATAACAAGATTAAGCCAATGAGTAGTTTGTAAATGGTTTTA

>contig_12137

GAAGTGACGGACGCCGTGATCACAGTGCCGGCGTACTTTAACGACTCACAGAGACAGGCGACAAAGGACGCGGGCGTCAT

AGCCGGTCTGAACGTGATGCGAATAATCAACGAACCGACAGCTGCGGCTTTGGCGTACGGTCTGGACAAGAACCTGAAAG

GCGAGAGGAACGTGTTGATATTCGATCTGGGCGGCGGAACGTTCGACGTATCCGTTTTGCAGATCGACGAGGGTTCGATA

TTTGAAGTGAAGTCAACGGCAGGCGACACGCACTTGGGCGGCGAAGACTTTGACAACCGTCTGGTAGCTCATTTGGCCGA

CGAGTTCAAGAGGAAATGCAAAAAAGACGTACATTCCAATCCGAGAGCGTTGAGACGATTGAGGACTGCAGCCGAACGGG

CCAAGAGAACGTTGTCGTCCAGCTCGGAGGCCACCATAGAGATTGACGCTCTGATGGAAGGTATCGATTTCTACACACGA

GTTTCACGAGCCCGTTTCGAGGAATTGTGCGCGGATCTGTTCAGATCGACTCTGCAACCGGTGGAAAAGGCGTTGGCGGA

CGCCAAGTTGGACAAGGGAGACATCCACGACGTGGTGCTCGTGGGTGGTTCGACGAGGATCCCGAAGATTCAGAGTCTCC

TGCAGAACTTTTTCTGTGGCAAACCGCTGAACCTGTCCATCAACCCCGACGAAGCTGTAGCCTACGGTGCCGCAGTACAG

GCGGCCATTCTCAGCGGGGACACGAGTTCTGCGATTCAAGACGTGTTGCTCGTGGACGTCACCCCCCTGTCACTAGGCAT

CGAGACCGCGGGCGGAGTGATGACCAAAATCGTCGAGCGCAATTCCACTATTCCGTGCAAACAAACCCAAACGTTCACGA

CGTACGCAGACAACCAACCGGCTGTTACCATTCAGGTGTTCGAAGGTGAAAGGGCTATGACTAAGGACAACAATCTGTTG

GGAACGTTTGACCTGACCGGTATACCTCCGGCGCCTCGGGGCGTACCCAAGGTCGAGGTGACTTTTGACATGGACGCCAA

CGGCATATTGAACGTGTCGGCCAAGGATAATAGCTCTGGCCGCTCCAAGAATATCGTCATCAAGAACGACAAAGGTCGCC

TGTCTCAAGCCGAAATCGATCGTATGCTAAGCGAGGCTGAACGGTACAAAGAAGAGGACGAACGACAAAAGGCCAAGATC

GCGGCCAAAAACCAGCTGGAAAGCTACGTGTTCAGTGTTAAACAAGCGTTGGACGATGCCGGCGATAAGTTGACCGAGTC

CGAGAAAAACACCGGCAAACAGGAATGCGATGCGGTCGTCCAGTGGTTGGATAACAATCAGTTGGCAGACAAGGAAGAGT

ACGAGTACAA

>contig_12791

TTTTTTTAAAACGTTTTAAATATTTTATTAAGTCATCAAATAAAGGTATTTATAAAAATAAACGTACATAAAATACTAAT

CAAAACTTATTTACCTAACTTATTATTGGTAATAAAAAAAATCCCATTATTATATAATCTGAAAAAATATAAAACATGAA

GGTACATGATATAAAGAGATATTTAATATAAATAAAATATTCATCTAAATAAAAAATAAAGTACTTAAATTATATTTATA

ATTAATCTTTAGTATTGATTTTCTCTTAGGTGAAGTACTCAATTTGGCTATAAATGCCAATTTTGTTATGTTTTTCATGG

AGCTCAGACCAATTATTCAAAAAGTGCTCGAAGAGTTCGT

>contig_12910

ATCATGTCTCGCGTTTCGTCGCTTTGCGCGGCTGTCGTTTTATTCTTCGTCGTTGTCGAGGCCTCGTCCAACGATCTCGA

AGGTCCGGTGGATCATGGCTACCCGAAGACTTGGATCGCTAACTTATTCTATGAGGCGCTGGCGAACTTTACCGGGCCCA

TGGACGTCGGCAGTACGGCATGCCGCCAACAGACGCAGATGTACATCAGACACTTGAAAAACGATTCGCTGTGGGCTGTG

AAGATGTCTGATTCATGGAGTCGGTATCCCAGTGGCCTACTCGTCGGTACCACACATCAGATGGGCGTGTATGAGGAGTG

TATCAAAGTGCACCATCCACTTAAGGGCAAATACTGTGTACCATCCGTTAAAATCAGCTCGTCGACCGTCATCGATTTCG

TGTCGGGAATAAACGACCAACCACAAGAGAACAGCCATGCCTGGCATGAAATACTTGGGTTCGTAGATCGCGACAACCGA

TTGCGCAGAAATGTCATGAAATTTGGTATTTGCATACCAGCCGCTTGTACCGCAGACGACCTCCAGGTTACGCTGCAAAA

GGAATTTGACACACACTTCCTACCACACCGGATCAACGCTCAAGTCCAAGTTGAATCAATATTGTGTTCGACGGACAAGG

ACGAGTATCCATACGATAATGGATATTATTTGACCAGTTTACTAGTTGGATTTATATTCTCTTTTTGCTGCTTATCGACG

GCGTACCATTTGATAATAATAATACGGAGTAACAACGGAAAAAAGAGTAAAATGCCGGAAATATTCAACATGTTCTCCAT

AATTCGTAACTCCAGAGATTTGATCAAGTACAACAGAAACAACGATTTGAACATATTCAATGGGCTAAAGGTTTTAACTA

TGATTGTGGTGCTGTTCGGCCATAAATTTCTGTACTTTGTAATAAATCCGATCACGTACGGGATGCATCTTGAAAAGTTG

TATAAGGAAGGACCGGATTTCTTATTGACTTCCATGAATTTGATCGATCCGTTTTTTTACATTGCTGGTTATTTAATGTA

CATCATGCTGATTCCGCAATTCAATAAGCCCGGCACCAATTGTTTCCAGATAGTTATGGTGATCGTTTACAAATATCTGA

AGGTTCTACCATCGTACTTGATCGTAATGTTGATGACGGCTTTCATCATACCTCACTTGGGCAACGGACCGTTTTGGGCT

TCGAGAATATGGCCAGAAGCGGATAATTGCAAAAACTATTGGTGGGCTAATCTATTAGCAGTCAGCAACTTCATACCAGT

CGAAAATCAATGTCTCATTGCCGGCTGGTATGTGTCGTGTCTGCTACAGTTTCTCGTCATCGGTACCATCGTGGTTTACG

TGTACGTCAAACATCGAAAAATTGGAATATCTTTAATTGTTATTCTATTGTGTGCGTCTTTGGCAATATCGTTTATTACA

ACTTACGTCAACCAAGCGTACGGAATAATTCGAGTAATGTATTCGTTTTTGGAGAATCCAAGTAACTCAATGGAGTTCAG

TAATTTCTACAGACCATTTTACTATCGGGGTACGCCTTTCTACGCAGGAATGCTTGCTGGCGTCGTTGTGAAAGAGCTGA

AGAAACGAGAAATAAAATTTTCCACGCCGGTCGTCTATGTCGGGACTCTGATAGTCATCGTCGTTTGCACTTGGGTCCAG

TTGTATGGTGCGGTGTTCCATGACGTGAACAGACCATACGACGCTTTTGAACAGGCATTGTATTCAGTTTTCAGCCACTG

CACGTGGGCCGTCATATTATTCTGGGTCACTATGTGTCATTTCACCACCGGTTACGGTCCAATAGAAAAACTGTTCAACA

ACAGATTAACGGTGCCACTAGGCAGATTATCCTATACAGTTTATCTCGTCAACATCACTGTCATGATGATGATAGAAAGT

AAACAAAGAACGGCCGTAACCCCTTCATCAAACTTGTTGATTGATGGATGGATTGTTGGAGGTTTTAGGACATACATGGT

GGGCACTTTGCTATATCTGATCATCGAAGCACCGTCGGGGCATTTGATCAAAAAATTATTGTTCGGAAAGAAACACGAAA

AAAGCGATCACATCGGCAGTGCGGTAACGCCAAATCGGACGGACGCCAACAACGCCACTACAAATACAGAACAAAAGAAT

TCCTCACGACTGTAAATGCGTATTTATTTACACATTTTATGCGTGTATAATATTCTAGTATATTAATAATATTATGTACA

TGGTGTGTCAATTATGTATGTCTCCCGTTGATAATTTTCATTCAATAATTAATATTTTTTAAGTTGATGTACGTGTAGGT

ATACGATTTCGGATAATCTATATCAGGCGTGTTACTGAATAGGATCTTAATGTTTAACAGTATTATTACCTATTCGGTTC

GCCTGAATTTGACGTACATCCAAACTTTGTTTTCATACATAGTTTAGTAACAATAATATTTTACTTTTAAGTACTATTAT

TAGTTAGAATCAATTTTAATGAATATATCCATTCAAAATATCTATAATTATTAATTTCCACAAATAGTTAAGTACCTACC

TATAGAATATATTATTAAAACAGACAAACACGTTGAGACACCATGTATTTTGTACATAATATATACATATAATATATTGT

TTTATATGTATATGTATATAATAGGTCTGTATATTGTACAAATGTATTTTATTGTTTAATTTTATATTATAATATTTTAT

TGTAATATATAGGTACGTAAATACTAAGTAGTATAATAAAATTGAGGTAAAATAGGAATCTATTTCGTCTAGCCGAGGCT

TATCGCCCACCGAAAACATCATTATATATATTTTTTTATTATTATAGTGCAACGGTCATGATTTTTAAGGTGTATTTTTG

ATGAAACAATCGTAAAAAAAACAAAATAAGATAATCAAACAATTTTAGGATATGTAATGGATAATTAGCAATGATGTACT

ATGTAGGTATACAACTTTTGGCCGTTGCGGGTAACACGAAATGTGTTCACCGAATAATAAACTCGCAATAACAATTTTAC

GGGTCCGGTAAGCGCGTATTCCGACAATTTTATTATACGGCGCTCGTTTCGTTAAATACTGTAATACAACCTTCACAACA

GCAACTACAAAAGCAATAATAATAATAATAAACGATACAACTTAGTAGGACTTACCAGAAGTCTGTCCGCGTAACGACAC

GTGAGCGCTTTTTGTTATAACTGCCGAGGACTTGACGGTGACAGATTTGAAGGATATAGTATTTTTTTCATTTGTTTTGG

ATGATTTACTAGGAGCTTAAGAATGAAATGTTTTGGGTATAACAGAAAGAACTGACTAATTTAACAAACTAACCTTATAA

AAAACATTTTTAATATTTGGAGCAAAATTTACCGATTACATAGTCAATTACACACATTTTGTCAATTATCTCGGCAGGAC

TCGAAGGTACAGTGTGGTCTTTTTGTTATAATCTAACTATTATACAAGATATTCCGTCGACTGTTATATTTCTCTAGTTA

TTAACGTTTCTGATTTTTTTTTTATTTTATGCACCCGATTTAAAATTTTTAAAACAACGTTTTGGGAAAATAAACTTATG

TATTTTTTATTGGTTAGTATTAATATTTATAATATTTCACGTTTTTAAATGTCTAAATTAATTTTTAAAAGATTATAATA

AAATATATCTTCATGTAAACAAAAGTTATTGCATATAAATCATTTACGAACAAAATTATTAGCCAGTTCTATCGGTTACG

CTCCACCCAATAATAGTAGGTAATAACAAGACACTCGAACCTAACAATTTATTTCATCATCATAATTAATATGATGGATT

CATAAATATTTTATATGATGTATGCGATGTATCATTCTTCGTATTCATAGTAATACATTGATTTATTTTTTTCATCTTTT

TTATCTGTTTGAACAACTTTGATCTGTGAAAATTCCGATATCGAGACTTATGACGAGTTATAATAGTATACAATATATAA

ATCTCAATTAATCGGCTCGCTGTTAATAAAGAAAAGTAACTTATAGTTAATTGTAGTGATTTTACTTTTCAAATATACAT

TTTATTCAAACCGTAATAGCTGTAATACTTTGGATAAGTTCCAACATTATCAGAAACATATTTTCAATTTTTTTTTTTTA

ACAACAATTTATTATATAATATAGTTATAATAATTTGTAAACTTTTAAGGTAAAACTTACTAGGTACCATTATAATAACA

GTTATACGTACATTATAATACTTGTTTTGATACATAATAAAAACAATAGACCTCGGACTATAAGATTAAAAAAAATTAAT

AAAATTATAATTAATCTTAGCTCATGTTGAACAGATATAAAATGTGATAAATTTCAACTGTATTTAACCCACGATACCTA

TAAATATATTGTGTTTATTAATTATTTGTATTTTTTGTTTTATATAATTATGT

>contig_13012

TCATAATTGGCTAAATCTTCTTTGGTAATTATGCTTTTTATTTTTTTTAAATCTTTTAATACTTTAGACGTTAATGAACC

ATTGTAAATGGCATCCACACCTTCTTTCGCTATGATTCTTAGTGTTTTAGCTAATTTAGGTAACTTGTAAATTTGGCCAA

CTTTTTTCAACCGACTATATTCTTCATCGTAAAAACATTCTCTTAGCTCTGGGTCATTTTTTATTTTATCACTGCTATTC

CTCATGTTTATCTCTAGACGTTCACTTATATCTATTCCATTTTCGCATAATTGTATTGCTGGTTCGAATAACGATTCCCA

TGGAACACCGCCACCATATAATTTATATACAACAGAGAAGCCTTTTAGTGCACCTGGAACTGCAACAGCCAATCCTCCGT

ACAAAGATTTTTTGGGGTTTTTAACAAACATCGAAGTACTTGCTGCGGCTGGAGCTGTTTCACGTGCGTTTACCGTGATA

ACTTTTTTGGTTGTTGCGTTGTAGATGCTCATTAAAAAACCTCCACCGATTCCCAAGTATTCTGGACATACAACACCGTC

ACATAACAAAGTAGCTATAGCAGCATCTACAGCGTTTCCACCCATTGACATAATATCCACTCCAATTTTTGAGCAAGGAC

CGCCATTCGAGACGATTCCAACTTTATTGTATGATCCCATCGGCATACTCGACAGTGGCACC

>contig_13050

ATAATAGTTTATTGACACTTCTAAAATTAATTATACAATATATATAAAAAGTGCAATAAATTTTTTCAAATTGCTTGAAT

GCTTCTGTATTTTACAAAGTATTTTCCTACAATTATATTAATTTTCATTAAATTAAATCATATAATATTTCAAACACATA

CAACAAATTTGTTCAAATGAGCAAAATTAAATGTAAAAATACAAAAATAGAAAAAAAAATGAATAAAATAATTTAAGTAA

TCTTATAATAATATTGTACTCATAATAACTATTAGAATATTATTTTTACATTCAAATTTACTTTCTATCGAATATAAAAA

CAATGAAATGTAGAGCACGAAACAGAATAAATCACAAATGAGATGTCTGTGTTTTAACTGACATTAAATTTGAATCGTGA

CTAGATGTTGCGATATTTGCTTTTGTTTTTAAAGTTCAAGTGGACGTTTCGCTTTCTCCTTGTAGAGAATCCATCACCAG

TAGGATCTAAAATCCATGGGTAGTAATTGGGACATTCCATACAAGTGAACAGTCTGATAGTCTCTCCGGGGAGTTCTCTG

GTGGTCAATGGGCAAGGATTTGATAGCGAGCAGAAGGGTTGTCCTTTGACATCACATTTGCTGTTTTCGTCTTTCCATTC

TGTGAAATCTCTCATGTTATCTAATTCGGTTGGTGTTTGCATCCATTGAATAACTTGCAACATGTTGACGAAGTATACGT

CGTTTCTCTTCAACATTTCTTCAATGAACTTGATAAGTTCGTCTTTAAATTCTTTTTTGCTCTTAAGCCACGAAGCGTGG

AAGTGTAATCCTAACGGAGCACGGTTGGTAGAGAAATGGCGATTGAAGTTGTGTCTGAGCAAGCGTCCGAACTGTTCGCC

AGTTTGGATGTTCGAACAGGAATCGACCATGTGACAACCTGGCAGAGACTCGTCGAAAGTTGGGTCGTCACGTCTGTCAA

GCTCATTCATGACCATTTCCCATACTGGATGGCTTCGGCTAGGACAGTTTTGGGCGTTACCGTTACACTTGTGTGGCATT

CTGAAGAACAGAGTATACGGCCAGATGGGAACTCGGCCCAAGGAGGCGGTAATGGAAGCGTCGTATACAAAGAACTGATG

TTCCATCATCTGGAACTGTTTGTTGCCACCGACTCTCAAGTATGGTGCACGGATACCAACGATTGAACCGTCAGAGATGT

TTGCGAATCTTTCTGTAATAAGTCTAGCTCCAGCCATCTCGGCCAACCAATCGTCGTAAGATCCACCGGTCCAATATTTA

GGATCATCTTTGTGTGTCAATGAGAACACAGCGATTTCGTGTCCTTTGCGGTGCAATTCTTGTACGAAAGCATAGTTGGT

GTACTTGTGCGATACGAAGAACGTTCCTTTGATGGAACAACCGTTGGGGTTCATTCGGTTGGGCAACGCGTTATTGAAAA

TCTCTTCATACAAATCCATGTTGTCGACATTTACGGCACCGTTGAATGTAATAGTAATCAACTGAGGTACCTGACTAGGT

TCCAATCCACCGGGTATTCTAGTACCGTCAGCTGAGCAGAAGCAATCAGGCAGCACACATTGGTTGGGATCACAATCTGG

TGCACGGTTCGGGTCAGTTTCCACGCTGCAAGCGTTTTCATCAGACTCGTCCTTGCAATCATATCTGTCATTACAGAAAA

GATCTTTGTTCATACATTCACCGTCACCACATTGTAGTTGGTCCTTTGGACATACTGGTTCGTCTGTTTTCAAATTTGGT

AAAGCTTTTCTGGGTT

>contig_13127

GTCAAGTCGAGTCAGTCAGTCTGTTATCGCGACCCGCCGTGTCCGACCATATACTGTAGCGCAAAAAAACAAGCTCGCGG

AACCGACAAACAGCAGCAAACATCGACACAATAATAATATTATTACCGATAATATAAAATCGTTTCAGACGATTTTTTCC

ACACTCACCCAAACACGACGTCGCGCGTCGCATCGAACGCACGTAACGAGCAGCAACAGCTCTCTCGTTCTCTCTCTCTC

TTCCGGCGCCGTCGTCGTCGCGTGTAACGTTTTTTATTACCTCGCACGGCACACGATTGTAAATAATAATATTATCCGTA

AACCGCGGACCTAACCCCGAAAGCCCGCCGCCCCGCGGTGCAAATCGCGACACAGTCGTCGGATTGATGTTCTCGCGGTG

AACACACTCCGTCGCCGCGCGTTTGGGTAAGCCGACAAGTCAACGCCGACACACCTGCAGCCCGCAGTAGCAGTCCGCGA

GGCCACACGCAAACACATAGACGCCACACGCCGTCGAGATGCACAACAAAAAGGGCAAGCTGTGCACCAAGCTCACCAGT

AGCTTCCTGCGCAAGTGGTGGGTCGTACTTGCAATAGCCGTAGCCCTTTTGGTCGGCGGCCTTCTAGTGGTGGCGTTTTT

CACGGCAGCAGTTGAGCTGGTTATTGATGACCAAATCACCCTGCGGCCCGGTTCTCAGACGTTCGAGATGTGGCGTAAAC

CACCTGTGCGGCCTTTATTAAAAGTGTACGTATACAATGTCACCAATGCCGATGAGTTTTTGAATGTAGTCGCTCCAGGT

GAAGTCCGTGAGAAGCCAATCTTGGATGAATTGGGTCCGTTCGTCTACGTGGAAACATGGGAAAAAGTCAACCTGACATT

CCACGACAATGGCACTCTGACATACAACCAGCAAAAGGTTTATAGGTTCGACCCTGAGCAGAGCGTTGGTTCTGAAGACG

ATGTAGTTGTGGTACCAAACATTCCGATGTTGTCAGCCACATCGCAGAGCAAACACGCCGCTCGATTTTTGAGGTTGGCC

ATGGCTAGTATTATGGACATTCTGAAGGTGAAACCGTTCGTCGAAGTGAGCGTCGGTCAACTGCTGTGGGGTTACGAGGA

TCCGTTATTGAAACTGGCCAAGGATGTAGTGCCCAAGGAACAGAAGTTGCCGTACGAAGAGTTTGGTCTGATGTATGGG

>contig_13185

CTTTAGTGCAGTCGATTCGTACAAGGCCTGTAGGAAACCTGTGAAGGAGGCTCTTGGTCCAACTCAAAATTCTCGTTTTT

TTATTATTTTTTTTTTTTTTTTTTGTTTCTTTAATAGTACAGTTTTTAGTTGAAAAATAAATAATATTTTACTTTAACAT

AAAATTTTATAACAAAAAGATTATGCTAATTGATTGGAAGAAATAGAATAAAAATAATTTTTTCTCTCTGTGTACACATC

CGCGTTATTGAAATTCGTATAATATTTTCGAAACTATGCGATTTCACTTATTAGTCGTGTGTCTGGTACAATTTGTGTAT

TATACACATGCAAATAACCAAGATTTTGTTTATTTGACAAGAGGATTTTATCACGAATCCAATGGACTACAGTCATCGTG

CCAAAGCCAAATTTACTGCGAAAGTGAATTTTTACATGACGTACAACTATCTAAAATTTATCCAGATTCAAAAACATTTG

TGGATAAAAAGCTAAAATATACAGAATCTGAGATTTTACAAAAGTACAAAAAATTGAAAAATACTTATAACGGTAATGTG

CCTCCTAATGACGAGTTAACGAAATTCGTTGACCAGAATTTAGAAGATGGTGACGAGTTGGAGGAATGGAATCCTCCTGA

CTTTACTGAAAGTCCATCGATCACTAACCGGATTAGAGACAAGAATTTTAAACAATGGTCATTAGGATTAAACAAAGTAT

GGAAAACCCTAGCAAGGAAAGTTAAGATCGACGTGAAAGATCATCCTGATAAGTACTCACTAATATGGGTTCCGAATGGG

TTTGCTATACCTGGAGGCAGATTTAGAGAACTTTACTACTGGGACACGTATTGGATTGTCAACGGAATGTTATTATGTGA

TATGTCGTCGACAGCTAGAGGTGTCATTGACAATATCTTATACCTCGTGAAACTATTTGGTTTCATGCCGAACGGTGCAC

GAGTGTACTATTTAAACAGATCACAGCCTCCAATGGTGACGTTGATGGTTGCAAGTTACTATAAAGCAACTAACGACTTT

GATTATGTAAAAAAAGTCATATCTACCTTAGACAGTGAATTTGATTTTTGGACGGAAAACCGAATGGTCACTTTCGAGAA

AAACGGAAAATCATACACAATGGCAAGATTCTACGCACCGTCGAGGGGTCCAAGACCAGAGTCTTATAGAGAGGACTATG

AAACAGCAGAAAATTTTAAAACCGAAGATGAAAAAAACGATTTTTATGTAAAGATTAAGTCTGGAGCAGAGACCGGATGG

GACTTTTCAAGTAGATGGTTTATAACTTCCAATGGCTCGGATCGTGGTGTCTTGTCGGACATACATACACCAGAAATAGT

ACCAGTCGATTTGAATAGTATACTACATATAAATGCATTGACGTTGAGTACGTGGTATAGCAAGATGGGAAATACAAATA

AAGCCGAAAAATATTATACGATCGCCACGAACCTCCTTAACAGTATACAAGAAGTTATGTGGAGACCAGATCTGGGAGCA

TGGTTCGATTGGGATATTAAGAATAATAAAAGTCGAGAATACTTTTATATTTCTAATATTGTTCCTCTGTGGACGGAAAG

CTACAACATGCCGAAAAAATCTGTAGCCAGTTCAGTGTTGGGATATCTAAGAGACCAACATATTATCGAACCCGATTATA

GTGTAAATTTCAACGGAACGCCTACGTCTTTATATGCATCCTCACAGCAATGGGACTTTCCAAATGCATGGCCTCCTCTA

CAGGCTTTCATCATTCAAGGTCTAGACAAGACACAACAAAAACTTGCACAACAGGTGGCACAAAAATTAGCCGAAGTTTG

GTTACGCTCAAATTATAAAGGATTCGCAGAGAAATCAATGATGTTCGAAAAATACGATGTTCTAGCTTCAGGAGAAACCG

GTGGCGGTGGTGAATATACTCCGCAGACAGGTTTTGGTTGGACAAATGGTGTTGTGTTTGAATTCTTAAATCGATGGGGC

GATACGGTTTCAAACGGGCCAAATG

>contig_13642

ATCATGGTGGGTTCGATGAGCCGAATCATCGTCTTAGTGTGCATGATTTACCATTCTTCGTGTGATGTTACTGACAGAGG

ATCGAGAGGTAGTTCAAGTCCCACACAAGGAACATTTAATTGTAAATCAGAAGGGTTTCATTCAGACCCGTCTGACTGTA

CATCATTTTACAGATGTGTAAATTTTGGAGCTGGAACATACTATACCAAGTTTAAATTCCAATGTTCTCCTGGAACTGTT

TTTGATCCAGAAAACTCTGTATGCGTACATCCTTCTTTTTCTAACAGAGAAGAATGCAAAAACGTACAATTTAGTAATCC

TGAGCCTGAACCTAGCCCAGAAGAATCTAATTCATGGAATTCACAACCTAATGGCGGCTCAAATCAACTAAACTCTAATA

AACCAAATAACTCAGGTTATGGTTCAACCGCCCCTGAATATTTAAGTCAGCCGTCTTCAAACTATGACTATGGGCGCCCA

CAGTCTGGTTATAAGCCAACACAAACTGAATACGTACAACCTCAATCACCACCAATATACCAACCTCCCCCATACATACC

ACCACAACCAAACTCTCCATCTAGTAATTATAAACCTCAACAGCCAGGATACGTAGAATCTAATCAACCAAGTTATAATC

AACCACAACCTCAACCAAGTTATAATAATCCACAACCTCAATCTGGTTACATTCCAACTTCATCACAACCGGCACAAAAA

CCAAATTATAACCAACCTCCACAACCTGGTTACGCACAACCATCAACGCCACCGTCACAACAGTTCAATTATAATCAACC

ACCACAATCTGG

>contig_13746

ATTTTAAACTTAATATATAATATTATCAAAATATTTTATCAAGTTATTTTCATACATTTTGTACGATTAGTTTAACTCTG

TTTTATAAAGCAAATATTATAAGAAACTTCTTCTAAGAATCTAATAATTAAATTAAATTTCAATAAAAATTTTCTTAAAG

TAAAAAGCAATAATAAAATGTATTAATAGTTCACTTTATAAAGAGTTTTTTAATCAACTATCACAATTGATAATCTGCCA

AAGAAATAATTATTATGTAGTTCCATAAAAATTATTTTGATAAAATATTTGTTAAATTTTTACACAGAACTAAATATGTA

ATTTACATACTATTCAAATAACTTAAAAGAAAAACAAATAATACAAATATGTTAGCAGATTTGTCCTTTATTTGCGTATC

ACCCATGATCCAATGCGGTATTTGAACCATATTTTTAATACATCATAACCCATTTCAAGCCAACTCCATACAGGAACTAT

TTTAGAACCTTCAATCTCTGTCCAATGAACTGGTACTTCAACTAATTTAAATTTCAACTCTTCAGCAACGTATAGCATTT

CTACATCAAAAGCCCATCCTTTAACATGTAAATTATTAAACAAAATTCTTGCAGCTGCCCGAGTGAATAATTTAAAGCCA

CATTGTGTATCTTTGATACTACGTACACCAAATAACCAGACATGAAAATGAAAGCACACCATCAAAAAAGTACGAAATAT

ACTTCTTTGAGCCATAGATTCTGTTTCTAAATGAGCACGCGAGCCACACACAACTGCATGTAACTTTTTTGCACACTCAG

TCATATCATTTGATGCTAAGAGGAGTAATGCTGATTCTAATTGTTCTAAATCTTGAAAACTTGTTGCACCATCAGCATCA

GCAAATAACAAAGTACGTCCACGTGCACTATGCATTCCCAAGGAAACTGCTCCTCCCTTTCCACGATTTTCGTATAACTT

TAAAAGACGTATCTGATCTGAACCATACTGCCGCACATATTCCATAACTACATCACATGTCCCGTCAGTACTCCCATCAC

TAACAACAATTACTTCAAAAGTCCATTTTCGACTGACGTCCGTCAAAAAATTTAAGCATGAATCGAGCATAGGCCTTATC

CTGTCCTGTTCATTGTACGCAGGCACTATGACCGACAAATCTAAAGTGCTTTCATCACTCAGACTAGGAAACGGCACTTG

CTTGCCTTTTTCGTCCGTGAAATGTTTTTCATTGTCAGCCCTATACTCATGCGCGCAAGGGGAACGCCTGTAGTACAACA

TCCAGTTTATGTATATTACACACAGCACCGTGAGCCCGAAAAATGTTTGATATAAATACGGCAGAAGTTCAACAAAACTT

AACGTTTGCGGCGATGAACCATCACTATTTAGAATTACCGCAGCCGATCCCATTATCACATACACGTCATGCCACTGTGA

ACGAAGTACAAAGGTGAGTAATAACTGCTTGAAATGTCGATAAAGCTATTGACGAGATCCCGACTACGTTTGCAACACGA

ACAGTTGTCGGTGATACACCGCCGCGGTAAACGGCATCAGTCGGCTGTCGACTTTTATTCATTATCCTTGTCGATAAAGC

TATTGACGAG

>contig_14095

ATTTTGGCTGGTGAAATGTAAAATGTATATGTTCCATCTCAAAGAGAAAGTAGTTACTTACCAGTTACTACTTACTATTT

TATTAACGTTTTCAGAAAAAATACATAAAACCTTTTCCCATACGCCGAATATGAGGATAGATATTTTGATGAGACGTAAA

TCATAGTTTTAGATCATCGTTATAATATTATTATAGGTGTTTTAATGCCAACGTGAATAAAATTAAAAAGTCGAACGTCG

TTTTCATTTATATCGATATATATATAAGATAAATAGGCTTTAATAATTCAACGAAAAATTATCTTTAAAGTATACTTCCT

AATAAAAACTATTGTCATCGCTGATTAACAAACATATCTGTTTATAAGATGGCTCAAGGACCAGCTTTAGTTTGTAGACT

TTTGGCTTCATCAGTATCTGTTGCTCATAAAGCAGGTAAAATAATTCGCGATGTTATGCAAAAAGGAGATTTGGGAATAA

TTGATAAGGGTGAAAATGATTTGCAAACTGAAGCTGATAGATCTGCACAACGTTGTATAGTTGCATCTTTTAAAAATCTG

TATCCAAATGTAAATATTGTAGCAGAAGAAGTTGATAAAATTAGTCAAAATTTGGATGTTCCTGCAGATTGGCTAATTAC

TGATCTTGATCCTAAAATATTGGATTTAGAGTGTCCAAAATCATTACAGGGTGTTACAGAAGATCAAGTTACAATTTGGA

TTGACCCATTAGATGGAACATCAGAATTTACAAAAGGTTTAATAGATCATGTTACAATATTGATTGGAGTATGTGTTAAT

GATGAAGCTGTGGCTGGAGTAATTTATCAACCATTTTGGCAAAACCGTGGTCGAGCTCTTTGGGGTCTGGTTGGCAGTGG

TGTAGGTGGGTTTGAGTTAAAAGAACCTCCTCAAACAAAGAAAGTATATGTAACTACAAGAAGTCACTATGATAAGGCAA

TAGAAAAATTTATTGATGGCTTAAAACCTTGTGAAATTATACGAGTTGGTGGTGCTGGAAATAAGGTACTTTATATATTG

GAAGGCAAAGCGCATGCTTACGTTTATCCTAGTGCTGGTTGTAAACGATGGGATACAGCAGCACCAGAGGCAGTTTTAAG

AGCAGCTGGTGGTGAATTAACTGATGTATATGGAAATAAATACAGTTATAGCAAGGAGAATGAAAAAGATCCTTTAAATA

AATATGGAGTATTTGCAACGGCTCCTTCTTATAACCATACTGAATTCATGAAACTCATTTGTGATATTCAGTCAAAAAAT

GAAAAAAATGATTAGTTATTTATTCAACAATGTTTAAGTATGTACAATGAGTATCATGTGTTCTAATTCAGTTGAGTAAA

TTCTTAATAAACGAATATGTAATGATTTAATTTCATGTGATCTAAATTAAATCTGATTTAAAAAAAAACTTTAAATTTAA

CGATTAACAATAAAATGTATAAAACAAAATACATTCCTATAAAATATTAAGTATCAC

>contig_1410

TTAATATTTACTTTATAAATTTTAGATATTTAAAAATGTCTAAGTCACTTAAAAATATGACGATCGTAATCATGGTATCA

CGGTACTTAAAAAATCTTATGAAGAAAAACGTGTTTCAGGAAATATATATGCCATAATATGATATTTAATATTTTCACTG

TATAATTAATTGCATTTTATTAACATGTATTACAACACTCATTAGGAATCAAGTTCTCTCGTTTTACTTAAAAATATATA

TTATTAAGGAATAAAATTTTATGCAGTATAATCATCAGATTCTTTTTTTTCTTCATCTTCATCGTCATTACATCCCAATT

TTTCTTTTATGATATCAGCAGCTTTCTCTGCGACCATAATAGATGGAGCACAACTTTCAGCACTAAACATTATTGGAAGT

GCAGAAGAATCTACAACCATCAAGCCGTCGGTACCAAAGACGTTAAGGTCTTCATCTACAACGCCTTTCGAAGAGTCTGT

TGCCATGGAGGATGTGCCAGCCATTTGAATATTCATTATTGTTAAATGTTTGATAACACATTTCCAATATTCATCGCTGT

CAACGTCCGGTTCTGGGCCACCCTCGATTTTTAAAGGAACAATTGTTGGACCGTAATGTTTGAATGCTTCAGATTCTACA

AGATTTTTTACCCAATGAATAGCTTTTACTAAAGTTTCAACATCCTCGTCATCCAAAGAACCAAATTCTATAACTGGTTC

ATACTCGTCGCCATCACAGGTCTTGTTTATAGTCACTTTACCAGTATTTTTTGGACGAAGTAGTGAAATACCGATAAGTT

TAATATCGTTCTCTTCGTTAAGGTCAATTATTTGTTCCGTGATGTTGTCGTTAAAGTTAAAAGCGTCCAATTGTGAAGGC

ATGAATACAGTATCATCCTGAGAATAGTAATAAAATAAGATTTGAATATCTGGAATACCATTGCCATCAATATCTATAAA

ACCAGTGAAACTGCACAATCCTATTGTAGCCAATGGTCCAGTATGCTTCATTAAGTATTCAAAGACCATTTCACTGATAG

AATATGACTTGACTGGCTGCATTTCAAATTTAACTACTATACCTAAGAAAGTTGGATGCGCTTGTAAATTTAAACCAACG

TTATCATTCTCTACGACCACTGGAATTTTCAATGAATCAAGTAGAGTCTTTGGTCCAACACCCGACTCCATGAGTAGTTG

GGGAGATCCTATTGGACCAGCACTTAATACAACGTTTTTCTTACTCTTAACTTGAATTAGTTCTCCAACTGAATTTTCAA

ATTCTACGCCTACAGCTTTTTCACCTTCAAATATTATTTTTTTGACTAACGAATTAGCTGATATTTTTAAATTGTATTTT

TTGTTAGCATTTTTCAAAAACGCTTTTGCTGCGTTCACACGTAAACCATTTTTAACGATCGCGATAGATGAAACGAAACC

TTGATGGTTTTTCACATCGAAAAAGTCTAATGTGTCATAGTTGACTGCGTTTAACGCCTTAGAATAGACCTGTCTGATTT

TAACAGTTCTCGTGTTACGGAATGAATCTACACATAGTTTACCACCTACGCTATGTGATCTACTTACTGTTTCATGTGTG

GTGATTTTTTCAAACTTACAGTCTTCTGAACGTTTATAATACTTCAAAACAACTTCCCAACTCCATTTAGTTAAACCAGC

CATTTCAAATTTAGTATAGTCTGATTCTATTCCTCGATCATACAACATTGTATTCAAAGCCGTTGTTCCTCCTAAGCATT

TTCCCCGAATAACATGGACACATTTGTTTTCCAAGCTCTGACCAAATGTGGCGTCTTCTTTTGCGGTATAATTCCAATCC

ATCACGGAACCAATCGAATTGGCCCAAAGTCCGGGTATCTCAGATTGCATGAGTGGTACACCACCAGCCTCTAATAGCAA

AACGTTCAAATTTTTCTCGTCGCTAAGCTTAGCAGCCAAAACCGATCCAGAAGCTCCACAACCTACTATGATAATATCAA

ACTCGTCATTGTTCTTGATAGACAGGCTGTAATCTTTAGGATAAGTGATTCTAGAAGCCATACTACATTGTTCCCGGTTT

AATGAGTTTAGGA

>contig_14185

CACGGTCGGAGCGCAGTGACCGCTCGTTCGTGTCCCGTTCGCGACTTGAAAAACGAAATCCTCGTCGTTCTTACCTAACC

TTGTCCAACCCTTATTTAATCTAACCTTACCGACATAACTCGTTGAACCACCCCACGACGACGATATGTCCACCGCGCTG

CAGCTACTTCTCGAGCTGGCGACCAGCTGGTGGACGGCCGGATTGCTGGTGTCGTTCGCAGCCATTGCGTTCCACTGGAC

CACGGCCACGTTCGGCTACTGGCGCGACCGGGGCGTGCCGTACGTGCGGCCCACGGTGCCGTTGTTCGGCAACATAGCCA

GCATGGCGCTGGGCACCGAACACCAGGCTCGCATGTTCGGCCGGATCTACGACGCGTTTCGCGGTCACCGGTACGTCGGC

ATGTACCAGATGCGCACGCCGCATCTGTTGGTGTGCGACCCGGCGCTGGTGAACCGCGTGCTGATCGGCGACTTTGCACA

CTTTACCGACCACGGGCTGTACACGTCCAGGCCGGACGAGAACCCGCTGGCCAACGGTCTGTTCAACATGACGGGCGCCC

AGTGGAAGGTGATGCGGCAAAAGCTCAGTCCCGTGTTCACGGCCGGCAAGCTGCGGCACATGCGAGGCCAGATAGCCGCC

TGCAGCGAGCAGCTGATGCGCAACGTGGCCGCGGACGTGCCGGCCACGGGTGGGCCGATAGAAGTGCGCGACGCGCTCGG

AAAGTATTCCACCGACGTGATCGGCACGTGCGCGTTCGGACTGCAGCTGAACGCCATCAGCGACGATCAGTCCGCGTTCC

GCCGGTACGGCAAGACCGTGTTCCAGCCGTCGTTTCGATTCCTGTTCAAAGAGCTAGCCTGGCTGATCTCGCCGGCCTTT

CGACGCGCGCTCCGCATCAGCGACTTCCCGCAGGACGCCGTCCAGTTCTTCACAGACGCGTTTACCGACACGATGCGGTA

CCGGCAGGAGCACGGCCTGGTCAGGGACGACCTCGTGCAATCGCTAATCCAAGCCAGGACCGACCTAGTCGTCAACAAGA

CCGAACCGTCGG

>contig_1429

ACTGGTGCAGGATCTAATCAACAAATAAATAATTCTGGAAATAAACCAACTTCGCCAATGTCATCAACATTAAAACCAAC

AACGCCAATTTATACAACAAAAAAACCAACGGTATCGACAACTCCGAAACCAACTACTCCGCAACCAACAACTTCAAAAC

CAACCACTTCAAAACCAATCACAAATAAACCGACCACATCTAAACCAACCACTCAAAAACCAATCATTTCAAGTACCACG

CAAAAAGTTACAACAACGACTCAGTCAACATCTACTTTACCAACAACTACAACACAAAGAGCCACTACGACTACCACTAA

AAAACCAACGCAATACACTTCAACTTCTAAACCAACCACTCAATCAACTACTAAACCATCTGTTACGACAACCATTAAAC

CTATAAGTTCAAAACCAACAACAGTTGCACCCACAAACAAACCTGTATATCAATCATCAACAACTATTAAACCAGTAAGT

CAGCAATCTACGAAAATACCACCAACCACATCATCACCTACAAATCATCAAACGCAACAACCTTCAACAACAGACACTTG

TACTGCAGAAGGATTTTTCCCAGATTCTAAAGATTGTAACAAGTTTTATAGATGTGTTGGTAATGGAAAAGGATTTACTA

AATATAACTTTAACTGCGGTCCAGGGACTGTTTGGGATCATGTTAATAATGTATGTAATCATCCATGGGCAGTGGATAGG

GATTGTTCTAAATCAAGTTCATCGTCTACAACTGGATCAACAGAATCAATTACATCTAATAGTTCTGAAAATCCAATATC

AAGTCAACCTAATAATCCAGAAACATCCACAAAATTGCCTACAACTTCTTACCCTCAGTCATCAACAAGTAGTCAAACTA

CTCAAACCCAGACTTCGACAACTACTTCTAGTCAACAAACATCTTCAGTCACAAGTCAAACAGTTGTATCGACCAATAAT

TCAACTGCTCCTTGTAATACTTCACCAAAACCACCTAGTAAAATAGTGTGTAATGGAAGTGGATTTTTCCCTCATCCCGA

TGATTGTAAAAAGTTCTATAGATGTGTCGACTGGGACGGTGATAAAGGTGAACGATTTTCTGTGTACCACTTCGACTGCC

CCGAAGGAACTATTTTTGATCCTAGCTTAAACGTTTGTAATCACGAAGCTTCTGTATACCCTCCCAGAGATTGTTCTGGA

TCAAGCTCCCCTCAAATTAGTAGTACTAGTACTCCAACAACTGAAACATCTACTACCACTCAAGCAAGTACTATAGTAAC

AACTGAAGGTCAAACTGATACAACAGGACCTTCTGAAACTAATGAACCAGAATTAAAACCAACTTCAAGTTTACCAGAAA

CAGAATCTACTACATCTAGTCAACCAGAAACTAATCCACCAACAACTAATCCTACAGGAACTTATCCTGTAACGTCTAGT

GTAACTGAGACTACAACGAGTCAAATAGAAACTAACCCTCCTACAACTAGTCCAACGGAAACAAATCCACCTACGACTAG

TCCAACGGAAACAAATCCACCAACAACTAATCCATCGGAAACAAATCCTCCGACGACAAGTCCTTCAGAAACAAATCCAC

CAACGACAAATCCATCAGAAACAAATCCACCTACAACAAGTCCATCAGAAACTAATCCTCCAACAACTAATCCATCGGAA

ACAAATTCACCTACTACAAGTCAAACAGAAACAACAGATTCTGAAACAAATCAACCGACAACAGAAACATTACCTGCCAC

AACAACTTCACCAGAAACTTCGAACTCCACTGACAAATGTCCCACTTTAGAGCCGGATCAAGTTGCATTAGTGTGCCCAA

CAGGCTTTAGAAGGCATCCAAAAAATTGTAACTTATTTTATCAGTGTACAATAGCAGATAATAATGACATAAAGGTATTG

GTTCTCGCGTGTTCAAATGGTACGTACTATGACCAAAAGAGAATACAATGTTTACCACCAGATGAAGCTGAATCATGTCC

ATTAAACGATGAAGGCCGGTCAGAACCACTTGAAGAGATATTACCAACTGTCCAAATTCAACCTTATCGGCAATTATGTC

CATCAGAAGGAATGTTTGGTTCTCCAAATGATTGTCAAACATTTGTAAAATGTAATAGAAACGCTCGAGGAATTCTCAAC

GGTCAAATGTTTCAATGTCCCGAAGGTCAGAGCTTTTGGGATACATCTAAAAAATGTGAGAAAAATAGGAAAATTCCCAT

GTGTAATAAACTCGGCCCTAAGCTAAATTGGCAAACGAGTCTAGCCCCTGTTCTTGCTAATTAATGAAATTTAATTTTAT

AAACAAAAATTTCAAATAATAATGTATAATTAGCTCAATCTAAATGTATACTAGTTTAAGCTTATGATAATAATTAATAT

GTAATTAAGTATTTAACCTTAATTTAGATTAGGTACAATTTGTCTTAATTTTGAAAAATGTTTCAAAGATTTGCCTCGGC

AACAAAAATACAAAATATAAAATTGATTCACTACGTTTAGTTT

>contig_1432

GTTTGTATAATAATAATAATAATAATAATAATAATAATAATAATAATAGTTGTTATATCCTAATAGTCTTAAAATTTATA

AACTCATAAATCACATATAAATGTATTTAAAATTACGTAAGTCGTATGCTAATTATGTCTTTGACAATACCCCGCGAGAC

GATGCCGATAACGACCTTTTGACACGACACAATTGATGGTACTTAATTTTTGTTTTATGGATGTTTTTTAAAACATTTTA

TTTTATTTTTTAATGTTATTCCACAAACGTCCCGCCGGGTTTTACAAAGAATCATAAGCTTTCGTCGTTACGTAATTTAT

TAAGATATGTATTGCGAATGGTTTTTAAGCTTAAATATTAATGATAATAATATTATAATCAATCGTATAATATGTAAATA

ACACTCTTCAAACATTCCTATAAGTATATATAATAACTAAATTATGACTTAAAAGTTAAAATCAATTTTTTGTTCAGCTA

TAGTATTCCTCATCTACAAAATTCCTAAATTTAAAATCGTATTTGGCTAATAGTTATCATATTTATATAGGTATATTATT

AAATAGCGCATTTAATTTCATCTTTATACGTGCATGATATAATATAGTAGATACTAAATACTTATAATATAAGAAGCTAT

TATAAAAACCGAAAATAAAATCAAAATTTAAATAGGAAAATATCACGTGAATAAAATATATAGCGGAATGGATATACCTA

TGCGGTTGAACAATATTGTATAATAGTATGTATAATTATGATATTTTTATGTGCAACACGTGTACGATGCAGTGCCTGTA

CGTAAAATGCTCGCCGACCAGTAATTTTGATTACTTACATAGCTATATAATAATATTATTATATTATTATGTCTAATATT

GCTGCCATATAGACCGCTTTATGCCTTATGCTAATAGACAAGAGGTGATTGCACGTCGGGGTAAGAAAGATAGAAGGCGT

TTTTCCGTAACTGAGTGACTGTAAATGATCGTTGCCATAAAATATATAACATCATTGATTATAAATACCAGTATTTATAA

CCAATGATAATATTATGTATTTATGCCATTATATATTATGAGTGTGTGTTTTTCCTTTACTTTACCATCCGCTGTCAGAA

TTCGAATGCTCGAATAATTAATTTTGAATTTAATTTTTAACTAAAACATGGGTTACAAACTTACAAATGTATTATTGACG

TGTTAAAAGAGATGTTAATAGGTTTGTTTTTAATGTTAGACGCTAGTACTATGGATATCCCACGTTCAAGATAAACATTA

TTCATCCAACTACGAGGATTAGCTTGTATCAATAATAATAAAAATTGGTATCAGAAATAACTGTACTAAATTAACAACTA

ACGTTTAGAAATGAATAAAAATATCTGTGATTCCCTGTTCTGTATCTTTCTATCGGTACGTATAATTTTCTGTTTTAATT

TTAATAAAAATATCATACATTTTTTTTATTAATAAAGTCAATTAGTTCTAACAAATATTTACCTTTTTTCACTTCTGAAA

CACCGACGCCCCCGGAAATTTAATACGCGTGCAGTAATTTGCATAGTGTATTATCGTAAAATTAGCACATCTGGTGTTAA

GACGGGCAACACCATTGCAATGTGTTTATACACATGTCATAATGCTGTTGTCATTCAAATTCAAATTTCTTGTCTATTAT

AATATAAGGTCTATATGGAGCCGCCAAATTTGTCGTAGTACTAGGTGCGATGGGTATGATGGGTTGATAACCTATATGGT

AAAAATTTCGATTTGTCGTTGATAGATTTAGGACGATTTTAAACAACGCGGTCGTTTTAAAAATATTCCCAATGGACGGT

TGTAACCCCCAATAGTTAAGTTTCTTAAATCAACAGCCCGACGATAATGATATGGAGTATGGACACACAACAAGTAGCTA

GCTGGCGGCACACGCACGCGTGTTATATTGTGTAGTGACTTATAATTTGACGGTAATTTTATCGTGGCAAAGGAATAAAA

AAAAAAAATAAATAAATAAAACTTAACGATTTAGCTCAAGGTTTCGTGTTCTGTGTTTGTTTAGATGGGGCTTTTTTTCC

GCGGCTATAATTTCAAATTAAAAATAATTACTATAGCGCTATACGCTGCCATAATATAATGTATTCGACGTCACTGTCAT

TGTCGTCGAAAACGATCGATAAATTCCCGAAGCGTGTCCTAATCGTTTTACCTATGTACACCATTATTGTACCTACACCG

GTTACACGACAACCACTGCGGTGTCCGAGTGGGGCACGCTTGCGTTGTACACTCTACGCTGCGACATATGGTATAGATTA

TTAAATTATATACGACACGCACGGGGATAGATATTTTAAAAGCGTATAATGTCTCCGATGAAAAAAAAAACACTTTACTT

AAAATTGCATTGCACTCTAAATAGCACTAAACGCTAAGAGTCGCGCTACACGTTTTCCATGTGGCTGCGACGATAAAATA

TAATATTATTATTAATATATTGTTGTGTTAAGATTAAATTATCATAACCTATTTATAATGTATTGTAAACTTATATGTAC

GATACGGATGATATGTGGTTGATATGTTGTCGGCAAAAGGTCCGACGTTTGTCGGCCAAATTGAAAGAACGATAAACGAA

AAATATACATAAAAAAAAAAAAAATAAAAAAAAAATATACAAACACCTGAGTATGTAGGTGATATTATAATATAATATTA

TTATACTCGTAGTCGGCACATTGAAATCGTATTGTACGACCGTCTCGTCATGTTGGTTCGATATCGATATTAAACATATT

CGTATCGCATCAATAATGACGGCTGTTGTCGGGATGGTGCGTCGTTTCGTTATTGTTTTTTTCAAAAGATGGAGAACAAC

ATAAATAAATAAAAACAAAACAATAATATCATGCGACGAGTGTAGGTCTTAACACCACGAATTTTGACGCTCTCCGTTCG

ATCGATTTCGGTGGTTGAGATATTCAAAGCTTAGTAACTGGTAATGAGTTATCATCGCGTTTTTGAATATTATTATTCAT

ATAGGTACATGTAGGTACCTAACATTATTTAGGTACCGGTCTTAATGTATACCGTATATACTCGGGTCTGGGCCATGTAC

ACAGTGCACATTGGCACGTTAGATATAGTATGTATTTACGAGTAATATCAACGTGAGTTCGGGAGGTCATGGCTGTTGAA

TATGCGTATGTATAACACAAACATATATTTATATGATTATGGCGTGAATATTAGATAGCTCGTGTGCATTTGCAGAGTGT

AAGCGCGTGTGTGTGTATGTATATCTGTGTGTATGTGTGTGTGTGTGTTTGTGTGGTGTGTGTTTGTGTGGTATGTGTGT

GTGTGTGTGTGTTTGTGTGGTGTGTGGGTGTGTGTGTGTGTTCGCTATTGTGCTTATTTGAATTGGTGTAGAGCAAAGAC

GACAACTATTAGGACGCGGGACAGGACTCTAGATTCGCCGATCCCGTTGACTGACGACGAGTATTTTTTACCCACGCAGT

TCATTCTTTCCACTGGCAAATTCAAAACACTGAATTCCCTGTGTTCATCTTCCATCAGGCAGAGTATGTGTTTCTTTTGT

ATGATGTCGTCGTCGTCTCGGTGACGTAACTCTTTTAGCCAATCCCTGTACCAGCGCAGGTCACAGTCACAAATCAGTGG

GTTATCCATAATATCAATAACAGTTGCGGTGTCTACGATTGCCTCGAGCGCTTCTTCCGGAACTCGCGTCAGTTTGTTGT

TTTGCAGGTTCAATATTTCCAAAGAATTCAAATTCTCGAACGCCATCATTGGTAACGTTTTAATGTCGTTTTTTTGTAAA

TTCAAAAACGTGATCGAATCGCCAAAACCTGTGAAAGCATCTTCGGCAATATGTGATATGTTGTTTGCTTTAAGGTCCAA

GTGTCGAAGTCTGTGCAAACGACGGAGCGATTCGCTTGGTATTGTATGCAAGTTGTTGTCACCCAATCTCAAATATTGAA

GATTTTCTTCTAAACCAAAAAAAGCCTCTTCGTCAATACTAGATATTTGATTCCCGTCTAACTCCAACGAGTTTAGCAGA

GGCAAATGTCGAAATACTTTGGCAGGAATGTGTTTGATATAATTGTGTGCTAGGATCAGCATATCCAATGTTTGTAAACC

TATGAAATCGCCTTCGCTGATCGACGTAATCTTATTTTCTTGAATTTCTAATTTCTTTAAGTTGTCCATTGTTTGTAGAG

CTCCAGTCGGTACCCGGCTCAATTCGTTACCACCAATGTTCAATCGTTTCAGTTTTTTTTCTGTGCCGGCGAAAGCATTA

GCCTCGATGTTGGTAAGCCTATTTTCGTACAATGTCAATATCTCCAGCGTGCTCATTCCCTCAAAAGCATTTGCATGGAT

AGCGCTGATTTTGTTGTGGTTCAAATTAAATATCAGCAATCGATCTAGGCTTTTCAACGCTGGAGTCGGTACGCTGCTTA

ACGAATTCTGAGACAAATCTAATTGGGTGAGCGCTTTTCCGATAGAGCTCAGCGACGACTCTTCGACTACGGCCAATGTG

CTGTTGTGTATGGTAAGGTGCCGGATGTCCATGCCCAGGAACACGAAACCCTGCAACTTGGGCAGGTTGTTGTGCCGGAG

CTTCAGGTAGAATATGACCAGTGACGGTTTTCCCTTGAGCACGGCCATCGTCTTGGATATGTGCTGCAAGTCGGTGAACT

CGCACAGTATGTCCAGACCACCGTTCTTCTTCACCGTGCACACACACGGTGCAATCTCTCCGTGCGTGGGACATTGCTGA

ATGACCGGGCCGCTGGCGGACCATGCCGGGTCCACGGATACGGCCAACAGCAGGCCGCAGAACACCAATACCGACGTGGG

CACCATCGCTAGCCGGTGGTCGCCATACCTTTTAATGGACAATTCAAATTATTTTTGCGGCCAATGCTGAAAACAACCTC

CAATTTGTTTCCTTTCGGTGTTCAGTGAGGTTTTAATTAATTTTCATTGTATGTTATAGTTCCCAGACAGAAAGAATCAC

GTACGTAACAGGTACGTCGGGCGCGAGAGTAGAAGGGTGATCCGGCGGCGGTGGTGCAAGTGGTCGACTGCGGCAATGGT

GATCGTTGGACGCGATCAGACT

>contig_14346

CTTAGGATACAATTACAGTTTTAATCAACGAGAACAATCCTGACAATCAATTAAAATAATTACGTGAACAATGAAAGTTA

GGCTTCGTTTGCTAATACTGCACAAAAACTATTTGGAACTATGATTGTATAACTTGATTATACATTTCTATAAATAAAAT

AATATTATTTTGAGTTAGAAATTCTATAGGAATTATTTTTGTAAGCTACTAATGTTGAGTTATATATAAAGAAAAAATGT

GGTTTCATTTTCATATTCTAAAATATAATTTTATACAAAGATCATTAAAACGACATAAGGCTGACGAACGTTCGTCGTTA

CCTTCGAAATGACCTTGAATCTAACCCCATTATATTCTTAGTCAGGTAATAAAACCAATATACTGAACGAAAAGATCTCT

AATAATATAAACCGGCCAGAAATAACATACCTACTAGTTATTAAAAAATATGTATATAATAAATTATCCTATTGTGTAGA

CAGGCTAACAACACAATTATTCAACCCCTTATGATAGAAATAATCTTTTATTCGTTTTGATTCATTTAAAATGATTTTAA

AAACCTCCAGCAATGGATTTAAAGTAAAAATTATATATTGTTTTGTTTCGAATGTCCACTGTTTTAAAATTAATTTATAA

AGTTACTTATATTAAAATAAATACAATCTAAAAACTTATCAATAAATAGTATATATATAAAATAAATTTAAAAATGCACA

GTCTTTTAATACACAAGATCTTCATCTGATCAAATTTCTGTTCATCTTCTTTCTTCAATCCTCTTGTTATCGGATATCCA

CATGGCGTACTCATTCACTGGTGTATAAATCAACGACCCTACGCCAACGCCAGTTTGGACGCCAGCCAGTTTACCGTCAC

ACCACAAGCTGCTTCCCAGTCGCCCTTCTTGATTGTATTGCATCCTCCAGTGATGGCACGGCAGTATTATATACGGATTG

CTAATCAACTTTCGTAGCTGTACTCGACCTTCACAGTTATCACTTGATTTCACTGTATTCTTAAAATTGGAGTTTTGAAA

AGTCCTGCATACTTCATTTGTTAGAGAATTTTTTTGGCTAAAACACGGAGTACATTCTTGACCTTCAAGTGGAACAGTTA

AACTAACTAGTGCTAAATCATTGTTTTCTGGTGTAGTTTTAGAAACTGGAGATTTAAAGTTCTCGTGCACTAATATATTT

GAAATACTCAGTGTAGCTCCCACTGAATCTTTTCGACTTACTGTTACTTTCCTTATACCTGATTTATTTAACCTGGAAGC

GCATGTGGCAGAAGTTAGTATCCATATGTCATCTAAAAGCGTTCCATAACACATGGGTTTTTTATCAACCAAAAAAGTTA

CTTCACTTTTACAAGATTTTTGACAAATACGTTTTTCAGCTTTAAATTCATCATCAATGGTTGACTCTTCCGTTTCTAAT

TCAGTTGTGTCCAATATATTTGTTTCATTGTCTTTTTTTATTTCTAGAGGTATATCTTTTTTCAAATATTGTTCGTCTAA

TGTTTCTTCTGGACTTATTGAAGGATCCACAGTAGTAGTATCTTTTATTTCAATAATATCATTCATTGTAGGGTTTTCAT

TATTTATAATATTTCTAATCGTATTTTCTTCCATTTTTGTTTCATTATTTAATATAGGTTCTTCGGTTTTAATATTATTT

GCAGTTTCATTTATTAATTGTTCGACAATTTTTTTAACCACTAACTCAATTTCATTTAGTTTTGCTAATTGATCACTGTT

CATTAGTGAAGAATCTATATTTTCTCTTGTATTTCTTCCAAATTTATTTTTAGTTACAATATTAGTTGTCTTTTTGGTAT

TAGTGTCTATTATCGGCGTCCCTGATTGCTTTGATCTTTGATTAAACTTACAACAAGCAAAAGGAGCACTTGGAGTGAAT

GCACGTTTCAAACCATCACAGTTACCAGTTGGGTCAGGCAAAAAATCATCATCCATGGTGCAATCACTACCCAGAAGACA

ATAACCGAGGGTGTTCCCACAAGTTTTTACGCCTGATATTACAAAGAGCCCTGGGTCTACTAGTGGCATGTTCATCGATG

CTGGCAACGAGGTCACGATGCATATAATGGTTATCAGGAATAACATCACGATGATGGGCCTAAAACAGAAAAACAAGTCT

ACCCGTGTGTTCAATGCGGAAGTAACGAGTCGCGAGACGTTCAGCTACAAAGTGTTTAGCAGTTTCACAGTTTTGCGTCT

TCGTGAGATATCGGAACAG

>contig_14387

AAGACATCGTCAGTAAATAATTCAACTTGGCTGTTGTTTTTTTTTTCATCGTTTCTTAATTTGATTAAAATCGAAATGAA

ATCGTTACGATCTTCTTTCTCACTTTTACGCAGCTCCATTGTATCTTGAAATAAATTTGAATAGAATTCATAGACCAGAT

CTGGCATATTACGTAACCTAAATAAATCCACAAGCCATCCTAGATCAATTAACCTCATAAAGTTTAATACAGACGATTTC

AGCGTAGGTGTAAAAACCGCTTGCCCAGCTCGTTGGTATTCTGTGTTTGAATCAAAAAGTGTATTGCAATTTATCCCAAA

AGCACACCTTCCAATTGTATCGATTGTAAATTTTGATGATATATCGCTGACATTAACAATACCTTGGCCACCGGACGTGA

GCAGCAACAGTTTGTCGACGAGTTGGCCGGTGCACACGTGCAGACTGGCGAACATGGCTTTGAGCTTGGCCACGGTAAAC

GTGGGACTCAACTTGGCCCGAACCGCTTTCCACTTTTCGCCCCTCAGGTTCACCAGACTGTTGAACAGCCCGTCATGCTC

GAACGACGTCCTACTCATCACCCGGTCGTGGAACGACGCGAATTCTGTGGCCAGCACCGAGTGTACCAGCACGGGGTCGC

GCAGCACCAGCATGGGGTGTCGGAGCTGGTACAGCCCGAAGTACCGGTGACCGGCGAACCGCCGGTACAGCGTGTCCACG

GTGACGGTGCCCGACTCGCGGCCCAACGTTGGCCCGGCGAAGTGGCCCAGCAACGGTGCCGGTTTGGTGTGCGGAACGCC

CAGCGTCGCCCAGTGGCCATAGTGGCGCGTGCAGTACGCGTACGCCAGTAGCACCGACACAACAGTGGCGGCCGCGGCGA

CCGGCAAGATGTCCCGCCAACAACCGAAGCAGATCATTACGAACGACGCAAACGGTCGGTGCGGCCACTGCAGCTATATT

ATTGTGGGCGATGTGCTGCACTATACGTCCGCCGGAAACGGCGTCGGCACTGACAGTCGGAGTA

>contig_14561

GAAACTTTGAGAATGCATCCACCTGGCCCTGGTCTATTGAGAGTATGTACCAAAAAATTTAAAATACCAGACAGTGACAT

AACTTTGGACATCGGTATGAAAGTACTTATACCCACATATTCACTACATCACGATCCTGTGTATTATCCAAATCCAGAAG

TGTTCGATCCCCTGCGGTTCACAGAGGACAATAAAGCTTTGAGACCGAATGGAACGTTCCTGCCATTTGGCGATGGGCCT

AGAATTTGTATCGGTCTACGATTTGCATTGATGGAGGCCAAAACAGGACTAGCAGAAATAATATCAAAATTCGAAATACT

TCCATGTAACGATACAAAAATTCCCATTAAATTAAATCCAAGATCCATTCTACTGACACCAAATGAACCAATATGTTTAT

TGTTTAAACAAATTAAGTAATTTTTTCATTAAAAATAAAAATA

>contig_14831

GGTGCTTAACTCAACTGATGAAAATAAATTTAAAGAAGAGGATATTATTGCAAATGCGATCATGATGTTTTTGGCCGGTT

TTGAACCCGTGTCTTCCACGTTGAGTTTCTGTTTGTATCAGTTAGCACTTAATCAACACGTCCAGAACAAAATGCGTGAC

GAAATGAATTCAAAATTGGAACAACACGGAAAACTGAACAATGAATTTTTGGTGGATCTTCATTATACCGATATGGTTTT

GGCAGAAACTGGACGTATGTACGCAGTGACAAACGCGCTGTTTAGAGAAGCTGTGAACACATATCACGTACCTGGCGGCA

AGCTGGTGATCGAAAAGGGAACCAAGATCATGATTCCTTTTCACAGTATTCATCACGACCCAAAGTATTACCCCGATCCG

TATACCTTTGATCCGCAAAGGTTTTCACCGGAAGAAAAGGCGAAACGACCATCTGGTACTTACTTCCCGTTTGGCGACGG

ACCGCGATTTTGCATAGGAAAACGATTCGCTGAATTAGAAATGAAAATGGTTTTGTCACAAATATTGACGACATTTTATG

TATTGCCATGTGAAAAAACTGAAGTTCCGCTTAAGCTACAAAATGGATTACCTATGATGGTGGCCAAAAATGGCATTTGG

TTACGTTTTCAATCCATTTCTTAATGACTTCCCGAAGTCTATAATGATTAATGATTATATACTCTGAATACTATATAAGG

CAATAATAATAACTATTACAATACATATATTGTTGTATTAACCTTAAATTTGATTGTAATTGTAATAAATTACTTAATAT

TTTTTATTATAAATGTCCATTTAGATAATTCTTGTACTATATTGGTAATTATAACTTTGCAGAAATAAAAATCTTAATGT

TGAATCACAAAAAAAA

>contig_1505

GTTAGCCATAATGAAGATAACACAAAAAAGCCGTACGACGGTCAATCAAATGAGTATCATAAGCACACGTCGGAAGGCCC

AAAAGAAGATTATACTAAAAAACCTTTCGATGGTCATACAGAAGATCATACTAAAGAAACATATGTTAACCATAATGAAG

ATAACACTAAAAAGCCTTATGAAAGTCAAACAGAAGAGTACACAAAAAACCCGTATGACCACCCAAAAGAAGATTATACT

AAAAAACCTTTCGACGGTCATACAGAAGATCATACTAAAGAAACATATGTTAGCCATAATGAAGATAACACTAAAAAGCC

TTATGAAAGTCAAACAGAAGAGTACACAAAAAACCCGTATGACCACCCAAAAGAAGATTATACTAAGAATCCGCATGACG

GCCAATCAGGATATATTACTAAAGAACCGTCTGTAGATAATACGTACGAGGTGACAGATAAATCAAATAATTCTGAACAT

ATTATTTCAACTGAACCCGATAAAACCTCTTATGATCATCATCATGTACAACATAAATGTGGTACTTGGAATAAATATGG

AGCTGGTTTTAGAATTGGAAATACACCTGATGGTGAATCACAGTTTGGTGAATTTCCCTCAATGGTGGCTATATTCAAAG

AAGAATTATCAAAAGACGGCGAAAAAAATCTTGCACTGAATTGTGGGGGTTCGTTAATCGAAAAAGACGTCATTTTAACA

GCCGCTCATTGCGTGATAAAGAAAGATATTTCAACATTGGTCGTTCGCGCGGGTGAATGGGATTTAAAAACAGAAAAAGA

ACTACTTAGCCACCAAGATCGTAGAGTATTAAAAATTGTTACTCATCCTGATTATTACGCAGGAGGACTTTATAATGATG

TGGCTTTAATTTTTACCGAAAATCCATTTTCTTTACAAGATAATATTCAACTTATGTGCTTACCTTCGTCAAATGATATT

TTTATAAATGACACGTGTTACTCGAGTGGTTGGGGTAAAACTGTTTCTTACAATAATTATACTATTGTTAAGAAAACAGA

ATCGGGAAAAATTAAAGAATCTGGTCTATATCAAGTCAGTATATTGAAAAAGGTTGAACTCCCGATTGTTCCTCGAAGTA

AATGCATGGACATTCTCAGGACTACTAGACTTGGACCAAAATTTGTATTGCATGATAGTTTTATATGCGCCGGTGGTATA

GAAGGAAAAGACACGTGTAAAGGTGATGGTGGCAGTCCGTTGATGTGTCCTTACAAAGATGATCCAGATCATTTAGTTCA

AGTTGGTATTGTAGCTTGGGGTATCAATTGCGGTCTGGTAGATATACCTGCGGCTTATGTTAACGTGGCCGGTTTCGTGT

ACTGGATTAAAAACGAAATAGAAAAAAGAGGATATTGAATATTTCTATAATACTTATAATGTGTAAGAATTTTATTTAAG

ATAGATACATACATATAAACATGTTCAAATGAAACTTTTTATAAAAATATTTATTTTTTTTTATACCGGCTTTACTTAGA

AGATTTTATACATTCATTATTAAAAGACTTAGATATTATAGTTTTCATAAATTTAAATTATAAGAATCTTCATTGCTATT

TTAATGATAATATTCCATGGAATATACTCGTA

>contig_1530

GTAAAATATAATTTGCCGATTTTAATTTTTAATGATTGATGAATAAATCCTTTACTTTCTAGTTAACCAATTGCCAGTCC

AAAGTGAAAAATAAAATTCTTTTTTTTAACCCTATTTGTTGAATTGGCGAAAACTCAATAAAAGAGTATGGCCGCACAAC

TGTTCAATCGAATTGGTCAATTAGGTCTTGGTCTAGCAGTAGCTGGCAGTGTAGCTAATACAGCATTATATAATGTTGAT

GGTGGTCATCGAGCTGTCATATTTGACAGATTTACAGGAATAAAGAACACTGTGGTTGGAGAAGGAACACATTTTTTAAT

TCCCTGGGTACAGAAGCCAATCATTTTTGATGTCCGCTCACGTCCCAGGAACGTCCCAGTCATCACTGGTAGCAAAGATT

TGCAAAACGTTAACATCACCCTCAGAATATTGTTCAGACCCCTTCCAGAACAATTGCCAAAAATATACACTATTTTAGGT

GTAGATTATGATGAACGTGTCTTACCCTCAATCACCACTGAAGTCTTAAAGGCTGTAGTGGCACAGTTTGATGCAGGCGA

GCTTATCACTCAGAGAGAAAATGTTTCTCGTAAAGTGAGCGAAACTCTTATTGAACGTGCTGGACAGTTTGGTGTTGTGT

TAGATGATATTTCAATTACCCATTTAACATTCGGTAAGGAGTTCACTCAGGCTGTAGAATTGAAACAAGTGGCTCAACAA

GATGCAGAAAGAGCAAGATTTTTGGTAGAAAAAGCAGAACAACAAAAACAAGCTTCTATTATTTCAGCCCAGGGAGATTC

AGAAGCTGCTTCAATGTTAGCAAAATCATTTGGAGATGCGGGTGAAGGTTTGGTCGAGTTAAGAAGAATTGAAGCTGCAG

AGGATATTGCTTATCAACTATCTAGATCTAGACAAGTGTCATATTTCCCCCCAGGACAAAACATTCTTCTTAACTTACCA

GCACAATAATTTCCAATAATGTATTTAGAGACTATTTAGACAGTTATGATTTTTTTTTTCGCCTCAGTGTAATATAAAAT

CCAACTATACATTAAATCTGTATTTTTTTTTTATTATTATTATAAATTATAAAATATGATTTTGTTGCAATAAAATCATA

GTTATATAGGAATGGGAACTTAAAATCCAGTTTTTTCTTATATCTTAGAAATACCAAAACAACTGCTTAAAGAGTCTAAA

TATTTTTTTTATTTATCTCAAAGCGAAAGGCTAAAAATATTTTGTTAGTGAAACAAGGCTATGTGTCATAAATTCATCAG

AGTTGTCATCTTATATTTCATTTTTAAATTAAATAAATACAAAAAAAAAAAAAAAATACAGTTTTTCTTTTTATATAAAA

TAAAAAATATTACACATTTCTTATTAAAAGTTATTAAAATTTGTTTTCTTTTATATATAATAATATAATTTATAATACAA

CTCTTAGAAATTAAAAACAAACAAATAAGTTTTATAAACAAAAATTAAAAACAAAATCTTATAATCTATTTAATAAATTA

GAAAAATTCATCTAGGGAAAGTAAACATATTTCTTCGATATTATTTTGTATGTAATGCTGTTGCTTTCTCTGAATAGACT

AGAATAGGTTTTTTGACGGCCAATGTCTGGTTTATAAATAAATAACCAAAAATAAATAAAATATATGTGGGAAGTACATA

TACCATATTTTATTATATATTTTATTTCTTCCCCAAGAACTCGACCATCTGGTTGTATTGAGTCTCCTCCGTTAACAATT

GAAGAGACTCCAAACAAGCCTTAAAGTATACCGTGTTGAAGCACGTAGATCTAAGCCCCAACCCCGACAGGATCAGCCCC

GTGCAACTGTTTAATCCACTTCTGTATCTTTTCCACTTCATCCTCAAGCAGGAGCACGTCCAGACATCCTTTGAAAAAAT

CTGTCGTGAAGCAATTTTTCCTGCACAGTGAGTGGAGTTGTGGTTCCCGGAACAAGTTGTAACAGTCCTCGCACACCCTG

TCTAGTCGGGCGAATATCGTCTTGTCGTACACGCCTTTGCACTGGATGTCGAAGAACGACCGTTTGCTGAGCGGGTGATC

GATGCCCGACAACGAAGGCGACGACGAGCCGGCCGCGCCGTTGCTCGTCATCCGGTGGTGGCCGCCTGAGTGGTGGTGTA

GGTGGTGAGCGGCCGGCACGGCGACCACGGACACAGCCATTATCGCCATCAGCGTGGCGACCAGTACTAGTGCGGGTTCG

TAGCCCATCACACTGTATTTCGACATTGATTTACGAGAAACAACGGCCGTGGCTGAGGTGGATGAGCGGGCGATGCTGCC

CGTTTGTGGCGACGTTGCGGCGGCTGTCTGGCGGTGGCCCAGAGGACCTCCGGCTTTCGGCTGCGGCGTCGGTAGTTAGC

CGAGCGGGACGACGAGCGAAACGTGTCCGGTCGCGATATAAAGTTTATACAGAACCGTCCGAGGGTGCAACGGTAGCGCA

CTGGGCCCGGTTCAGGTTTTCGAGTGGTTGAGCACGAATATGTATTCGACGAAACTGTCTCCCGACGGTTTGTACGCCGG

TATCATTTTAGTGGCGCGGAGACCATATTTCCTCTCGCACCGTGCAAAACGAACTTGGTACCTCCGGATCAGGCGGGCGG

GCCTCGGAGGAGACACACAAAAAAATAAAACGCACACACTGATGGAGCGGCGACTCGGGGATAGAGGGTCAAAGAAAGTC

TCGAAAAAAAAAACCGACGCGGTTCGAAAAAAAATTCCGAAAACGCGAGTGTTTAAAAAAAAAAATTATCAAAACGACAC

TCGTCGAGCGGATGACTTTTTAAAATAGTGCGTGTGTTTAGTATTACAAAATCACAAAATCGTATACGTATCGAGCAATA

TATGTGTGTGTGTGTTTGTGTGTGTGTTTAATTGACGGCGACTCGTTTTAAACTAAGAACGAGACTGACGAGCGTTGCAC

GTTTATGCGCCAAACTCACACACACATAATCGTAT

>contig_15324

CAGCTAATACCATTTCCAAGTAATGTAATTCTTTTAGAAATTCATCGTCAATTTCACCACTATGTTTAATCTTTGTCGCA

TTCATTTCTTCTCGTACTTTGTCTTGTATTGGTTTTTTCAATGCTAGTTCGTACAAGCAAAAACTCATCGCCGTAGACAC

TGTTTCAAAACCAGC

>contig_1566

GTTTTATTTTTATTGATCAAAAACTATATTTGATAATATTCGGAGTACAAAAAATTATAGGTATGTTTTTTAAAAAATTA

AAAATAATAAGCTTAGTTATTTCTAATTATATATCTTATAATTATATTTACATATTACACAATTTACTTAAACACAAAGT

TACAACATAACGATTAATATCAAACAAATTACAACAGGCGTTCTTTATTTTTTCGCTTTAATACCTTGTTTTAACTTGAG

ACTTAATATTGAAACAAAAATTGAAACAAATATCCAAGCCAATATTGATATAAACCCTTGCATGACTATTGGATGAGATA

ATTCCCAATTTCTTGCAGTTATTGATCTAAATGCGTCAACTGATAAAGTTAACGGGAAAAAAAGACTAATCTTTTTTAAT

ATTGGATCCATGGCTTCTAACGGCCACATGATACCACTTAAGAATACTTGTGACATAACCAAACATACTCCAAAGAACGT

AGTGATTTTCTCTTCATCAAAAGTAATACTCGCCGCAAAACCCATAAATGTCCCGCATATTCCTTGTAAGAATACAATTA

CAAAAGGTGTAATCCATGGACTTACGAACGGATGATCGAACACAATGAACTGTAGTATGAATATTGTGGTAGTTTGAACG

ATTAATATTGTAACTTGTACTACCAAATGCGAAATAATAACTTCGAGAATGGTGAGTCCTGAAGAAAGACTCCTTTCAAT

TGAACCGCTATTTTTTTCCATGGTGATTGCTAACGCCGTGAACAGAAATGTCATGTATAATTCCAATATGGATATTATTG

CTGCAGCTCCGTAATGTGACATGTTAATATCATATAATACTCCAAATACAGGCGCCATCAACGTTATTGGTACTGGATCT

AACGTCGTAGTTACTGTAGTTTTATTACAGTTTACAAAAGCTTTAGTAAGACTTTTATAACTTTGATGTAATTTTCCTTG

GATTATTGTTTTTACTACATAGTTTGACATATCTAATGATGCTTTTATTGCGGAGTCTTCTATCTCATGTGAGCTCATTA

TTGATGGATTATCAAATTTCATCCCGATCATATCGGTAAAATTTTTTTCAAATGTTACAAAACCCCACGTAACACCATGT

TTAGCGTCTTGTCTTGCTAAGTTTTCTTCATTGTATTTTGTCAAAGTTAAATCATATTTTTCCATCTCCTTCATAAATTT

GCAACTTAGCGGGATTTTTCCATCACATATATCAAACGAGTTATAAACGCAATGTGAAGGATTCTCATAGTTGACGATTC

CTACGTTGAGAGGTTTTGGGGCTTGTCCAATGGTTAAATTATAAATCATTATAGTGAAAAGTGGCAGGAGACAAACAAAT

AGAATAATTGTCCAATTTCTGACGCTCCAATACCAATGTTTCAATAATTGAGCTTTGAACCGTATTGGCGAGAAGAGTGA

TGATTTTTTCAGTGACGTCGTAGCTGGCTGTAAAATGGATTTTCTTCTTGGTTGTTCTATTGTATCTTCATTAACTGAAG

TTTGCTTTTGACTGAGTATTAAAAAGGCATTTTCAAGGGTGTCAGCATTACAAGACGCCATAATTTCTAGTGGAGGCGCT

TCAGATAGTAAAACGCCTTTTCTCATTAAACCTATTGTATTTGCAAGTCGTGCTTCTTCAATGTAATGAGTTGTAATAAT

TATTGTTTTTCCACGATTTGTTAAATCTTGTAAATACTCCCAGATGCTAGCACTAATTATTGGATCGAGTCCAACTGTAG

GTTCATCCAATATCAACAAACTAGGATCATGCAAAAGAGCAGTACATAGGGAGAACCTTCTTTGTTGTCCACCGCTCATT

GAACCAAATTGGGTCTTAGACGGTGGCATTTCCATTAAACTGATTAACTTTTCACCTTTATCAATAATATCTTTATAAGA

CATTTCGTACAAAGAACCAAAATAATGGAAAGTTTCTAGTATGTTAAATTCATCGTAAAGTGCTAAGTCCTGCGGCATGT

AACCTATTTCTTTTTTCTTTTTCACAGAAAGTTGAATGTGTCCAGAGTCCAATCTACCTCGGCCAATAATACAAGAAAGT

AACGTTGTTTTGCCACATCCACTTGGTCCCAATAAGCCATATATTGTTCCCTTTTGCACATTCATGTTAAATCCATTTAA

TACGACAGCCGCAGAGTTGTATGCTTTGTACGCATCAGTTATTTTAACTGCTAAATTGACATCATCACTAGTTTCTTGGA

CATGATCGAGATTGATTTCTTCCATCTTAAGATTTTATACTAATCCTCCGGATCAACAAAATCTATTGGGAAGTAGAACA

ATGAACGTAAGTATTATATTTTTCTAATCAAAACAAATACATTTTTTATTACTATTACAATAAAATTATTGAATGATGTA

TTACACCGTAGTACGCGGTCACTGATACTAGTTTACGAAGTGGTCTGTGA

>contig_15965

CTAGTCCCTACTATTAAAACATACAGTCGCAAACTGTTATTATATATACATTATATATACGCGAGCGCTGCGCTACTACG

GCGCGCGAGCAAAAAAATACGTAGTTAAGCTTGTCAGCATAGTAATATTATGTAGCTAGTAGTGGTTGCGATGAACTGCA

GGCAAACGGTCCTCGTACGTTCCCGGTTAGAGTATGCGTGATAATTAAAATCGGTTTTATATTCTTTTCGAATTCAATCC

GTACGGAATCGGACATTGATATCCGTTCAATTATTATTAATAATTATTTATTAATATAGACGTATTATTGAAAAACGTGC

ATTAAAGTGTTTATCCTGTTGCTTCTGCAGCAATAATTCATAATGACTATAATCGATGAATCTATCGTTGTCCCCATGGA

TGAACTGTCCGCTGTGAAACAAGACGGTCGTTTAATTATCGATCCCGTGTCTGTTGACAGGAAATCCGGTGCGGTCAAAG

TGA

>contig_16027

GTTACTTTTAAACAAATTTTATATTTTTTAATATACCTATATTTCCAATTTATCGACATTCCTTTATAAATTTAAAACAT

CTCTTAAAACATTAAACATAGCCAAATTGTAAACAGTCTTATAGCATATATATATTATACATATTTCTACATAGCTAATT

GCTTAGCTTATTGAAGAAAATATTTTCCCAACTATCGTTATCCTTTATTATTGTATATTTGGTTGGATGGCTTATATGGT

TTGCTAAAAATTATAATTGAAAACAAAAAATATGTTGTGTTATATGCATAGTATGTACATGATATTTACATTCATATTAA

GTTATTTTGATGATATTATAAATAAAGGTTAACAAATTACGTATGACTTGTGATACTCTTGTTGGGGATTGGTGTTATTT

TCACGCTCGGCAAGTAATCAGCTCTCATTATCAGCTGTGTACTGATGACGATGCTTTGTTGCGTCCCCAACGTCTCTACA

ATTGTGTACCTCAAGATCGTGGACAATACTGTTTTCATTTGGTACATAGCAAACTTTTGGCCTATGCAATTTCTTGGCCC

GGCGCTGAACGGGATGTACGCGTAAGGATGACGTCCGTTGCATTGTTCAGGCAAAAACCGGTCGGGATCGAATTTTTCCG

GATTGGGATAAATGTTTTCGTCACGGTGTAATAGATGAGGAACGACGACCATCACCGATTTAGAAGGTATTGTATACTTT

TCGAGGTGGAGCGGCTCTTTGAGGGTTCTCGTGATACCCGTCACGCTCGGATAAAGTCGCATTGTCTCTTTTATCACTCT

TTCTAAATTTGTCATTGCTTTGAGGTCTTCCATTGTTGCGTCCCGGTCGCTGTCTCCGAAAATTTCGTACAACTCATCTC

TGACCAAATTTTGTATGTTTTGATCGAGCCCCAAATGAATTATAGCCATAGTAATAGCTATTGAAGACGTGTCGTGCCCT

TCGAAAAGAAACGTATCCACTTCTTCTCGTATATCTTTATCGGTCATTGTACCGGGATTTTCTTTGGAAACGCCGACAAG

AAGGTCCAAAAATGAATATATCTGTTTCTTTCCTTTTCCATCTGTTTTTTTTTGTTCCGCGGTTTCTTCTAATATTTTTC

GTCTTTCCTTTATGACATTTTCGGTGAAATTGTGTAATAACTTTAATGATTTGCGAAATTCTCTGCCAATACTACTAAGG

TTGAAAACAATTTCGCTCCAATACCAAAATGTAAAAATACGTTTGATCAGTATGTGACTGACATTTTTGATTGCTTTAAC

GTAATCTGTTGATTTACCCTCTTGAGTACGTAAGTTTACACCCATAATTGTCTCGCACACGATGTCCAAAGCGCAGAGAG

TTACGTGGGAGTCAATATCTGCTATGGATTTTCCATTGTCTGAAGCGTTGATGAGATTTTTGACCAACGAACGAGAGTGA

TTATTGAGTGATGGCACGTAAGTTTCCAAAATTTTAAAATGAAAAGTGTTTGTTAATAGTTTTCTCCTAGAATGCCATTT

TTGATCCGTGCTGGTGAGTAACCCTTTGTTCAACCATGGTTCAAGCATCTCGTAATCGGGACCTTTCGTAATGTGCTGTA

CACTCGACAATAGTACCATGTATTTCCCAAGATTTGCAATAGTGAATTTGACTACTGTCCGACAACATATTTTTATACAT

AGAAAACCAACTCTCTATATCTACTGAAGTGATCGGTGCATATTTCATACAAGAGGTT

>contig_16409

CCAAAATGTGTTAAGTCGTAACGCTCATGCGTGTTTTACTTTTAAACTTTTAAACGATACTTCGTTAGCGCTAAAATATC

ATATTATATTGTAGCTATAACTAATAATAATTATTAAAATATGTGCTGTATATATTTGTGCGTTCACTAATTATTATTTT

ATTAACTACGAAAACCTGCCAAGACATCTTCGTACCGAGTAATAGGCATTTAAAAAACCGTCTTCATTGATAGAAAATTA

AAATTTGATAGGTCTTTCGATTACGTGATGTTCTGCTATAGTGCTGTCTGCACAAAATAAAAAACGGTCTCACGCCGATT

AAGTTTGGACGTTCGAGTTAATTTTATATTTCACCCCCCGTCAAAATGATCGTCTACAATTTGATCGGAGCTCTAACGTC

GGATTCGAATACGCTGTGGGTGGCGCTGCTCTCGCTCACGGTCCTCAGTCTGTACTTTTTGTACTTCGACAAATTGTCCA

AAATTCATGGAAAACAGATTTCGCTATTGCCATCGATCACAAAAACCCAATGGGCTTCGTTAATTGTGTCGCTGAAACTC

GCCAATTTCGGGCCAAGAGATATTTTGCCGTATTTCGACAGCGTTATCAAAAAATACGGATCGATAATCCATTTGAAAAT

CATTGCACGTCATTATGTCATCATAAACGATCCGGACGATATAAAGGTACTATTGTCGAGTGTACAG

>contig_1651

TTTCATCATAGAGATCCAGGACTGATTGGACTCATAGCATGTCCACCTAGTGTTGAGAAACCATTATTTTTTGGTATTAT

AGCTGATGGAACTCATACGCATCCGACAGCATTAAAAATTGCTCATAAAATCAACTCAGAAGGACTTGTGTTGGTAACAG

ATGCATTGTCAGCTATAGGGCTACCAGATGGAATACACTATTTGGGTGATAAACAGATTGAAGTAAAGAATTCAAAAGCA

TACATTGCTAACACAAATACATTATGTGGAAGTGTGACTCCCTTAGATGAATGTATGAGATATTTTCTAAATGCAACAAA

TTGTAGTGTTGCTGAAGCAATTGAAACAATAACTTTACATCCAGCTAAGGTCTTAGGAATACAACAATTTAAAGGTACAC

TTAATTATGGAGCTGATGCTGATTTTGTATTTTTGGGAAATAAATTGAATGTTCAATCAACTTGGATCAATGGACAATGT

GTATTTAAACATAGTAGTGGAGCTGGATATATTGAGAAAAAAAACAACTTAAATATTTCTTAGTTGACATTTTATTAGTT

TTAAATTCAATATAATAATTTAATAGTCAATAATATTACATAATGTACATATTATGAAACTTAAATTATTGAACATAAAG

ACACTAATGGAATAGCCCCATTAATTAATTGAACAATATAATTAAATAAAACTTAGAAGTACATTTTTTCTATTTACTGT

CTTGCTTCTGGATTTGATGCTCCAGCAATCATCATAAATATGACTAATGGTAATATATACATCCAATATTTTCCAAGCAT

TGATTTGTTATTATTTGATTTTCCTTTTTCTCTAGCATCACGCTCACGCTCCATCTTCTGAATATAAGTTGCGGTATCAG

GTACTGGACTGGTTTCTGGTCTTCTGAAAAATACTGTAGTTGTAAAGTTATTTGTGTTCAGTTCATCAAAGCTATTGCAG

TTGCTAGATGTAGTAGCTAAATTAACTGCAAATGGAGAGTCTAAGTGATCCAAATGTAATGTTAAAATATCATTTAATTT

AGATCCAACTATAAGACATGCTCTAGTAAAAGTGAGAAAATGTGTTTCTTTGCCGGATAAAGTTCTAACAGTGACTTTCA

ATCTGTATAGTTCATTTTTAAATGCTAATTGTTTTAGTAACTGTATATCTGAGTCATTAATAGTAGAAATAGATACAACA

TCGTTCCTGGACTCCAAATTGACAAGAATAGAACTGCGTTTGGTGAAACCAGCATTGGGAATCAGTTCATGTTCTAAATA

AACATCAGATATTGAATCATAACTTGAATCAGAACAGTAAATACTACTAAATATTGAACAAAATAAGCAAATTAGCTTAT

AATTCATCGTGTTGAGGATTCTTTTTAATAAATCAGTGGGCTCTATACAGTAATTAATATTACTGGTGATGGATCTAAAA

GAATTATAGTGTCACTCTAGCATCAATCATGAACGAAATATTTAACATTTTTAGTGGGTTTTCATATTAAAATTCCTGTA

TTTCAATATCTTTTTAGTTTTTACCATTATCAAAGCGTAGATAATGATTTTTGTTTACATCGTAACCATTTTGATAGCGG

ATCAAAAAGTAAAATCAATTGTAA

>contig_16521

AACTTTTGGACAGTGATATGGTACCTGCTATTATTGAATTTTATTTGGATCCAACTTCATTGGTAAAGAAGACATTTTTA

AAACTGCTAATAAACCATTGGGTTTCTGAAATTTGTGAAATACAAAATCTTTTGGACAAAATCATCGATCCCTTTGCATT

TACTCAAACAACAATAGAAACATCTATGAATATTATTGATACTATCTCTAAGAGTAATGCAAATACCAACATGGATTTAT

ATGTCAAGTTTTCAAATGGAATGATTAATTTTTCAAAATTTATGGAAAAAATATTTACTACAGCCCTTTTTGAAAATAAA

GAACTTTCTACAACTTTTGAGCAGTTTAAAAATGCTCTGAGAGAGTACAATGCATGTTTAGTCTATAATCAAAGAACAAA

TGACAGTTATAATGATGAATCAATTAAAAATAAATTACTTAAAAGATGTACAATTGTAATAAAATATTTGAAGAATACTC

AAATTTTAATTTCTAATATACTAAATGATAAAAATTTGTCGGAATTAGAAAATACAAATTATATTTTATCTAAAACAAAA

CAATCCTCCCCGAGTATGTTACAAACTGATAACTCTGATAATAATGAAGATTTGGATTTACTGCTAACAACTATTCAAGT

TCAAAAATCATTTTACAATAAATCAGAACGTAAAACTTCAGAAAAAATGTTTTATCAAGATTATGAATGTTTAATATCCG

CTAATGAAACAACCTATGAGAATGTTGTAACAATGGATCTTACAAAAATTCTTGATAATTTTATTCTCGAGTCATTCAAT

ATAAATAAGGATAATAAATTTACAGTTAATTTATGGGAAGAAATAGGTATTGACAATCCTACAAGGATACAAGATCTAGA

TAATGTTGATAATAAGATTACAAGTGTTAATGAATTATATTAAAGCGCTTATTTCTTTTTCTAATAAAATTGTTACAAAT

ATGCTTTAAATAAACATCCTAATTAGTTGTTTGATTTAAAATTTAAATGATAGTTTTATATTTTACATCAAATGTAAAAG

CTATTGTAAATATTAAGAATGGCG

>contig_1678

AGTTAATCTCTTGCGCATCCATCACTAACGCGTCTACACGTACCACACCCCAAAAAGAAAACCCGCTACTGCTGACGATT

AGGGGATTACCGGAAGCGGGTATTTCGATCGTCTCAGAGGCTTGGTTTGTTTTTCGGGAGTTTCCAAATACGATTGCTTT

AACGACGCGTCCGTCCCTTTCCGGTCACGAGTGACAAAGCCGACATATTTCTACACCGAGGTTTCCAAAACTAATGTAAG

ATTCAGAAATGGCGGGTACCGATACCCGATGGCTGGTGATCCTATGTTTACTATCTTCAGCGCAATGTGTACCTTCCAAG

GTTACGTCTCAATCGGAAACAGATTCCGAAGATTCAAGAAGAGTTCTTGGTTCAAGTGTGGTCACATCTGTATCTGTAAT

AATGGATCATGGAAATGGTACCAAAACATATTTTGCTGATGGACAAAGTAAAAACGTACATAAACCTACAGATACCATAA

TCGGTAGTCCTGGTAATTTACTATCACCGGACCGTTATGAGTTTTATACTTTTGACGAAACGGGAGATTTAGTTAAAAGA

TTAATGACATTGGATGAAATACATGGTTTAATTGCTGGAGCAGATCCAGAATCAGTGATGGCTGATGCTTCTCCTACATA

TTATCATGATGCACTTGCAAGTCATTCATCAGAAGCACTTATGCATTTACCCGCTTCAGATCTCGAATCACTAGATGCAC

CAGCAGTGCACAAGGTTTTGGAAAGTGTACAAAATGTACTGAAAAATGAAATGGCAGCTAATTCAGGTAAACCTATTCCT

TCTATGCCTAGCACATCTTTACATCGCCCAGATTCAGCTTCCGCGTGGTCAGCCTTGTTCCCAGGCCTAATTGAAACTCC

AGATGACGTAGCTCATCTTTCTGGATACTTACTTCCGTCCAAAACAACTGAGTCAATGGGCGCACCATTAACTAAAAATC

CATTGTCACCTACTAAGGCACCGTTAGTATCAATGCAACCTCAATTAACAACACTTAAATTGCCATCGTCATCTATAAGA

ACGCAATTGACCACTACAACAGAAAAAATAAGGTCAACCCCAAAATACTCTACTGTCAAACATGAAGTAACAACGAAATT

TACTCCAACACAGTCAACAACTCAATGGACAACACTTAAACCATCAACGACTAAAAAACCTTTTAAGCCAATTCAGTCAT

TTGAGAAGCAAAAGCCAGCACTACAAAAACCAATTTTAATGATGCATTCAACTGCTAAACCACCTATTGTTTCAGATAGT

TTTGAAATAAATAAAGAAATGTCTGCTTCTATATCAGATATGTTGTCACAAGTAACTGACAGCCAATCGTCCAATCATAA

ACCTTTACAAACTAACGATTTAGTAGAAAAAGATAGTACATCAGATTATGTACCAGAAGAGAGTTCTCCAGAATTAAGTT

ATTATAGTCCAATAGCAATGTCATTGAGCACTAGCGGTGCATTGAAACCAGTTACAGAAGCTTTAACATCAGCTAGAAAA

CCTCCAACAACAATGTCATTTAAAACTACAACTACAGATGTACCATTTTCACAAAAGACCGCTCCTACTATTATGTCAAC

AAGATTTTCGCCACCCAAGTCTAATGCCACTAAGCCTCGACCACATGCAAATGCTACTACAGCCTCTTATATGACAAAAC

CATCATCGATTAATAAATATACTGCAACTACTCCTCCGACTACCAGGAATAACTACCATTATCCACCAAGCAACATGTCA

ACTAAATATATGCAAAAAATTACACAAACCACTCAAGCTTACCCAAACAAATTAATTGACAGTTTCGTAAAAGTTGTTAA

CAAAACGATAAATAATGCAACTCTTAGTGTAAAACAACCAAATGATACATTTTGGAATTCTACCGAGGAATCAAAGAATA

AATTTGTCAAAGAGCCTGTTAAAGTTTTTCAGACTAAAGTCACTTCATCTAGACCAAATGGCATTAATCGAACGAATGTC

ACTTTGTCAAATCGCATACAAGAATTAAAAACTACGGCTATTCCAAACTTCGGATTTGGTTCATCTACCACTACGGTTCG

TAGTCAAACAGAACCTGATAAATATGTATCAGTGAAAGTGGAAGAATGGCCAATTACTGTTCAACATCCAACTGCATACC

CTTCGACTACTCCATATATGAGTACTGAAAAATCGTTCTTTTCCAGCAATAGTCAGTCACTGAACTCTGTCACGGATGCT

AACGTAATTAGAGATTTAATATCATCTTTTATGACTAAAACGTCAACAGCTTCCAGTATTTCCACTAGTACGCTATCTCC

AACTACCGTTAGTGCTCAACAAACGTTGACTGCGGCCATGTCGACTACTACACCATCAGCAGTAAATACTATTACTGGAA

TTCCAGCATCAATGATATCATCCACAGTTGGAGATTTAGTGACACCATCAACAACAACTACAACCACAACAGAACCAACT

ACAAAAATATCGATCAAAACAGAAGCTGTGGAAAATATGACAATGGAAACCACAACGAAATCAGCGTCAACTACAAAGGA

AATAGAAGCGGTAAAAACTGCTGTGGAAGCTGCAGATATAGGTATAATTGCAGTGACGACAACTGCCGCTTCAACAAGTG

CTGCAAATACGATAGCAGTCGATACAACGTCCGCTCAGGTAACAACTAGCACACCGATAACACAGACGACGTCCAGTACA

GCAACAGGTTTTATATCATCCACTACAGCAGCTGAAACCACGGTTGCCAATGCGACCAAGGTTACATTTACAACACCGGA

ATCGGAGGCGCAATACGTACCCGTGACACATTTTTTACCTGAATTACAACTGAAAGACAGAACAGAAAATGAAAATAACA

ACAGTGCGCCGGGCGTAGATCTGATGCTCAAAGACGGTATAAATGCAGTAACTAACATGTTGCAAAACACTCAAAAGCCA

GCTGTGGCCACGGTTCTTGAAACTGAGTACCAGGAAATTGGCAAGCACCCTTACGAAAAATTATCTTCGGTTGAGGGTAA

ACAATCATCTACCGAACAAAGTAGCCGAAAACAAGCGACGGTTAAGTCAGACTTTGACGAAACCACCACATTTTCCATAT

CCGAGAACACTGCGTCACAGGAAATCTACGACTTTACAACCATTGCAACGGAATCGGCTACCAAGTTTCCAGAACAGAAC

GACACAGTCGCTGCGGAATCGGTGAACACCAATGACACAGAACAAGTTTTAAGAAATTCAGAACTAGGCTTGGAAATGTT

GACTGTATTCAATGTTAGTTCCACTGATGAACCTGGACAAGTGGCCAAGTTGGCCGACTTGTCATCGGAAACGCAAGAGT

TATACGATACCACTACGCCTTTTGAAAATCGAGCCGCTTCTACAAATATAGCAACCAATACTGAAGAACCAGTAACTACA

ACGTTTGCCCTTTATGTCCCTCCGGTGACGAAGGAAGTGACAACGTCAACCGTAACCGTGCCGTCTACATCAACAACCAC

GGTAAAACTACCATCACCAATACCATTAGTGAAAGCAGCGACAGTAACACCAGAATCAGTAGCAACAGTACCAGAAGCAT

CAGTAGCGACAGCAACAGCATCAACAACAGCATCAGCAACAGTATCAGTCACGGCTTCAGAATCTGAAATCCAAAATCCA

ACGACAACAATAGTCGAAAACTCTTCGGTGGCCGCGACAAACTACACCACTTTATCCACGCCAGCTTTAGTCACGTTGCC

ACCTGCGGTCAAATCTCCTGGACCTTCATCTAATGCGTACCGACCACCAATGGCCAATAGTGCATCTCCGTCTACAGTCG

AGCTGCATCCTGCGCCGCATGAGAGCATGGGTATGGAAGCGAGTGTAGCGTTTTTGGGTGATGACGTTCGACGATTTGCT

GACCTTTGTAACGAGCTATCGTTCAAAATGTGGACAGCTGTTACCGGAAAAGGTCAGATAGCGTCCAGAAGTCTGGTATT

GTCTCCCTTCGAGCTAACTGCTATGCTGGCCATGGTATTCTTAGGTGCCCGTGGTTCCACTTCTGGTCAAATGAACGACG

TACTAAGGCTTGATGATATGGTGACATTTAATCCTCACCAAGTGCTTCGGAATATAACTCACTCGATAACGAATGTCAAC

AATCCAGGTGTGGCTACCGCATCTTTTGTCAGAGAGATATACAGCCACAAGGGAAATGGAAAAATCCTAGAATTCTATAA

AGAACGAGTACAGCAGTATTATGATGGTCACGTAGAAGAAGTTGACTTTAATACGATTGGTGACGTTTTACGGAGAAGAA

CGAATCTATTAGTCAAAAGGCAAACTTTGGGTCGTGTTGTGGAATATCTTCGAGGATCTGGATTAAATTTGACACCACCA

TTCGCTGCATTCAGCGCTAACGTATTCCAGACTAGCTGCGAGAGCGCGTCAACCGAAGGCCGGGACGGTGAAATGTACTT

CGTGGTGAGACCGTCAACCAGACAGCGTCGCCTGGTCCCGGTTCCGGCGGCCGTGTGGCGTAGCGGTTTCTTGGCCGGTT

ACGAACCCGGACTGGACGCGACAGCCGTTTGCCTGGGCCCAGACTCGGCGGTGTCCACCATACTGGTGTTGCCAGGCCAA

CAGGGTCAGGTGGCTCCGGGCGACGGACTTGCACGGCTGGAACAGCGACTCATCGAGACGTCTTACCGACGGGGCGGTTG

GTCTCGGGTGCTACGGAGCCTGTTGCCCCGGCCCGGACTCGAGCTGCAAGTGCCACGTTTCTCGCACCGATCCGTGTTGA

ACGCAACGGCAGCTCTGCAAAAGATGGGTCTCCGGGACGTGTTCAGCGATCAGAAGGCGGACTTACGTGGCGTCAATGGA

CTGTACGACCTGTACCTTTCCGACATGCTGCAAGTGAACACGTTCAGTACGTGTGGCGAGGACACGATCGGCGCGCGGCA

TCACGTGGAAACGTATCCTGCTTCACCGCAGCGGATGGGCCGCTCCGGTCGTCACGAAAGCGGTCAAAACGGCGGTGACG

ACGATAGCCGCACAAGTGATGATGAGGAGACGGTTCGCAGTGGTGGCCCGGAAAGCGGTCGCCAGAAACGCTACGCCGAC

GACCTGCCGCTGCACAGTCCGTACTCGCATTTGCCGCTCAACTTGCGGCCCAGACAGGCGAGACTTCCGGACGTGCCGAG

GCTCCGGTTCGACAGGCCGTTTTTGTATCTGGTCCGACACAACCCCACGGGCATGATCATCTACTTGGGCAGATTTAACC

CGAGGCTGTTGCCGTGATCCGTTATTATATATGGAACGATTACATAATAATTATTAACGAAATCATTATTATTATTGATT

ATTATCGATATCGTACGAAAAATATAACGTAGCATCACAAATATATTGTACACACTATAACACTATTGCCAAACGATGAT

TTAAAAGTGAGTTTCTGTTTTTAGAATATCAAATAGCCCGCGAGACATGCATCCCTGTTATTGCGACAAAAAATGATTAA

ATTGATTTACACACGACTATTATTATGTTTGGCATTTGGTTTTATGGAATTAATCCGGTCGATTTCACTGACATTTAACA

ATAAAAGTATTTAAAAATGATCCATTGATATTTTTCAAATAATTTTGTGATCTTGGTAAAATCGACATCCTAAACGTATT

TTAAGTTTAATATTATATACAATAATCTTACATTTTATTTATATAGATATATTTATTTTTATATACGTATATAATTTATT

ATTATTATAACAATTTTT

>contig_17105

TGAAGTTTAAGTCAGACGTGAATGCATCTGTTTCCGAATAGTTTGTAAACGTTGCGTTATAACTTTAAACTTCAACGTTT

ATGGTTAAATTTTTTTTTTAAAAATGGTTTTTAAACTGTTATTTATTTCGATTTTCATAGTATATGCCACCGGTCAGGAT

ACTAAAACAAATTACCAATATAATGAAAACAATCAAATCAAGCTTATTTCAGAGCCTGCGCTTTACAACAAGGGTGAAGC

AAAGTGGAATTCATATGGAGGAGGTAATAGTGTTTATTATACAAATGGGCTGCCAATAAATTTCGATGCAAGAAAAAGAT

GGCCAAATTGCCCATCGATCAGTCATATTTATAATCAAGGAAACTGTCGTTCGAGTTATGCCATATCGGTAGCGTCTGCA

GTAACTGATAGAATATGTATCCATTCGAACGAGACAAAAAATCCTATAATGTCTGCACAGCAGATTATTTCTTGCTGTTA

TTTGTGCGGATATGGATGTGATGGTGGATCACAATTCGAGTCTTGGGACTTTTATAGGAGACATGGGTTTGTGTCAGGTG

GTGATTACAACTCCAACCAGGGTTGTCAACCATATATGATCCCACCATGTAAATTAATAAATGAAAAATCACCTAGACAC

AGTTGTACAACATATAACAGAGAAGAAACACCAGCATGTGAAATAAAGTGTAATAATCCAAATTATTACACTTCATTCAA

AACCGATATTTATAAAGGTAAATATTATCAAGTTTATCCTTTTATGGCTATGAAAGAAATTTTTGATAATGGTCCGATAA

CAACTCAGTTCTACATGTATAGAGATTTAATCGATTACAAATCGGGTGTATATCAGTACGACGAGGATTCTTCGGATGAT

TTCTTCACTGTACAAAGTGTGAAGATTATCGGTTGGGGTGAAGAAAATGGTGTACCGTATTGGTTAGTAGCCAATTCATT

TGGTACAGATTGGGGAGATAAGGGAACGTTTAAGATTTCAAGAGGCAATGACGGATGCTTTTTTCAAGAAAAAATGTACG

CTGGATTACCATTATAATGAATTATTCATAATACCATAAAAATTATTATAAATAAGCATCATTCAAGTGTTTGTTATAAG

TATCTACTTTGAATAATGTATTTAGCATGTTTGACATGTAAATAGCATGATGTTAAAATTAAAAAAACCTATCACTTCAA

AGACCATGGCTTTCATTTTTTTTAAGTACCACTGAAAGCTGTTATTATATTTTTATATACCTAACTATTTGTATTATTTT

TAAATGATCCTCTAGATATACAAACACTTTTAATACAGTTATTTAAAAGCTATTTTAACCAAATAACAGGATTGTTGTAC

AATATATCAAAAGTATAATATTTAATACCATTGCGGTAAAATAATTAAGTTAAGTTTAAATATATGATAACTAAACAAGC

ATATTTAATATATTATTATATATTGATTGATTAATAACTATACAATACTACAATACAAATAAATTGCAATATAT

>contig_17526

CTTTCGAAATGGATACATTAAAGCTAACGATGTCGTGTTGCCTGTGTACTTCAAGAAGTTTGGACAGCGTATACAGGACA

TGGAAATCAGGGATGATGACGTTTGGGTCTGCAGCTATCCAAAAACAGGAACCACGTGGTGTCAGGAGATGACGTGGTGC

ATTGCAAACGATTTGGATTTTGAAGGTGCCAAACAATTTTTGCCAGAAAGATTCCCATTTTTAGATCACACACCATTATT

TGACTATGAAAAAGTGCTGCCGGAAAAACCAGACCTCAAGCTACCGTTGTATGTATCTGATTCGATCACGTACATAAATG

AATTAAAATCTCCGAGATTTATCAAGACTCATTTACCTTATAAACTCCTGCCAAAAAAACTTAGAGATCAAAGTACCAAA

GCAAAAATCGTGTATGTGGCCAGAAACCCAAAAGACACTTGCCTATCATATTTTCACCATTGTTGTTTGTTAGAAGGATA

TACTGGCAATTTTGAAGATTTTTGTAAATTGTTTACCTCTGATTCCCTTTGTTTCAGCCCATTTTTTGATCACATTCTTG

GCTACTGGAACCGAAAAGATGATTCGCAAGTCCTTTTTTTGAAGTATGAAGATATGAAACAGGATCTACGTACAGTAATT

CGTCAAACCGCACAGTTTTTAGGTAAAGATTTGGCTGACGACCAGGTGTTGGTTTTGGAAGACCACCTAAGTTTCGAAAG

CATGAAAAACAACAGAGCTGTCAATTATGAGCCAGTTATTGAGATTAATAAAACCCACAATTTAATTGACGCTAATGGTA

CTTTTATGAGAAGTGGTACAGTGGGAGGAGGAAAACAAAAAATGTCACCAGAGTTTATAAACATTTTTGACGAATGGGAA

GAAAAATGCTTAGGGAAAAGTGGATTAAAGTTTTAATGCAATTACTTAAGTATAATACCTCTATTATGATTCTATTTTTT

ATAGCTAGTGTTTATATAATACAAAAAAGTATGATGGTGTTAGGTATTTAATGAGAACGTAAATACAAGACTAATGATTT

ATTTTATATTATATTATTGCACCCACAGGGTTAATCATTCCAATTAACCAGTTAAATTTTATATACGTTTTTCTTATGGA

ATAATTATTGTTTGCAGGATAGCTGAATCATTTTTCAAAGTTCATTAAATCATTAATTTATATTAATTTATAACATTTAT

TCAATAAAATATTTATATAATTTTAGTCTAATATGGAAAAATAATTAAGTGACGTTTAATGTAAACATTAATCAACTATA

GACATTTTATTTTCTCAATCGTAACACATTTCAACTATATGTATAATGTGTTTTATTTTTATTAAAA

>contig_1801

CTGAATATAACATTTTAACTATAAAAAAAGTCAAAATAATTATTTTTTACCTGCATTTTAAATTTATTAAAAATGATTCA

ATACGTTTAATTATAACAAAGTTAAAGTAAAATATACGATTATGAAAGTAATATTTTTTTATAATAGCATAATAATACAA

GTATGTGTCAGATTGAATCTGGCTATAATGGAAAACGAAATTTGTTTTGTATATTAAAATCGAATAGGTAGCTGGATAAT

ACTGATTTAATCGTCACATTTGCAAGATTGATCCATTAACAATTGTTGTATACATTCTTCTATATCCTGAAAACTGTCTT

TTGTGGAATATCGTTTAACAGGACATCCGTTTCTGTCTATTATAAATTTTGTGAAATTCCATTTTATACTTTTTGATTTG

AAAGCGCCTGGTAACTTATACTTGAGAAATCTGTAAAACGGATGTGTGTCTTTGCCATTTACGCAAATTTGCGAAAACAC

TTCGAATTCAGGATGACTTCGTGCCAATATTTCCGCTGCAGTATTCCCACCGATATTCTGGAAGAAGTCATTGCTAGGAA

ACATCAGTATCGTTAAGCCTTGTCTTCTGTACTTTTTAGACAGCTCGGAAAGATTGCACACGTTGTCGTAGGTGAAACCA

CACGTGGACGCGTAATTCACAATGATCAATACTTGTCCAGCATATTTTCTCAAACAAACTTCCTGTCCATCGAGATTTTG

TACGGTGTAATCGTAAACATTTCCATATGTGTAATCGTATTCATCGGAGTTTGAGTTTTCAAACTCAAACTTGTCATATT

TGAATGGTGTCGATTCAGAAATGTCCATCATAGACCGCGAGGTCTGCCAGTCAAAAGGGTCGTATGCTTGGGACATGTCA

GCAGAAATCGGCGCCGTCCAAAATGAAACAGCAAAAATTAAAATAATATACTCTTGAGTGTTCATTATTTTCATTTTCAA

ATTAATGATGATGATAACAGTATTGACAGTATTTT

>contig_18013

TTGATATTTATAATCAGAGTTAATAATTGTTTGAATTGATAACATTTGTAAACATCTAATTGTTTCTAATGATGCAGATT

GATCACATCCCAATAATGCAGTCATATTATGATATGTTTGTTTAGGTACAGGATCTACTGCAAATGCTGACAGTGGGGTA

CCACTCAATGCGAAAATTCCACTTATCATTCCTTTTGTAAACTCGGACATTGTTAGCAATGTTGCCGCACTAGCTCCACT

ACCTTGACCAGATACAACTATTCGTTCTGGATCTCCATTAAAATAACTAATATATTCTTTAACCCATTTAACAGCAGCAC

GCATGTCAAACAATCCTACATTGCCAGGAAGATTTTTTAAATTACTTGTAAGCCAACCTAATGATCCAAGTCTGTATTGT

ATAGTAACTACAGTTATGTTATTTTTCACTAAATGAGCAGGTCCATATTGTAGAGCTGAACCACTAAAGAATCCACCTCC

GTGTATCCAAATCAATACTGGGTAATTTGCATCTTTGGGTTTTTTCAATGGAGTAAATACATTGAGAAATAAACAATCTT

CACTTCCAACTAGTGCACTGCCATCAAAACTTAACTGTGGGCAAGGAGGTCCAAATTTTGTGGCATCAACTTCTCCAGAA

AGACGAACAAGTGATGGACGCT

>contig_1893

TTATGTGGCTTTTGAATATTCTAAAAGGAAAGAAAAGATCTTAAATTTATTTGTTTGATGCATGCGAAACCATAAAACTA

CGAGAACCAATTTGGTTAAAATCATTCTTCAGTAATCATGAAAACCCTAATAAAGTTATTCCTACTAGCACTAGCAGTAG

GATTAGCTTTATCAGGAAGTTCGAATCCTTTAAAAACTATTATTAAAGAAGATTCTGAAGAAAGTGAAGTATTATGTTCC

ACAGAATGTACCGATGGAATTTCAAATAACAATATACTGTTTGATGAGAACAGAAAATATGGGTATTTGTTCAGCGGTAA

ATCCGCTATTATTGCTCCGACCGGCACTGAAGAAGGTATATCAGAAGTATTGATTAAAGGAAGAGCCACTGTCATCGGGA

CACAAAAATGTGGTGCCGTCCTTTATCTTAAGCAAATTGAAATAACTCAAGGATCGAATACTTATGGCCGAGAAGTCTTA

CGGGAATTGGAAGGATATCCAGTTCTGTTTTCTTACAATAATGGAAAAGTTGGTCGGAATATTTGTTCGATACCACAGGA

TTCTACCGCATCGTTGAATTTAAAACGAGCGATTATATCAGCATTGCAAGTGTCGTCGATACAAGAAAATCAGAAAGAAT

TTTTTGAGACTGATATTCAAGGGACGTGTAATACCACATATTATCCTTATTGGACATTGTCGGACTCGCAATTCACAGTA

ACGAAGGATAAAGATCTTACACAATGCACGGGTTACGACAAAATCAATTTATTGGGATTGTCATCAACACAATCGTATCC

GATTTATGGAGATTCTCCGTTCTCATCAGCCGTTCAGCATTCTGAAATCAAAATAAAACAGTCCACATTAATATCAGTCA

CCAACGAAGAGACTTTCCTCTATCAACCGTTTAGTGCAATTGACGTCGTAACTAAAATAATAGTTTCTATGACTATGCAA

TTAACGAAAATGTCAACGATTCAACCCCTTGTATTAACCCGTCTGTCCAAAAGCCGATCTATTGTGTTTGAAAAACCAAC

TGCAACAATTGGATTGGCAGATGCGAATTCGTTAGTTACTGCAACTCAGTTGGCTTTGGATAAACTTGAGCCAATAGTCG

ATGTGAATGGTGCTAATAGTTTTTCAAATTTAGTGCAATTATTAAAAGTAGCAAAGAAGGACGACATACTTTCTGCGTTC

AACATGATAATGAGTGGTAGCACAGGATTTAAGGATACCAAAGTTGCGGAGAAAATTTTCATTGATGCTCTAATGGCTGC

TAATAGTGGTGAATCCATTGAAGCCGTTGTAGAACTGATAGATAGTCATCAATTAAGCGATTCATTAACTATTTCATGGT

TTAATAACTTGGCAAACGCAAAACATCCCACCAAAGAAGCCGTGGTCAAAGCATCAAGTTTGCTCGATGGCAGAGCTCTA

AAAGAGAGCTATCTTGGAGTTGGCGCTTTATTAAGACAATACCTACAAGACACTCAAGATTTTTATAGTACTGAAGTAGC

AAATACTTTAGATAATTTAGGAAAACCGTTAATGAAAGCGGGCCAAGGTCCATTATCAGCTGAAGACGAAGATTTAATCA

TTGCTAGTCTAAGAAGTATCGGCTATGCGCAATACATGAATAGTGATCTAGAAGATTTAATTATTCAAATAACTATGGAT

AAAAATACTATTCAAAGGATTAAAGCAGCATCATTAGCCATGCTTAAAATCTATGCTAAAAATGCAAAGGTGGAGAAAGC

TTGTGAACATATTTTTACCGATACTTTGGCAGATTCTGAACTACGTATTTTAGCATTCAAAGTATTTGCCATTAATCCAA

CCAACTCTAAAGCATCTGTTATAAAAGATGTATTAGATGACAAAACTACCCAACTACAAATTCGAGCTTTCTTATTCAGC

TATTTGAGTAACTTACAGACGACTGCAGATGAATATAAACTAGACCAAAAAAGATTTTATGAAGGAATTATGATTAAGTC

GTCAAAAAGACCACTCAATGACATTCTACGTTACTCACAAAATTTAGAGTATTACCATCAATGTCCTCTACTACATAGTG

GATTTTTGTTGGACGGAGAAGTCATTTATTCATATGATACTTTTTTGGCACGGTCTTCTTCGGCATCACTTAAAGCGAAT

ATATTTGGACGTCAATATAATATTTTTGATATTGATGTTCGTGTTGAAAATTTGGAACCATTACTGGAAAATCTATTTGG

ACCTGAAGGATATCTTGCAAAAAGTAAAGGATCTACTTTCGATAATGTTAAAAATTATGTAACTCAGGCAATAAACTATG

CGAAGAATAGAGAACTGAATGGTATAGATTCTGAATCTTATTCCAAACAGACTTGTGAAAATATTCCTGAAGAAGAAATG

AAAAAAATGTATATTGATGTAGCACTTAAATTATTCGGAAATGATCTCGGTTGGTTTACATTCCAAGGACTTGAATGTCA

AATTACTTATCAACATATAATTGATGCTTTACTTAAGCAGTTTGATAATTCTGTAACTGACAAAAAGAACATTGAACTGA

ATATAAGGAAACAAATATCAATCATCGATTCCGAAACTAAATTCGCAACATGTTCTGGGCTTCCAGTTTCCCTAAAAATC

CAGGCTACAACAGCTATTAAATTTGATATAGCTACTAAGATTGATTTAGAAAATTATTTTAAACATCCAGAAAATTTAAA

TTTCATACTCAAACTTGTTCCTAGTGGTTCACTTGATCTTAACGCTATGTTAATGATCGATGGATATATTGTTGATGCCG

GATATAAAATATCGTCGTCACTCCATACTTCCACAGGATTTGATTTCTCGTTTGAGACGGTTGAAGAAAAAGGTTTCGAA

ATAGCTTATGGACTTCCATTGGACGTTATAGATATACTGACTATAAAATCTGGAGCTTATTCAATAGTACAAGAAAAAGG

AAGTCAATCGATTGAAACACCTCTTTTGTCTACAGATATTGAAACACAATCATATACAAGAACATTTGATCAATTTGAGG

ATATTATAGGATTATCATTTTTCATTAATTATAAATGTCCTTGGAACGGAGAGTTAAAATCTTTACTGCCATTCTCTGGT

CCATCATCATTTTCTTTAAACATTTTTAAAGTTGATGAAAAATTAACTAAATATATTGTAAAAGGAAATCACGACTTTAA

GTCACCAACGCTAAAATATATCAAGTGTATATTTGTTACACCGGGTGCAGCCACTAAACGTGGTTTGGAGTTACTCTTTG

TTTATGATACTAATGAACATAATAAATTAGTAGCAGAGTTTAGAACTCCATTCACCAGAAGAATTCTGCAAGGTACATTA

GTTGATTCCGATGAACAAAAAACCATAGAAATTAAATATATTGATGGTTCCCAGCACATTTTGAGTACTGGTGTTAAATG

TGAACAGGTGGACTCAGATACAATGAAATATTCACCAATATTTGAAGCTAAGTACTCTACAGAAGTTAACGATAAAACGC

ACAAACATCATCTCTCATCTCTGTTTGACGTAGAGGGTTACGTTCTCGTGCAACGTAATATGAATCCTAAAAGCCATTAT

CCAACTAAATTAACTTTAAGAGACATTGCTGTTGTTACTACAAAGAGCAGACATTCTTTACAAGGGTCGTTCACTTTTGA

AAACAATGAAGTATTAGGCGATGTTAGTTTAACTGCTAATAGTCTTGCTGTAAAAATGAACGGACTTCTTAGTGGAGATT

ATCCGATTTTTAGATTGGATTTAAATTTAGATATGACTAGAAATGAACAATCCGATCAACAATTGGAAACTGATGAAAGC

TATGATAATAGACCAAATTCTAAATTTGTTGCTTTACTATACAAAGTTAAGACTTTGAATTTACTTACATCCCACGAACT

TCATATTAAATCACCATATGTATACATAAGTAATAATCATATCCGCTTGGACGATGATTATTTAGAAGCAAACTGTGACG

TACTAATTGAGAACAGCGAACCCACAATACACGGAAATATTTCAGCTTCTAATTTTCTTGATGTTGCATTTAACGCTAAT

ATCACTGTTCTGCCGAAAACTGAAGACACAGGACTATTTGTTCGTGGATTTTTAGAAAAGTCTCTTGTTTCGGACTCATC

AAATGTTGGATATAGAGATTTTAAATATGTTCAAAATAAAAATAAAAAAAGTAGTCATCTTGAATTTACTTCAGAATACC

CGCAAACATTTGGAAAATATGAAATTAGTACAGAACAACAGTTTACAGACGACGCTTTCATGTATACTCTTCTTTATGAT

ACCCACGATGGTTCCACGTTTACGGTAGATTTATATATATTGACAAACGATATTACTTGTAATATTAATTTTGATTCTCC

CAATCCTGAAAATAAGTCTTATATGCTCATACATGGTTGGTTAGAAACAAGAAACACATATGCATTCGATACTAAAGTCT

TATGGCCCGGTGATCAAAATTTAAATTATATGAATGCAAATGGAAAAATGCTTTTGAACGGACGTCAAATCCACCTGAGT

GGAAACGTTGATTTTCCATTAGCACATTTAGTAAATGTTCATTTATCAATAAAAAGTAAACCTGATCCCGAAATTGAAGA

TGGTGGATTTGCTACAGTTCGATGCACATCTAATAATCAAGTGTTACTATCTGAAAAGTTCAAGTATACATTAACACTAA

AAGATGATGAATTTTATGTGTCTGGAGAGAGTATTTACGACGAATCACAAAATGTCAAACCTATTAATTTTGAAATATTA

TACACAAATTACATAGATGAACTACACTTTACTGGCAAACAAATCGAGTACAAAATTGAACTAGAACAAAATAATCAGAA

AATTGTACACCAAAAACGTTTGGAAGTTACTAAACACGTGTTCAACTATAGACAAATACACAAAATAGATAATAAAGAAC

AAAACATAATGATGGATTTAAGTTATAATATAACAAGTGCAACAGATTGGTCTTATAAATTAATATCTAAAGTGAATTCC

CCGAGTATTTTTAATTATTTGGTTAATAGTGACGAATTAGAATTTAATTCTTATGTTACATTTGGTAATTATTTGTTGGA

TCAAGAGCATATTCTTAAAATGTATTCAACACAAATTTTAACTGACTTGTATAGACTTAAATGGTTTACAAACTGTGAAA

AAACAGCATTCAAATTGTTATATAATGAACGAGAAATAAGAGGTTTAGCGTATTATAGCTTAGATCAAATTAAAGGAGGT

TACAATTTGAAATATGCGGGTCGATTATGGTTAGATTTTAAAAAAGATCCAAATTCTGAAAGCACCTTGTCAATAAATAA

ATACTATACAATTCAAGATGGACTTTCTGCAGGCACTGACATAAAAATATGTGTGCCATCATTTAATGATAAGGAAATGG

GTATAACAACCGATGTCAAGATGTTTAAGAGCTGGGAAAATCCTTTATATATACTATTAGAACTTGATATTTTCCCCACT

ACAGAACAAACATTAAAACTCCATACTAGTTTGAATAGAGATATTCAAGATAATGGAGTTTTGTATTCTGCTTCGTTAAT

GGCTGAAAGCGAAGGAATGAAATTGGATTATGGAATCAAAAATGCTATTTTTATTTCAAGTGATAAATGGGCAAAATATT

TAGATATACGTTTTATTGACTCAGATAACACTATTAAACCCATTCAATTACATTTTGAATTAGTGCCTAAAGGATTCACT

AACAAAATTAACATTTTAGATATTGCCGTGGTTGATGTTGATTCGACTATCACGGTAAAAGACGGAGTTTTAAATACGCG

TTCGAACATACGTTACAATGAGCGACCTTATTCCTTTGAATTGTCGGTTAGCTCAAATCCAAATTTCAAATTTATATTGG

AATGGAATGATAAAATGGGAAATCATGAAGAACTGATGGTCGAAGGTTTTATTGTTATGGATGAATCGGCCGTTATTAGT

GCTACTCGTATTCGAAACAATGGCAAAGACGTCATGCCTGTGGGATACTTAAAAAAAATCTTAGATGAAAAACATTGGTG

GGAAACAAATAACCATTTAAGTTCAGAACAATCTAATGCAATTTGGCAATCATTAAAGGGACAATTCATTGAATTATTTA

TAAGCGTACACAGTAACATAAAAAATATAGTTGATGTTGCCAAACAGCAGCGTAGAATACGTTGGCATGAAGCAAGAAAA

GCCCAACCACTGCAATATCGTGCATTGATAAATTATTCGCGTGATGAGGCTAAACAAATATTTAAAGAAGTCTTGGAAGA

TACTTATATACGTCAAGTACCAATGTTCATACAAAATACCACAGAAGCTATATTTGCTGATGTTATTGAATGTATAGATC

ATAATAGTCACTATTTCGTAAACTCGATTAGATATCTAAATGAAAAAATGGGTATTTATTTAAAGAAAATGTATAAGTAT

TATTCATATCAAGCAGATATATTCAGAAATAAATTGGTGCATGATATTGATGAATTAATGGAACAAGTGAAGCTTACTTT

ACAGGACATATTAAGACAGTTAAATCTATATACTGATGAACCAGTGAATGACATGCAATCGTCAGAACCTCGTTCGGTTA

AAGAGTATTATGATAATGTTATGGATAAATTTATGAAAGCAATTAAGGAAATTGAAAAAGCAATAAATAACATTAAAGAC

TTAACAGAAGATTATATCAAGAGCTTGTCGATTTATGACCTGTTACATGATCAAATTGAAAAATTAAAATCATATCCTTT

CAAAGAAAGGTTATGGGAAGAAATACACAACTATTTGAAAGAACTAGAAATGTCAGCTCCAACTACAGATTTTGCTAGTT

TTGTCAATGCTTTGGATAAGTACATAAATAATGTCATTCATGATGTACCGGTGAGCCAGAGAGAAATAATAGCCTTAAGG

AATAAAGGATTTATATTGGTGAAGTCAATGATGTTTAATTTCTCCAATTACGTGGATCACGACAGAGCAATGGCTATTTA

TAATGCATTACGTAAACTTTTTAACTTTGGCCAGACTGTTCTTCACATAGCCTCACCAAATACATTCCAATATCCAACCG

ATGAAAATCCCTTGTTATTTGAGCTCAATGAATATAATATGCAGTTATATAATAGGCATGTGAATCCATCGAATTTAATA

CCACCGTATGATGCTCAAAGTGTTATTATCGACTCCGTCTATATAATAACATACAATAGTCAAGCCTACAGCTTTATGCC

ATACACTGGATGTTATACATTGGCTGAGGATTTTGTAAACAACAAATTTTCAATACTTGCCTATTATAAAAATAACGTTT

TAACAAAGTTGTCGGTTTCAACCTATAATGGTGAAAAATACGTGTTATTTTCTAGTGGAAGGGTTACTGTAAATGACAGA

GATGTTGATTTTCCACTTATAAATGACAATTTAAAAATCTGGAAGGATGTTTTTTACTTTGGAATTGACATTTCTGTTGG

TGTCAGTATTAAATGCACTTTGGATTTCAATATTATTCAAGTGTTTATTAATGGATACCATTATGGTCATGTGTTTGGTT

TACTAGGATCTATGTATCAAGAACCTAGATATGATTTCAAACTTTCTAACGGAGAATTATCCGAAGATATGGTATCGTTT

TTGTTGGCGTACAGAAAAATAGGAAACACCGAACCAACCAATACCGATTTAATACAAACCGACCAACCACTATGTTCCAG

CTTGTTTTCTGGAAAATCTACTTTAAGACAATTTTTCCAAACTATTTCACAAACAGCATATAGAACGATTTGTAATCAAA

TCGTTTCTTCAGCTACATCTGAACAAGACAGTTTGGATAAGGCTTGTTTAGTGGCAAAAGCTTTCGTGAGCATGGCTAGA

CAAAACTTTATGTCAAATTGTAATATTCCAGATATGTGCATAATGACTTCAGTACACGAAAGAACAATAGACGCGACGAC

GAATGTCCAAATATCTGAACCAAACGACGTAGCAGATATAATGATTTTGTTTGAAGAAACCATAGAAATTGAAGAAACAT

TTTCGAAAATATTAAATCCTTTCATAAAACAAGTTGAGAGCAATTTTAAAAGAAAAGGAATAAATGACGTCAAATTTATT

CTTATTGGATACTCTGGAAAATCTACAGATTCTGAGGTGCATATGTATACAACTAATGAGGATGATGGCATTACAAAAAT

ATTGAGTAACATGCCGGAATGGATAGGATCTCATATCGCTACTACTGACGATGACGCTGGAACAAGTAGACTCCAATCTC

AGTTAATTCATTCGTTTAAAACAGTTACTGGTCAAAATTCAAAAGACAAAGCGTACAAACTTTCGGCAGATTACCCGTAC

AGAGCGAACGCTGATAAAGTTGTCTTGTCCGTCGCACAATCATTATACGATACCCAAAGCTCTGTTATAGGCGTATCTCA

ATATACGTTCAAATACGTAACGTCATGGTACACGAAACAAAGTATTTCCTTTTTCCTGATTGCCCCCATCAATTTGAATG

ATGTTTCAGATAATGGCATATTCGGAGCAAGTGGTGAGAACACCATTTATACAATTTCATCGCCGGACGGTAAATACAGC

GACAACGGATATACTTATAACATATCACTAGAGACGAATTTGGTGCTTATGACAAGTGGGACCATGTACGATTCTCAGTT

CTTAACCCACACCACAAATTCACAAGAAAAAATTTTCTTACAATCGTTATGTGAGACAATAGTAGGTATGTCCGTTACTG

ATTCAGTGGTCGAGAGAAGTTGTTCCAGTTCATTGTACAACGGAGTGATGCCATACGCAAGATGCGTTGTCGTCCAACCT

GATCTATAAAAAACAGAGTTATAGATACACATTAACACGTGTTTTGGTCAAATGAAATATTTCAATTAACAATAATATTT

ATTATTTTTTAAATATTAAATCAAGAATACAAAAAAAATGTTTAAATCTATTATAATTATCATTAAATTAGTATAATATT

GTCATAGTACCTAGTTATTATTGTTATGTAGGTATGTTAATTATTACATATTATTATGAAAAAAATAAAATAAAATATTT

TATGGCATTGAAAATTAGTAAAGATTTATTCATGAACATGAACCATATCTGTTGTCTGAATACTTAGAAAAAATTAGATT

ATTTTTTGTTTTAATTCAAAAATGAACAATCGTAGATATTATAAAATAT

>contig_19

TTTTAAGAGTTGAATCCAAACTTTTAGACGAAGAAAAAGAAGTCGCTCGTGATTCATATAATGAACTATCAAATACATAA

TTGCTATCAATTTTATGCGTCTGATCAAATCTGACTAAGACATTTGGGTTAAATCCACAACGCAAGATTTCTTGGGTTAT

CTTATTCATTATGCTCTGACTTGGGATTTGATCTCTTGTATAACATAGGATTATAGGTGTTGTATCTGTATTATAGAAAG

TGTTCTTGAACTGGTTTGCAATGCATACAATTAAGTATTCACCTGGGGAGTATCCTACAATACTTAAAAAGATTTGGTAT

TTGTAAAAGTCATAAGGTCGTTTGCAAATCATTCCATCCAATGGAAAATCAAACAAATCATCATCTTGGTTTGGATAATA

TCCTTCATAAAACAGCGTAAAAACGGTTGAACTAAATTCATGATCTGCAGGATAAACGGACATTAAATAACCTCTAGTTT

CTTCAATTGAACCGGTCCTCATGTTCATACCACGTTCCACGAACATATAAGGAGTATTGACATTCCAATCATCCATACAT

GAATCATCAATCGTAATTTGCAATGAGCATGCAGGGAAACCAGAATTTATCATTCCAATTCTTGCATCGGGACTATCGCC

TAACGAGACTGGTGTACATTGGGATAAGTAATATGTTCCACTCAAAGCGTCTCTAACAGTTCCAAAATCCAGGCCCTTAT

AGATTAGGCCACCGTTGTATCTCTTGTATAAATCACAGGGTCCAATATCATAAGGACTAGTGAAATCTGGGCTTATGCTA

ATTATAACACTCGATGTCTGCACTGTAGTTGTTGAAGTTGTTGAAGAACTGCAACTGTTACCAACATTAACAGATTTTGT

TACTGATTGTGTAGTTGTGGAAGTTTGTTGTATCGCGCTATCGATATCACCAGATAC

>contig_19037

AGGCGACACCAGTGCGATTCAACGAACGAGGCATACTATCTTGTGCAACTCTGCGCTATTCACAATTTTTTCAGTGCGAT

AAATTAAACATTAAGTGACTTGTAAATCGTTTACGAGTTGTGTGCTATTACCTTATACGTTTTAAAGTTAAATAATTTTA

TAGTTTCTATTCATATTTATTGTGTGTGAACAAAATCTACAAGTTTTCGGTGAGAAAATGGTTGGAAAGACGGCGATCGG

TATCGACTTGGGTACCACTTATTCTTGTGTGGGCATCTGGCAACACGGAAAAGTTGAGGTCATCGCCAACGATCAAGGTA

ACAGGACCACCCCGAGTTATGTGGCATTTACAGACACCGAACGGTTGATCGGCGATGCGGCTAAAAACCAAGTGGCAATG

AACCCCGTTAATACAGTGTTCGATGCCAAGCGTCTGATCGGACGTCGTTTCGACGAC

>contig_19911

ATTCATCATTTTTACAACAGGCTACAGAAAATAGTCCAACCTGAATTAATTCGTCGGTATATTTATCCAAATCACAAATC

AAACTTGATAAATCTGATGAATTTCTTAGTTGTATGTCAACAGATTCACCACACTTTCTAACCAGTTTTTTAATTATATA

AAATGGATCATTAAAAATCTTAGAAATAAGATGCAAAACAGTTGTATTAATCCGTCTTTCAAGAATAGATAATGAAGAAG

ATAAATAATCAAATTGGAAAAAACTATCAGTATCATTGATATTAACTTTTTCAATTTGTTGAATCGCTTTTAAAACATGT

TTAGATGAAGATGTTATTTCTAAGTTATCTATTTCATCTGCAACTTTAGCAATAGTTATTGCTTGAAATAAAATTTTATT

TACATTTGGTTTAATAAGTTCAAAATTATCCCTATTGTTTAATGAATTTAAAACAATATTCATTTGATCAATGAATTTTC

CTGGATTTTCATCTAAATCAGGTGTATCTATCATTTCCAAACAGTCTTCCAACTTATAAAAACATTTTTGAATCCGCTTG

ATTATGTACTTGCTTAAAATCTTTTCAAAATGTAGAATAGTAGTTTGTTCTGATATTGATATTTCATATAGAAGGAGACA

TTTTCGAATTTGACTTAAACATACACAAAGCATGTAAACATTTTGATTTTTAAATGTTTTTAAAAATACTTGAATAGATT

TTACAAAACAAAGGAGTGGTCCATTAATTAAATCTTTGAATATTAGTTTAAAAGATGATTCATAAAATTCTTTTTTCTCA

ATATTATATAAATATCTAGAACACTCATTTAAAACTATATCAACTTTG

>contig_21800

CACACTGACCATAAAAGAAACATTTGAATATTATGGAACAATTTATAACATGGATAAAAAGAACATCGATACTAAAATAG

ATGAGCTCAATACATTTCTACAGTTACCGAATTTGAATAGTTTGATTAAAGATATAAGTGGTGGCCAAAGTAGAAGAGTA

TCTCTTGGTGTTTGTCTATTGCATAATCCTAAGCTTATTATACTGGATGAACCTACAGTTGGAATTGATCCTTTATTAAG

ACAAGAAATTTGGAATGGACTTACAACGATGGTTGAAAAACATCGGAAAACAATCATAATAACTACTCATTATATTGAAG

AAGCCAACTTAGCTAATTGCATTGGTTTAATGAGAAATGGAGTGCTAGTAGAAGAAGGATCTCCTCAAGAAATTATTTTT

AAATATGAAGCAGATTCGTTAGAATCGGCTTTTTTAACGCTATGCTCTCATCAAGATTCGAATTTAGCACCAAAA

>contig_22140

GGTAAATCTTGAAAAGCTGAACGGCGAAGGAACTATTCTTCTCCCGAATTTATTCGTTAACTGTACGTACGACATCGACG

GTCGTTTAATGGTTGTTCCACTCCAAGGTCAAGGAATATTTAGAGGGAATATAACTAACACAAAAGCTGACGTCAAAGCA

AGTCTTGAAGTACTAAAAGACAAAAAGAATCGTGAATACTTCCAAGTTAAAGATATTCGAATCAAACTCAAAGTTGGAGA

CGCAAATGGCAAAATAATACCCCAAAACATCAACAAAAACAATGATGTCTTAACGGAAACTGCATCAGCGTTTTATCACC

AAAACCGTCGAGTCGTTTTGGATATTATCACTCCAATCGCCGAAGAGATCACTGTCGAATTCGCATTGCAAATTGCCAAT

AATATATTGAAAACTATACTTTACGATGAAATTCTACCTAAAGAACTACCTTGATGAGTGCATTATGTAACAATCGAATT

TAGTTTTTAAATTTAATATTTTTCTTAATTTATGTTCAATCATTCATTAATCATATTCATACATCTCAGTGTACTATATT

CATAAGTCATCTATTATTAAATACATAAATCTATGATCACG

>contig_2264

CAATAAAATTAAAATTTTTTTTAATAAGTGATGTTTAATGTTTATAAATAGTGCCACACGTGATAATTACAAGAAAAAAA

TATACAAACTGTGCAGTAAATACAAACCACTACGATGGTGCCATATCGTTGCTGTTGCGGCGGTGGGTTGATTTCGACAT

TGATGGCGGCGGTGCTCGTGAGCGCGGCTGCAGGGACGATGAACGTCGAATACAGCTGGGTGTACGTGGACTACACATTC

GCCAGCCCCAATCACCGGGAACTAGCCATAAATTCCGGGAAGTTCATTCCGGAAAACTGTGTCATACTGGACGTCGACAA

ATTTCATGGTGCATCTGAAGGAAAATTATACTTTAATATGGGTTCGAATTCAAATTCTGCTTCCAAACAACGAGTGTTCG

TAACTATTCCTCGTATCAAACCGGGAAATCCTGCATCGATCGCAGAAATAGTTCCAGGGGATCGCCCTAACTCAGTACTA

TTAGCCCCCTACCCAAATTGGAAGGCTAATACTATTTCGGAAGACACGGTCAATTGCGACGATACGATTATTTCCGTATT

TAGAATGAAAATTGACCATCTCGGTCGATTTTGGATTGTTGATGTCGGTACATTGGACCAATTTGAGATGACAGCTCGCT

CCATTTGCCCCCCAAAACTTTTAATATTTGACTTGAAAAATGACGATAGGGTTATAAGAACATATAAATTTCCATCATCA

CAGGTTAAAGACGTTTCGTTGTTTACTAACATCGAAGTCGATGTCAGAGATTCAAAAGGCCGTAATACATTTGCTTATAT

AGCTGATACTACTGCGTATAAACTTGTCGTGTACGATTTTAAAAATGACGAAAGCTGGGTGGTAGATCAAGCATATTTCT

ATCCGTATCCAAATAAAGCTCATTTTAAAATCAAAGGAGTTAACTTTGACTTAATGGATGGAGTATTAGGGTTAGCCTTA

GGTCCTATAACAAAAAACGATCGAAAATTATACTTCCATGCATTCGCTAGCATTAGGGAATCGTGGGTGTATACCAATAC

GTTACAAAATAAATCGCTATTTCAAAATGGCTTAATTGACGGTTCGGGTACATTTTTTTTATCCTCGGAAGTCAGAGACA

CCCAATCTAGTGTTGAAGTTATGACAGACAGTGGAGTACTTATATACGCATCAATGGATAACAGTTTAGGTTGCTGGAAC

AGCCAGGACCCATTTACAACAAAACACACCCACACGATATATAAGAGTGACGAAGATTTTCAATTTCCCAGTGGTATGAA

AATTGTGGGTGACAAAGTGTGGGCAGTGTCATGTCAACTTCAAAATCATTTCACAACTATGGTCACAAATCGTAAATCGG

TCAAGTACAGGGTGCTGGTTGGTCGAGTTGACGATTTGATAAAACGCACGGGATGCGATAAGAGGTCTGGAAATGTCGAT

ATGAAATCGCCACCCGATGTACAAAATCTTGACCCCACAAAAGATCGCTTAGTATTTAGTGGTTAAAAGAAGTAAATACG

GTAAAGGGATGCTTGCTTAAATGTTTATTAAAATTAAATTAAAAAAATTAATGTGTAATATATATTAGTTGACGTAGTTT

TACACTTTAATTTGTGATAACTGTGAAAAATTGGTACACGATTACATGCATATTTGCACAAAATTATTTAACCTTGACAC

AATTATGTATTTAAAGGAATCACATTTTTTTTAGTATAATTTATATAATAACTAACAAACCTTATAATATTTCAAGGACT

AACAAATTATATGAACTATTATAGTATTTATTATTTATAATCAATATATTGTATATATTATATATAAATATTTAATTTAT

TTAAAATATTTTTCTGTAAATATAATATGTTTGTATTGCAGCAGTTAATGTGTATTTTTGTAAAAAAAAAAAAAA

>contig_22703

AATTCCGGAGTGAAGTGTATTGTCATATGTTGGTATGCCCTCTGGTTCTTCGTCACTAGCTCCATCATAGTGTAAAATTG

ATACTTCAAAGGCCTTGGTGAACCTTTCATCGCAGTCCATTAATCCTCTAAATCGCATCCAATAATTCCCAACATTAGCT

GTTGCTTCCACTACAAAATCCCAACGTTCACCAGCATAACTTACAAATGAATCGACTACTACTGGTTGAACATTATATCC

ATCTGTTGCAATCATAGTAAACGTATGATTATCGATACTCATTGAAATTGGACAATTTAAGAATCCAGCGTTAATCAGTC

TAAACCTATATCGTTTACCTCTGGTCACGTTGAACTGAGCC

>contig_2364

CAGCCGCCGTTGACCGACGACGCGGCGCCGAGAGCGGCCGCGGTCGGCGAATCGTCCGCGTCCGCCGAGGAGTACGACGC

GGCCGCGGACGACGCTTCGCCGGCCGGCACCAGTCCGGTGGGCCACGTCCTCCAGCGGTCCGTCAAGTATCCCGAGTACG

TGCGGTCGTCCAGCTACCGCCGAGGGTGGCCGGGAGGCAACGTCACCCGCCCCGAAGATGTGTTCCGGTTTTGGGACGCG

TACGAGTGGACGGCCACGCCGGCCGTATCGCCGCTATGCGTCGACCAAATGCAACTCTACCAGGCGGCCCTGCGCAACGG

CAAGATGTGGGCGTTCAAAATGGCAGATTCTAGTGGACATTACAGCAGTGGTTATTTCTGGGGAAATACGTTCTGGTTAG

GATCAAAAAGTTTGTGTACAGAAATATCAGAATATGATGAAGAAGTACCTTTCAAACTAGGATTTAATATAATAAAAACA

TACATCGCCTTAAAAGCACCTATTAATTATACGGAGAGGTATCAGCACTTGGGATTGTGTATGCCGTACTCGTGTTCCAA

AGAAGACGTAGACGTGATGGTCAATCAAACGCTACATCAAGACATCCCGGAAAGAAACGTCAAAGTTGCCAAAGTGAAAT

CACCTCACGATTACTATGACTTATTTGGTGATAGAGTTTTCTGGCTCCTCGTGATAATATCAGCCACGACTGCGGTATTC

CTGATAATCGGAACAAGCTTGGACGTGTACTTAGAAAAAAACAGCCGTAATAAAATGAACGGGTTCATGTTCGACAATTA

TAGATACGCTGTTAGCAGTGCCAAGTTACAGCCGCTTTCCGAACATTCCAAAATCGATTTGGAGTCTGAAAGTTCAAAGA

GTTCCGAGACGTTGTCTAAAGAGCCACCAATGACTTGTACAGCAGTGAACATGTTGCTGGCGTTTTCAGTGCGAAGAAAC

TTAAGACAAATATGCGATAAGTCTATTGGTGAAGACACGATATCCACGGTACACGGTCTTCGATCATTGAGCATGGTTTG

GATCGTTTTGGGTCACGTGTGCATCGTTTCATTTAAATACTCGGACAACATGGAATTCCGTACCACGGCCGAACGGCATA

TGCTATTTCAATTGATCAACAAAGCAACCTTTTCAGTAGACACTTTCTTCTTCATCAGCGGACTATTAGTTTCATTCCTT

TACTTCAGGACCACGGCAAAAGTAGACGTTAACAAACTGACGAAAACCACGGGATTTTTATCTAATTTTATCGAGTTCAT

TGGACTGCTGATTTACCGATTCTGCAGACTTACTGCACCGTATTTCTTTGCTTTGGGCGTCGTACAGCTGACAATGAAAT

GGTTCCATTATAATTCCGTATTCGACCCACCGACAGACGACCACTTAAATTGCCCAAAATATTGGTGGAGAAATCTGTTG

TACATCAATACCCTGTTTCCCGTTCAGGACATGTGCATGCTTTGGAGTTGGTACTTGGCAGACGACACACAATTCTACAT

ATTGGGAGTTATTCTTCTCATACTATCTGTTCGGTACTTCAAAGCAGCCGCAGTT

>contig_237

CTTTTTTTAGAAACAAAATATAAATATATTTATTAATCATAAAATATACCTCAATCAATCAAATATCAATAATATATTTA

AAAATGTTGTTCTCTGAAAAAATATTATTTTATAGTTTAATAACAGTAATGAAATAAGAATAACATTCGAGAAAAGTATT

ACAAAATTAATTTATATGAATTAATTAAGTGAATAAATCATTTAGTTATATATATACCTCTACAATCGTACACTAATATA

ACATTTGGAAAATTTTACAGCGTAAGAATTGTTCTCATTAAAATTTATATATTCTTTAAAATCCAATCAGTGTATGAAGC

CACATTGGTATACACGGCCGGGGAAACTCCACAACGTTTGCCAAAAGAAACAATACCCATCAAATAAAATTGTTTTTCTT

TAAGCCACATTAACGGACCTCCGGAATCACCCTGACATGTGTCTTTTCCTTTTTCTCCAGCGCATATTACTCTGTCATCG

ATTGAAAACTTTAGGCCTTCATATAACTTTTTACACTCCGTCATGTTTATAATTGGGATTTGAACCTCCTTTAAAGATAA

AGACGTTGGGCCACTCGATTCAGTAGTTCCCCATCCGGCAACAAACGGCAAACTATCTCCCATATCGATATTTTTCATAC

CTGGCGATAGTGGCAAACATATTGGTTGGATAAATATGTTAAAATTAACTTTATTTTTCAATACTATTAATCCAATGTCG

TTGGCCAAACGTGGTGTGTTGTATTCTTCATGTTGTATAACACGTTCGATTGGAACATCTAATGGGGTTGCTCCATCATA

AATTGTGGGATCCAAATTCATTTCCCCTAAGCGTGCAACTGCCAATTCCATTCTCTCAGGGTTGTTAAAGCATGAAGAGC

CAGTTAAAACGTGTGTGTTAGAAATTAATGTACCACTACATAGCCATTCGATAGAACTATTGATATTATTTACATCTTTG

TATCCGAGAGCTACCATCCAAGGCCAAGCACCTAATTCAGAGTCACTTCCTCCCATTATTCGAAAGCTTGGTGCCTTTCT

TTTACCACACGTAGCTTGTGGTGGCAATTTAGATGCAAAACTTGATTTTTCACCAAATGTCTTGCTCGGTATGTTAGGTT

TATCCATAAACACAATATGGTCTTTAGGAAGTGCAGTTACAATTCCGACTATAAGGGTTGTGATTATAAAATATTGTTCT

TTTCCCATGTTGATAATTGAACTCAATACTAATTTATTTATTTTCTTCCGTGCACGGTCTTGATGATGAACGTATTTCTG

TTTTAATACTAACTGTTGTAAAAAATTGTTGCCAGCAGAGAAGCACTGTCTTAGCAATACGAGCCAATAACAAACTATAA

TTATAACTATACTTAATTATTATTACTAAAAGTAACGAACTACCAGCAAGTTTAACTGCGG

>contig_23830

GTTACTGCTGCACATTGCATGGCCACTACGGCGAACAATAATTTAAAAGTACGGCTTGGTGAATGGGATGTACGAGATCA

ATCAGAAAAGTATGCACATGAAGAATTCAACGTGGAGCGAAAAGAAGTACATCCACAGTATAGTCCTACTGATTTTCGAA

ATGACGTCGCATTAGTCAAAATTGATCATGACGTAACGTATAAACAGCACATTATTCCAGTGTGTTTGCCTAGCTCAGCA

GCTAAATTGGTTGGCAAGACAGCCACTGTAGCTGGTTGGGGTCGTACTCGACATGGTGTTGCAACAGTTCCTACGGTTTT

ACAAGAAGTGCAAGTTGAGGTGATACCAAATGAAAGATGCCAAAGGTGGTTCCGGGCTGCTGGTCGAAGGGAAACGATTC

ACGATGTGTTCCTGTGTGCTGGTTACAAAGAAGGTGGTCGAGACAGTTGTCAGGGCGATTCTGGCGGTCCTTTGACAACG

ATGCTGGATGGACGGAAAACTCTCATCGGATTGGTCTCGTGGGGAATCGGTTGCGGTCGTGAACATTTACCTGGAGTGTA

CACCAATGTACAGCGTTTCGTACCATGGATCGACAAAGTAATGGCGTGATAATAATATACGGGTTTATAATATTATATTA

TGCAAACATTGAGCTATTAAATTTAAATCTATTTAATGTTATTATAAATTATTCATATTTATGAGTTGTTGTAACCAGGT

ACAATTTACCATTGAGTTAATTTATTTATTTATAAAATTAAACTTATAACTAAATATAGTATTTATGTAATAAAATGTAT

ATAATTTGCAAGCATATTGCAATACACTTAGAAATATAAAAAAATCTAGTAATAGTTATTTATCATTATAATTTTGTTAT

TTTCTCGATGTCGAATTGTGCTATTCTTTAATTTAAACATTTCTATT

>contig_2400

TCAAAATCATTGGAAGACAAGTAGTTATAGTCTTGCATTGCACCTCTTATACTATACCATTTAGCACCATTAGTAATTCC

ACCTTCTTTTCCAAAATTTGTGTCACCACCTCTACACGGCATTCTATTTGGATTGGCCATATCTGCATGATAATTTGCAT

AGGATAAAGCTAACCATTTAAAAGTATCGTCGTCAGGACTTGTAGCGTATTCGCCTTGCTCATTACCATATCTACTGGCG

TCGTATGGATAATTGGCAACTAAATCGCCTCCGTGAAGATTAGCCGAAGCCACGAACGGTATAGACATAATCATTTGCAT

TACAGCCTTGGTTTCTGGTTGCACCGGTTGTGACAAATGATCCACCATTTGCATCAAATGATTGTTAATATCTTTGTAAT

AT

>contig_2428

GAACAATCATCGCCATTACGTCTTTTAAAGAAATTAATTAATTCAAACGATGCTTCACAATTTGACGGAAATCGTAGATC

AATTCATGATATGATCTCTAGCTCTTCAAATAAAAAACCTCTAAGTGACCTTGACAATAGGAATAATAGTAACGATCAAA

TTGATACGACATTAAATGATCCAAGCATTCAAAATAAAAAAAATAGAATGAATTTTGAAGACCTAAACAAGAGACCTCTA

AGCAGTCTAAACAGTAGAAACATGCAAAGTCTAAACTTTCCAAGTATAGGAAAAACAATAATCACTCCTTCAGTTTGGCG

ATTAGGAAGTACACGACAAAATTCTTTGTTTCCTTCTTTGAATGAAGATGATAGTCAAGGAATCAGTGATGGTAATGAGA

ACTCTAATATAATGAATATGATGAACTTTAACAAAAACATATTTAATAATCCTGGACATAAAATTGTTGGTATTTCATTA

ACACGATGTTGTATTAATAATAAATGCCGTGTGTTAAAAGAAGGAGAAAAATGTGATATTGTAGATCTTTTGAAAGATAT

GAAAAATATAAAACAGCAAAATGGAAACGATAACAGTATCATGACAAATTTAATGGGAGAAAACTTAACGCCTTTAAAGC

TAATGTTGTTCAATAGGTTAAACACTTCTTCTTTTGGTTCAAGTAAGGGTATGCCGTATATGGATTCAGAAAAAAAAAAA

CCTAATTATCCGTACTCAGAGCCTATTGGTGTAAATAAAGATGAAGCTAATAAAATTCAAAATGAAGTCCTTCAAAAATC

TAATGTTTATAGAAAAAAATTTGGTCTTGTGCCATTTACTTTGGATGATCAGTTGAAAAATTGCGCTCAAGACTGGGCTA

ATTATATGGCGGAAAATAATTTTTTTGATCATAGAGAGCACAACTTTTATGGAGAAAATCTGTACGGAAGACAAAGTTTG

AACAATTTAGGCGAAATAGCGGTCGATTCATGGTATAATGAAATGTCTCTTTTTACCAGCGCTAACAAAGAAAAAGTTGC

TACATTACACATGACACAATTATTATGGAAAGAATCAACTAAATTGGGTGTTGGTGTTGCACTCAACTCTACAAATAACA

TGTACTACGTTGTGGCTAATTATGATCCCAAAGGAAATGTATTAGGCAATTTCGAAGAAAATTTACCTAAAATAACAAAA

AAGGACATTGAAGAAGCAAAAGTAGCAGATGAAAAACTGAAAGCGGAATCGACGAATCCTGTAGTAGAGAGTTGGTCTTC

GGGTTCATCAAATCCTTTCGGATCAGAATGGAATTATTAATAGACATTATGGGTCAGATACCAATGAGAAAGAGTTGTTG

CCAATATTCTACATACTATAATAATGAAAAAAAAATTATGTAATATCAATTTAAATGCGTTTAATTAAAATTGTAGATTT

TACAAATAATACAATTATACCTAATTTATTATTAAACAATAAAAACTGTAAGTTGAATTTTCAGGGTATTAAAGTTATTT

AAATTTTAAATAATTATTATCATTGATAACGTTATCACTAATCAGCTCCTATTTTTTTTTTTTTTTTTTTGTTACAAACA

TTAATTGTGCCTTGTGGTGTAATTTTTCGTAAGTAACTATAAACATTGATAAGTAGTTTATTTTTTATTGCTTTTTAATA

TAACTCAAATTGTATAAAATAAATGTGTATGATAATATAACCATTAAATAGAATAATAACCTTTAAGAACACTTTAAATC

AGTTGCGAGGAAGCAATGCTAAAGGGATGGCCAGCAAATTGTATATAATGGGTAATTGTATGCAGTATGCACCCAAAAGT

TTGAAGCATTGTGAAATTTTTAATGGGAATTTAAGGTTCCAGTTTAAGAGTAGTTTTATTCATTATTATATAGACTAGAA

AATTGAACTGTATACTATTTGTACCTAAATTTTTCCTAAATAATAATAAAAATATTAAAAAATAACAGATTTTATGAGTC

AATAAAGTGATCAATATTTTATCGTTAAATAAAACACTATTATAATTTTTTTTTTTTACAATTAACATTTAATAAAAAAC

>contig_24607

TTGAGGCATATATCCAACATCTTTTAAATTTTCTGCTTTTAAGTCAATTCGTCCCGAATCCAATTTCATTATTCCTATTA

AACATTGAAGAAGTGTCGATTTACCACAACCACTCGGGCCCAAAAGACCATAGATAGAACCTGACGACACATTCATGTTC

AAACCATTGAAAACATTATGTTCTCCATATCTTTTAAATGCATTTATCACTTTTACATCGTATTGGATTTCTTCGTCAAT

CATATTTTGTTTTGTTGTTCTTAAGATTATATTTTGTTTTTGAAGTAGGTAATTTCAAGAAATTATAGTAGGTAAAATAA

CGTAAATCAACATATATATATATTCACAATATATTTTTTAAACATGAAAATAAATTCAATCGAAAATTCTTGGATGTAGA

TAACTTATATCGACTACAGACTGTTTATAAATAAACTATACTTCAAACATTGGAGGTTCACGAGAGCATCTACAATGCCG

ATAATGTATGGCGAATGTAAAATAAAGTGGAATTTTTG

>contig_2473

TTGTCAGAAAATGATCCTATAAAATTTGAAAATATCTCCAACGAATTTAAAGCATTAGGATTTACAAAAGTAAAAGATTT

GGCACAAACAACCTTTGGAAAAGCTAGCTCAATAAAATTGAGTGGTGATTCTTTAAGACTGCTTTATAAAGCTTTAGAGA

TGTATGATGAACACCTTGATGATCTTCAAAAATCTGCAAAATTAGAAATGGAAAATAGAAATTTTACCAGAACTCAACAA

ATATGTGAAAAATTACTGCAGCAAAAACCTAATGATTTAAATGTTATAATTCTAATGGCTGAATCATTTTTTAAATGTGG

AAACTATGAAAAATCTATTAGTTTCTTGAAAATGGCAAACAATTTGAATCCAAATTCTTTCCAAATGTTGATTAACATTG

CATTTAATTATTGGAAACTATCTAATTATGATTTAGCAAGACATTATTTTCTAGAAGCTATTAAGAAATCACCATCTTAT

AATGATTGTTGGATTTATTACGCGGAATCGTTAATTAAAACAAATGATTTAAAAAATGCTGAATACATATACATTCAAAT

TTTAAAAATTTATCCAGATTCGTATATAATAAGAAATAAATATGCTAAATTTCTATTGTATCAAAATAGATTTAAAAAAG

CTGAAGAACAATTTAAAATTGCTCTAAAAAGTGCTCCAGAGTGTGAAGTCACTCTGAGTAATTTAGGAAATCTATACTAT

TCGGCTAATCAATATGACAAAGCAATACTTAATTTTCATAAAGCTTTAAACATAAATCCTAATCTAAAAATTACTTTGTT

TAGTTTGGGGACATTATATTTAAAATTAACTGATTACCCTAAAGCAGTTCAAGCATTTGAAAAAACCATTTACTTAGATC

CAGAAAATGCCTCTGCCCTTAATTATTTAGCGATTGCATATTGTAATCAAAATAATATGGTAATGTCAGTAGAAACATAT

AAGAAATGTTTAAAATTACTGCCTGATGATTTAGAAATTAATCTGGAATTGGCGATGATTTACTTTAATATCCTGTTAGA

CTATCAAGAAGCAGAAGTATATTTAAAAAAATGTATTGAATTACAACCTCAACGAGACGATATATACAAGTATCTGTATG

CAGTATATCGAGAGTTAGAAAAATATAAAAATGCATCAGACATTTGTATAGCATTAGGTAATTTATATTTGGATAAATCT

GATCTAGAAAATGCAAGAAATGCATTTACAACTGCCTTATATTCAACTCCTGACAATGCTGATGGCCATTGGAAACTTGG

ACTTACAATGCACAAACTGGGTCATTATGATCTGGCTTTAATTAGGTATAGAAAAGCTATCGAATTGAAACCAAACTTTG

CCCATCCTTACTGTGATTCTGCAGTAATTTATGAAAAACAGGATCTGTACGAAAAGGCTTTAGAATATTATAAAATGACC

CTTCAGCTACAACCGGACCATTTAAATGCTTTGATTAATATGTCACTACTTAAACAAAAAATAGGTCAATTTGATGATAT

TGTTGATATCTTTAACAGGATTCTTCAAATTGATGAATCAGATGCTTTTGATGTACATATGAATTTAGCAAATATTTTGC

ACAAAGAAATTGGAAATCTAAATGATGCTCTTTTTCATTATGAAAAAGCCTTGACATATGACAATACATCTGTTGACATT

TATGTACGTATGGGAAGTATATGTATCGAATTAAACATGAGTAAAAAAGCATTAAGTTACTTTCATATGGCAATTCAGCT

TGATTCACAGTGTTTAGAAGCCTTCATTAATGTAGGTTCTATACAAAAAGATAGTGATAATTTCATTGATGCTATTCATG

CTTATAAAACCATACTAAAACTAAAACCAGATTTTCCAGAAGCATTTTTTAATTTAGTAAAATGTTTACAAAAAGTTTGT

GATTGGTCAGATTATGATTCTCACATAATGAAATTAAAAGAAATACTTAGTAAACAATTGGATGGTGGTCAAATCTTGTC

CTTATTACCTCATGATACATCGTTGTTACCTTTACCATTTGAAGTACAAAAAGTAGTTGCTGAGAAGTATGCACAGCATT

GTATTGAGAAATTAAAAAAATCAATTGAAGAACCTCAACAGTTTGTTTATCCTACATCTTTAGCATCTTCCAACGAAAAT

CTAAGGATTGGTTTTGTATTGACCAACTTTAGCGAACACCCAAAAACAGCAATTTTGGAATCCTTATCCAGTTTAAAAGA

CTACCAAATAGATGTCATTTGTTATTCTCTAACCAGGTTGAATCTCTTCAGTGACTTTAAGCAATATAAAGATTTGTCAC

ACCTTAAAGTAATTGATGCTGCTAAACTAATAAATAATGATGGAATCCATATATTGGTAGACATGTGTGGTTATACAAAA

GATTCTCAAACTGAACTATTTGCTCTAAGACCGGCTCCAATTCAAGTTTCATGGCTTGGTTACCGTAATACTAGTGGTGC

ACCATTTATGGATTACTTGATTACAGATAAAGTATGTTCTCCTCCTGAATTTAAAAATTTATATACTGAAAAATTGGCTT

TTATGAATCAAACTGTTTTTGTTGGTGATCATAAACAGAAGTTTCCTAATTTACATCAACTCAGTGCTGTAGATAATACA

AATAATATTTCATATCAAAGCCTAAATGGAAATAATTCTAATGAAATAAAATCAGCTGAAACTATTTACATTGATGAAGA

ACCTGTCTTATCTTACTCTAGATCACAGCATAACTTACCAGAAAATTCTGTTGTATTTTGTAATTTCAGTAAGCTGTATA

ACATAGACCGATTTACTTTCAATTTATGGTTAACTATTTTAATTAAAGTTCCTAACTCTGTACTATGGCTTTTACATTTA

AATGATGATGCTGAAAATAACTTGAAAAAATATGCTGATAATTTTAATAATGGATGTTTTGATTCATCACGTATCATTTT

TGCTGACTTCATTCCTAAATGTGAACATTTGAAAAGAATCCAGTTGGCCGATATTTATTTGGATACATGTTTATATAATG

GACACACGGCTTCCCTGGATGCATTATGGGCAGAAGTTCCAGTAGTAACATTACCTGGAGAAACATATGCATCTCGTATA

ACAGCTTCACAATTGACTACTTCAGGGATTACAGATACTATTGCAAAAAATGATGTACAATACATTGATATTGCAATAAA

ATTGTATTCAGACCGAAAATTCTTGGAACATGTCAAAAAATGTATTTTGAAATTAAAAATGAATAGCGATTTGTTTGATA

TAAATTCCTATGCAATAAAAATGATTTCAATCTTAAAAAGTATGTGGAATAGCTATCCGTCTATACATGATCTGGACATG

TAGAAATGAATATTTATTAAAAATCTACTATTTAAAATGTTTAAAAGTTCATTTTTTTTTTTTTTTT

>contig_25003

GTCCAGGGCCTACGGCTGAACGGGTGTGATGGGCACTGTACACACTCGCGACCGTCCGCGCCCATCACACCCGTTCAGCC

GCGCGTTCCCGTAGGACGGCACCGCGTCCCACTCCATCCCTCAGACAGTAGCCGCCGCGCCGCAGTGACGGTGTTTTTCT

CCGTCGACATTAAATTATCGTTTTCACATTTCTCCCGATCCGGTGGCGATCTATTTTACTCTGTTCGTCCGTCGAGTTTG

CGTCGTCGTGCTGCAGATTGGCTGGCGAGTACTCCGTCCGACAGGCGCGCCGAACATTTCCCAATAGAGGATATCGCGAC

GTCGTTCCGGTGGACATCATGTCGGGCCCGACCCTCAAGAGACTTTCGGCGCAGCCGTTATCTGTTTTGTTGTTTGTCAC

GTTATTGTTTCACGTGACAATGACTGCAGTATCTGGTCAAGAAGACATTCCAGCAGCAGACATTGATAAAGTGATTTCGG

AAGTATTTCCATCACCACCACCTTCACAGCCACCATCGGATCAATGTATTTGCGTACCATATTATTTGTGTAGAAACAAG

ACCATCAATACAGATGGAGAAGGATTAATAGATATTAG

>contig_2545

CGATAACACGAGAGCCGAGTGAACCGACTAACCAGCGATATTGGCCGTCGCCGCCGTCGTCGTTATTACGTTTTTTTCCG

CACAAACAAACGGATATTCGAATCGCGTTACCTAATCGGACGCGCGTACGTCGACAAAAGACGAAACCGAACAACCACCT

GAGTACCTGACGACGAACACGGTGTCACAGTCGTGTCCCGATTCTCTTATCGCCCACACGTTCGCGGTCGCCGTATGAAC

TTCGTTTGATCGAACGTTGCCCCGGCACAATCGTTCCCCGACTCTCGCTGCGCGTGTGTGAAATATTATTCCTTTGTTCT

CGACTTCCGCCGATCGACGATTTCCCGTTGCAGCATAAAAACCGTGGACCGACATGACTCCGCTGTGGTGTGTACTGTTA

GTGGCTCTCGCCAATTCCGCGGCGGCTATATATCCGTCCAATTCTGACGTCATCGAACTCACCGATGACAATTTCAATCA

GGTGCTCCAGAGCGTCGAAATATGGGTGGTCGAGTTCTACGCCCCGTGGTGTGGACACTGCCAGAGACTTGTGCCCGAAT

ACTCGAAAGCCGCCAAAGCCCTGAAGGGTATCGTCAAAGTGGCCGCCATAGACGCTGATAAGTATCCGTCTTTTGCAGGC

CGTTATGGCGTACAAGGTTTCCCTACTGTCAAAATATTTGTTGACAAAAACAAACCTCAAGACTTCAATGGAGATCGTAC

AGCCGTCGGTATTACCGATGAGGTTATCAAAGCCATAAAGAACTCCATTAGTGCTAACCTTCAAGGTGTACCTTATGGAT

CATCTAAATCTTCTAAAAAATCATCATCTGGTGATGACGTCGTTGAACTGACTGATTCAAATTTCGATAAACTTGTATTG

AATTCTGATGATATTTGGTTGGTTGAATTCTTTGCTCCATGGTGTGGACATTGTAAAAATTTGGCTCCTCATTGGGCAGC

TGCTGCTTCAGAACTTAAAGGAAAAGTTAAACTTGGAGCATTAGATGCTACTGTGCACTCTTCAAAAGCTCAAGAATTCA

ACATTCGTGGATATCCCACAATTAAATTCTTCCCATCAGGTACATCCAGTTCAAGTGGAGCTGAAGAATATACTGGTGGA

AGAACGTCTTCAGACATTGTTAGCTGGGCAATGCAGAAACATCAAGAGAATGTACCACCACCAGATATTATTGAAATTGT

AAATGAAGATACATTCAAAGCTGGGTGCTCAGAGCATGCTCTATGTGTGGTCTCTGTACTACCCCATATTTTAGATTGCC

AAGCCAGCTGTCGTAATGAGTATCTGAACACATTACGCTCATTAGGAGATAAATTCAAACAAAAACTTTGGGGATGGCTG

TGGGCCGAAGCAGGCAAACAACCAGAATTGGAGTCAACACTAGAAATCGGTGGTTTTGGATATCCCGCTTTGGCTGTGCT

GAATGTTAAAAAAATGAAGTACTCAATTTTAAGAGGCTCATTTTCAGAGGATGGAATTAAGGAATTTTTAAGAGATCTGT

CTTATGGTCGTGGTACAACTGCCCCAGTCAAAGGTGCTGCATTACCAGAAATTCAAGCAACTGAACCCTGGGACGGTAAA

GATGGTGAATTACCAACTGCTGATGACATTGATTTATCTGATGTCGACTTAGATGATATTCCTAAAGAAGAATTATAAAC

ACATCTCCAGTCTCCAATTTTTTTTGTATTTTATATTTTTTTAAGACTGAACAAGTTTTGCTAATAAAATGTCTAATTGT

AACACTAACAGTCAACCTTCTGATTTAAACTTCAAAACTAACGTATTATAATTGGAAAATCTCTAGATTTAAAAAAATTT

TAGCTTATAATTTATTAATTAACTAGTTATTAAAATAATCTAAACAATATCTAGATTTTCACCTATTATTGTGTAATATT

CTTTTTTATGATATCAAAGTTCAATTTTTAAAGAATGTTTTTTTATTTAATTTTTATTATTAATTTTTAAACATCCATTG

TAATCCTTAGTTGATGAAATTATTTGATGGTATTTATTATAATTAATAATAGTAATAACTTCTAAATGGTAACTTATTGT

ATTCTATAATCCTTGAAAATGATTGTTAAATTGAATTATTCATGGCGCACACTTAAACACATTATTTAGAATAAAATGTA

TTAGTTAATAGATCAGAATTTTGTCAGTATATTGTGTTAAGTGTATTGGCCAAGTTGATACTTTGTATAAAAACAAATTT

AATGAAGCTATTAGATATGTATAATAACATGGATTTATTATGTTGTAATTTGAAAAATCAAATCCTTCAAAAAATTAAAT

AAAGACATTTATAAGGCATTAGAAATATTATTCTAT

>contig_25819

TAACGATGAATACACTTTTTGGGGATTTTATAATATTTGAATAAACCACAACAATTTATCAAAGGTACACAAAAACAATG

ATTTACTTTCGTTTAAAAAATTTACTAGCCAAACTTCCTGCAGGAGCTTCGATAACCATAAATAATATTAAGCCCATTAA

ATAACTTTTCAACGTATCATACAACCAAGCATCTATCACAGTTCGTTTTGAAAGATAATTTGGCGCTCTTAGTCGACTCT

GTGATATCATCATAATAGTGAGGTTCACCAAAAAAACGGAATAAGAAAGTCTTCCCATTGGTACAATAAATCTATTATTT

AAGAATTTTGTTAGTAGACCATAACCACTTGTGAAGTGACAAATAAACACCCATGCAAAAAATATAACCCATGTGCAATG

ATTTAAAGTTGAATATAAAGCTTGTTCTAATAGATAATAAGGTCTATCTCTTTTATAAAATATAGCTCCATAAAACTGAA

CCCACATACAAATTACGGCAATAGTGAACGTCCCAGCGTGGACAGTCATTTGTGAAAATTTTACTTTATTTTCCTTCAGT

TTATCAATTATTACACCTATTGCTATACCAGTAAAAAAAGGAAAAGCTCGCATGTAACTTGCTCTGTATGACTCATTAAA

TGTCGCGGAATATCTTGTGTTTTCTAGAAACGGAAGAAGTATTTTAATTATTCCCTCCCTTTTCGTCAAAATCGTGATTA

TAACAGGCACAGACATCGACACACCAAGCATTGCGATGATTAATCCAATCCCGAATTTAATGTTCTTTGTGTAAATGTAA

ACAATAATAACTCCAAGTATGCAAAACTGAATATCACAAGATATGTACCAACTGACTATGAGACATTCATACTTAGAATC

AATTAAATTACTTATAAACAACATATTGGTCCACCAATAGTTTTTACATATTTCGGCTTCGTCCCAGGTTTTATAAGACC

ACAGTGGTCCGTCTCCAAGGTGCGGTACGATGTGAGCTGTGATTGCCATCATTGCACAATAAGCCGGTAGTATTCTGATT

ATTCTGTAGACAATTGGTGTTATAAGTTTTACCAATATTGATTTCTTTTTCAATTTTTTAAACATTGGTACTAATGTCGT

GTATATCAAAAAACCAGTTATGAAAAAAAATGGGTCAGTTAAGTTCATACAAGTCAAATAAATATCACGTCCATGGAGGT

ATATATGTTCTATAAATTTAGGATTAATTATTGGATTTCCAACTAAGAACATAAATCTATGACCCAATAAAATTGGTAAC

ATGATAATCGCTTTCAAACCATTAAGTACATTAAATTCATTTTCTTTATTGTAGTTTAATAAAAATTTTCCAGAATGAAT

AAAAGAAAATATGTAAAAACAAGATTTTGGAACTTCTTTTTCGCCTTGATTCTTTTTTTGTTGGCAAGATAATGCTACAT

AATGATGAGCTGTTGCCGCACCACAAATTAAAAATATGGCTCCAAAAATTATATTTGTTACATAATATGCTGTATTGTGA

GGATACATGTCTTCACTGATAGTGCATAATAGCGGATTCACATTAACAACTGCTTTGAACTGTTCTGGTAAAAATATCTT

GTCTAATTCATTTTGCAATGATTTTTGAAGATCTGATGCTGAACAAGAACTTGGAATGCAAATTCCCAAGTTTAAGCTAT

TTCGTTGAACTTGATCTGGATAATCAACCCACCCGAGAATTGTTTTCCATGCATGATTGTTACCAAAATCATCTAAAT

>contig_26518

GACCTGACTGTGTGCCAGCCGACGGCGTTGCAAAGCCCATTATCGTAATCAATAGAAGTCTGCCAGGACCTTCCATACAA

GTGTGCTTGGGCGACACGGTCATGGTGGACGTGGAAAACGCCATGATGGAGGAGTCGACATCCGTCCACTGGCACGGGCA

CCACCAACGCAACTCACCGTACATGGACGGCGTGCCGTACGTGACGCAGTGTCCGGTTCCACCACACAGTTCCTTCCGGT

ACGTGTACGTGGCCGACAACGAGGGCACGCACTTCTGGCATTCGCACTCGGGCTGTCAGCGGGGCGACGGAGCGTTCGGG

TCGTTCGTGGTGCGAGCCCCGAAGTCGCGGGACGTGCACCGCGACATGTACGACGTGGACGTGCACATCATCACGGTTAC

CGACTGGCTGCACGAGCTTGGCATCCGCAAGTTCCTTGCCCACTACCACGGTTCGGGCAACAATAAGCCCGAGACCATTC

TGATAAACGGCCGCGGCCGTTACAAAGTGTTCGACGGCGGTTACCGCACGCCCCTGGCTCAGTTCAACGTGACCAGA

>contig_27288

CGGGATCGCTTTAGCAGCCCCAATGTGTAGCCGGCACTGTGTCAAGCATAGCCCTCTTGTTGAGCGTTCAGAAATTTTCT

TTGACTTCGCCAAACACACACGCACAATATGACTGCCAATATTATCAAAATTGCATGCTGTCTTGTGTGCGTTGTCGGGA

TCGCTTTAGCAGCCCCATCCACTGTTAAATTGCCAAAAGACTTTGTGCAGTGTAAAAGAAGCGACCCAAAGTTAAACGAA

TGTATAAAGAATGCTCTGAGAAAAGCCATCCCACATTTAGTAAAAGGTGTTCCTAGTTTTGGTTTGTACCCAATTGATCC

AT

>contig_278

CGACCACGGCCGAGATAACGTTTTTTTTTTTATAGGATAAAACTATTAAATTGTATTTCTTTCGCTAATCGCGGTTCACC

GTTCGTGTCGTATTTGATTCCTTGACGCAGTTAATTATTAACATTAGGCCTAGTGACACGTTTCCACTTCGTCGTTGTCG

TTTTCGTCGCCCCAGTCCGTTGAATACGTCGCGAGGCCGCTGATAAAACATTCGAAAAACCACACTACAAACCGCCAAGA

AAAACAATAGCTGTCGACCCGCTATATTGTTGATTGATTCGGACAGCCGTCCCGATACGCAATTTCTACTCAGGGGCCCG

CGCCTTTTTCAATCTACCCCTCCGTATACCACCAGCCCCCTCCATTACTTTAGACTTTTTCCGACGAAACAAAACCCGGT

AATCCGTGATGGCCAAAAAGACGTACGATTTGCTGTTCAAGTTGTTGCTGATCGGCGATTCGGGCGTTGGCAAGACCTGC

ATTCTGTTCAGGTTCTCAGACGATGCGTTCACCACGACGTTCATCTCGACCATTGGTATTGACTTCAAAATCAAAACAGT

GGAACTTCAAGGCAAAAAAATAAAACTTCAAATTTGGGATACAGCAGGACAGGAGCGATTCCATACCATAACAACATCCT

ATTATAGGGGAGCAATGGGAATTATGTTAGTATATGATATTACAAACGAAAAAAGTTTTGAAAATATAGTGAAATGGCTA

CGAAACATTAATGAGCATGCTAACGAAGATGTAGAAAAAATGATTTTAGGGAACAAATGTGATATGGATGATAAACGTGT

TGTTAGTAAATCAAGAGGTGAAGGCATTGCTCGCGAACATAACATATCTTTCTTAGAAACATCTGCTAAAGCAAATATAA

ATATCGAAAAAGCATTTACTGACCTCACATTATCAATATTGAATAAGACGCCTGGACGCGAACCTACCGATGTGCCCGAA

AAAATTAACATAGATAGGAAATCAGATAGATTGTCTAATCGCTGTTGTTGAATAGTTAAGTATTTAATACAGACAATTTT

AAATGGACACTATTATTATTGACAGAATTTGTGAAACACCAAGAGATAAACATTTTGCTAAGCTCATTGATATAAATATA

CATAAAATTGTATATTATTATGTGTTCTTTAGCACATTTTTATATTACAATAAGAACTATTAACAAGTTATATATTATTA

TTTTTTATTTAAATGTAGTCATGTGAAAAGTTTAAAAATTATTATGGGGGGTACAATCTAGCAAAATATTAAACTTAGGG

GTACGGACGAAAAAAGGTTGGGAACCACTGTTTTATTATGTCAACCAGGCCAGTACAAAATAATTTGACACGTATTATAT

TGCTGAAATTAAGTTGTTACTATAGACAGATCTAACTGAACTATGTACTAATTATTATTTATTTTTATATTAGATTCAAA

TTTATATAAGTTTGATTCTTTGAGTATTACATAACACGTAAGTCTGTTCAGTTCATACTCATTAGTTGTTATAAAGGATT

AACATACTTTAATAGGGCCGTATTACCAGTTAGGCTGTATAGGCTAAAGCTTAGGGCGGCAAATTTTAATGGTTGAAACG

AAATAGCAAATTTTACC

>contig_27856

CCTCTTTCAACAACATTTCTGAACTTTGTTTAACAAGTCTGTATTATTAAAAAAAAAAAACATAACATTTATATAATAAG

TCGTTAACAACCATCTTAAAGAAAATTGTTGTCCTTAAAATTTAATGAACTATATTTTCTTTTGGCACATTAAAATTGTT

TATGTGCAATGTTAAACATACCATTTCAGTATTGCACTGTTACCCGGCAGCGCGTTATAATTTTCAAACATTTACTTACT

TATACAATATACACATAGCTAATTTAAAACTAAAATAATACTCAATAGGAGATTATAAATATATAATTAATAAAACATTT

TCAATAACACTACTTCTAAGGTGTTAAACTTTAAAATAATAACCCGTACAAAAAATACGACGACATAATATATTATAATA

TTATTATGTATAATGTAGCTATATATATACAAAATGGCAAATATAAATGGTCAATAGTCTACATAATAGAAATTCTATGT

TAATATTTGTTTAAAGTGTGGTAACTTAAATAAATATACGAATATAAAAGTACATATTATATATTATGAGAATAAAATTA

TGCGTAATATATAATACTTAAAAATAAAACTATTTACTATAGTATTGGAAAGGTAAAGGAAATTACAAAACAAATGATAG

ACATGAAAATTTATAGTAAATTATTCGGAGTCTTTCAACCACACTTTAAGTGGGATTTGTAAAAATGCGTTGTTCAAGAA

ATTTTTGAAATGTTTTCCGATGGCTTGCGAAGCGGCGGGTTTCATTTCCTCGATAATTTCTGCTGAATTTTCATTCAAAA

ATACATTCATTGCTTCACCTATGACGTTTTGCATATTGATGGCATCTTTGATTTTAAAATCAGATTTTTCCAATTTGAAA

CCGATTCCCAACCTTTCGGTTTTCATAAATTTCACTCCTTCTTTATTCACCTCAACTCCGTATAATTTTGCTAAGGCCGT

TACGTTTTCGAAGGACGCCCAAAATTCTCCTCTACTTCTAACGGGTAACAGTAACACGTTTCCGTTGACATCGTAGTTTC

CAGTAGCTTCGATGTGTGGTATGAAAAGTCCTAAGTCTATGCGGTATTTATTCATATCACTCCTGACGTTTAAAACAGTA

TAGTTGCTCGCGCCGTGTATAGTCATGTTACTGAGTATGGCCCTCACTCTGACCGCACCATGACCGTTTTCCATTTGCAT

TTTTGGTATAACCAATGGCTCAATCGGAGGCACGGTAAGCTCTTCGATTCCATTAATGAGGTAAGGACGAAGATGGTTGA

ACGTTTCTTTAATACAACTATTGAATTGAACATCCGCTTTACGACATGAATGAATGTATTCAGGTGTGGCATTTACGGAA

AGAACGGCTTCGGTGGGCCGGGAAAAGGTGGCGATCGTCGTTATCAACAGAGTCGCTCGGAAGATGAGTGTCAGCGACAC

CGGACGATCGGCGGCGGCTGTGTACGACGGTGTCACCATCCTCCTAACTGCACGCACGTTAGGGGTTGTTAATGAATTTA

CGTGTTTTTTTTTTTTATGTAAATAGCAATTTATCGTGTTGCGCTCTGCAGTGATCGGCCAACTATACGACGCTGTCGTG

CGTAATGCGATTTTAAATGGTATATTCTTAAATCGGTTGTTTCTAAGCCGATCGTTCCGCACACGAGGATCGATGAGCCG

GCCGATTCATTGCGAACCGATGAACAGAAATAAGAGCGAATCGATTGACAAAAAAAAAAAAAAAACACTAAAAGAACACG

ATATTGGGCGTGCTGGATATATAATTTATAATAATATGTGTTTTACGGAGTTAATAAAAATAGACTGCGAAACGGCGCGT

ATAATTTGTTTGCAGGAATCAGCTCCTTTTGGCTTTTTTCCTGAGCGTCGATGGCGGCAGTGATGTATACTTCTACAGGT

GGGCGATGCGATGTCGAATAATAACTGTGTTATTATAATATCATGAGAACGGAAGACGGCGACGTGTTACAGTGCGACGT

TATGGCCGACGAGTGCGATCGGTTCGTCGACGATAGTGAAAGTATATCGCGGGGGCTCGTACGGACTGTC

>contig_2861

TTACTTGGAAATGGTATTAGCTGAAACATTACGTAAGTATCCACCATTAATAACACTATTTAGAGAAGCAACGAAGGATT

ACCAAGTGCCCGATGACACTTTAGTAATTGAAAAAGGAACAAAAGTTCTTATTCCTGCGTATGCTATTCATCACGACTAT

AGATATTATCCCGATCCAGAAACTTTCGATCCTGAACGGTTCTCACCTGAAGAAAAAGCTAAAAGACCAAATGGAACTTA

TATGCCATTTGGCGATGGACCTCGCTTATGTATAGGAAAACGATT

>contig_2862

TTTTTTTCAAGTTAATAAAATATAATATTATGTTTGATATTTACACTAATTAATATAGTAAACTAATACGGAAGTATCGT

GATTATTGTACAAACTTAAATTGTAATTTACTGTTTTTTTCAGTAATTTGTAAACAATACGTAATATATTAACAGGGGAT

GTTTAGAAAATAACCATTTCTTAACAGTCTGAGTATTCTTCAATAAGATAATAAATGTTTTGTGTGCTATGGTATAATAT

ATTTTTTGACCCACCACGTTTTCTTATTTAAAAGTTTTTAGGCTCAAGTTTTATGTACTAATTAGATATTTGTATTTATT

GGTTTAAATTTTAACCAAATGCCATTCTCTGGTGTAATAACTATAGTTTTGCTACTGAATCTCATTGGAATGTCTGTTTT

TTCACATGGTTCCACTTCATACTTGGTTAAAAGTTCAGTTAACGCTAATTTCATTTCCATTTCAGCAAATCGTTTTCCTA

TACATAAGCG

>contig_29020

CACCGAACGGTTGATCGGCGACGGGGCCAAGAACCAGGTGGCGATGAATCCGGTCAACACGGTGTTCGACGCCAAACGTT

TGATCGGACGTCGTTTCGACGACGAGAAGACGCAAGCGGACATCAAACACTGGCCGTTCAAAGTCGTTAACGACTGCGGG

AAGCCTAAAATCCAGGTGGAATTCAAAGGCGAGCGAAAGGTGTTCGCGCCCGAAGAGATAAGTTCGATGGTGCTGACGAA

AATGAAGGAGACCGCCGAAGCGTACTTGGGCCGAAAAGTGACGGACGCCGTCATCACGGTGCCGGCGTACTTCAACGACT

CGCAGAGACAAGCGACCAAGGACGCGGGAGTCATAGCCGGCCTGAACGTGATGCGGATCATCAACGAGCCGACGGCCGCG

GCTCTGGCGTACGGTCTGGACAAGAACCTGAAGGGAGAGAAGAACGTGTTGATATTTGACCTGGGCGGCGGCACGTTCGA

CGTGTCCGTTCTGCA

>contig_29301

AGTTAGTCGAGTCATATAATTGAATGTATGCATATATATTTAAAAACTGTTTATAAATTTGCCATAAATCTTAAATAAAC

CATAAGCATACATATCTTAGTTAAAAACAGAATAATACATAAATAGTAAGCAAACTGTACATAAATAGGTATAAAAATAA

AATCGTATGTAAGAGTGTAAATAGTTTATGTTTTTCATGGTGTACCTAACTCACGACATTCCATAAGGGGCAACAGCGTT

GGTCTTCAGATAAAAAAGTCCTCTTGCTTTTGGATTTGCGTCATGTCCCATAGGACTTTTATTATTATTTTTACATTGAC

CACTCGTATACTCGTCCCACGAAGTACATTCTGTAGCTATAAATGTGTATCTATTGTATATGGAATCGGTGTAAAATTGA

TGAGATCTTGAATGGCTGCAACCGATGAGATTTAATAATCCGCTCGGCGTATATGATTCTAAACATCCTGGTTGAGGTGG

TTTCCCCTCGTTTGGATAAAAATCTACATGCCCTATGGGTTTTGAGTATCCAGCTGTACCCGCTGCCGTATGGATTACGT

CCACAAATGCTGCATCAGTACTGTCTAAGTAGTCTGAACGTAATGACACATACTCAAATCCAGGACCAGCCGGGTCTAAA

CCTGTAATTCTGCCAATTTTCCCAAGACTGAACGCAGCACCACATGCCCCAGACACATGCGCACCGAGACTATGGCCAAT

AACATGAATATCTGACGGATCAATCACTGCAAGGTTAACAACATTTTCCAACATTTTGGCGACTATGGAACCCACCTGAA

CAGTTAAATAGGCCGGCATTGGGTATAGTATATTTTTTGCCACTCGATTCCAGTCCGCGGTGATAATGTTGAATCCTCCA

GCATCCACGTATGCAGTTTTTATAGCAAATACTCCTTTGTCGTCTTCATCTGAACCACGCCATCCATGCGTAATAACTAT

TAAAGGTTTACCTCTAATCCAATATTCGATTATTTGTTTTTCATTGCCAAATTCAATTAATA

>contig_2952

AACTTGTTAAGTAGAATAAAAATATATTATACATACGCCGATAAAAAATAACTATTATAAAACTTTTAAATTTGATTTAT

TTTTTAATTTTACCGAAATCAGTCAGACGGAGTCACGGTTATGATATTTATTTTGTTATGATCGAGTTTGTAAACATAAT

GATACTTCGAAGAATAAATTTAACGGAAAACGATGATATTATAATGACGTATATGATTGACGTAGGTAAACAAAATTTCC

CTAAAAGCTTGTAATAAATAATAATTAAATATAGATTATTTCTTTATAATAATTAGAGGGTAATTTGTAAATGTTCTGAG

AAGAACCGCCGGCAATATTATGACGTATTAAACTCTTGGACCACAATAACTGAATATAAAAACTCATTTCTGGTCGACCC

GTTCGATACCACAACAATAATATTAATGCATAACAAGTATAGGCGGTAATCTAGGAAATAAGTACTAACCAACTGGTGGG

TCGGGCAATGGGGAAATGAGTTCAAGAGGTAGGTACTATGACGACAATCACGCGTCACTAGAAGGTCGTCGAGCATAAAT

TATAATGCTGTAATACCACACTTTGTCTTAGTTTGGCGAAGTATAAAAAAAAACAACAACACACGATTGATGCCGATGAT

TATTGCTTATAAAAGCTGATCTAAAACGTCGTCTATTCAGTATACAATTGTACGCCATGGATTCCAGAATTGCAGTAGTC

TGTGTTGTTTTGGCTGCGTTCGCAGTGGACCAAACGGTCGGAGCGCCACAAAAAGATGCCGTAGCCGCAAGCGGTCCCGC

TTACACTACCAAATACGACCACATTGATGTTGATCAAGTTCTGGCTTCCAAAAGATTAGTAAATAGCTACGTTCAATGTC

TGTTGGACAAAAAACCATGCACTCCCGAAGGAGCTGAATTGAGAAAAATTTTACCCGATGCCTTAAAAACACAATGTGCG

AAATGCAATACCACCCAAAAGAATGCTGCTCTTAAAGTTGTTGACAGACTACAGAAAGATTATGATGCTGAGTGGAAACA

GCTTCTTGACAAATGGGACCCAAAACGTGAACATTTCCAGAAATTCCAACAATTTTTAGCGGAAGAAAAGAAAAAGGGTT

TTACTAAATTTTAACTTTAAGGAAAAGTTAACCAATTAGTATAGTAGTTTTTAATAATTTAGATTTATCAATACATAAGA

ATGCAATACCTATATGCACTATTTATCAAATGTATACAAAAATAATAAAAATACCATTACTTATTTATATGTACAATTCT

TGTTTGTTTGTTTTTTTATGATCATAAAGGAAATAATCAATATTTTGATTTATGTTTTTATTATGTTATATTTATTTTGT

ATTCGTCTAAAACATAAAAATGAAGAATAATATATATTCAATTAACATACAGTTTTTGTATTTAATTTGAAATAAAAGCA

GTCATTTTAATTAAGATTAATCATTTCGTATTCTATAAAATCTTACGAGTGTTAAAAAAAATTCACAGTTCGTTGTCAAA

TCATTATCCTTATAAAGTAATAATAACATTATTACGCACTAAAATATTGTTTAAAAATGATAGTGATAAATTTAATAAGT

ACTAATAATAATTATTATTGTTTTAAAATTGAACATTCATATTGACAACTCAATATAATTCGTTAGCCTATAACAATTAT

TACTATTACCATGATCTAGACGAGATAAATTATATAGGCAATTTAACTCGTATAAGTATAATACAAAAACAAAATTTCGA

ACATGCCTTAACTTCCGTTTCAATTATGTATTAGCCGAGTACCCATACCGATCATATTATTTCACGTTAATTTCTAATTA

ATGAACCAAAAAAATTCAAGAATTATTTACATTATTATTTTTGTATAATTGTAAAGTTGTGCTTAATAATATAATATAGA

GGTGTTATACTAAAAGGATAACAAAAAAAAAAAAAAATTACTGCAGTTTTTTTTAACGGTAGAAATTCACAAGAATTATA

TAATTTCAATATAACTGTTCTATCCAAGTAAAATTACTAAAAAAAAATGTTTACATAGTATAATAATGTTTTTATGAGTA

ATGTTTTATTATGCCTATAAAAGTCTACAATCGAACTTGATTGAAAGCTTAGTCCTCATTAAAAATGATGAACTAGTCTC

TCATGACATTCCTGGGAGTACAATATTTTATGAAACAAGGATTATGTTGTACCCTGTTATGTTAATTTTACCGGTCATAC

ATAATATGATATTGTGTATCTAAAATAATTATTATTAGTCTGAACTAAATATAATATTGTAATATGTATTATACTATATT

TTGAAGAAACATACTAAATTTGTTCATTAGAAATGTGTGCGTAAAGTGCACCTTAATTCAAGTCTAGGAAATTGTGTGTT

CCGTAGTATTGTACATTTTAAATTTGTTTTAAATGTCATATTCACAATACTAAAAAAAAAAAAAAACAATAATTATCATA

AACTCATAAGATTAATTGGCACACTTAAAAGCTTCTTATATAACATAATATAAATAAAAAAAATAAAAAAATAATAATAC

AAGAATGATAGAGGTAATAATATTAATGAACTAAAATTATACAGTCTTTTTTTAGACTAGAAATGATTATGTAGTAAATT

ATATTTTACTATTACATGGTTTGTACGAAATCATATAGAACGGAAGTAGGTATGACAATTACAAAAATTAACGATTCTAT

TAAAACATTTAAATTGAGTTAATACCGTCAGTGTATCGAATTAAATTGTATTGAATACCGAGTATTGCGTATATTAGTCT

TAAATAATTATTATATGTTATTATATGCAATTTTATAGCATTAATTTTTGTAGGTAACACAATTTCATTTTAATATAAAC

TTATTTATAATGTATGACATCAATCATTATTGTTTTATATTTACACATAATAATTTATTAAATATTGCATATTTTTAGAG

AAAAGAATTTGTGTTGTTTAAGTTTAATATAAAAACAATTAAGTACTTCAATGAAAGTACCCACATGACAAACACAAGAT

AGTGATGATATAAAATTATAATAACTACTTTTTTAAGATACAAAAAATGTATTGTTGCGAATGAAATAAAATTTTTAATT

ATTATGTATAAAACATTTATTTCCTTAATAATTAAAACCTAATTTATTTTTACATCGCGGTTTTAAATACAAATTTTTCC

TTATACTACCTGTATAATATGTTAATCTACGAACATTTTATCAATTCTGGTCCCAAATACATGGGTCCAAACGCAGGCTT

GAACACCAAATACGAATTATTATCGCTGAAAAATTGTCCAAGTTTATTTTTCATATATTGCTCTGTTTGCGTTTGGTTGG

GGTTTGTTGGCCACTGTTTGGAGCATTACCGGTAGCTCCATTTTCGAGTTTGGCTAATTTTTTGTACTGCGACGAGTTAA

TAAATCTTGGATAAGAATCCCTATGCATAAGCGTGTAAATTTGTAGCTGAGCTTCATCGAACATATGAGATGATGGTTGA

TTGATGTTCTTATTGACGATATCACGAACACGAGAGTCTAAACTCACCTCTTTAGGAGATAAAATCGAAATGTAATCTTC

ATAAATGAACCGAGCTTTTTCCTCTATTACATCAGGATTCGTTTCTTTTTTTAACTCTTCAACTGCCATCCAGAACAATA

TGTTCTCTTCGCTGTATTCGCATCGTAAAAAATCCCGAAATATCTTTTTTCCCGCAGGACACTTTAGAAGTCGGTCAAAT

GATTTACTCCAGCTTCGTATTTCGTCCAACGGAGGCCTTAGCTCAACAGGATTTTCGATTATGCCGCCGCTAGACCCGTT

TCCATTTGTTTTGGGGGGATGACCTTTTAATGGGAAACAAAAACGATCCCATAGCTTGCATATCCTACTCC

>contig_3019

AAATACAATTTTTTATTTTTTATTAACTTTATAAACAACTTGTTAATAAAAAGTATGAGTATTGTGCTTTATATTGTGAA

CAAAACATGTAAAAAAAATAAACGCACTTCTCTTGATATGTTCATCATTGACCAATGATATGATTATAAAAATTTGTTCT

TTTAAAAATAAATAGTTTACAAAAATAACACAACCCCCAAAAATATCTCATTTAAAATAAATAGTATATCAAACAAAACA

AGTTTACAATTCATCGTGCTGTAGTTTGAATGACTCTAAAATGTTTAAACGAACACTCAAGTCTGCTTTCTTTTCTTGAG

TCATTGAACCCTTAAGCACGTTGTTGATGCGAATAGTTTCCGTTTGGAAAAACTCGTCACCCTTTTCCAAAGCTTTCCTC

ATACTCTTCACATACATCTCGGCTGACTTTTGCTTTTGCTTACCCTCACTGATGTCCCATAGTTTCTCAGCTCTCAGCAA

TATTTCTTTGCGTTTGTCCACTGATTTTTCTCTGGCGAATTCTGATGCTAAGCCATCAAACTTCTCTAATGTACCTGGAA

GTCCAATGTACAAGATTTCTTTGCTGTGTTTGACAACAAACTTTTGTAACTTTTCCTGATTGAAATCTTCATCGTTATAC

ACATACGGCTCTGACTGGCCGTTCACAAACAGTTTTACAACTGGGTAATCTTTTTTTGATATACCATACCTGATGGCCAA

ATCTTCATTGTCTTTATCTCCATAATCTTTCACCGGTACTTCAGCCACGAGCAAATCTTCTACCGACTGATAGGCAGAAC

CTAATTTTACGTACTCTTCGTGTTTTTCACCATATGGATACGACACGTCGAATTTGACGATTGATACTTTAAATTTTGAA

AGGACCTTGTCAAAAGTCAACGAGTCTAACGAAACTACACCTTTGGATTGTGTAGCACGACACGCGCAAAAACACAACAC

GAATACCGACAGGGTCTGTAAAAAAAGCATTTTATCAAGGCAGAACATGGATGAGCGTTCGGTTTACGAATACCAGACTT

AATACTGTAATTAGGTATGTTTCAAACGTGTGATTTATGACGAAATCACATCAGAACGTATTATCATTAATAGTAATAAT

TTATATTACAACTATTCGTATTTTGTACAATAACTGAATAACGTTCATTCAATGTCATAACTTAATTACTTGTCACGTCT

CGTATTCTTGTTTGACCACGTATAATATTAAACAATAATATTATTATCATTATTGCCATTGTTGTTACCACTCGCCGGTC

CACATCGACCGAGGTACTTTTGTGATACGAATCCTTTCGGTGATAACTGTTTAATCAACAATATTATTTTGATGGTCTGA

ACGTCGTCTGGTACCGTTTGAATTGTTGAAAACACCGCTCGACGTGCGGAATTGAACTATGAATAACTAGACGATTTAAC

ACAG

>contig_3063

TGTGGACTCCATAACCAACAATGGCTACACCATGATCCAGTGATTTGGGACTACATAATGCGTGAATTGGATGTGATACA

CCACCCATATAAAACTGCATAGCATTAGCATTTACCCCAACAGATAAGGGTCCATGTTTAACTAAAAATTTGGCAATATC

TTCTTCATCTGTAGATACATTTACAGCCTTAGATATTGACACCTTTACTTCAGGTTTTTTTAACTGGCAACCTTTTCGAT

CTGCATGACCTTCATAAGGATAATCTGACTCTGTCTCTAAGCCTCCTAAATTTTCAATGGCTTCAAACGCTTGCGTCATT

AAGCCACCTCCACATCCATTGTCCAAATTATCACAATCAATTAGTTCTTGTTCTGAGAGGGACAGAAGTGTCTTAGATTT

TAAAGCATACTGTCCTTCAATATTGGCTATGGCTGAGAATGCCCAACATGATCCACAAGCTCCCTGATTTTTTACTGGTG

TTACTACATTGTAGTCTCTCCAATCAAATTCATTAGGAATAGAGGCACTTTGTGGAATAACTGCCATAGGTAATGTTTTT

TTACTTGACAATGATGGGTTCAGACCCAAGTATTTTTTTTTAAATTCATTTTTTGTAAGATCTGCAAATTGTGTAGCTCC

ATAAATAGCACTTCCTTGTTCATGGACTTGTAATAATTTTACTTTCTTCATATTAGCAGCAAAGATTCGGAATCTTCTAT

TCTTTTCTTCAAGATTAGAATACATTTTATTATGCACCATAATAAAATTTTCAAAGTCTGTCTTTAATTGTAATCGATCA

TCATTAATATTCTTCTTTGCGGTAAATGTTTTAGAATTTGATGTATCTATGTTGGTTTTGTTTGGTTTATAACAATTGAA

TTTTAAAGTATTTTTGTTGTTCTCCCATGCTTGTTCCCATACTTTTATATTACAAATTCTACATGTCTTTTTATTAATTT

CACCTGTAGAATTCTCGCATACTTCTAATTCAATGTTATAGATAATTCCAGACACAATTTGTTTGGACACATTTAAGATT

TTTGATAAAACATGGGGTTTGTTAGATCCTTCTTCTTTATCAATAGATATTAAAGCATAATCCACTATATCTTGAACTTT

CTTATCATTAATACTCAAATTTATTCTACCTCCAAGATTTAAATTTTCATGTATGTCTCTTTTAGTCACAGCAATACTTG

AAAATGAATTAAGTTCAGTAGGTCTAGAACAAGCAACTTTAGTTACATTTTTATTGTTCAACCATGATTGTTCCCATATA

GTTACATCACATGAACGACAATTATTGTGATCCATAACAATATCCTTTCCTTTTGTTGAATCTTTTTCACACACCAATAA

TTTTATTTTATAATTTATTCCAGCTACAACCTGACTCTTTGCAGAAAGGACTCGGATTAATCCCAAGGATCTTTTAGACA

TTGTTTGTTGTGTTATAGAATCTAATGAAAAGAGAGCCAGTTGTTTGATATTCTCAGTATCAGCATCGATATCATTGTAA

CCTCCCACAGGTGATGGCT

>contig_33555

TTTTAAATGTGGTTTTATTTCACTTATAAAATTCATACACATTACTCATACAAACATTTTTCTTATTATGTACATTTCAT

AAGATATATAGTTTAAAAAATAGAAATTAAGTGCTAAATATATATTTAGATAATATATTCTAGTGTACCTAATAAAATTT

ACAAATATAAAAACGTAAAAATTGTAAAAAATAGCACATTTTCCATTTATAAAATATATAAATGCATTATACACTTTAAA

AATGTTTATAACATGTTTATATAATAAGTATCTTAAATTATCGATTATCACTAATATAAAACAATTGAAAACTGTTTTTT

TTTGTACAGGCGTTCAAGTATATTTTTATGTGACAAATCGAAAGAAACACAATAAAATTTATTTTCAAAAAAAAATCATC

TAAAACATATTTTCTATATTTACAGTGACTTTTTAATCTTTATAGATCTTGCGTTGTAATCGAATCCACAGAATCATACT

CATTGCACTTGATATTAATGTCACTGCGACCATAAACGAATACGTCATGTAAAACTTTTTTGACGAGATGCACAAAGTCC

TGTCGCCCGTCGTGAACGCCTGCAGCGGATCCAGCACGTCGGTGGCGGTCTGATTGAAATTGTCGGCCAGCAGGTCGTCG

GGCGAGTATACGTGTACGCGACCGACCAACATCATGGAATCCGCACTGTCGTCGAATGTATTGATCGTATTGTTCGTCGC

AATATTGTGCACGTCTCGTCTTTTACGACCGGGATCGATTTTTTGATTCGTGTCTGAACACGGCTGGCAGTTGAATTTGC

ACAGTTCGACGTTGCACTCGATCAACAGGTCCATGACGTCCGGAAATTTGAAAGCTTGGAAGTATGCGTAAGCTATCAAC

GATCCGGTAGCCGGGTTTGTCGTTGTCTGGAAGCTACCGAAAAGTTTGGGTTTCGCCACACATCCTTGGGAGTCGGTCAG

CTGTAGTTGATTGCCCGACTCTGGGTTGTTGTCACGGGCTACGCATTCTCTTACTAAGATGTTAAATCCGGGGTCACCAT

CCACCGTCACCACCAGTGTCATGGTCTCTCCGATTTTTACTAGACCGTTAGCGATCGGAGCGAACGGTCCTTTGCCGGCT

TGGATATCGAGTCGGGCAGTCGCCGTATCTCCGCTGAATGTCACTACTTCCTGGTTCAACATACCAATATTCAACGCGAT

AGACAGAGCTTTGTTCAAATTACCTTCCCACAGACATCTGACACTACGTATGACGTCCCATACTTCTTGAATTCCAGGTT

CGTTTTGTATGACCAATACGTTTTCCAAGTATGCCTGTTTTCCCGAAGAAAACTCATCGACGAACCGCGTCCCGCAAGAG

TTCAACATCACTTTGAACGAATACTTCGTCTGGCCGGAATTCGGGTTCACATATCTGCACTCCGGTGTATTGTAGAACCC

CTTTGAATAAACAACCCCATAGTAAGGTCTGTTAAACTCGACATTAATGGTCATCATGTCTTTCGAACACTGCACGTTCA

TCTCGTGTATATGTGGTGGGTGTGTAATATCGTTGTTGTTGAATTGGAGGTTTACTGGGTTTTCCAATTCTGGTTGCAGA

GGTTGATTAGTGGCCTGAAAACTAGTCGGCGAAATATCTACGAAGCCCTGTGTTTGTGTCGAGACGTCTGGTGACGACGA

GGGTTGTAATTCAGCTCCCGCAACTGGTGCAAGAGGCTGTATCGGTGTGTCATCTGAAGGTGGTGGGGGTGATACCGTCT

GACTGGGTTCGGCAGACTGTACGAAAAATGGATTTGCGGACGGAGTGGCAGCTGGTTGATATTGTGATGAGGTCGTCGGT

ATGTTGAACTCCGGTTGGGGTGGTGGTTGCGGAGGAAGATAACCCCTGTAGTCCGATTGAGCAGGAGACACCACGAATGG

GCGAGGACGCTGTTCGTTGTTCTGGCGAGCTAAAAAACATAATGAATTCGTGGTCAATATGATACTTCACTTATTTGATA

TAATATAGTGTAGTATAAAATAAATGAACTTTTAAGAGTAGTGTATAAA

>contig_336

AGTAATTTAAATTTAAATATCTAACCTTTTTTAAACTTATGTCTATTTGTTCTTTTAAAATGATAAAAATCATTAGGAAA

ATCACAATAGGACAAAAAACATGGTATGAAATATAGTTAAAAATTATTATACTATAATAAGATATACATTTCATTTAATA

GATACATTTAGCATAACAAGAATAAAGAATATTTATTTTTCTACATCTAAATAATCTTAACCAATTATATTAGGTTGGTA

ATCTATATTATAATTATTATATTTAATAAATAGTAATATAGGTATATATATATAATCCTAAGCCGTGATTAAAATAATTT

ACGGTTGAAGACTAACATCACAATCTATGATTTGACATTGCTTTACAGAGAGCGTGCATAAATAATACATAAAAAGCTTA

ACAACTAGAATATACAATATATATTTGATCAGTCAATCCAAATAATAATAAAAATAAAATAAACAGTGAAACTCGACTAG

TATCTAGTCTATTGTTCGTTTAAAAATGTGCAAGTTTACTATTACCCTGCACTGAATGACCTTGCATTAGGAATGAAATA

TAAAAGTGAGAAACATTGCTTTTGATTGACTGTATCCAACACCACTGACAACCAACTACCATTAGGAACTTTTCTGCTAG

GAAAACAAATATTGGATAATAATCTAATCGTGATACAAAGAATGATAAAGTTCCAAAATTCCAAGTATACATTTATTAAT

TTTTATAATGTTTGTTTGGTATGTAGAAAGTGGTAAAATGTGATGGGTTACTAATAAAAAAAAAATATATTTATAATAAT

TATTAAAATATTCGCTTAAGATTTACAAAATGAAATAACTGGAATTTAGTTTATCTAGTACAAGTTTGTCATTTGATGGT

TCTACGGGATACATTTTTAGTTCCTTTAAATTGAACCACATCATTGTCTTCTTGTGAATAATCACATGAAACGTCTTTAT

CTTTCTTTGTTGGATTTTCTTTACGTTTTATTAATACACTCATCATTACTGCTGAAATCAATAACAAAACAGCGCAGAAG

CTTATTAGAGGTAACCATTTATTTTGCGTTTCGTCTTCAATTTCTGACAACATTGGTTGATACTCGTGAATTTCTCTAGT

AACGATTCTGGCTGGTCTTGCAGCCACAGGGCTGTGAGGACAATTCGCCTCGCGCACATATCTGGCCCACGTACGCTGTA

CACTAGATGTGAGTTCTCTTCCAGGTCTTCTTAGTTTAGCTTCCTTTATAGGCGTGCTCTTAGGATCGAAATTGCACACT

AATCCTTTGGTTTTTTTATTGTTTACATTGGAAGCAATTGATTTCGTAGAACCTACCAACACGCCTATATTTCCTTTAGA

CGATGCAGCCCATTCCCACATATCCACAACATAACAATCACATTTCCAAGGGTTACCAACCAGTCGTAACGACATAAGTT

GATGATTATATCCGAATTTATCAGGTTTAATTGCTGTGATATTATTGAAACTCAAGTCCAACTGTTCCAACCCAGTTAGT

GACTCTAAAAACTTCATAGACCCTTCTTCGTCACTCTTTAATTTATTATCGTTCAACAACAATTGACTAATGTTAGGAGT

TGTAGTGAAAGCATCAGCAGGTAAATAACTCAAGTTACATTTACTCATATCCAAATTCTTTAAACCGGATAATTGAGGTG

GTAATATTGGTCCTTCTTCGGGAACTTGAAGAGGATTTCCTGAAAGTCTTAACTCTTCCAAGTCTGGATATGTAAAGTTG

TCGTAGAATACTGTATCATTAAGACGACTAAGTCCACAGTTATTCAACTCTAATACTTCTAAACGCGTAAGATTTTGGAA

TAAACCTGATTCGAGGCTTTTAAGTTTATTACCAGACAGGATAAGCGTTTTTAAAGCGGACATTTTTAAGAAAGTCGTTC

TAGATATTCTTTGTAAGTTACTGTAACCCATATCTAAATGCTCCAAGGATTCCAATGGAGATAAAAGTGCTGTAAAAGCA

TTGTTTAAAGGATTTCTTGATATGTCCAATTCTTGTAGACTAGTAAATTTATGGAAAGTATTAGCAGGCAACCTTGTTAA

TCCACAGTCTCTCAATGAAAGTTTCACTAGTTTTAATAAAGGTGATAAAGCTACTGCTAAGTCTGCTGCAGATAAATGGT

TTCCAGATAATATGAGAGTACGCAATTTCGTAGCATTAGCGAATGCATCTTCAGATAAAGGTCCTTTTAAATGACAATAA

CTAAGATCCAGAAAATCTAAATGAACTAAATTGTGACCTAAAGTAGTAGCCACGTCAAGATCAGATAATTCATTTTCAGC

CAACACTAAACCTCTTAGCCAAGTATTATTGAAAAAAACATTTTCTGGTAAATTAGACACACCCGATTGCCTTAAATCGA

GATGTTCCAATCTACCTAAATTACCCAATATTAAAGTCCAGTCAACATGACTTTTAAGACTATTTCCAGCCAATTCTAAT

ACCGTAAGGTGTCCAAGGCTACTAAATGCTACGCTCGATATGTGTGTAAGATTACAGTTATTTAGAATAAGATGTTTCAA

ACTAGTAAGAGGATCAAATATGCCTGGTTCAATGATATGAAGAGGGTTTCCAGAAATATCAAGTTTGTCTAGACCGCTGA

TATTGGCGAAAAATTGTGGTGACAATTTTGTTAAATGACTATTTGCCAGGTATAGGTACAGCAAAGACTTGGAATATAAA

AATGGACCTTCGACTTGTTCTAATGGATTTCCCTGTAATTCTAACGTAATTAATCCAGCGCTATCCACAAATGCTTCAGG

ATCAACATAATTGATCAAGTTAGATGACAAATCTATATCCAATAGGTAATGAAGTCCAGCAAACGTTGTTTTTTGGATTT

TTGTTAAAAAGTTTTCGGACAACTGTAATGTCCTAAGTTTAATATCGGCTGGGAACGGTTCGATTTCTTTTAGGAGATTT

CCGTTCATATTAAGTACGTAGACATTGCTGTCGAGTCCTTCATATGGCATGGATGTCAAGTTACTTTCCGAACAGTCTGT

CACCCAATTAATTCTGAAGAAATGGCAATCGCAATCTTCTGGACAGTCAAGTTGTTCAGATAGAATGGCACTTGTATTGC

ATACGTAAGCCAAAAGTGTGACAAACACTATAATGGATATACGTGTCGCCATGTTGCGGCGGATTTCTCAATTCAAACTC

CCAATAAAAAAAAACGTTTTGACGACTCGGGTGATGATGAAAATTCGTATCGATGTGGACCGGTTCGAGTTAAAGATCGG

TATTAACACTCGCACGACTTATACCCCTTTTTATACAGACTATAAATTCGTAAACGAAACGTTCGCACCAAAGTGCTGGT

GCGGCAATTCTTGTTGATAGCGACTACGACCACCGCAGAGGGACACACGACAACTGGTGGCTGGATAGTGGATAATGATT

TTGGACTCAGTGAATCGTTCCTTGAGCGCGGAACAAGTCAAACCAGC

>contig_3508

GTAGCTATATTCAACTCGGGCAGGCATTTTTGCGGAGGATCTCTAATCGACGACACACACGTATTGACGGCGGCACATTG

CGTTGCTCACATGAATTCGTGGGACGTAGCTCGTTTGACAGCGAATCTCGGAGACTACAATATCAAAAGTAAAACCGACG

TGAAGCACATGGAGAGAAAAATCAAAAGGGTCGTTCGACATAAAGGTTTCGATCAAAGAACTCTG

>contig_351

CCCCCCCCCCCCGGCTGTGACAATGATGAAAAAAATGTATTAAGAAAAAAAAAATAATCTATTAAAACAATTCGATTTCA

TCTATGTATTTATATTAAAAGTTATGATTTAAATCAATAACACAATAAGTCAATAAGTAATAACAACAATTAAAAAATAA

GAGATTTTATAGGTCGACAATGATTGGCGTGGTTCACCGATGTAATAATAAAAGATGTTGCGGGGACCGAGTACAGCAAC

AGCGTGTATTAAAGACTTCAATTTCATTAGTCTCGGAACATTACTATGAATAAATAAATAATTATTGTTTATTAAAGATA

TTAAAAAAATACGTCCGCTCCCAAAGGTAAGTCCTTGATAATATAAATTAAATTTGTTCATTTCGTGATAGGTGATTCTA

GCTGAGTTGGAAGTATCCATTTTAAAAAGTGTCACCAGATAGTCAGATCTCAGTGCACTTTTGGTGGAGGATGAATGAAA

TAATTTCATAGTGATACAGCTGCTCGTTGCGGTCCCCAAAAAATACAAAGAATAAAATGTCATTTTTGTAATGATAGATT

CGTTATAGACAAATAGATAATATTGTATCGACGCGGTACGGTGTTTTTTGTTTAACATGGCGTGTGACAAGGGATCAGCC

TGGGTCGTCTCAACGAGCCCTATAAGGGGGACACTCATTTCCGCTTTCGACCTCCGGATAATCCGCTGCAGTTCCGTTAT

CGTGGCGAATGTCCGTAACAAATATAACGATATGAATATAATATAATATTATTAATTATTGTTTTCATTCACTTCAGTTT

GCAATTGTCAAAATTTGGCGTTTTTTTGACAATGCAGCGGATTTTTAATACCTAGCATACAATTTTACGATGATAATAAT

ATTATTGTTATTATTATTGATATTGATAGGTATAAGATTTTGCATTGGGCGCGTCGATGGATCTATAATCAGTATGGTTA

CGATACAGTGACGACAGGGCGATTGGCATCTCGCCCACCTCCACTCAATAGATTTGAATAGTTTTTTCAAAGAGTGCTTG

TTCGACGTATTTCTGTCGGTAAGGTTTGGACACGTACAGTGCTTCGAGGAACACTGGTTGGCCAGTTGTGTTCTCAGACG

GTCGAAACCACAGTACGAATCCGATAATAACATCTTCTTCTTTTCTAGTTTTTGCTATGAAGCATTGGAAACTTGGATTT

TGAGATTCGAATCCGTCCTTTCTGAAAGTGTCTTTGTTCAATTGTCTGCTGCTTGGTATACCATATTCGGAATAGTATTC

TTGTACTAATTCGTAGATTTGATCACAATCATCAATCAGAGCTCGGCAGATGATGAAATTTATTTGTGGAACATCTTCTA

CCAATGATTCCATAGCACATAACGTCTTCATTCGGTTCATTTTGATTTTATTTATAAAAAAAAATTCTACTTTTAATAAT

TTTTATTAATGCGAATATTGAATACCCCGTAGTAAAGGTTTACTTATAGGTTTACTATTTAATTTATTTTTTATAAAATA

GTATAAACCTTAAGTAGAGTGGTTTTCTTCTGTTACTGTAGTACTACAAATGTACAAGCCATATATTATATTTCATATTG

TTGACAAATAAATATATAGAAAAAGAAGATAATTTAAACATATATGACCTTAAGAAAAACAATGTTTTAAGTAGGATGAA

TATACCTAGGGAGTTAATTACAAATGTAAACAAATATATGTATACAAACATTGAAACATCAAAATTACAATGTACAATAC

GACTGAAATTATTTTGTTTTTCACATGATTTGAATTATTATAAATTAACAAGGAGCTCCTTAGAAGATATATATGAGGGT

TAGAGAAAAAAATTAACATTAATCGCACACTATGTTAAAAAAAATATAGGTGTGAGTGAGTGCTCAATGAAAAACAATAT

GTGGCAGTCCTAAACTGGTCGGATAAAATTTAAAATAAATAATACAGTAAAATTATAATCAAATATCAACTTAAACAATG

ACATTATACACACAGTCTTTTTTTTACAATTCATCGTGGGGTTCTTCAGATTTGGATGCAAGTTCTTCATCTTCTTCTTC

ATCCTCTTCATCAGGTTGAACATCATCAGATTTCTTGGCTTCTTGTTCTTCTTTCTTTCTTTCTTCTTCATCAAGTGCTT

CCTTCATTTTCTTTTCACCCTCATATCTTGATTTCCAAATGGCATCTGCCTTTTCTTTAGCTAATTCAATGTCGTCTGTG

ATCAAAACATTGTTGAAAATAGTACCAGATTTTACTTGCCACAAATCAAAACCAAGAGCACAAATTTCTTTTCTTGCGTA

TAGTTTATCATCTTGTTTGTATTCTGGGTTGTCAATTTCAGGATGTACCCATACTCCCTTGTAATTTGGATTATCAATCT

GTTTTGGTTTCCATTCACCCTTGAAATCTGGATTATCAATCATTGCTGGTTCCCATTCCCCATCCATTTCATCGTCCCAA

TCTTCTGGTTTAGTGGCAGCTGGATCAGGAATGTGTTCTGGCTTATCCCAATCTTCTGGTTTTTCATCAGTAGGATCATC

AATAGTAGGCTTCTCATCCCAATCTTCAGGTTTCTTAGCTTCAGGGTCCTTAATCTTCTTGGGTGGTAAAAAGTCCCAAT

CGGCTTCTAATTCACCAGATTCTACCTTTTCATTGTCAATTAATACTTCATAAGTATTATCAGATTTTACAATAAGTGTG

TAAACATGAGTATAAACATCATCACGGCAACGAATGTCTTTATTGATCAATAAGTTTTTGCCATTATAGCTGAAGATAAC

ATGCACTTTCTTTGTACCAGGTCCACAAATGTCAGGTCCTAGAAATAAATAAAACTTTTGATTAAAAACTTGAAAATTAC

AGATATAAAATAAATTGAAAATTAAAACAATTAGTAAAAACTATGTTTTTATTTGATGAAATATAACATCTTAGTAGGGA

AAAATATTTACTATTTAAAAATTGATTTTTATCATTTAATATGTATTATATTATGTACTCATATGTTATATTAACTCTTG

ATTTTATTCATAATCAAATAACAATTTTGTTATTTGATTATGAATAAAA

>contig_352

GCGCAATGTGCTTATATATTTACTTGGCTGTAACAGCTAGAATGGTAAAAATCATTTCAATGTTGATCGACGACAAATAT

CATTGCAGACCACTAGCTAATGTTTGACTTGTAGCTGTAGCTAGGTTGCTATGGCTCGCTGGTTTTTGGCTGCGTGCCTT

TTAGTGACAGTCATTGCAGTACAGAGTTTGATAGTGCCTAGGGGTTACCGTTTAGATTTACATCCGTATCCAGGTGACGG

AGTATTTAAAGGCAAAGTCCGAATTGATATCATCAATACAGGAAATCAGTCCGTAGATTCTGTGACACTAGACGTACACT

CAACTCTCAATGTGGTCGATCGTGAGGTTAAAGTCACTCGCGTTGCGAATTTGGACTCGTCAGAAGAAGATGAAGTTTCG

ATGGAAGAAACTTCACTAACAGTCACAAAGTTAGAAAAACGAAAGGACATACATAAGTTTCAAATCACAACTAAACAGAG

CCTTAAGAAAGACGTTACATATCAAGTGGAAATTTTATTCGACGGGACCCTAGGAAGTGATTCGTCTAGTCCGTTTTATC

AATCAACGTATTCTAATTCAAAACATTCACAACTGAAAAAATGGTATGTAGCAATGAATTTATTGAAAGGAGGAGGAGCG

CATTATGTATTTCCTTGTTTCGAAAAACCTAATCTAAAAACTTGGATAGAGCTATCAGTTGCTCACAAAGGAGACTTTCA

TGTTCTAACAACTATGCCATTGGTTGATACAACAAATGTGACTTCTGAAGGCGTATGGGTTCGAGACCATTTTTCTAGAT

CACCGCCAATGTCAGTTAATTCTTTAGCCATGTTCCAGTCTGATTTTAAACATCCACCAATGGGTGAAAATAACGGAGCT

AAAGTTGGTGTATGGGGCAAAGAAGATATTTTAAGATCGTTGAATACGGCACAAAAAATGCTTCCAGCAGTTCTAGTAAA

TTTGGAACTTTATTTGTCTAGACCATACCCTTTACCAGAACTAAACTTGGTGGCTCTCCCCGGTTATACTGACGACAAAC

CTATTGATGCTTGGGGGTTACAAATGTTTAGAGAAGTAGACCTTTCTAAGAAACGTGATGATTTTTGGGTAGCTCATCTT

CTCGCTAAATATACATCATTACAGTGGACCGATCATCTTACAACACCACTGTTTCCGACATCATTAACCCTAGCATTGTC

AAAATTCTTAGCTGATAAGGTGGCATGGCAGTTAGAGAAACCAGTATTCAATTCATACGTAAGTGACATGTATGGAATGT

ATTTAGAATATGATAAACCTGGTTTTACAGAAATAAAATCACAAATGGAAGCACTGAACGCAACAAAAATTCGTTTCGTA

TTGGGAATGCTCGAACACATATTTGGGCCTCACTCGTTCCGACAAACAATCCAATATTTTATGTCTAGCAAAGAATATCA

CGCTTACTCTGAAACTGATTTTTGGAACAAGTTGTCTGATGGAGCATACCATGCTGGTGTATTGATCAGAAATAAAACTC

CCGTTTCGATTGAACAAATTGTAAGGCCATGGTTACAAGCTGATAGACTACCTGTGGTTACATTTAGAAGAAATTACATA

AACACAACCGTTCAAGTTATGCAGGAACCATTTGTATTAAATGACGATGAGGATGATGTGTCATCCCCTGCAAAGAACAC

TAAAAATTATTTATGGTGGATTCCCAATATTGTTGTGTCACAAAATAATTTGACCGTACCAGTTAGAAATATAAGTTGGT

TGAAACCAATAGCAAGTCAAGAGGTAGAAATGAAACAATATAATTTCACGAATAATCAATTTGTACTCTTCAATCCGGGG

AGCTTAGGACCATTTATTGTTAACTATGACCTAAAAAATTGGGAACTGATAGCCCAACATTCAAAGCGTTTCCCAGTAAG

CATTCGTCAACAGTTATTGCACGATTCGCTAACACTGGCTTTATCCGGTCGTTTATGCTACGTATATGCATTCAATGTTA

GCAAACTTATCGAGCAAGAGCCCGAGCCTGCTGTTTGGAAAACGTATTTTGGACTGGCCACGAGATTACGCACTAAGTTC

CAAGGAACACCAGTAGCTCCTAAATATGATATATACTTGAAAAAAATGTTAAAATCTGTGAACGCTGCGCTAGAACAGCC

GGAAAGGAGTGAATCCAGTTGGAAGGCAAATTTCCGTGCAGAAAATAAACGTTTGCTCTGCGAAACAGGGCATCCGACTT

GTCTAACAGAAGCCCGGGATGTGCAATCGAGAACTAATACCTCAAATCCCGACGAGGATAAAATATTCTTGGATAATTAC

TTATGTATACTTCTCGGATATGGAACACTGGACGAATGGGAGTTTGGGTTACATAGGATTATGTACTATCCAAAAAATAA

GTCTCAAACAGAAAGATTATTTATATTTAGATCATTAGCTAGTTGCCCTAAAAATGAAACAAAATTTGTCAGAATGTTGA

ATTTGACGTTAATGAGTGGAGATCATAAGTTTAGTGAAGAAGATATGTTAACAATGTTGACTGTCATGAGTACAGTATCA

TTGGGACACGAAACAATGTTCAAATTCATGATGAAAAACTTTGAATACCTAAGCACTAAGTTGGAAAAGACTGTATGGGA

ATACTTTGTTAAAACGTCATTCAACAATTTTAGAACAGAAGAAGGACTAAATAAGGCCACCGAGTTTTACCAAAGAAACA

AAAGACATTTTGTTTCTGTGGATGATATTATAAAAAACGCACTAGAGAAAGTCAGGATCCAAGTGGATTGGGTGAGGAAA

CACCTTACTCCCTTGGACGGCTGGCTGACGAATGCCTTGCAAGAGCCATGGAGACCACACGAGTTCCAATTCAGAGACGT

GCCGTCATTTGTCGTTGGATAAATCAATATATTAAATACTAAAAACTACATAATATTAGGATTTTAATTGAATTAATTTT

TTCCAATTTTATAACATTTTAAGATGTGTAAAATAATAAATGATTTTCTATATTAATTTTATAACACAATATGTCACAAA

GTATTTTATTGAACTACCACTTAAATGATGCATTTTCATTTATAATAGCAGCAACTTCCAAACTATTGATAAATTCAGTA

TACGTCGACTTCTTAGAAATATGTACTGTTAGTTTGCCACAATGGTTTTTTTCAAAATTAATACAGGAGTCTAAAATATC

CGAAGTAGAAGATCTAGCGATGATCGTATTGGCTTCCAGTTGAGTATTATTAGGGATGTAATCTTTTAATGCATTTAATT

GGTTCATAAGCACATCTATCGAACTATTTATTTTTTCTTTTTCCTTTGAATTTCCAATCAATTTTTCAAAATCGACATTA

TCTACAAGTTGTAAGCGTTTTTTATTGTCGGACGGACAGCCATCCAATAATAAGAGATTGACGGTTTCACCAAATCCTTC

CATTATCTTGGCAAGTTCGACAGCAATATTTCCGCCCCATATTTCGCCAACGATTGTGTACGGTCCTTCTGATTGAATTT

GTCTTAAAGGCCATAGTAAACCTAACGCTGCATTTTCGACGGAGTTAATGTACGATGGAAATTTAGCACAAAAGACTGGA

TGCATAATTTGATTGATTAAAGGTTGTATTCGAGAAACTCCCAATCCAGGAATAATGAACACTGGTAATAAACTATTATT

GCCAGTAATCGGAAGACTTTTGGATGTGTATTGTTCAAATCGTGGATATTCCGGTAAATCTATATTAAGGTCAAGTAATT

CGTCGATAGTTTTAGGTAAATATTCTGAGATTGAATCAATTTTTTCATGTTCAGATATTAACGGATCACTCAAAACAACT

GAAACGGGTTTAGAATCAACTATAGACTCTGTCAATTTATTCATGTACTTTAACATGGTTTCTGGTTTTCGAACAGATTT

GTCGCATTGTACGTTAATGCAATTAGAGTGTGCATCGTTTCTTGACTCACAGACTTCATTTGCATCACTTTGCATGCAAA

TGAAATGTTTTAAGTCTGGTAAAATATTTCTACAAGCTTTGTCAATGTTTTTTAATTTTGTGTCATTCATTTCTACACAA

AACAATGCCTCAATTTTTGAATACGATGTGAATTCACGTAACAATGTTTCCCCTTCTTTTATAGTATTAAACCTTTCAGC

CGAAGTCATAATAAACGAAACATCGGAGTATTTTTGAATTAGTGAATATATAGTTCTTTGACTTCTTCTCATTACTGAAT

TGGTACCATTTATGATGAATATCAATTTTTTTACGTCTTGCTGTAGAAGCCAGTTAACGGTTTTAATCCACAAATTCGTC

GCTGAACCAATAATTACGTATGCCATTTCTTTTTTAACAAAACTATGATCTGAATTATTTGTTTCAGTTATTTTTTCTGT

ATTATTCAAATTGTTAGTAGATGTTGTATTAGTTATCGGTATTTCTTCATACAATCTACTGACTATTTTATCCTTAATAC

CATTTTCAACAAGCTGCTTGAGATGGTTTTTGGTTTCGGCAGAAGAATTTACTATGCTTGATAAGCCTTGTACACCGTAT

AGTGAAATATTTTTTAAAAACATATACATACCAATTTCTGTGTGAACACTCACATCTTGTGAACCTATATGTATAAATGA

GCCAAACGGTTTTATACATCGAAGAGAAGCCCACAGGAATTCGGTTGGTATAGCATTGATAACGATGTCGCATCCTTTGC

CCTCTGTTAAACACCTTAGTTGTATTTCAAACTTGCTATTATTGAGATCTAGTATGTTGCCGCTACTCAACT

>contig_3571

GATTCGGCGAGGTGGGAGAAGTGCCCGATAAATATAACCGACAAACCGGCACCGGCTCTAAACAGTCAGTCGACATACAC

GCCAGTCGCTAACTATTTCAAATTCTTTTCTATCGTCAAAACTCGAACCGACCGATTCAGAATGCCGATCGACTTCTACT

ACACTCCCGGCAGCCCGCCATGCAGATCCGTTTTGCTGACTGCTAAAGCTTTGGGCTTGGAATTGAACTTGAAAACTTTA

GATCTCCACCACGGTGAACACATGAAACCAGAATTCCTTAAAATAAATCCTCAACACTGTGTACCAACATTAGTAGACGG

TGACTTAGCTTTGTGGGAGAGCCGTGCAATCATCGTGTACTTGGTTCAAGCTTACGGTAAAAACGATTCACTGTTCCCCA

AAGACCCGAAGAAGCAAGCGCTGGTCAATCAAAGACTGCAATTTGACGTCAGTACCCTGTATCCGGCGTTCGCCGATCAA

TACTACCCGTGGATTTTTGCTGGAGTGCCGAAAAGTGACGACAAGGAAAAGAAAATTCACGATGCGCTTGCATTTTTGGA

AATCTTTCTGGGATCTTCCGCTTGGGCTGCCGGTGATTCAGTGACTGTGGCCGATATCGCTTTGGTTGCTTCCATTTCGA

CTTTTGAGGCTGTCGATATTGACTTGAAGAAGTACGCAAACATCTCTAAATGGTTTGAAAAGTGCAAGAGTACATTGGCG

GGATACCAAGAATTCAATCAAAAAGGAATCGATGGATTCAAAATCATGGTGGCTAACCTCACCAAGAAATAGGCGTTTAT

ATTTATTTACGTTTATCCCGATCAGATTTTGCGTTTCGTGTTTTGATTACACCATTTTTGTTTTATTCTATTCAATGGTA

CAATAATTATTGCTTTTTACAAATTTTAAATTATAAATACACACTTTTTTATTTACTTATGTGCATTTTTGTAAGTATAT

ACTAAACGATTCAACATTCTTTTAAAACATAAAAATAAAAAAGTGATTAACCCGGATTTGTACAACATACACATGGTATG

TTTTAACAACAGTTACTAACGAAGCATTATTTTAGTACTTTTCGTCTTTCGATATCGTTTCATAATATATGATGGCATAT

CGGTTATCATATTGCTATATCGAATACCTTTAATATTATTATAGGTATTGGAAGAAATTTATTGACGATTATATTTTAAA

TTCGTGATAAATAGTTTTTATAAATTTATTATTTTTTATTATACTTAAACTTACAGAATAAAAAAAAGGTGAGCCACAAT

ACTCACGATAATTGTCTTACTTGAAAATGAGTGAAACAATTTATATTATACTGTATACAAAATATGGCATATATCAAACA

ATGTTATGATATTTATAAAATGAATAACCATTAGCAGCATTAGCAACCATTCAAACAAACCAAAATATATTCAATTTTCA

CGAACCAAAATACTGTAATGCCTTTTTGTTCGTTTGCATAAAAACACTTGAGCTTTAAATGTTCTTGTAACGATAGTAAA

TAGATATTTCTACATAAAAAATGTATAGCAATATCATAATAAAAATAAATGGTTACTTCTACCTATGTATTTGCTTTATA

GTATTGGTGATATATTATTATATAATATGACACTGGTTAAATTTATATGCTATAAAAAAAATAAAATAA

>contig_371

TTTTTTTTTTTTAAACAGTAAAATATTTATATTTATTCTGCATCAAAAAAACATTATATGATCTATTTGATCTAATATTA

ACGTTATAGATTAAAAACAAATAAGCTAAAATAATTATAAGAACCTTATTTTTTAGATAGGTACCAATTTAAGGAAATAG

TTTTTTTTTTTGTTCTTAAGAGTAGTATAAAATGACGATATGTATACGTATTCCGGTACAAAATAATTACCTATACATTT

TTAAAATGACAATTACGATACAAAATGAACTTTTAAAACTAAAATCGATTTTGTATTTCGTGTACTCTGTTCAGTTTTAC

AATATGTTCTTAACAGCTTTTAAAATGGGGTACTTATTCGCGTCGCATAAACCTCTGGCGTCATCCAAGGATAGATCCAT

TAAAGCTATACCTCCCAAATTGTTTTGCTTCACATACGTGGCTTTTTGTTTGGCTGTTTCCGGTTCCTCGAAACTAATCC

AAATTCCTTTTACGTCGTCTTTGGGCAAACGGAAAGCATAAGTTCCGAGATTTTTGCTCGGGTCCGGTACGCGTCTGTAA

AGTGTAAGTGAGGTGGTAGCCGCAGTGCTCTCAACTAAGTGAGGGCAGATCTCGTAGTAGGCCAATAGACCTTCGGTCTT

AGTATATGTGCCCTCTTCGCCGGGACCGTCAGCGTTGACGGGTGGTGCTCCGGACTTACCACTGTCTTTGTCCATCTTCC

AGGTTCGGCCGTATGTGGAGATTCCCAACACTAGCTTGTTGCGATCAGCTTTAAGGGCAATGGCTTTGTTGACTTGCGAC

TCAATGTTCTGCCAAGCTAGACGGTCGGCGTACACGAACTTGAAAGGCGATGCGTGGTCAGCCAATTTAGGCACTCGGTC

TGGAGTCCGGAAGTCATACGTCATCAATGTGAAGTGGTCTACGTACTTTTGCAGCCCCGGAAAATCGAAGTACACGCTAG

AGTTTACGTGTGGAAGAATGCTGACTGAAAGATAAGGACAATTGTTGGCGTTTAATACTCCTTTGACTTCGCGGACCAGT

GCAGTGAATTGTTCCTTGTGTTCGGCTTCCTTAGAGTCCTTGCTAGTAATACCCACTGTTTTAGCCACTTTGTGGATAAA

AGATCCCCACGTGTGTTTTTCCTTTTTCTCATTAACCACTGGGAATTGCCAGGCCAAATCAACACCATCGAAACCATTTT

CCTTAGCCATTTCCGAAATGGTGGACGCAAACTTCGTTCTCGATTTCGGCGTCTCCAAAGCAGTTAGATATTTATCAGGA

TCTTCGACATCGGCATTGCCACCAACCGACAGCAGTATGGTAAGACTGGGGTAAATCTTCTTAAGAGCTGTGATGGACTT

AAAGTTTCCCTTTCCTCCCTTAACATCTTTGCTCTCGGGTAGGTCCAACTTTGGATCCAATGATTTCGCTTTAAACTTAT

CATCGTCAATACCAGCATAACCGTACACTAAATGCGTACAATAACTTAGGGCGGGTTTTAACTCCTCGGCACCTACTTTA

ACGACATCTTTTCTCCAAAGAGCTCTGCCATCATAATAGCATACTGTTCTAAATGCTTGCGAAATGTCGACAATAGCCAG

AAGGCCACATAGTGCGACCGCACCGAACAACTTTACGCCTAACATCGTACTACCGAAAACAAACTCAAACTGACGACCTG

TTTGGTTTGGACCAAAGGTCCAAGCCAACGATTATTTGCTGTACGATGTACACCTACCTATCTAATAATTTTGTTATATT

ATCATCACCCCTACTCGTTCG

>contig_37590

GCCTCCTCTGTTCAACCACTACTCCAAAGGCCGAGATAACCTGGTCTTGGCGCCTTTCGGGGTAGCTACTAACGTGGCCA

TGATGCTGGAAGGTTTACAAGGTCGGGCCGCAGAAGAGGTAACCACGCTGTTCCGGCTCCAGGCAAAGGAAGTACGTCAA

CAGCTGAGGAGAGGTTTTAAAATGATTTTTGATACGTTCGGGGTAAGACTAAGAAGATATCTTTTAAAACCTATATTATA

AAAATGTAAAGTGTAAATTCTTCATACTTCATTATTATACATATTTACGCTTACCATTTTAGGAAAATCTTCAGAAAAAA

ATTGTCGTGTCGTCGGAAGTGGTGGTCGAGCCATATGGTCTCATAATAATAATGATATTGATATCTTGTACTTACGTAAA

TACAATATTGATTCTGAACAAAAATTTATTTATAGGATGATTCGGATGGTGATTTTGCCGGATCTTACAACAAAGCATCT

GTGACTAGTTCCCGGGTTATGCCATCGTCATACATAAACAGACTTTCGAAATATTACCAAGCTAATGTTACCACAATACG

AAGCGAGAATTCGAACAACAGCTTAAGTGATATTTTAGAATTACGAAGCGATACAGGAATAATAAGTCATTGGAAAGACT

ACCAAAAACTAGCAACGTATACTTACTTATCGTATCAACCATCAGCACCATTTACTAAATCTGACGGTTCAGTTGTCTAT

GTCCCAATGATACCACAGACCGGAATGTTCAAAATCGGATATGTACCACAATTAAAATGCTTAGCTGCTGAATTAATGTT

TGAGACCGACAAAGTAAGTATGCTCATCATGATGCCGGACGACGTTAACGGATCGGAACTGATGATCGAGAGGTTGTCCA

AAGACAACTACTTGGACATTCTGGACTCGCTGGGCTACCAGAACACAGAAGTACTATTACCACAGCTGGCCGCATTCACC

AACGGTCTGGATTTGGAACCGTTCTTCAAGAAGCTCGGCGTCAAGGCGGCCTTCAATCAGACGGTCAGTGACAACGCCGA

CAACAATAACAAAACTTCGTCGACGAGCCGTGATCACGCGTCCAAGTTTCCCGTGGTCACTTTGGTGTCGATGAAACAGA

ACGCGTACTTTTCAATGTCGTTCATCACAATCAATTCGGTCGGCTCGGTCGGCACAAAGTTAGGTATCCGTTCATCTGGC

TCGAGACAAAAACGAGGCATTGCTACTAAAGTAGTATTTGACAGGCCGTTTGTATTTTTTGTTTATAACAAATTAACTGG

ATTAATAATGTTGGCAGGAAAAATAACAAATCCTACACAAGTGCCCACCACTAGTTAATAATTTATAAATAATAGTAATA

TTTTTTGTATGTTTAATAATATAAGTAGAGACTTATTATTATTCATGTAATGATTACTTGCTACCTAGTTACAAGTTATA

CATAATATTTTAAATATAATTTATAATTAAAATGTTTTAACTTAAATTATA

>contig_37766

ATCTGACGCGGCACGGTCGTCGTAGCGCTCAGCGATAAATGATATAATATTGTATCGATTTACATATTATAATATTTTAT

ATCGCGTCGCGTATACGCTCATACGAAGTGCCGTCATTGGCGATCTGCACGATAAATATTGTTATTTGATTAGCACGCGC

GAGTCGGTGTTTACACGCACACGAAATCGCACGCGATGAAAACGTTACTGTGGTCCTGCTGCTGGGCGCTGATGTTCGCG

TCGATGGCTCTGTCCACCGCGCCGACAAACAGCTCGTCCTCCGCGGAACAGCACGACGTCGTCGGGACGTCACAAAACTC

CACGGCCGTCGCGACGTCGTCTCCGGCGGACGACGGTGACGACGGCGATCGAGCGGCCGATTCGGGTGAGTTGATCGGTG

CGGGTGACCGCGGGGAAGACGACGGCGGCGGCGGCGGC

>contig_37882

TTTCATCAGTTGAGTTAAGCACCAACTCTTTTCTCGCTTCTATTAAGCTCTGTGCAACGTCATGTCTGATTATGCCGTTT

TCTTCCCTGTATTTGATGACCTCTCTAAATGCCGAAGTATAAAATGAAGTAGCTTCAGGTGAAATTTCGGAGATCCCCAA

AAATTTCAATATTTTTGGTGATACCGTTTCGGTCAACAAGAACCGGAAGTTCATCTTCATTATTTTCCGGGCGTTCGACC

GGAAATCAGAATCCTCGTTCTTGACTGTGTCCAATTTCAAACCAAATGCACAAGTCCCGATGACGTCTGTCGAGTACTTT

CCCATGGTCTCCTTGACTTCGATCTGATTGTTTTCAATCATTTTATCCTTGATAAATCGCATCAGCTCATCGCTGCACGC

CACTATCTGGTCGTGGGTGAGCTTCAGCTTGCCTGACGTGAACCCCGGGCTCAGTTTCTGTCTCATCGTCTTCCACTTAG

ACCCTTCCAGGAAGAATAACCCATTAGCAATTATATTTAACGCTGGATCCTTGTGGAAACCTCGATCCGGGAAGTACGAG

AAATCCTTAATCAATATATTATTGATCAGCTCTGGATCTCTGATCATCAGGAACGGCGTCTTCATGTGATAGAAACCGCA

GTATTTTTTGTCGGCAAACCGTTTATAGATTCCATCGATAGTGGCCAACTGATGTTCGATCAAAGTCAACATCTTCACCG

TGTTGCCTACAAGCGGCCACGGTCGGTCATGGGGTACATTCAACTTTTGCCACTTGTCGTGCGTGTTGGTCGAATAATAG

TAAAGAAACGCCGCGATGGCCACGATTGCACTGATAACGGGCGTGTGGAACAAAAAACCGATCAGACAAGAAATCATGGT

GACCGATTTTAAATTTAAAACGTCAATATAATGTACAACAATATCGCAATGAATTAATTTATTTCACTCGGAACCGTTTC

CGCGTGAAAACTGCAACGTATAATGCGGTGCATAATAGCTCAAGCCGATTGTGTTATGTTTATACTCATATACAAATCTG

AGTTTACCATGCACAAGTGGAATTTAAAGCATGTTTTTTATCTGCG

>contig_38

AATAATAATAATAATATTTAACATTATCTTAAAATTACAAAGAAAAAAATTTGTCATACATGAGAATTGTTGGTTCTGAA

AATTAAAGCCGTTGGCAGTGTTTAATTTTTGATCAGAAACACGTTTGTACATATAAGATAGAAATTAATGAAGGTGTAGT

TTAAGTTTATGTAACAACAACCCCCTCCAAGGATACTTTTTAATTGAATTCTACCACATAATAAGTTATCTGAATTGTAG

ATATAATTAAAAAAAGCATTTATTAAACGGCGTCATTAGTGAAATATTCCTAAAATACAAGAAGGTGTGGACAAGTGGAA

AAATAATAACTTGTTATAACTATGAAATTAATGATTTAATGCTAACAATATTTTTTTTTAATTTTAACAAATTCTACAAA

TATAACACTGAATAAATAATAAAAAGGTCTTACGAGTAACAAATAAAATGGTTCACACCAATATATACGTAGGAAATAAA

ATATTAACGTTTCATATCTCTCTGGTGTCAAAAAATTGATTGTAATGTCACTACGTTAATAGGCTTAAATTAGTTATGTT

TTGAAGAAAGTTTTTTAAATTTCATTACAACAATTTAACAACAAGCAGTGACATTATATATATATTACATAATAATAAAC

ATAATTTTATACGATCATATACGATTTAAACTTAATTTATGCTTATTGCTAAAAATAAATTTCCAACTGGGCAACTTTCA

AATTTGATAAGCACCCTTAATATACTAGTTATAAATAAAATAAACCACTTATAAGTAGAATAATAATAAATATATATTGC

TGGGGGACAGAGGAAGTAATTATTATTAAATTAGATGAAAAAGTGAAATTGTTAGAAATAAAATCAGCGTGTAATACTCT

ATCATAAAACTAACCTACGAATAAGTAACTCACATTTTTTTTTTTTAACAACACTTTTATTATAAGCTACCGATAAAATA

AATCTATGACCAGAATTAAAGTAGTTTACTTTTAGTTAAACGATAGGATAAATTTTATTTTATTACGAACTGAAGGAATA

AACATTTATTGAGCATTAAATGCAATATTTTCAGGCTTGTAAAAGCGCAGTAAACTTAAGATTTGGATAAGTTAGGCATG

ATAACATTGAAATTTTAACAAATCATTGAGGTTAACAGGCAGGGAGGGGGGCGATTACTGTATTTTATTAATAAAATAAA

AACACACATCAACTTTGGTAAAAAAAATAATAATAAATAATAAAATAAAATAATATTAATAATAACACAACAAAATACGG

AGAACAAATGATTTGCACAATGAGCCTAAATTTGTTTATACAATACAGCTAGTATACCATTATTGAACCTGAATTATTCC

CAGTAACGCGTGGGAAAACATTCAATTTCGTTTTTTATGGAGTAAAAAATACAATAAGCAGTAAGTATAACACTGTTTCA

GCTGGACCAAAACAACAAATAGCTGTTTCATTTAGTTATTTGTTAACAACTATGATAAATTTTTTTCTAAAAACATAAAT

AAAAATTGGTCTGGCAAACCTAATGCATCTGTATCTAAAACTTTATTCCTTACTTATGACCTTTGTTTAAAATGTGTTAT

TATTCATTTAACTGTCAAATTATTTCCGGTGTCGTACATCACCACCGCTGTGGTCGTTAGCATTGTTTGTTTCTGGCCCT

TGGTCTGTGTCACTTTCCTCAAATTCAGACTTGTTACGTTTCTTTTCATATATAAATATAACCAAACACAGAATGATCAC

TTCAGCACAAATACCCAAGAACGGCCACAATGCAGCCATTTTATCTTTAATGCGCACGTAAGAAGCAGCGGAATTTACAG

GCTCAAAGACAACACTATTCTTTCCACTGCAGATGAAGTTTCCTCTATCTTGCATCGTGATCGGATCCAAAACTAAGATG

GCATTGGAAATATTCTTTTTGTTAGGTAAAAATTTAATATGTTCAGAAGGCACAAGAGTGGTATTTTCAAATTTCCAACT

AACTTCTGGTGTAGGTACACCATATACAATACATTCCAATTCCATTTTCTCACCCTCAATAAATGTAGCAGTTTTTGGCA

GTTTCATGTAAGGCTTTAATTTGTATTGGGTTCAGTCTTGATATCACCAGTGAACTGAACGGA

>contig_38300

GTGGTGGGTTCGTCTAAAAATATCACCGGTGGGTTGTTAACCAATTCAAGTGCCACGGATAGACGTTTGCGCTGACCTCC

AGACAATTTCTCCACATATGTGTTACGGCACACTGATACCCCTAAAAGTTCCAGCACCTCGTCCACAGCTTTGTCTTTGT

CTTCTTTTGATAATTCACGGCCAATTTTCAATCTAGCTGCGTAACTCAATGATTCTATAACTGTAAGTCTGGGCTGAAGC

AGGTCTTCTTGCATTATGTAACTTGAAAGTTTTTTGAAAAGTTGCATATTCCTAGGATGACCATTAGTAAGAATTTGTCC

TTTGATGTCAGTTCTCACGTATCCAGCGAGTATATTCATCAGCGAACTCTTCCCAGCACCGGAAGGACCCATTATGGCGG

TCAGGTGTCCTGAACGAAAAAAACCGTGTATACCTTTCAGTATCGTCCTTTGTCCCGCTGTCGTGTTGACGGCATAAGTC

AGGTCGTGGAATTCGATGTCGACGGCCGGTCGTTTCGCTTTGTGCGTCAACGTCTTCATGTCAACCCGGGTCGGGATCCT

GGTGATCGGCCTGTCGGCGGAAACGCTGAACATCCTCGTCCTGGACGAGTCGACCAGCGGCATCTCGTCCTCGTCCTGCC

GTTCGGTACTGTACGTGGATGAGTTATTGTCGTCGTTGTCGTCGTCGCTACGGTTGTCGCGGTTGTCATTCGCCGTAGCC

GCAAACGAGACGGTGCTCTGCACGCGACCCATCGGAGGCGGTTGCTCGTCGCCACGGGCCATGATCGCGACGATAGTAGT

TCAAATATATAATATGTCTATATTTAAACGACGAAAAAATAAACACGGTTCGAGTGTTGATCGGTCAGTGGGCCGCGGTA

ACGGACTACGGTGTTCGCGAACTTTGCGTTCGCACTAAGGATTACGTTCTGCGGTCGGTCCGCCGAATGAGCTGTGACCG

GTTTCCGTGTGTCTATGGTCACGCGCACACAGCATCAGCGCGTCTGCCGAGAGTATAAAATAATATAATATTATTGCGGT

GTCGCGCGGTCCGT

>contig_3884

CTAAAGTTCCTTATATTAATTGTTAGAGAAACCTTACGTCGTTATTTTACCAATAATACTTAGATAGGAATAACTAAAAT

AAATTACCGGATAAATACTCTGAGCACCTCAAGGCCGTTAAAGATCTTTATTGCGTTTCAGTTTTTATGATAATATTATA

ATATGTAATTATGTAAATTATACAATACCTATATTACATGCCGTAAAAAGGTAACTATTTTATTAATATTTTTGTTTTGT

TTTAAAAAACAACATTTACTTTGTTAACTTATAAATCATGATTAAAAGCTTCAACATATATAAATTATGTATTAAAGAAT

ATACCTAGGTAATCATTAATCAAATCAATGTGAGCTTCTAGAACCGATGTATGATGAAATGGAGGAATACGAAGGAATTT

AAATAATGTAAACTAATATAGAAGGTAAAAAATAAAAATAATATAGTAAGGTAATATTATGTTTAAGGCGTAAATTGAAA

TATATATTTTATGCTCAAATAATAATATATCAGAAAATCAATCGACTCAAATTCGATTAACACCCAGTCAAAGTATTAAT

ATAAATTAAAATGCCAATAGTTGGAGAAAAAAATGATGTCTTCATTGTGTAATCATTTTAAGCTCCTTGTTGATCCATTG

GGCATTGGCCGTTACCGATGAGTATACACCGGGTATATTTTCATCACCGCACCCAATACCCCAAGACACAATACCCACTT

GCATATATTTTTTAGACTTCTCTTGTGATCCTTTCATTGCGCATATCAACGGTCCGCCTCCGTCACCTTTGCACACGTCT

ACTCCCTTTGAACCACCAGCGCAAATAAAACTATCGTGTAAACGGAAAAAGTTTCCAAGGCGCGTGTTCCGTAGTTGCTG

CTGGCATAAGTCATTAGGCACAAGGGACAGCTCTACTTTTTTTAACACCGTTTGATATCGGCTACCTTGTTCGAATCCAT

TTTTACCCCATCCTGTTGATATACACGATTGTGGGTCGTAGTTTAATTGTTTTTCATTGCTGGGAATGCATATAATGTTA

ATATGTGGATATAGTTTAATTTCAGATTCTAGTTCGACTAATGCAACATCATTGAACATAGTTCCATTATTGTGATTTGG

ATGATAAACTATTTTTGAAACACCACGATCTTGGTGCGAAATTTCTTTTTCTTCTTCGTTATTATTGGACTGAGTATCCC

AAACGCCCACTCGTACTTTCATCAATTTTACATTTTTATCTCTGACACAGTGTGCCGCTGTTAAAATAACTCGTGGGTGT

ACTAATGAACCACCACACAGGAAAACATTTTCAGTTTTTACATCAGTCTCTTCATTTATGTCTGTTTTTAGAATAGCAGC

CATCCATGGAAATTGGCCAAAATACGTGTTTGAATAATCTTCAGAATTAACAATACGTGTTCCTATTCTCTCAGAATTTA

TGGTATCTGGTGACTTACCGATACCACAACTACGAACATTATTATAGTTGGGTTCATCAATAACAGGTGTTTCCTGAGTT

GATGATATGATATTAACATATTCATTTATGCAACAAACTTTATTGCTACTACAACTGCCTTTTCTTATATTATTATTTTG

CCCATCATTTGTTGGTAAACATTGATTTTTAGATACACATGTACATGGTTTATTATTCTCGATATATGGTTCTTGAGTAG

ATGAACTAATTTGAGATAATTCAGATGACGATGGAAACGATTCTTCGGTAACTGGTGTATCACTTTGTACTCGGCAACAT

ACCAAATCGCTTATACAACCTAATTCTGTAGAT

>contig_3885

AAACTATAAATTATTTCTCAAAAAAAAAGTTATTGTCGGAGAAACCTTACCAATATAATATAATTTATATTTCAATATAA

ACGAAAAAAATGATTATTATTTCATTTTAATTATACTTATTTTAATTATCAAATAGGAAATCCGAGGAGGGAAGCCTTAA

ATTTATCAGTATAATTAATACCCACTATAATACAGCAGTGCTTGAAGCAAACACACAGTAACCACTGAAAAATGGAAATT

GTTATGGAAATTCAGTAAAAAGGTGCACCCATGAAAACCGTAAGTCATACTTGCAATAATAATATAATTATAATTTACAC

ATTATATAAATATAATAATAATGACAATTTGTTCACGGATTCGACAGGCGACCAAGAAATAAGACGTCATCGGTGCGCGA

CACTATCGCGACGACAAAAGGATGATCAGCGTAGAATTCAACATTTTTCGGCGGTATGAGCGGATATCTGGCACTGTACT

TCATCATAATCATTGCTGTAGCTGCTGCAGCCTCAGTTCCTTTTTCATTGACATCAACATATGCTTTATGCAGAACCTTA

CTTACAAATAGATCACCACTTTCAACAATGTTAGAAAAGTTTGCCGCTCGTGTAAACATTGTTGGACATCCAAGATTTGA

TAAGACATCTTCTAATTGAAGTGAATGCTCTAATTTGAAACGAGGTAATTTGACAGTAACATGATATTGGGTCATTTTCT

TTGAAATATCATGTAAATTAATTTTTGAAAAATTATTCTCCAGCTCTTGCAAGCCATCTTTAGCATCAGGCAACAAAATC

ATCATTTTGAAAGCATGGTGTTCATATGGTAATTCAAGAGCAGCAAATTTTAGGTCACTATCATGGTAGTATTGCAAATC

ATGTCTTAAAGTCATCATTTTGACTGTTACTTTGTTGTTTGGAGTCAGATAAAAAGAATCGTCTTCTGCATCATTAAATG

TATGTGCCCACCCACTTTTAAAATGTACAGCATTTGCCAATACTAAAGCAGTGGCTTGATTAATGGAGTCTTTGGGAAAT

AAATCTTTGATTTTGTTATTGGTTTTGTTTAAGACCCAATTATTTAAATATTGACGCTGTTGTTCTGGGTCTTTCTTGAA

ATTCAATTTTTCCATGGAAGATTTCAAGTATTTTTTGGAGTTTTCCACAAAACTATCTTTAACATTGAAAGCAGTATCAA

CAAACATTCCAGTCGCCAGTTTCAGATTATCATTTTCACTTATTAAGTCTTCCAATAAATTCCTATAAGCTTCCAGAGAA

TGAGTAGTTTTATTTAAATGAATAGTGGCTATCATTTCGTCAAAGGTTTTTGAAGCTGCTCCAACACTAGCCATAAACAT

GATGACATGGATACTGAAAGGAGAATAGAAAATATTTCCTTTTTCAGTTTTGGCCAATTCCTTGTACAAAGAAAATGAAA

AATCATGATTTGCCAAGCTTAACGCTTCTAAATTTGTAGCGAGTGCCATATTTTTTGGATCTTAGAAACGGTTTTTTTTT

TCAAAACCGCGTACGAAGTTACTGTCCGGCAAGTGGCGATATATCGGTTTATTTTTCCAACGTTCGTGATAGATAGTGCT

TCGAAGACTCGGGAGAACAGACTGCGGGCGATTGCGACGATCGCGTGAATATATTCCTACCTATTTATTATTTATGATGT

TTTACAATAATAGTACTTTGGGTCAGGCGGGACGCAGTCAACAACAACAAACTGAAATAAACCGGCG

>contig_3956

CTCGCCGTCGCAGCCGTTGCGCTGCTGTTGGCCGCCTCTTGCTGCTGCGGCCGGTCGGCGGCGACCGACGCAGACCGCGA

CGACGGGGCGCTGTACCTGTCGCCCATGATACGCGACGGCCGAATTGACGAGGCGCGAGCCGCGTGCAAAGTGCACCTGC

AGACGAACAACGGCGCAGTGGCGGCGGTGGACAGTTGCGCCGGTTACCTGACCGTGGACGAGGCGCACGATTCCAACTTG

TTCTTTTGGTTCTTCCCGGCCGCGAACCGGACGTCCGGCGACGCACCGGTCGTGCTGTGGCTCCAGGGAGGGCCGGGCGC

GCCCAGCCTGTACTCCGTGTTCAACGAGCACGGCCCGTTCGCCGTGGACGCGGCCGGCGGTCTGCTGCCGCGCCGGTACG

CGTGGACGTCCACGCATTCAATGCTGTACGTGGACAACCCGGTGGGCGTGGGGTACAGCTTCACCGGCGACGAGGAAGGC

TACAGCGGCAACCAGACGGCCGTGGCCAGGAACATGTACGCCGCCCTGGTGCAGTTCTTCGCGCTGTTCCCCGAGTACCG

GGACAACGAGTTCTACGCGGCCGGCGAGTCGTACGCGGGCAAGTACGTGCCGGCCGTGTCGTACGCCATCCACCAGAACA

ACCCCGGGGCCCAGGTCAAGATAAACCTCAAGGGACTGGCCATCGGCAACGGACTTATCGACCCCGTAAACCAGATGGTG

TACAGTGATTTCTTGTTCCAGTACGGACTCATCGACGAGGACGGCAAACGGTTGTTCAAAGAACAGGAGGACTTGGCCCG

AGATCGAATCGCCTCGGGCGATTACAGGGCCGCGTACGACGCGATGACCAAGATGATGATCACCACTCCGTCGCTGTACC

GGCAGCTGACCGGCATGCGGAACATCTACAACGTGGTGTGGAACAAGAACCCGATACCTTACGAGGGCGGCCTCTGGGAC

CGGTACGTGCAACGTCAGGGACGAGCTGCGTTGCACGTGGGCCGGCGCCCGTGGTCGTCGGTGGACGTGGTTTACGACCG

TTTGAAGTACGACATACCACTGTCCGTAGCCCCGTGGCTCGCCGAGCTCCTGGACGCCGGCCGGTACCGCGTGCTCCTCT

ACTCCGGACAGTTGGACGCGATCGTGCCGTATCGCGGCACGGTGAACGTGGCCAGGGCGCTCCGGTGGACAGGAGCCGAG

CAGTTTAGGAACGCGACGCGATCCATCTGGAGGCCCGACTGTTCTCCACAGTGCTCCGTCTCCGAAGTGGCCGGCTACGT

GACCGCGTACGGCCCGCTCACCGTGCTGTTGGTCAGGAACGCCGGTCACATGGTACCGTACGACCAACCTGCTTGGGCCC

TCGACATGATCGACCGATTTACATCTGGCAAACCGTTTCATTGAAACTCCGCAGGTCCGTGACGCATCCCCTTCCTACCA

TAAAATTAAACAGTTACAATATTATATAGCTGTAGTATCGAATTCATACTTATATTCAAATAGTCATTAATCGTAACTTG

TTTTATGGATGTTTGACAATTTTTTTTTTTTGAAAATTTGTTTATAAATGCTATTCTTAATTTAAGATTTGCTGTAGCTT

AAGATTTTCATTGAGGCGAGACTGGCGCTCTCAAAAGTTCTCGATTGTATAGTTATAATCTATTTATAGCCATTACTGGT

GAAGATTTGGGCCGTCATCTAACAGTATGCAAGACTTTTGTGCGTGATAAACTTCTTTCGCTATTTCCAGAATATATTAT

AACAAAATATAAATCTAAGTTTGTATAATAATATAAATATATAAATTTAATGTAACCATGTAGGTGGGTCATATCACATT

CTCAAAATTGTGTAATGACAACATAGTACATTAAAAAACTTTTTTATACACTTATTGTGTATTCAATTAACATATTTCAC

ATGCTGTAAACATTTATGTATTATTATTACCACTTATTGTATAATGTTATATTACAGTACCTGTAAAAAATGCGTAAGTT

ATACCTATTTATGTATCAGACTCCAAGATATTTTTAATGAGTATCATTATTGCGTAATATTTAAAATTAAATTTAAATGT

GAATTATAAATTATAAATTTATGTATAATAATATATATAGTGACCTCTGCAGGTGTCTAAACCACTCTACGTCCAAGTAA

AACGTAATGAACGTTGCTCAGTATCACGTTTGAACTTAAAGAGCGGTGTGTCGAAGATATACAGAGTGAACGTAGTTGTA

GCGTATAAATATTAAGTATAATAATTATTTGTTTATTTAATAAGCTAAAATAATAGTATAATAATTTATACTTGTAAAAT

TTTTGGACAGAGTGTAAGAACTCTACCCGAGATATTAGTTGAGCTTTAAGACTTGGGATCCGAACTTATATATAAGGGGT

GGTATGAAGACGTGGCTCAACTACTCAACTAAGTCTAGTGTTTTGGGTGATCACACTAATTATTTTGATTTGACAGCGTA

TCCGTGTCCTGTATTTATACGAAATATTGGCTGGCAAATACACGGTAGAGAGGTTTATAGATGGTGGATCAAAAAGGAGT

CGAAAATGTGTTATAACGCCTTTCGGTTTTCGGCATGTTGATTATAATTAATAGTATTCTAACTAATAATGTTTTACTAA

GCGCATTATAATGTTGTTTACCGATTTTGCATGGATGAATAAATAAATGTGTACGTTTCTAGTGTCGTATAGATGGCAGC

CTGACTACATCTCTCCATTCTAAAATAGAAATCTTCGACTTCCTTTAAAACAAAGTTTTTAGTCTTGGGTAGCAATTTGG

GAAATGACTTGGTGCAAGTGGAAATTCGACTATATCAATAACTCTTGATGTTGTGACTTTAATTTCTTCTTCCTCTTGTT

GTTATGAGCTTTTTCTTCCTCGTCTTTTGTGCTGGATTTTGTATAATTAGCTGTAATATTTTGGTTAGATAGGCTATTAT

GGTAATTAATACTAATAAACTAATTAATAAGTATAATGCGTAGTCATTTACGCTTTGTTTATATTGTAAGTCGTTGATTT

TGGTATTTTCCTTGCTTGTTTTGTCCAAGTGTTCTTTGAGTTTGCAATAGTGTTCAAATCTTCCATGTTGTTTGTCTTTA

TGTGATAATCTTCTATTAGATTTATTGTTGATCATAGTACTTCCAATTTTCTTCTTGCTTAATTTTTGATTTTGGAATGA

AGTCTAAATAGTCCATCTTGTATAGTCGGTTTTCCTGATATAAGTACATCTCGAGTTGCATAGTCTTTGCATGTCTCGTT

TAAGGTTATTATCCTTACTCCTTCTAAGTTCTAATACATGACCAGTTAACTCCTTGTATTCATCACACTATGTAATTATT

GATCACCAATTCAGTTGTGGTAGTATAAATATTTCGGGTTTTATTTCGGTAGTACCCGGAATTGTTAACAGGTTTTGATT

TTTATAGTCCATGATGACCTTGTGTTTTTTCAGTTTAAGTATAGGGAACAAT

>contig_41158

CTACAATGAAGATTAGAACTAACCTGTATTTAATCACGGCGATACTGCTAACATCAAACTTATTGACACTAGACTCAGGC

GACAGTGATATTGATCATGACCCATTCCCTGACCACTTAGATGATCATTTTGATCACTTGAATGATTTACATGATATGGT

GGGTCCTATACTAGATAATGCCAAAGTCATTCATAGAGTTGAATTAGTTGAACATAATGGAGATATATTGACTAAATATG

CAACAACATCTCATACAAATCAAGAGACAAAGAAAGAAATATTTCCTAACACGATTACTACCATCCAACAATCTAATGTA

CCAACCAGTTTTACAAAAGTAATAAAAAAAACGGTAATAAAACAATCTAATAATGATGGAAATAGTTCCCCAGAAACCGA

TTTATCGATGAATGATATGTACAGTACAAATAAATTGAAAGAAAACGGAATTAGTAATTCAATGTCAAAGTCTCAAACTT

TATCTCAAATGGATCACGTGAAAAACATTGGTTTGCCACCTTTACCTTTAAAAAAAACTTCTGATCTACAAGGTAATATA

AAAAATATAATACATACTACAGTTACAAAAACAACTACAAGTGCAGGAAGTCCGTCAAATGAATTAAACGAATCTTTTAA

TAGAAAACATAGTTTGGACGGACTGTCTTCAAATAATATCATATCCACAACGAGTTCTACAAAATCAAATTTCAATGATG

GAACTGTAATTTCAGGTTCCTTACAATCGAATTCAATGGAAGGAGGTAGTAATATTAAAAAAATCTCAATGACAGAAGAT

ATGATCAATGGGAATGGTATAAATAAATCTCAAAGAATATCTCGTACACCAGTAGGTTCCATCAGACGTCCAAAAACTAC

AATAATAAAACAACATAGTGTAAATGACGACAATGATAGTGAAGATTTAGATAAATCTAATATGGAATTTAATACAGAAA

ATATATCTTCAAATAATATTGCATCATCTATGACAAGTCCTTTAGGGTTTAATACTATGACTACTACAACCATTGACAGG

GATAATTCTAATAATAATGCATTTAATCAAGGAATCCCATCCAGAAGAGAAAAGACCCGTTTTCACAAATTTAATGGAAA

>contig_41556

TCTGGTCGAACATACTAAACTGCAATATCGTTACATATACTGTGTGTATATATTATATATATATAACGTGTGTATGCGTA

TTTTTTTTTATTTTTTTTTTATTAAATTTTAACGCAATACCGCACACGATATTATAGATTTTTTTTACACCTGGACCGCA

CCGGGCACCTGACGGACGAAACGACGTTTTCCTGTAAAATCTCGTGCGTTTTTATCGTTTTTTTTTTTTGTTTTTCGATT

TTTCGTTTCGCTCGTAATACAATATTAATATTAACTTTATTGTTATAATTATTTTCACGAAGACTGTAACGATATTATAA

TATTACATATCTCTACACATTGATTATTATTGTAATCGTTATTCGCCGCCCGACATCGACGTCCGGACAACAGCAACTTC

GTGAAGCCATAAATAGAAACAATGGTACGAAATTAGGTTGTGTTTCGTGATATCGATATCTCTATTGATTTATTTGCTGT

TCATTCGCTAATTTATTTGCAATCCACTACACGGCCAGACTGGGGTGGACGATATGGATCAAAATCAACGAAATTATTAC

AAATCAAACATAGCTGCTTTGAACTTGACCGCAGCACTGGTGAAAAGTGAACCGGTCGACGAAAAAGAGGATGATGCGAA

ATGGCCCTGTCTCGAAACGGCCAGCGGCAAAGGATTGTCCACGGAGACGTCTTCCACAACCTCCAGAGGCGAAACGTCTA

ATACATCTAATCACATAAGGAAACCGCTGTGTACCATAGGAAACTCATTCTACATATTCTCAGCATTAGTATTATTCCTC

GTGGGCAGTATAACAATAGTATACACACCATTTAACATATTAATGAATGAGAGACTGAAAATGGTTCGTGGTCTGCCAGC

ATATGAATGGTGGAAGAATCCTCCTGATGAAGTTTTGCTTAGAGTGTACCTATTTAATGTAACAAACAGTGAACGATTTG

AAAACGGAATCGATAGTAAACTGGAGCTAAACGAAATCGGCCCAATAGTATTCAGAGAATTATTACGACACAGCG

>contig_4215

GTGCGAACCACTTTCATCTCTCTCCCTATCGTTCGTGGCGGTGGTGTCTTCAGCTTTATTATATTTCGTTTTTCCTCTCC

ATCGTCGCGTTTGTCCTCCTCTGCTGTCCGACGCGACTGTCCATCGTCGTCGAACACGACCGCCTTCTTGCGCCGTCAGT

TACTGCTCATTATTATTTCCGCGACAACGATGAAGCGCAACACATGTCTTTGGGTGGTTCTCACCACTTACCTGGTTCAA

CAATGCTCCTGTCAGGCACAACTCAAGTCGTCCAACGTACAGGAAGATCCGTGCAAAGCCAAAAACAAGGTGGTAGCCGA

TGCGGCTTACTGCGATCGTTATTGGGAATGCGTGGACGGTCAGCCTGAACTGTACGACTGTCCGAACGGTCTAGTTTATG

CGGGCAAAAACAGGGGCGTCACCGAGGGATGCGACTACCCGTGGAGGGCGGACTACTGCGAAGGCAAAACTCAAGCCAAC

GGTCCGATAGCCCGAGAACACTGCGATTGGCTGTACGGAATATTCGGTCATGAGACGTCTTGCACACGATACTGGACTTG

CTGGAACAGCACCGCCACCGAGCAGCTGTGCATCGGCGGATTGTTGTACAACGAGAAAACTCACTCTTGCGACTGGCCCG

AAAACGTCGACGGATGCCAGAAACACCCTCTCTGCAACGAAGACGCCAACGGAAACGTGCCACTGGGCAAGTCGTGCAAC

AGGTATTGGCAGTGCCAGGGAGGTTACCCGAGGCTGCAGAGGTGTCCGGCCATGTTGGTGTTCGACAGGCGCACGCTGAG

GTGCGTCGTGCCGCCCACCGAGGATTGCGAAGTGCCGACCACACCGGCCCCGTCACCGGACGACTTGCCGTCCAACGAGG

ACGACGGCGAAGAGAACGTACCGCAACAAGCTAAGGCCACCGCCGCCCCGGCACCGCAGCAACAGCGAAGCCAGCAACAG

CAGCAACAGCAACCGCAACAACTTCATCAGCGACAGGGCGGTCAGCCGCAACAGAGACAGCAGTTCCAACAGCAACAGCA

ACAGCCGCAACAGCAACAGTTGCCCGCTGCCACGCAACAACAGCAACAACAGCAGTTGCCGCAACAACAACAGTTCCAGC

AGCAACAACGGGCTCAGTTCCAGCAACCCGCGGCTAGAATTGTTCAACAACCGCAACAGCAACAGCAACAGTTTGTGCCA

CAACAGCGGGCACAACCACAACAGTTCCAATTGCCCCCGTTGCCGGTTCAACAACAACAACAACAACAACAACAACAACA

ACAACACACCGTGCAGAGGAGAGAAAACACTCCGGTATCGTACGGCGGCGCCGAAGACGATGTCGGCGACGACGACGACA

ACTTACCGGTCGCGTTTTCCGAACACTAAACACAATCATTGCACTGTACAACTAACGTCGATACACCTACACACACACCT

ACACATTTAGTATATTATTAGAATATATATATTATAGTTCAAAACAAAAACGAGTATTACCTACGTTTAGTACATCGAAA

ATATAATCCGATTCGCGATTCCTCTGTCAGAAATGATTTTATTCGTATACTTAACTACCTACAACAATAGGTCGATTTTT

CGAATTTATTTTCCGCTATAGGCGATTTTTCGAGGGTAGTCGCGCAAACGTATTAGATACCATGCATAAAGACCGCCATA

AAAATTACACGTCAATCGTCGTATCGAGTTTTGTCGAGATATCGCGTATTCAAAGATTACGACTTTTCTCAGTTTCTGTA

TCTTCGTTTGCGGTCGGTAAACTACGTTTTAAGGTCGTATCAATAGGTTCTTTTTTTCATTTGCGTCGGTAAAAACTCAA

TAACAAAAATAAGCCAAAACCCGTCGAACAAAACGAATTCAATAAACGAGATACGTGTGCGTCTGTCATCCTCGTAGAAT

CACTTGATATAAAATATTCATAATACGATATAATAATAGTATACTTTTAAGAGTGTAAAGTTTAGATGTTATTCGTGTTG

ATACAAATTTGAAATTGCTTGCCAAAGGACTGAGTTTATAACGCATTTTATTCTCTAATGATAAACAATAACTTAATAAG

TTGAAATGTTTGTACAACTATTTCCGTCGAATATAATTAATATACATTGTATTATATATACTCGTATATATATAAAAAAT

ATATAATACCATGTATGTATATTGTCGTACAAAATTTTTTACAATTTTTATTTTATTTTTTTTATATAAATATTTCTATT

GTTATAGAGATTATAATATTATAGTTACATTATTGTTTATTATTAAGATTTATAATATTGCCATTTAAAGGTAAGTTTTA

ATATTTAGTATAATACATAAAATAAAATATAATATTGTACCCTGAGCACTTATATACCATAATATAGTGTTGACATAAAT

TTAAGAAACTATAACATTGTAATGTGTGTATTATTGATTACAATATTTGCAATTAAAAAACGAATGCG

>contig_4343

GTTTTTTTTTAATTAAAAAAGATTTATTAAAAATGTTTAAAAAGTACAAAATATTTTTGGATAATATTTATAAATATTAT

ATTCTGACAAAAAGTTACAATAGCAAAATAATTCTGACAGTTAATTTATTCATAGGTCATGTATACCTATCATCAATTTT

TTCAATGCACCTACCCAAATTATAAATTATTTGATAAATTTTTCTAGACATAAAAATGAGTTAAAATTAATATGAAATCA

TTTTATTATACACTGTACATATTTTCTATTTTAATAAATAATAATAGTAAAAACATAAGTCGAATCTTATGTTAAATTAA

TAAGTAACACATTCTTCATAATAACTTATGTACATAATATGGTAACGATGAAATTGTTGCCGATGTTTGATTTTTAGATT

GAGCAAATGAGTGTGCACGTAGAAAATAATCAAAAATATATTTTTCTAAGCATAAAGATGTACCTATCTATTAATAACAA

TGGTGAAAAACTAAGTAATAAATGCCCTATTAATTTTAAGATTTGTCAGAAACAAAAGATCATGTATTTATATTATTTAC

ATATTAATTAATTATACTAAATTGACAATCGACATTAAATTCTTTTGTTCCGAGTCATAATAATACTATCATGATACTAA

TAATTAAGTCTATTATCATTAGATATTTGGCTTTAATTTATGATTGAATATAAAATAAATTTCAAAAACAAACGTATAGG

TATGCAGTATTATATAATTATAAAATTATAAAATAAGAGCACCTTTATTGTTTCATTGATTTAATTTAAATCAATAGTTT

GTATATTCTTCAATGAATAAATAAAATAACTATATCACGGTTTGGTAAGTATAGCTCGACAACATTATTTCTATCCAAAT

TTCAATTCAATTGTAGTTCATTTCAATTAAATTTAAATATGAGTTGAAAAACATTCAATATTTTATTCTGCCCCAAAACC

AAGGCGTACAATATTATGTATCTATTGATAAATTACGTTTGATTTTTTTTCGTTTCTTATTTTATTTTCTCTCTATATAT

ATATATATTAAAAAAAAATGTCTTCATCTATAAAATTCGTATCACAGTCCAATTTGAGAATTTGTTTGTTTCGACGTTGA

CACGGACATTAAACATTTGTGTACTTTTCGTTATCTATATCACAGTTTAGACTACACAATAGTTTATTAGCATATCGTTC

GTATCTCTTATACGAAAAGGTGATGGTAGAATTTTCCAAATTTCAAAATCATTAAAGGTACGTTATAACAAGTAGTCTTT

AGATCACGATAGGAGCTGTTGTTCGTCCACGTCGGTTTCCTGTACGGTGTTGCTTATCCGATGGTACGATTCACGAACGA

TTAATAATCGGTGTTGGCCATTTACCAGCATACTGCTGCCTCGTCGTAGGATACTTTGCCTGCCACGTTCGATGTTTTCC

CGGGAAAATACAATTTGGTGATATGTGCGGACAAACACTGTAACCAGTACTAATGCGCCGCCAAGAATCAGCCCGTATGT

GAACAACGGCACAGCTGTGTCTACGAAGGTGGTGGCCATGTATATCCAGCGGTGAATGTTTTCTGGCAAGTCGCTTACGC

CCTCTTCAAGCCACATAATCGGGAATGTAATGCTTCTGAAGTTGGACGTTGGGTAAATATTGGATTGTTCAACTTTTAAA

TTGAGCTGGACTTTCACACGGGCTTCCAATGGTACGCCCAGTCTAGGTTGAATTTTTAAGAATGTTTCATGCTTTTCTTG

ATCTGGTGAAAGACCCTCTACATCTTCTAAAAGTTTTGGATCAGCTTTGTAGAAATGAGGAAAAGATAAGTAAACTGGAG

CATTAAATTGACAAGGTCCAATGTACTGCAACCCTTTAACTGTGCATTCAGTGTTGTCTTCGCAAAAACATGCATTATCA

GGATTTTTTTCAACAGTTTCTAGAAGATTGTCATCAGGTGTATAGTACCCTGCAGTTATACCATCTTTGATCACATCTCT

TCGGTACCTAAGTGCCCATACTCGACATAAATCTTTGTCATACACATGTACTATATCAGACTTTGTAAATTCTCTTGGAG

GGAAAAACGAACCTTCAGATGCTCTAATAGAATTGCAAGATCCCTTCCAGAATGGTAATTCTTCCATTCCATTTATACGA

TTGAGAAGTCCAAAATTTTCCATTCCGTTATGACCCGTGTATATTGTGGAAACTTCATCTAGTGTACCATTTCTCATTAA

GAAAAGTCCCATTTTTTTGGGTGGTCTTTTCCCTTTTGGATAATATGTATTTGCTAGAGAAGTTAATGGATCGTCATAAC

CAAAAACTAATTGTTGTGGGGTAACATGTTTAAACGGGGTCAAACCAGAAAACCTTAAAATTGCCGAAAGTGAACTTTTT

AATATTGATGGTAAACTATTTGCTTGAGTAGTCAATGTTAATAAAGGAATATTTGGTACAGTTAACTTGATTTCATCATT

TTTGATAATTGAAAGATGTGGCATGAATTCTAAAATTTTTTTGTGTTGGTACGTCACCGTACCGTTGTTATTAAAATGAA

TGTTAACTTTTTCCATGTTTTCTCTATAAACAAATGGACCAATTTCTTGAAGTTTTGGCTTTTCACCATTATGCAAAAAG

CCATCGGGATTGGTGACATTGAATATGAATACTTTTGTCAGTCTTAATACGCCAGGTTTTTGCCAGTAATGATAACTTAA

AGTTCCATTCCATATTTTCAAGTTCTTTAAAATTATATAATGAACCCACGGAACTGAAGAGAGTATTAGTCCAGCTATTA

AAGCTACAACGCCAAATATGATGACCGCTAGTCGACTGTTGCTGATCCTCCTCTGTTGGGAGACGAGCATATTGATAGGT

AACCCATTTCGTCTGCTGGTTCGTCGGTTCGATTGACGTTCGTTACTATATGGGTCGTTTATGTTGAAAGTCGGTATTTG

CTGAAAGCTGGCTGACATCCGGGGTTGTATACCAAACAGACGATTTTTCGCTGACTGGTATGGTAACTTGATCGGCGTTA

AGCCCATTTTTGTTGCTTATTATCAACGGTGGAAATCGTTGATCGCGTCCAAAATATGTGATATGTTCGTACGATAATCT

CGCATCCCATTCGCTGTGCACTCGGGCAGCCACCTCAAGTGTGTCCGATA

>contig_4515

ATTTAATTTTTTAATGTTTAATAAAAATGTTCATATTGTATGTATTTTAAAAAAAAAATTCGTCAGAGGACATAATAAAT

TATTATAGTATATAATGTACGTGCGCTATTATGCGTATATATATTACCATTATCTCTAGCATATAATAATATAGTATATT

GTGTTGGCGTTTAAGCAACACATATTAATAGTAATGTATATTATATATTATGATAAAATAGTACAAAAATAATAGTTATA

ATAATAATAAGAAGAGTGTTTGGAATAATAAAAATAAATGAAGTAATGTACAAAAATCTTATAAATATAAAAAATATAGT

AGAGAGAAAAGAAACAAACAATATCTATATATACCTACATATCGTATTATAAGTTACAAAAATGAAAATAAAATGTACAA

TAATATAATATATATATATTTATAAATAAATAAACAAATAAATAATAATTTACAGTGATAAATTATGGCGAAATTATATT

CTGATCACTATGATTATGCGTTTTAATCGGACGAGTAGGTATTTACAATACTCATACACGTCATCGATATTATGATTATC

ACATGCGGTAGTGGGAAATCGTCGAAAATAATCGTATCAATGATGAGAATAATAGTACAAATCGTCGGCGAGGTAAAAAG

ATACGTTTGTCAGCTGTTACGATATTACAATCTAGTGAATTTATGATATATTGTATAATATATTATTAATAGCTGTGTGT

CGGTATTTTTCATATTCGATCTGTTTCCCGTCACCCTACCTCACGATTTGTTTTCTCTGCATCCTCGGGTCGAAAGATCG

ACCACCATAGTTCCTCTATTCGTCTACTCGCTGAACATTTGATTCCGCCGTTATGTGCCTTTTGTGGTCGTCGAAGGAAT

TTCTACACACCAACCCACATACACCCACGCACATATTAAATAAAGATTCAACGTCGTGTTGTACGACGTTCCATCACTCG

TTATTAGCTGTAACGAAATACTAGAAACGAATTATACGCACTTGATTGTATTGTAATGGAATCAATTTTTGAAAGTAAGT

AATACATCATATCGATAATACTATTGATATTGTGTTTGAACACACACGAAATAATTAAAATAAACACGTTTTTAATATAT

GTATCATTAGCGTAATGTTTGAGACAGGATTTAGAAGACGATAATGTTTTTGAAGTATCATAAAAACATTATCGTTTCCA

AACGCGGTCGGTAAACAGTACAGTTTATTTATCACTCTGTCTGTATTTATTTTTTTTAATTATTATTCACAGTCTATTAT

TCTAATGATATATTTCAATAATCTGAATGAATTAAAAAACAAAAATTAAAAAATGATTTACTAGGTAGGTGTACTAAAAA

TAATTTTGTTTTTAAATTAAGAAACAGATCGTAGATAAAGTTAATCGTGTATTTCGTTTTGAAACGCATTCAGATTGAGT

TGATAAAATATCTATACTGTAATTTCTCTGTTTTCGATATTATATTTATGATAAGTTATTTTATAGTGACTTTTTACCTA

TAGTGTACAGAGTAACCTGATCCCATTACTTTTATCGAGTAACTTGCCCGACATTGAACGAATAATGCGTGAACACGCGG

TGGGGATTGACTTATTAATCGATAATTAATATTATTATTATTTTAAGATATAGATGCGCGATTACAATATGTGGTCTGCT

GACATATGAGGAGGGTTCCGGGATCTATTTTTTATTCTACATAATATAATAATTACTATTATATCGTACCTTATAACGGT

GCGACCATAAAATCATAACAACAACAACTATAACAACAAAAAACAATAACAATAATAGTATATTGACGATTCGGCGATGA

CGTGTCTACTGTACGCCCGTGATGGTTTGCAACCACGGTTTGTAGTACGTGGTCCTCATGTAGACGCCCGGGAGATATGG

TGCGGCGCACTTGATGCCGTGTGACACTGTGCCGATCAAAGTCCAGCGACCGTTATCCTTTTCGATCATCAAAGGCCCGC

CACTGTCACCCTCGCAAGAATCTCTCTGCCCGTTGGCGTAACCGGCGCACAAGAAGCTACTAAGTATGGTCTTCGTGTGT

CCCGCTGTCTCAAACATGTCCTGACATACTTGGTTTTCAATTATAGGAACTTGCACTTCTTGCAGGATGCTCGGAACTCC

GCCTCCGTATTTCAACCGTCCCCATCCTGTGACCACGGCCATGCGTCCCGTGAAGTCGTCCTCGTCATCTGGCATGCAGA

TGGGCACGATATGCTGGTCGTAGTTGACCGGCGACTCGAGCTCCAGAAGGGCTATGTCGTTCTCGAAAGTCGCCGCGTCG

TACTGCCGATGGACGATGACGCGTTTCACGTTCTTGCTGATGCTCCGCTTGGACTCGACCTCGCCGCTAATGTCATACTC

ACCGAACACGGCCACCAGGTTCGCCAAAAATCCGGGTTGACAGTGGGCGGCGGTAATGACGTGTCGTTGAGTGATCAAAA

CGCCTCCGCATTTGTTCTTGGTGAACAGTCCCAGCCAGGTGGCTTCCCTGACTAACACTTGCCACGGCCATTCGCCAAAC

GTCGAACCCGTTCCGCCGACGATCCGACCGCCTTTCCTCACCAAAGGCCGCACTCCGCACGTTTTCCTGTAGTCCGGTTC

GGCGGTGGTGTGATGGTCGTGTTCGACGTTCAGCGTGTCCAGTATGACGGTCGTGGAAGGCGTCAGCTTGACGGGCAACT

TAGTCGGCCGCTTGTTGTCCGTGGCGTACGTGGTGGGCTGGAAAAGCTGGACGGGCGTGAGCAAGGTCGGATACTTGACC

GGACGAGACACGGTGGTGGTCACCTCCAGGAACGGGACGGAGTCCAGCGGCGGAGTGATGGGCGTCGGCCGGTACGTGGA

CGTGGTGGTCCACTTGACGGGTTGCGGCGACGGTTTCGTGGGCTTGGCGGTAGTGGCCGG

>contig_45158

ATCTGTTAAAGACATTTAGTTAACTGCAAAAATCACTTCCACTCAAGACAAACACCATAATACTCTCATCGCACATCGTC

ACAGACTCGATACACGCCGATAAAGGTACACTTGCGTCAAGGGCATCCCTAACAGCATAGTATCTCGCGAGACCGTTCGC

GTATCGCGTATTCCGAAAACGATAATCGTTGATCGTTGACCGGTCGTGAGGTATAACGCCTGCACCGCGGACGCGCGTTC

CTCATCTGTCCGCGTAAAATACGCACTGTGCGCCCGCGTGTTTGAAAGTTCGACTTCGCCGCATATTACAACCGCTGCAG

CACGGCCGCGGGTTACAGACGCATAAAATCCTGTTCAGAGTTTATCGTACACGCATACCTACATCACCGTCATTCTACCG

ATATAATATTGCTTACACATTATATTATTTACATATGAGATGAAAACGGAAGAAAAATGAAACCACAGTGCTCTACCATC

GTGTTGTTTTACTACTGCTGTTGTACTATCGCGGTCCTGTGTCTGACGGGAGCTGTGTCACAGCGACACGAATATGAACA

GGACGACGCTACCGCCGACGTCCATCCTTGCCAGAGAGAATGTCGCGTGGACGAACCGCCAAAAACGTGCGAGTACCATT

TCAAAGTGGAATG

>contig_4861

CTCTCTCTCTCTCTCTCTCTCCGTCGTCTCTATCCGTCTTTGGAGCGTTTTTCAGTCGGAAACAGGGGTGGGAGGAACGC

CGCCAACCCAATAGCGACGACATCAGTGGTAACCGACTGTCGCGGTCGGATCTTTGAGTTATTTTGTACTCGCGTGGAGC

GAACATATTTATTTCACGCGCACAAGTCGCCGTCGTCCTCTCCGATTTTCGCGTAAAAACCGTGGCTCGCAAGGCCCGCG

AAGTTTGACGCCAGTCCGGAGACGAAAATACGATTTACCATGTTGGAACCCAACCTTGTCAACGTCGTGCTTATCCTGAC

GGCGATTTTGATATCGTGTGCGATATGGTCGCGACTCCGGAAGCCGCTGGAATACCGGCAGATCTCGTCGCATGTGCCGT

CCCTGACTAAAAACCTCTGGAACGAGATGCTGTTTTCGTGCACCATGGGGATGAAACACCCCAAAGATTTATTGCCGTTC

TTCAAAGAAATATTTGAAAAAAACGGCCCAGTAGTTCATGCCAATATCACTGGACGGTCATATGTGTTACTAAATGATCC

GGATGACATAAAAACTCTGTTATCCAGTACTGCGTACATAAACAAGGGTCCAGAGTATGAAATGTTGAAACCGTGGCTAA

ACGACGGACTTTTATTAAGCAAAGGTTCAAAATGGCATAACCGTCGAAAGCTCCTGACCAACACTTTTCACTTTAAAACA

TTAGACATGTACAATCCGGCCGTCAATAAGCATGCAAGAGTATTTGCGAAAAACCTCTTGGACGCGTGTGCGGACAATAA

AGAAATTTCAATTTCGGAATACGTGACGTTATGTTCTTTAGACATAATCTGTGAAACAATTATGGGTACTGAAATGAATG

CACAAAAAGGGAAATCAGTTCAATACGTCCATTCCATTAAAAGTGCTTGCAGGTCAGTGATTGATCGAGTTTTCAAATTT

TGGCTTTGGAATGATCTAATTTATAGAATAAGCAATAGTGGTCGATCGTTTTTTACATCAATCAAAGTGTTGCATGAATT

TACAGATAATGTCATCAAACGTAAACAATTGTTGTTAAAAAACTCTGAAAATCAAAAAGTGCAGCCTGAAATCAAACCTG

AAAAAAATCGACCAAAATCATTCTTGGATTTACTTCTTGACGTTTTGAACGAAAATCCAGATCAAATGACTATCAAAGAC

ATTCGAGAAGAAGTAGACACGTTTCTTTTCGAAGGACACGACACATCTTCCATAGCGATAACGATGACAATTTTATTGTT

GGGGTTGCACCAAGATATTCAAGATCATGCTAGGGAAGAGCTATATAGTATATTTGGCGACTCCGACAGAGACGCTACAA

TGGATGATTTAAATGCCATGAGATATTTAGACGCAGTCATTAAAGAAACACTCCGACTATATCCGAGTGTACCAAGTTTT

ACGAGGGAATTGAATACAACTTTACAACTCAAGAACTATACCATACCTCCAATGACAACAATGGCTATATTTCCCTACGT

TTTACATCGAAATGAAAACATTTATCCAAAACCCGAAGAATTTATTCCTGAAAGGTTTTTAGACGAAGAAAATAAATCAA

AATTTCTATTTAGATACATACCGTTCAGCGCAGGCGCAAGAAATTGCATTGGACAAAAGTATGCTATGAATCAAATGAAA

ACAGTAATATCGACAGTATTAAGAAATGCAAGGATTGTATCTTCGGGATGCAAAGAAGATATTAAAATTAGTATGCAATT

ACTTATCAGAATAGAGTCACTTCCAAAAGTAACATTTCATAAACTATAGCTAATTATATTTTATACAGTATTATATAATT

AATAGTAATTTGATATTTGTTTTTTTGTGATGAATGTAACGGCCATAGAAATTTTTTGTAAACCTAAATTTATTCATACA

GAATACAGAAACATAATAATTTAAGATCTTCGTCTAAAAGTTACCGAGGATTAAATTATTTGTTTAGTTTTTAAGTATTT

GATATTTAATTTTGTTAGTAATCATAATGGATTACATAAAATAAATTGTTGTTTCTGTGTTATAACAAACTTGTTATTTA

TTATGTATTTTCCTTTTTTTTTTAATACTAAATTATTTTAAGTACAGTTTATAAATCATATAATTATGAGAGCTCTGAGC

TTTGGTCTATCAAACACGATAACCTACACACTAAATTTTGAACCAAAGAACTTACAAGTATCTAAAACCAACATTTATAA

TGTCATTTATTCAAAATGTAATTTAATGGTTTGTTGTTTATACACTTGTACCAATATACTAA

>contig_4871

CTGCGTTTAAGCACGAAGTCAGACGTACGCGCACGGTCCCCGGGCTCTGCACCCCGGCGGTCGACTACCGGCAGAGCAAC

ACCCCCGCGACGCCGTCGCGTCACTGTGCTTTCACACCGACTTCCGCGCGCACAAAGCACGCACGTCATATCGTACACAT

ATACCCACACACTCACCCACATCACTCGCGGGCCCATTATCGTCGGGCGATATATACGCGAGAGTCCGCTCTCCGCACGC

CAGTCGCGCAGAGCACCTATAACCATCATACATAATATTCTCCTCCACCGATTCCTTTTTTTTTACTAAATTTTTATTTA

CTTTTTTTTTTTTTTTCGAGCGCGCAATACACCCCCCCGGTAGCTTCAGCAGCAGTATAATACGCGCCGCGCCGCACATC

GCCGGGCGACTCCGTCAGAGACGCACGTCGTCGTCGTCGTCGTTGTCGCCGTCACAGTCACCGTCGCCGCCGCCGCCGCC

GCCGCCGCCGACCGCCTAACCACCCTCAACCGTCGCCGACACACGGGTTTAATGCTTTTGGACAAGAGTTATCATTAAAT

AGTATGCAGACTACAGAATCGTCTTCTAAAAAGTGAATTATTAGACCGGTCAACTTCAATTTAGCTTTAGGCGATATATG

GTCTTGGACAGAGGAATGTTTGAAGAATGGCTCAAACGATGTCGCGAATCTCGCCAACTCGTAGTGGTGATCGTAGCTAT

AGCTTTACTCCTGGACAATATGCTTCTTACTACTGTGGTCCCAATAATACCGGAATTCCTCTACGACATCCAGCACCCCG

ATCAACCACTAACTGCTACCATCTCATCTGGCCCGCCGAATGAATATCTGGTACCACATGGAGCTATGACAGCGAACGGG

ACAGTGCCGACCTGGGAACAACGAGAAGCGACTCTCAAAAAGGAAAGGCACGACGATCTGGTTCACGAAACGGTCGCAGT

CGGTATGATGTTCGCATCTAAAGCAGTCGTGCAGCTAATGGTTAACCCCATTGTTGGACCTCTCACCCACAAAATCGGCT

ATAGCATACCGATGTTCACCGGATTTTTCATCATGTTCATATCGACTATAATATTTGCTTTCGGACGGAGTTACGGGGTA

CTGTTTTTGGCTAGAGCATTGCAAGGGATCGGATCTTCTTGTTCAACTGTTTCTGGAATGGGAATGTTAGCTGAACGGTA

CCCTGATGATAAAGAACGTGGAAACGCAATGGGTATTGCATTGGGTGGATTAGCCTTGGGCGTGTTAATCGGCCCACCGT

TTGGTGGAATTATGTACGAGTTTGTGGGGAAAACTGCTCCGTTTTTAATACTATCAGCCTTGGCACTAGGAGACGGAT

>contig_4891

CGTGTTAATTTCGCATTATTCAAATATATCGATATAATATTAATACGCAGTGATACATGGCTCTGGGCGCTTTCGATTAT

GTCGTGCTTACGGTCACGTTACTCGCGTCCGCCGCGATCGGAGTGTATTACCGGCTCACCGGTGGCAAACAGCAAACGAC

ACAGGAATATATGCTGGGAAACAAGAAATTGTCCATTATACCTGTAGGATTTTCGTTAATGGCTAGTTTTATGTGCGCCA

CAGCCATGTTTGGGTTAAGCGCTGAAAACTATTTGAGAGGAACACAATTCATGACCATTAATGCATCCAATATTATCGGT

ACACCTATTGTCGCCTATGTTTTTCTACCAGTATTTTATAAGCTCGGATATTTATCCGTTTATCAGTATTTGGAAGAACG

TTTCGGAAAGTCGACTAGAATCGTTGCGTCTTTGGCTTTTAGTATCCAAACGGTACTGCGTACTGCACTAGTACTGTACG

CGGCCTCATTGGCTTTGAATGCCATCGCTGGATTTTCACCGACCGCGTCCATGATGGTCGTTGGCATGTTGTGTACTTTT

TATTCTACCGTCGGAGGCATCAAAGCCGTAATCGTCACCGATTTGTTTCAGTCGTTGCTCATGTTCGGGTCTGTTTTTGC

GGTCATTGGAGTGGCAGCGGTCAACGTCGGTGGACTGACAGAGATCTGGAGGATTGCCTACGATTACGGTCGTGTGGAAC

TGTTAAACTTTCAGATCGACCCGACGGTCAGGCACAGCTGGTGGTCACTGATGCTGGGTGGAATGTTCACATACGTCTCG

GTGTACGGTGTCAACCAGGTGCAAGTGCAGCGTTACCTGACCATGAAGGACTATAAAACCGCGGTCAGGACCCTGTGGTT

CAGCTGGCCGGTAACCGCTTTCATGTCGGTGTCCATGTGCTTCGCAGGACTGGCCATATTCTCCAAGTACCGAGACTGCG

ATCCGATCAAGGAAGGTAGAATAACAAGTGGCGATCAGCTAATGCCCCTGTTCGTGTTGGACACGATGGCCAATATTCCC

GGATTAACTGGTTTGTTTTTGGCGGGTGTATGTTCATCCGCATTGTGTTCCGTGTCCGCAGCTCTCAACTCCTTAGCAGC

CGTTACACTTGAGGATTATATTACACCACTGGCAAACGTCGACATACCCGATGAGAAACGTGTGTTGTGGCTCAAAGTCA

TTGTCGTCGTCTACGGGATCCTATCAATAGTGTTAGCGTTTTGTGCGCATTTTGTGGGACCGCTGCTGCAGGCGTCAATG

ACGATTTTGGGCATTATCGGAGGACCCATGTTGGCGGTGTTTACTGTCGGCATACTTGTACCGTATGTAAACCAAAAGGG

TGCTATGGTGGGTCTCGTTGTGGGTCTCATCTTTTCATTTGTCTTGGGACTAGGAGGACCCAAACCACCAGTGAACAATT

TGCCCACCTACACCAACGGATGTTCGCCGGACTCGTTTAGTAACTTTGATGTAAACGTCTCGTCTACTACTCAACTGCTA

GCGTCCACGATATCACAAGTGGTTCATGAAGAAAGTTACATGTATTTGTATAAGATAAGTTACATGTACTATATAGTCAT

TGGTTTCGCGACTACCGTATCATTGAGTTTGGTGGCAAGCTTGTTCTTCGAATCCAATGTCGAATCACTAAATCCGAAAT

TATTTTTTTCGGCTATTCCAGAAAGTGTAGAAAGACAAGTTGAAACAAGACAAAAAAGACATTCGACTGTTTCAAAAACC

GTCACGTTTTCCATTAACTCGTGAAAATTATCGTTGTTTATTATTACTGTTACTAATATTAATAGTTTAATTTTTGATAA

TATGTATTTTTGTTTGATTTTAATCATTGTAACTACGAAAGTGAAGTATTTATTTATATGTATTTGTTGAGTATGATGTA

TCTATGTGTGTTTTCTATTTT

>contig_49128

CTGTTACCTTGATCGTTGGCGATGACCTCTACTTTTCCGTGTTGCCAGATACCCACGCAAGAATAGGTGGTACCCAGGTC

GATACCGATCGCCGTCTTTCCAACCATTGCTTCGTCGAAAACTTGTAGATTTAGTTCACGCACAATAAATACGAATAGAA

ATTATAAAATTATATAACTCTAAAACTTATAGACGGTAATAGCGCACAACTCGAAAACGATTTAAAAGTCACTTTGTTTT

GAATATATCGCACTGGAATTATCGTGGATAGCGCAGAGTAGCACAAGACAGTATGCCTCGCTCGTTGAATCGCACTGGTA

TCGCTCGAAATTCAAACTGATGCCCGC

>contig_4951

ATAATATATAAAAAAAAATCTTAAATTGTTCAGAAAACACAAAAAACTTAAGATATCTGAAAAAAAATTGAATTACAAAT

TGTATGTTATAGATTAATATAGTTTAAGAAATGGTATACATGTTTCCATAATCACATTAAGAAATATAATTAAACTGTAT

GTACCTTAAGGTCGTGGTCATGTGCCGTTTATGGTTCAAATTACAATATTCATAATCGAAAATAACGTTTTCTTATAAAT

AAGTAGCTTTAATATGCATAAAAATTAAAATTAAACGATTAAATGCTTTACAATAGATTATATATTATATTGAGTCACAA

AAATTTGATATATTTGAAATACTTATAAATTTAATTATAGAAATATGTTCTATTTTACCTTGCTTGAAGTATGTCAGTTA

AATAGTGTAAATATGCTGTAATTATTTGTTATTCTAAACATATAGTATGTTATCAATAAAGTAAAAAGAAGACGTAGATA

CAAAATAGAAATATATTCAATATTCATAAATTATAACCATAAAATAAACCTCAATCTATCAATAATATATTAGAAAATGT

TGGTTTTTGAAGATATATTATATTATAATGTTTAGTTCAAAAGAGCGATGAAATAAGAATAATGGTACTCGAGAATAACA

TTGCACAATATTTATATGAATTTATTAAGTGAACAAATCATTTGGTTGTATAATATAGCTACAATTGTACATAAATATAA

AATTTGAAAATACTTACAGCGTATGAATTGTTTAGAATAAAATTTATAAATGTTCTAAAATCCAATCAGTAAATGAAGCC

ACTTTGGTATACACGCCCGGGTATACTCCACATTTTTTACCAAAAGAAACAATACCCATCAAATAAAATTGTTTTTCTTT

CAACCACATTAATGGACCTCCGGAATCACCCTGACATGAGTCTTTCCCTGTTTCTCCAGCGCATATAACTTTGTCATCGA

TCGAATAATGTTCACCTTCATATAACTTTTTACAGTCCGTCATGTTTGTAATTGGAATTCGGACTTCCTTTAAAGATGAA

GATGATTGACCTCTGAATTCAGTAGCTCCCCATCCAGTAATAAACGGCAAATTATTCCCCATATCGATGTTTTTCATTTT

TGGCGTTAGTGGCAAACATATTGGTTGGATAAATATGTTAAAATTAACTTTATTTTTCAATACTAGTAATGCAATATCGT

TGGTTGAAGTGCGCGCGTTATATTCTTCATGTTTTATAATACGTTCGATTGGAACATCTAATGGGGTTGCCCCATCATCA

ATTGTGGGATCTAAATTCAATTCCCCTAAGCGTGCAACTGTCAATATTCTATTCCCAATGTTGTGTACGCATGTAGAGCC

AGTTAAGACATGTGTGTTAGAAATTAATGTACCACCACATAGCCACTGGATAGAACCATTGATATTATTAACATTTTTGT

ATCCGAGAACTACCATCCAAGGCCAAGCACCTAATTCAGAGTCACTTCCTCCCATTATTCGAAAACTTGGTGCCATTCTT

TTACCACATGTAGCTTGTGATGGCAATATAGTTTCAAGATTTAATGACTTATTCGGTATGTTAGGTGTATCAATAAATAC

AATCTTGTCTTTAGGAAGTGCAGTTATAATTCCGACTATAAGGGACGTGATTATAAAATATTGTTCTTTTCCCATGTTGA

TAACTGAACTCAACAC

>contig_5042

TTCACAATTTAAAAACATAGATTTTATTTATAATATTCCGTAATTTAATAAACAGGGTAAAACACATTACACAAAATATT

TTTTTTATAATAATATAATGATAATGAAACCGAAATAATTATACATTCTTACACTAACAATCAATAACGGACATTTTTTA

AATTACAATATTCTTAAAATTATTTAATGTTAAATAATACATTTTGATCAATCATAAAACACTAAAGTGACAATTAACAG

AGTATTGGTGCTACATCGGTAACATCGATGTATAATTATGTCTTATGCGATTAAATTTTCGTTTTACTTCATAATGACGG

CAATAATAATACAGTTTACCAAAATGGAATAACATCTTCCATGATGCGTCGAAATCGTCGTATTTTATCGTAAAATATCT

GTCAAATAATATGGTTGGCAACACTAGATAATTTGTATTTTTTTGATAAATACAAACCTCCGAGACTTCAGCTCGGTTTG

AAAATAATCGATTTACGTTGCGTTTCTGTCGAAAATTATAAATCAAAAATACGTCTGTTCGATTAAAATATGTATGTATT

GAAGACCAGGAGACACAATTAACAATAAATAATAATACATAACCTATACAATTATAATCAAATCGCCTTTATATATTGTC

TCGTTTTATTATTATCTGAACATCCGAAGGGGCGGGATTCGATCGGAATTATATCAATCGCAATTAATTAATTAATAATA

GTATGTACACAATGTAATTATAATATTAAAGTAAATTTCGCAGCTCCGTTCAATTTTATAATATAGTATTATATTATTAT

ATTACGCGAACGTAGCGCGACCCTAAATAAAATGGCATCATCATTATTCATTACAGTATACCAGACTTTTGGCGTATACC

GCATATCGTCTGGTATAAATTTTATGGTACCTATCGTTATAGGCTAATTTTACAACATGGTTTAGCAATAAATTATCGTC

GGGTAGGTGCAATGTCCAGTATAATATTGACTGCTGATCATTTGAACAGCAGCAGAGAGTTATTGTTGTTGAATGCGTAC

GGCAAGAATTCCGGAACTCTGACTATCCTCATGATACGGATGTTTACTTCCCTCTTGTCCAGTGTTTTTTTGAAGAATCG

TTGTAAACGCGTCGATATCACCCAAATTGCGCCTTGGTCGCGATCAGCCGATCTGATATCCAATACGAACTGTAGTTCCT

CCGGGCTGTATGCCAACACACCGTGTTCCACGGATCCCGGAATCCAAGTGGCTAAGGCCGTTTCGGACACCGGACTGAAT

ATGAGAGCTCCGTCCTTGGGATCGACAGCCAACGGGGCAGCTTGAGAAGATTTGTGGCCTACCAGCGACACGGGAAGGTC

GGCATCATCACCCAAATTTGGTCCAGCCTTGAGTGCAGATGTGGGTACGGAGAACAGACGATCGGAAGCGAACGGCTGGA

AGTACAGGCGGCGACCTACGGATCCGAAAGGCGATAGAGCTAGGCCGATAACGCCATCCATCAGGGTGAAAGATTCGCCA

GCTACCGAAAAGGTTCCGAAGTCTGGATCTGGAAACATGTAAGGATGCGAGAGTCTCCACGCTGTGTCTCGTCGTGCGTC

GTATACCAAAATGCCTGGGTTTGTGCTGTCGGTCATGTACACAAATACGTTATCACAGTCACCGAGGAATCCGTCTTGCA

GATGGGATACTTCGACCTGATCATCGATCACCAGATTTGTCAAAAGAGTGTTGGGCCTCAATATTTCAGCAGGCAGTGTC

ACGCTTCTCACCACTCGATCTGTTCTCATATCGAATACCAACAACTTGGGCGGGCAGACCGTCTTGAATGTCTCTATGCT

GTCCATGACTCCACTGTCCAGCACCCACAGCCTATTACACCTGTCTGCCCTCACCCGAAACACGGATATCAGGCTGGAAC

AGTTGGGCGTCGACGTCGTCAGTATATCGCCGCTGGACGCTTCGCTGTGCCATTCCCAGTTAGGATAAGCCTGCAACAAT

GGCGATTTATGCGTGTCATAATTCACGCCAGCTCGATTGCGTGGCACCCATGCCAGCGTGGCCGGATTACCATTGAACAG

TCTCGGGGTCGTGATGAATATTCTGTCCCATCCAATTTCAATTCCGGTGGATATCGTTACTTCGGGCTTGTAAGAGTCGG

CGTTGGGGTAATTAAACGGCACATCGTATTGGAGCAACGTCCATTCGTTGACGACCTGTAAGTTGCCGGAACCGGTGACA

ATTGTCACAGCGCACGCTAACAGTACCGCTGTTGCGGAGTTCATGTTTGAAAAACTTATCGAAATTCGATCACTTCGCGA

TCTATAACTCTGAACGGTCGAGAATAATTTACGAGACCAGAGTACCCGATCGGTGCAAGTCTGCGGCGAAACTGAC

>contig_5272

CATATTTTTGGTTTCAGTTGAAATATTATTTATCCACTGATTGAATGATATTATTTTTTTGATGAATTATGTATATTCAT

ACTTAATGTTACACAAAATAAATATTTCAATATTTTATCTTTGATTTTGTCTAATAATTATTATCTATAAAATTTCAATT

TGTATTTATTTAGTAAGAATTATTAAATCGCATATAATTTTAAGTATTATGGTGTTTGAAATAAAAGGAACATTGTGAGT

CGTAACAATATTATGTCCTTTGTATAGCAAAATCAATTCGTGAAGGTGTCTGTCAAGTATTACACTGATGGACAACTCAA

TAGTTTAAAGACGAGTGTACTTCCACGTGAATAAGAATAATAAGACATACATCTAGTGAAAACAACTATAACAAGTATAC

AATGTGTGACATGTCGATCAATATCTAAAAAAGATGTTCTATTTAATTGCATCCGTTGACGTAATAGTCTACAGAGATCT

ATAAAACGGCTGTGTTTGCTGTAAATAAAACACCTCTCCTATTAGCAATAAACGTATACGAACAAAATTAGAAACTATTC

ACACTAATCAATAACACTTAAACTGACCATTCCGCATGATGTTCTTTTGTTACAAGTCGTGCGAAAAAAACATATACTAT

GAGTACTGTATAAAATCATTAGGAACAAAAAAAAATGTCTAAAAACTGTGGTTGTACAACCGACCGACTTGATAATGATA

TTTTGAATGGTTATGTCGAGTGTTGCGCGTTTGTTTTGTCCGTTAAAATTGCAATATCTGATTGATCCAGTCTTTGAACT

TAGAAATTCTAGTGTATACACCGGGTTGATTTGGTTCAGCACATCCAATACCCCATGATATAATACCGGCTAGTAACCAT

CTCTTGTCTGGTCTTTGGATTACCATAGGACCACCGGAATCACCTTCGCACGAATCGAAACCTCCTTTTTTCCAACCAGC

GCAGATAAATATATCTGGTATGTGTTCAATGTATCCAGCTGCACGGTACATAGTTTCGCAAACAGAATTGTTGATCACAG

GAACTGTCACTTCTTGTAGAACACTAGGAAGCGGCCCGTCTTCATATAATCTCCCCCATCCGGTGACGTAGGCAGAAGAA

CCTACAAAATTTGAATCATCTTCAGGAACGCAAACAGGTATGATATTTGGTTGAAACTTAACGGGTTCGTAGAATCGCAA

AAGGGCCAAGTCATATTCAAAAGTTCTGGGATCAAACTGTGGATGTGATGCGACTATTTGTATTCTTCGTTCTTCGTAAC

CGTAAGGTTCTTCTTCTACTGACAAATCGTGTTCACCGAGTCTGAGGAGTAAATCACTCGGAGGAACGTTTTCAACACAA

TGCGCAGCAGTTACAGCCCAGTTTTCGTTGAATAATGCCGCCCCACATTTATGTAAGTATGTAGACGTCCTCCATTGTCT

TAACGAAATCTGCCATGGCCATTTGCCAAATGAAACTTTTTCACCTCCTACTATTCTAGCAGTCGGGAAAAGTCGTCTTC

CACAAACATCTTTGTAGTTGGACATATTCAGGGGAGCATTTCCGGCTTCGATGGAATCCGTCGAAGTCGACTGCGTAAGC

ATTGACAATGTACTTGAAGGATTTTGCGTGCTACTGTATGTCGTATCGACACTGTTTGAAGTCATAACGATGTCATTATC

GGATGGACTAGGTAGGGTCGTCGGCTTGGGCATCGTCATCGTCGACGAGTATGATGCACTAGAGCTGCTCTCTTTCGGTG

GTGTGCCGACTGTGTTTGGTTCATGTGTCTCGACTGGGAGTTTGACCCAAGAAGGAAGTGTCGAACCGTCGATAACCGAT

GGCTTATGCGTCATGGTTGGTGGTGGCGGTGGTGGTTTTGTGTTTTCGGCCGGTACAACGGGAACTTTGTCGAGAGTGGT

CCAAGTGACCAGAGTCGGCGAGGTAGACGTGACGGTCGTCATGCTGCCGTAGCTCGGTGTCTGACTCTGACCGTTCGTGG

TTGCCGCAGTGAAATTGGCGATGATCTCAGTGACGTTTTCGACCGTTGAGCGAGTCGCGTACGTGGATCCGGTCGCGGAT

TCGACAGTGGATCCGTACGCTTCCGGTTTTCCGGTGGTAGGTTTTGGTTTGACCGTAGTCGCGGTGGGTTTGGCCGTGGT

TTGCGGTTTGACCTTTTGCGCAGACGCGACGGTCGTCGACGACGGGGTCGTGGACGCTACCGTCGATTTGTACGTGGTGC

TCGACGGTTTCATGACGGGTTTGGTAGTAGAGAACGTGGCTGTGACGGTACTGTTATGCTGCGGGCCGGGACGCATGGAC

GTCATCGTGGTGTACGGTTGACCGGTCGTGGCGACGGGTTTCGTAGTAGGTTCGTGTGAAAAGCTGGCCGTTGAGCTGGG

AACGCTGAACTGGACGGTCGGTCTTGCGGTCTGAGGCTTTGGCTTTTGGACGGCGGCCGTTGTAACAGAAGCCGTGGCCG

TGGCGGTAGTGGCTTGGTCCCTGATAGGCGGCGGTTTGAACGATGTCGGCTTGACCGAAGGCTTGGCAGTGGAAAAAGTG

GTCGACGGCATCGGTTTCGTCGATTGTGATATCGTCGTCGAGTACGAAGACGATATCGAACCGGTGGGCGGCGAATTGCT

GATGCTGTTGCTGCTCGCGTACGATGGCTTTGTGCCGCTCGACACCGATATCTCTGTGTATTGGGTGCGCGACGTGGACG

TGGACGTGGTGGTGGACGGCGTGGACGACGACACGGCGAAATGGCCGGTGGGCATG

>contig_5354

AGCCTTGGCACTAGGAGACGGATTGCTACAACTACTGCTTTTACAACCAGGAGTAGTTACAACTGATGCAGACCCCCCTT

CCTTAAAATCTTTAGTCATGGATCCCTATATAATAATTGCTGCAGGCGCTATTACTTTTGCTAACACCGGAATCGCAATG

CTTGAACCTTCTTTACCTATATGGATGATGGATACTATGGGTGCCGGAAGATGGAAACAGGGAGTAACATTTTTACCAGC

GAGCATTTCTTATTTGATCGGAACGAATTTGTTTGGACCTCTCGGACACCGTATGGGAAGATGGTTAGCTGCAATGATTG

GATTGATCGTGATTGGGATTTGTTTGATGATAATACCAACGGCTAGAGACATAAATCACTTGATCGTACCGAACGCTGGT

CTGGGTTTTGCTATTGGTATGGTGGATTCGTCTATGATGCCCGAGCTTGGTTATTTGGTTGATATACGTCATACAGCTGT

ATATGGTAGTGTTTATGCTATTGGAGACGTGGCGTTTTGTTTGGGCTATACTATCGGACCGGCTTTAAGCGGAACATTGG

TAAACACAATTGGATTTGAATGGATGTTATTTGGAACGGCTATGTTGAATTTCTTGTATGCGCCACTTCTCTACTTCCTA

CGCAGCCCACCGACGAAAGAAGAAAAAAAATCTTTGGTCACCGGAGAAAAATCATCGGTGCGTTATATAACCTATCAAAA

CGAAGCGGAGGAAGAATAACGTGCATTATTAGCTTCGTGATACATAACTAACGCTTGATAAATTACGCTTTTTCATTTTT

TTTCTCGGCAAACATACTTTAATAATTGAGATCATAGTGACGTTGTCAGCACGTCACTGTAACAAATATTATACAATACA

GAGACTACAATAAACAGTTAATAGTTATATAATTTAACTTATAATATAAAACACTTTACATTTACATTTCGGTGGTCTAA

TTCGAAAAGATATTAATCACATTATACCTAGGCATATATAGCGGCTTTATTTATTTAATATTTATACATTATACATTTAT

ACTTATATTCATAATCGAAATGGGCCACCGATATAACGAGACCCCATAACTGATCTTGTCCGTACTTGTATGAAAAAATA

CAAAAACAAATCATATACAAAAATTAACTACATAATAATATATAATATTACCTATTATTATCAACAATAAAACTAAAATA

ACCGTTACGTGTACATTATCGTGTTATACATTCGATGACGAAACATACAATATACGATCGACTTTGCAACTTATTGAACG

TGGTTTAAAATATAAATAACACAATATATGAATATTTTCTTATACCTATTTATTATATTATACTTAAATCGTATTATTAT

GCATACGCATTTTATTGCTCACCATTTACATCATCATTGAGCATCAGAACATTTTGTTGATTAAGACTGTTTTATAATAA

TTTAAATAAATATATTAATCGTGTTTTCTTTATATATATAATAATAAAGTGTCAGACCAAACTAAACTATAATGTTACTT

GTATTTATTTATCACGCACGAGAACCTACCAATATAGCACACATCTAATACCTATTG

>contig_5415

GGCAATTTTGTTTTTGGGACAAATCGTAGTGTCTTACTTTTTTGCGTTTATCCTATCCATCGCATTCGAAGCACCGATCG

TGTCGCTGCTGAAACTCGTGTCGCCCACCAAACGGAACAACCACTGATCAATTATATTTCCTGTATTTCATTTCATCGAC

GTCTGTGCGACAAAACCTATTATTTTATTTTATTATTGATTATTGTATTATGTTACTCGTGACTCGTCCACCGACGCGAA

GCACTCGAAAATACGTTCTTCTCATCACGTCAACAGACTACAAATTCGCAAATTAAAATAATATTAATAATAGTACTTTA

TTACGTATTATAGTGGCGACTGGCGAGTGAACAGAGTATTTATAATTTAATTTATAATATGTTTATATGTTGATTATTGT

TTTAATAATATAACATATTATTATATTATATGTTAATAATTGTTGTTATGTTAATAAATAAATGATTTTTGTTTTAAGCA

ATTATATTTTTACGTACTTAACTAAATATTATTGATTCATGAAGTACCTATGTTGTAACTATTATTTACGAATTAGGTTA

TTACTATAATTTACATCTTTTTATATATACATTGTATTAATTTAATGTAACAAATTAATTATAATTTGATTTTGATAGAT

GTTAAAGTATATAATGATGCACTGCTTCCTAAATCATGAGATATTGTTAGATATAATTTCATTATGTATCTATTTGTAAC

ATTTAAAAAAATATACATAAAATGTTATTTAACGTTTTGAATTATTTTGATGAATCCTAACATAATACCATAATGAGATA

TTCTTTTTTTTTTTTTTTT

>contig_5436

GTGGGAGGTTTCGAGATGCTAATAATATTGCCTGATAAAATGGATGCAGTTAAAGATTTAGAAAACGGGTTCCTCAAGAA

TGCTAAAAATTACGCGTACTTGCTGAGCAACATGACCATCCACAATGTCGAGTTGGACATACCAAAATTTAAGTTTGAAT

CTGATTTGAATCTCGAAAAAACTATGGAAAAATTGGGTTGTACCGATATGTTCTCGTCCCGTGCCGATTTCTCCGAGCTC

AGCAAATCAGCTCCTGGCAAGCTAAGAGTCAGCAGCATCAAACATAAGGCTTTCGTAGACGTCAACGAAGATGGCACGGA

AGCTGCTGCCGTCACCGGTGCCAACGTCGTGAACTACAATCTGGAATACGTTTTGACTAATATCAAGACTGTTAAATTTC

ATGCGTGTCATCCTTTCTTGTTCATTATCAAGAAAGAAAAAGATATCATTTTCATGGGTAGACTGTCCAACCCGAACGCT

TAGATCGCAATATTGATATCACAATGCAATGACATGAATAACAAATAATAAGAGACTTGTATTCCTAGAAATAAGATTTT

TATGATTGATTTTACATCGATAGTTATGATGATGATTGTAAATTTTACAAAATATATATATATTTTAAATTGTCGTAATA

CAGAATGAGAAATAATACGAAAGTAGGTACAGTTTGTGTACCAAAACTATTATAATGTACATATTTTATTTTAATAATTT

TGAGACCATATCGCTGTTATATAATTTTGTTACTTTTTACGTTTTTATTCATATTTTGTGATTGCCATCATATATTGTTA

AGGTTATTACACATTACGCAGTTCTCAATGATTTGGAGATACCATATTACAAGCAAAAGCGAAGACGATATGTATTATGT

ATACACACATAAAACTGTGGAGTTGTTTTGACCACCAAATAAGTCAATTCCGTGCACATTTGAGCGTTAATTTTCGCTTA

AGGATGAAGAAGTGTTGATTTGACTACGCTATGTAGTTAACAGTGACCACTTGGTTTAACTTATCTATGTTAGTTAATAC

AACTAAAAGTTTCAGTTAAAATAACTTCATAAAAAATATCGTTACAACTACATCATTAATATTATCTTAACTATTTTAAG

GAGTCAAAATAAAT

>contig_5565

GTTACTAATAGCTGTTAGTATGAAAAGATCTATTGTAGATATTACAAACATAATTTTATATTATTTTAGTCATTAAAATA

CAATGGCAATATATGGTTTACAACAAAAAGATTTAAAATATTCTATTCATGTTTATGTCGGAAGGTAGAAAATTTGGTCA

ATAACCAGTGGTATTAATAAAATATCAAATAGGTGTGGCTTGGATTGGGATCATAGTACAGTACCGGTTTCTGTATACAT

TAAAGAGTTCCCTGTGGTCAAGAATTAATTTAGTTTATCCTCATTTAGTTGTTCGAAATGGATGGTTGTTACAGAGTGCT

TTACTGTGTGATTACACTTTTAGCTTTAGCAACATTAATAATATGTTTAGAGAATAATGATCAACATCGAGATGTCGAAG

AAAAAATAACTTATAATGGAGACCAAGTACTTAGAGTTGAAACAGTGAACTCTAAGCAAAGGAAAAAAATCAAGGAACTA

GAAAATCAAGGATTGATTGAAAAATGGTTTACCAATACTACATCAGTAGATATAATGGTAAAAAAAGACAATTCAGAAAT

TGTAAAAAACACACTGAAAAATGGATCATTATTATTTGATGTTTTTATTGATGACATACAAAGAGCAATCAATGAAGAAA

ATCCTCCGATAAATGAAGATGAATCAGAATTATCTGGAAGACAAGGACATAATTTGACATTTGAACGTTACCATAAGATA

CATGATATAAATAAATATATTGATTACTTATCTCAAGAATATCCAGATATAGTAGAAATTGAAACTATCGGTAAATCATA

TGAGAATGTCCCATTAAGAGTGATACGAATTAAGCCAGATCAAAATGCAACTGATGTAAAAGCTATATGGATTGATGGAG

GAATACATGCTCGAGAATGGATTGCAGTGTCTTCTGTGTTATATTTAATAAATGAATTGATATATAACAGAGATTCATTG

GAATCTCATATGAAAAATATTGAATTTCACATAATACCAATTTTAAATCCCGATGGTTACAAATATTCACATGAAAAGGA

ACGCCTATGGCGAAAAAACCGAAACAAGCAATCGGCGAATTCGTGTATAGGAGCAGATTTGAATCGTAACTGGGATTATC

ACTGGGGAGAAACTGGTGCTAGTAAATACTCATGTGCAGAAATATACAGAGGAGTAAAAGCTAGTTCTGAACGTGAAACC

CAAGCAGTGGTTCAGTATATTATGAAAAATCCAAATAAATTTAAAGGATTTCTGACATTCCATAGTTATGGACAATACAT

CTTATATCCATGGGGCTATGCCAAACGGGTACCATCAGATCACAAAGAAGTACATCGTGTAGGCCAGGCTATGGCTACAG

CTATCAAAAAGGCTACATCAAATGAGTATACTGTCGGAAACAGTGCAACACTTTTATACCCAGCAGCAGGT

>contig_5566

CACTTTTATACCCAGCAGCAGGTGCATCTGATGATTGGGCTAAAGGAGTTGCAAAAATTAAATATGCATACACTGTAGAA

TTGAAAGATACAGGAAAATATGGATTCATCTTACCACCATCTGAAATATTATCTACTGGAAAAGAAGCATTTGCAGCAGT

CTCAACACTAGCTAATGAAATAAGTTCAGAAAAGTAATAATGTGTAAAATTATAAGCTTACTTTTAGCTCAGCTGTACAT

TTTT

>contig_559

CGGTAACACGAAACACGTAGTACGTTCGGTATACAAATACTGGAATACGTGATTTACGAACAAGTGCATACGACCGCTGA

ATTAATACCGGCGGCCTAGTTGTTAGTTGCTCAAATTTCGATCCGTTCAAATCCGGTGCCAATTATTTGGTTTCCGACAT

CGTTTCGTGAACGGTTTCTAGCCGATCGTCATCCTAGTGTTGACATTTTTTCCTATTTAATTTTATTCCCGTTTCACCTC

ATCAAGCTATCATGAAGTTGATACCCGGATTGATCGTGTTCTCCGGATTCTTATTGAGCTCCATATTTGCCGACTCCGTT

GTCGATCTATCGGATTCTGACTTCGATTCGTCGGTAGCTGAATATGATACGTCTTTGGTCATGTTCTACGCCCCATGGTG

CGGACACTGTAAGAAATTGAAACCTGAATTCGAAAAGGCAGCCAAGAGTCTGCTAAAAGAGGATCCACCCGTAATATTAG

CAAAGGTGGATTGTACAGAAGCTGGTAAAGAAGTATGTAATAAATTTGGTGTTTCTGGTTATCCAACTCTAAAAATTTTC

CGAAACGGTGAAGTTTCTAAAGAATATAATGGACCCCGTGATTCTGCTGGCATTGTCAAGTACATGAAATCACAAGTTGG

GCCAAGTTCAAAAGATTTATCCTCAGAAGACATTATTAAGAACTTCTTATCTAAAGATGACGTGGTTGTTGTTGGATTCT

TTGAAACAGAAACTGATCTCAAGGGCAAATTTATTCAACTTGCTAACAAATTACGTGAAAAAGTAAACTTTGGTCATACC

ACTTCACAATCAGTTATTGACAAATATAATTATAAGAATAATGTTGTACTCTATAGACCTAAACATTTAAGCAACAAATT

TGAACCAGATTTTGTTATTTATGATGGTGAAGAAACAACTTTGGCTCTAGAAGCTTGGATTACATCAAATTATCATGGTT

TGGTTGGATACCGTCAAAAAGAAAACATGGGAGCTTTCAAACCTCCTTATGTTGGAGTCTATTATGCAGTTGACTATGTT

AAAAATCCAAAAGGAACAAACTACTGGCGTAATAGAGTTTTAAAAGTAGCCAAGTCTGTGAAAGATGTCACATTAGCTAT

AAACAACAAAGATGATTTCCAACATGAAATTAATGAGTATGGACTAGAGTTTGTATCAGATGACAAGCCTATAGTATTAG

CACGTTCTCTAGACAACAAGAAATATATAATGAAGGATGAATTTTCAGTTGACAATTTAGAAAAGTTTGTTAAGAATTTC

CAAAACGGTAACTTGGAACCATACATTAAATCAGAGGCAGTTCCTGAAGATAACACTACACCAGTAAAAGTAGCTGTTGC

CAAAAACTTTGATGATTTAGTTATTAACAATGGAGTTGACACATTAGTAGAATTCTATGCCCCGTGGTGTGGCCATTGTA

AAAGTTTAGCTCCAGTTTATGAACAAGTAGCTGAAAAGTTAAAAAATGAAGCTGTGTCTTTGGTTAAAATGGATGCAACT

GCGAATGATGTTCCTTCTACTTTTGATGTTCGTGGATTCCCAACCTTATATTGGTTGCCCAAAGATAACAAAAATAAGCC

TATTCGTTATGAGGGAGGTCGAGAAGTAAATGATTTTATCAAGTACATTGCATCTAAGGCCACAGATGAACTCAAAGGTT

TTGATAGATCTGGAAATCCCAAGGATGGCAAAGATGAACTGTAAACGGCAAATTCAGTTCTAAATTAAACCACAAGGCTG

ATTTAATAATTTTATTTGAATTGGACTACTCTTTTTTAAATCGAATACAGAGTAGAGAGGAGAAATAATATTTGAATCAA

TCCAAATTCAATACTAAACCAACTACTTTAAATTATTCTTGAAATAGATTATGCATATATACATTTTATAGATAATTCAT

GCTCCATTTTCCTAATCATATCTGTTGAAGTAAAAAGTTATTGTAATTAAACATGATTATTCTGTAACTAAAATTATAAT

TTTAATAAATATGTTGTGATTTTTCTATTTTGTTTAAAAAATTGT

>contig_57301

ATTATTCGCATCACGTTCAGACCAGCTATGACGCCCGCGTCCTTTGTCGCCTGTCTCTGTGAATCGTTAAAGTACGCCGG

CACCGTGATCACAGCATCCGTCACGTTACGGCCCAAGTACGCTTCGGCGGTCTCCTTCATTTTCGTCAATACCATTGAGC

TGATTTCTTCCGGCGCAAAAACTTTTCGTTCACCTTTGAATTCCACTTGGATTTTAGGCTTTCCACAGTCGTTCACCACT

TTGAA

>contig_5749

CTGACTTACTATTAATAATAATTAAACTTATAATAATAATAAATTTATACCAGAAAATTAGTATATTTTGTAAATGGAAA

GTTTTTCACTTATCTATTTATAATATAGCATAAACAAAAACAAATAGTTTTGTACTATTATTATTGCTACCTAAAATCAA

TGATTTTAAGTCATCTAATAAATTTAAAAAAAATTTAAAACTGCTTTAATACATCGTACAAAATACAAATCAATTTGTTA

AACACAAACAAATAATAATATAGGTACTCTTATTTATTTTATTTTATAATTTATTATACCAAATCAAAATTAGCTTATGG

TTGATAATAGATAATACGCTCCAAATATTAAAAAAAAAAATAGTAGTGATAATAAAAACGGTTTGACTTATAGTAAATAA

TTATGGAATTCGTAAATGTTTAATCTGTAAATAAGGCAAATTAATATGCTGGTAAACCAGCATTAATTTGTCCTTCAATA

CCACACTCGTCACTTCCTCGCAATATTTTGAAAAAACCATTACTACCCCAATCAGTGTTCCATGAATTAGCTATCAACCA

GTATGGTATATTACCATTTTTATCATCTTTTTGAACACCCCAACCAAGGATTCTAATTGCATGACCTCCGAGAGCCTTAC

CAGCAACATGTTTATACACTCCAGCTCTGTAATTTACAAAATCTTCATACACAGTAAATGCGCCTTCAACTGGACCATTT

CTATAAATTTCTTGTCTGATTTGGTCAACGTCATTACTAATCGAGTATGCAATTTTACCGCGGTGTAAATCTTTTTCATA

AGGCACTTTATATCCATCTTCACATTTTTTTACACATTTAGGAGTTTTACCTCCTTCCTTACATGGTCCTCGAGTACCAT

TCACGTGATGTTCACACGGAGCAATTTCGTATGGAATACATCCCAATTTAGATCCATATGGACCTCCGCTAACAATGCCT

TTATTTTTCCAATAGTTCCATGCCGCTCCTGGGAAGCCACCATTGCATCCAAAACCACAAGTCCAACAACAGGAAACTAA

ATTCTCAGCCGAAAAATAGAAATTTTTCGTTCCGTTTGAATGAATGCAGACACGGTCAGACATAGCTTCTACAGCTCCAA

AAGCCCAACAAGACCCGCACGAGCCTTGGTCCCTGACTTCTCTAATGGTGGGGCAATTCGGCCAGTGTTCCCTAGCGTCG

AAATTCTCAGGCAGATCAGTCACTGCGTCGGTGTAAGACAAAAGTTGTTCCAGTTTTGGATAATGCTTGTTCATCTCGTG

CACGCCCATCAATCCTTTGATATAAGAAAGCGAAGTGTCCTTATGAAAATTGCGGCCGGCGCTCCAATAATCTTGTATCG

AATTTATGTGATCGATGAAGTCATCCGACAACGGGTGCATGTCGTCGCGGATCGCATCGTCGCAGTGTCCGAAACCGAAA

ATTAAAACGCCCGCAAGCGTGAATATCATCGGCTTGAACATTTCGTCGTAAGTTTCGTCTGGAAACGGTTTTATACAGCT

GTTATACGCGGTTACTGAACTGCAGCAGCTGTACCGCGTGCAAAGCGACCTGTACTGGCGAGTGGCGGCAGAGGACTAAA

ACTAACTAAAATATGTTATTTAATCAATGCTCTATATTATGAATATTATAATATGAACGTAAAAACACGACGTGTTTATT

CTATAGAGGACTTTGATTGACGATAAAACGCAGGCGTACGTGCGCGTAGAGTCCGACGAGAGACTAAAGAGGGAAGCCGT

GTC

>contig_575

TTTAATTTTTTTTTATGTTTCATTAAAGAAACTTCATATATTAACATTATTAATCATTTGATAATTTTTATTTAAATATA

TACAAGTAAACATAAAAATATTTTTAATGTTTTTAATATTATTTATAAATGTGTCATTATAATATAATCTATTACTTACA

ATATATAAATTGTAAGTATTTAATCAATAATTCAATCTTATAGCGGGTTGGATTATATAAGGTATTTAGACGGTCAGCAC

TAGCTATTTTTAGAGTAGGTACAAGATATAGTTTTCAGTTATTGTATAATAATTGAAAAAAACACAATTAAGATTTTCTA

ATAAAATATTTCTTAAACTGTAAATGATAAATAATAGATATACATGTTGAACATGATATTCATAATAATTTGCGAATTCG

TTTGTTGACATAAAACATTCGTGTAACTCAAATCACAATATAATATACATGACAGACTGTGATGTTTTCCCTTTATACCG

CGTGATTACCATGCAGGATATTGGAGAAAATATCACCACCTCCCTGTATAATAATATACGATGTACATGCGTATACCTCC

CACCGAGTAATTCAATTGCCAATAAATTATGTGAACAAACTAGCAGTTTTGGAAGCCGCAATATGGTATACGCATCTCAT

TCAGTTGGATTCATATTTCCAACTTAAAAATAGCATCTTAAACACGTCACTGGTCCCAAAATCTTACTCGACCGAGAATA

TTAATTTAAGAAAATTAATCAACTTAAAAGACGGTCGTAAAAAGACTACCGCGATAGTATTATCTATAAATTATAATAGT

ATTATATATATTATATAATATTGTAATGCGGTGTCTACCGTAATGCACGTTGCTTGATGACCATATACATGATATTATCA

CAGAATAGATTACATTTGATCTACCTACTCTACGATGTTATATTATAATATACTATTATTGCAGTTGTCACGTTTTTAAG

ATGTACTTTTTTGTTCATTTGAAACGGTAACATTTTTTTCGAACCCACACATATTATTATCTAACAATTTAATATAAATC

AAAGAGAAAAAAAAACATTCTACACGAAAACGAATAAATAATTGGTAAAACCATAATATTTGAATATTAAACAATATAAT

ATATCAGCTGTATATACGATGGGTTTCGGATAACATCATCGTGGAATTTTCACGGCGCCTCTACCACCAGTTGCTCCTCT

TGTCTTTTGTATTGGCCAATTAGGTCCGGTAGATCGTAGGCAATGGCCATGTCCAGTTTGTTGATCGGACATCCTCGGAA

GTACGTACATGTATCAGACAACCGCTTAAACTCGTACACAGGATTAAGCCGGAAGAAATCTCGGTACACGTCCGGATCAG

GCAGGTCGTATCTCGATATGTTAGTCAGCGTGCTCAGGCCTTCGTAGATGTGGTAATTCTGTGGATTTTCAATGATATCG

TCGCTGACAGTCTTCCGGTTTGCGAAGAACGACTTGTGATTGTAATACGTGGTCAGGTAACAGTCGACCATCTTGGCGTG

GTTTCGCACCCTCACCGCAAATCGCCGAGCACTGGCTATTTTGTTTTCAACGCGCTTGTCGATTGCTGAACGGATGTCCT

TCAAGAATGATAGTTCTTGTGAATGAAGCAACCTGGCCGGAGACCCGATTTCGTACGGGTTTGACCACAATGACACAGAA

TAAATAACCGGAGGCTCGGCACTGGACATGAGCGGACTTATGTTCCATATGAGCGTTCCCTGTATACGCATCAGTTCTTC

AGCGCGAATTTTGTCAGCCTTGTTTAGCAATATCCTTGTCTGGTATTCCCTGCCTTTAAGCTGGTCCAATATTGCTTCAG

TTTCCGGGCCTACGTCTAATTTCGCCGGGTCGTACACTAAGAATATCATATCGGCCCTGTCGATGAACCACTGACACACA

TCGTTGAACGGGAATACCCGGTCCACGTGTTTACGCATTTCCAATATTCCGGGTATCTCTACGATGTTTACCTTCTCTAA

TAAAGGATGAGGTAATTTGAGTCCACGGAGCTTGTCCAATAGACCTTGTCCAAATTTCTGAAGACCCGAAAACGTCCAGT

CGGCTGCTAATTGCGTTCCGTCTAGAATCGCTTCTCGATTATTGTACATCATTATGTTAAAATACGCTGGAGATGGTTCC

GCTCCAGTTCTTAATGCAAATTGAGAATGTTCAATGTCCAAAAGATAATTAACTATTGAAGATTTACCACCACTCCAAGG

TCCCATGAACAGAATAAGTGGTTTAGAGAATATTTCTGGTTCTCCGAAATGTCTGTTACTCAGATCTCTGTACTTGAAAG

TCATTTCCAAAGGTTTGATTGCCACTTCATACACTTTTTTTAGCTCTTTTAATGTGGAATCTACAACTTTTGATAGAAAA

CTATCATACTGAGCAGTGTCTTCACCTATTTTTAACGTTTGTTTTATATGACTTTTATTTCTTAGATTCTTTGGTACCAC

TTCGTCCATTGTTAAATCTTCTTCTGGTAGGTATTCTTCAACTGTGACCTCATCTTCAGTAGTTTTAATCTTTTCGTTTT

TAGTATGCTTTTCCCATTTTGATGATTTTTCTTCTTCATCTAATTTCCAATACATAGTTCTTTGAGTTTCCATTAGTTTT

TCTTTTATTGGGTCTATAGGTTTTTCTTCTGTTTTCCAATATTTATTTTTTTTAGATGTTATATCTTCATCTTTCACTTC

TTTTTGTTTTTCTGTTTCTTCTTTTTTCCAATACTTATTTTTTTTTGCGATTGGTTCTTCTGGTGTTATTACTTCTTTGG

GTATATTTTCAACTTCTTCTGTTTTCCAATACTTATTCTTCTTTGGTTTCTCTTCTTCTTTTTCATCTACTTTTTTTGTA

TCCTCATTAGGTTCATTTGTTTTCCTATACTTATTCTGTTTTGATTTATCTTCTTCTTTATTTTCTATTAATTTTGTGTC

TTCAGCGTTTTCATCTATTTTCCAATATTTATTTTTTTTATGAGCTTCATTTTCTGTTTTAACTTCTTCAACTGTTTTGT

TAGGTTCATCGAGTTCATTGTCGGGATCGAATTTCCAGTATTTAGGGTTTCGCTTAACTTTAGCTACTGGCTCTTTTTTT

ATTACTACATCTTCATATTCATCTGGGTCAAATTTCCAATATTTCGGATTTCGTTTTTTGGTTCCTTTGACATTTTGATT

TAAATCTTGTTTTAATGTATTATCAATCGTTGAAGATTTTTCGGTGTTGTCTGTTTTTGTTTCTTCGTCTGGATCAAACT

TCCAGTACTTCGGATTTCTTTTACCTTTTTGGGGAACTGATTTTGTTTGTTCAGTCTTTTTAGCTTCCATTAACTTGTAT

AATTTTTCTTCTTCTGGATCGAGCTTCCAATATTTAGGATTACGTAATTTTGGAGTTTCAACTGGTTTGGGTGTTTCTTC

AAGTGGAAATTCATCCGGATCAATTTTCCAGTATTTAAGATTTCTTTTTGGTTTTGGTTTTATAATTGGTGCAGTATTTT

CAGGCTCATCGTCTGGATCAAACTTCCAATATTTAGGGTTCCTTTTTTTTGTTCCTTTAAATAACGGTTTTGGAATTTCT

ATTTCTATGTTACTCTTAGTAGTTTCTATTGTTTCTTGTACCTTAGTCACACTAGGTTCCACTATTTTATCAGAATTTTT

AATTTTGATATCTTCAACATCAATTATAATTTCAACGTTATTATTAACTACATCTTTGTCTAAAATAATAGGTACAATTT

CTTCTTTAGGTTCTATTACTTCATTTTCTACGACGTGTATACTATTATTTTCCAGATTTTCCTCCTCTTCACTAACGATT

TCAATCTCATTATCTTCCTCTTCACTTTTATATGCTTGAGTTTTTTCTGTTGTTTCATGTGAATTGATTTCTTCAGCTTC

GATTACTTCATTATTACGATTATCAATTTCATTTATTGCACTAATGTTTTCATTTAAATTTACTTCGTCTCTGGTTGTTT

CTTCTTCTTTTTCGAACTTTTCCGGTTCGGGTGGTGTACTCTGTCCGCTATCATTCGGAGAATTACCATCTTGAGAACTC

GAGCCTGTAACATCATCATTTGGAGTGCTTCCACTTGTGGGGATTTCATTCTGAATGTTTTGGTCGCGTTCTGTACTTCC

>contig_5822

ATCACACTTTCGACGGTTTCAGTACCAGAAGTAACTTCGGGAATAATTAAGATTTTTCTTAGTTTTTTCAAAACTTACAA

ATAACCCAAAACATAATTTGCACTATTTCGATCCGATATCGATTTCATTTGAAATATTTTTATTTTTTATTTTACAAGTT

AAATATTTCCAATCTAAAAAATGGCAAAATCGTTGCTGATCGTATGTGCTTTATTTTTGGCCGTCGTTGCTTCTGTAACC

GCTGCACCCACACCGAAAAAACAATTATCTTTCAATTTATGCAAGAAAAGTGATCCAAACCTTGAAAAATGTTTGAAGAC

CTCCATCCAATCAGTCATTCCAGATTTGGCCGAAGGATACCCGAAACTCAGGATTCCGGCTATCGAACCGTTCGAGCTAC

CGTCTTTGGAAATCGAACACGGAAAAGGCTCTAGCAAAGCTGTAAGCATTGACTTGAAGTTAAAGGATGTCAAGATCATG

GGTTTGACCAGCACAGTGATCGATTCCTTGAAAGTCGATGTGGACAACTACAAGATGAGCGGCAAGATAAGCTTCACTAA

GCCTCTCGAGATCACTGGACAATACACGGTTAACGGCAAGGTGCTCGTTTTACCGATCACCGGAAACGGACCGTGTACCT

TAGTCTTGCACGATCCCGTATTGGATGTTAAAGAAGTGTCGGGCACTCCATTTGAGAAGAATGGTAAGACCTTCGTTCAA

ATTAAGAAAGTCGATCTGAAGGTAGCGAGTGTCAAAAAACTGAATGTCAAGTTGGAGAACCTTTTCAACGGAAACAAACA

ATTAGGTGATAGCATGAACTCCATCCTGAACGAAAACTGGGAAGTTCTTCTTGATGAACTGAAACCAGCATTCGAAGAAG

CTATTGGAGCTATCGCACAGGATATTATCAACAAGGTATTTCAGAAGACCGCATACTCAGACATATTCCTTTTGTAATCG

GTTCGGTCCGTTCCTGATGGTTGATGTTTCCTAACAAAAAATTATGTTTTTTTAAATTTAATACTTGTTTCCGTCAGTAA

GCGCTAGAGTAGCTCGACAAAAATGTCTTAAGTGATCGACGCTAACGAGATGCTCACAAAAGCGCCCACTTTCTGGGGAC

TTGTTATTATTTTTTATAAAACGATGACTGTTACTTGTACGTCTCAGTACCTAATTGATGACGTGCTATTATTTATTTGA

AAAACCTAGTGTTTTTATTAAATTATTGAATATTATTTATATGAAACAAAATATATAAATTTATTAAATTATTATTCAAA

AAAAAAAAA

>contig_585

CGACGCGCTGTCGACGGTCCGCATGAATAGCATTAGTGTGTGTCCGTGATTCGACGGCGGCACTAGGCTATTTTTTATCG

GGATAATACTTGAACACTGTACAGGCAATTTTTTTTTCTTAATTGTATTTATTTTTTTAACGCACTCTTATCGACATTAA

ACAGTGATTTTTTTATCCCGTAAGTTGTTGAAGAAATGTATTATAACCTATGATCGTTACATAACCGACAATCGTCGTAA

GACAACGTTTGTAAATTTTGGTAGACGCGCCGTTGTTAGAATCGTATACTCGTAGTTATTATATCACTGACAACCGTTGA

AACATGAAGTGCGTCGGGCTGCTCCTGGTGGCCCTCGTGGGCGCGCTGTCCGTCGCCGAAGGAGCCAACATTCTCGGCGT

GTTCCCCATCAACGGCCGCAGCCATTGGGTGGTGTACGAGAGCGTGATGAAAGCCCTGGCGGCGCGCGGTCACAACGTGA

CGGTGATCACGTCGTTCCCGCAGAAAACACCGGTGGCCAACTACACGGACATCGACGTGTCGGCCACGTTCCCGCCCGCG

ATGAACACGGTGGGCATCGATTTGGTGCTCAAGTACCTGGCCAGCGTGTTCGCCAACCAATGGTTCATCGCCGACCATCA

GATGAACATTTGTCGGAAGCACCAAAAGCTGCCCCAGGTGCAAGCGTTGCTCCACAGTGACATCAAGTTCGACGCGGTTT

TCACAGAAATATTTGGAGCTGATTGCGATGTTGGTTTTGCATACCATTTCAAGGCACCCCTATTGTCCATCATGTCCAGC

TCACATCTGCCTTGGAGCTACGACCGTGTGGGTGGCCCAGACAACCCTTCTTACATACCAACGATTGTAACAAGAGCAGC

TGGAAAGATGAACTTTAAAGAACGAATGATCAACACTTTTTACTATATATACTTTAAATTAGCGTGGAAATATCACAGTG

AATGGCCAGCAAATGAACTGTTGAAAGAGAACTTTGGACCAGACGTACCTCATATCAACGAAATAATTTACAACACTTCC

ATGGTGTTTGTCAACGGACACTTTTCTTTGGATGGTCCACGTCCCCTTGTTCCCAACATGGTGGAAATTGGTGGAATACA

TGTTAAACCTCCTAGACCCTTACCCAAGGACATATTGAAATTTATCGATGACTCGCCAAATGGAGTTATGTTTTTCACAT

TTGGTTCTCTGATTCGCATATCTACACTACCGCCAAGTGTATTGCAAATGTTTAAAGAAGTGTTTGCGAAATTACCTATC

AGAGTCTTGTGGAAATATGAAGCAGAAATGAAAGACAAACCTGATAACGTGTATATAAGCAATTGGATGCCTCAAAGAGA

TATTTTGAGTCATCCAAAAGTTCGTATGTTCATGACCCACGGTGGACTGTTAGGGGTACTCGAAGCTGTACACTCGAGCG

TACCAATCATTGGAATACCATTTTTCTTCGATCAACCAAGAAATATATTAAAACTGGTAGAACAGGGTTCAGGTATTATA

TTGGACTATGAAACGTTGACCAAAGATGTTCTATACGATGCTATTATGAAAATAATTAACAATGACAGTTACGCTATAAA

TGCGAAAAAACTATCAAAACGGTTCAAAGACAGGCCATTGAACGCAACGGAAACTGCCGTCTACTGGACAGAGTACGTAA

TAAGACACAAAGGAGCGAAACACTTGAGGACCGCAGCCGTTGGAATGCCTTGGTGGAAGTACTACTTAGTTGATGTCATC

GGTTTTATCGCGCTAATCATTTTTTCTGTATTATATCTAATTTACTTTGTGCTCAAAACTATCTATAAAAAATTATTCAA

AAAGACCGAACCCAAAAAGAAGGAAAAGAAAAACTGAAACATTAAATTCGTATTGTACCTGAATTACTATACAAAACCAT

TTAAGCGTACCTATATTTAGTTTTATGATACGTTTTATTTCAACAGTGCAAGAATAGTTTCTATTAAAATTATACATTTA

TATAGGTATTTGGGAAAGAACAGATACGTATATTAAGTACACATACAAATTACAAATGCTTAAATTAAGGTACATGAATA

TCATTGTTTACTACTTCATTTAGTTTTATACTTATTTAATTTGAATTAAAATGATGTACTGTCACGAATAATTTTCATGT

TTAGTGATAAGTTACTTTACTATAGCACTGTAAAAAAATGTCATGTGCTTTCCATGTGCTTACCTAAATATTTTAATCGT

ATACACAACTGTTAGAATTATTAATAAAAAAAAGATAACTATTTATTTATATAATTTATGGTCTTAAAAATTAAAATACT

ATATTTTACTACAATGAGTTTTAATTTTATATACTTACTTATATTTAAATTTGAAATATATGGTGATATATGTATATTTA

GATATGGTCAATATTAAATTTATAAATACAATTTCTTAAATGTATACCTATGATTTAATGAAATCGATGATAAAGTCTGT

ACAAACAAAATCATATCAATACAGTTTTTTAATTTGAAATAAATAAGTACACATTCTAAAATTTGTGTTAATTAAAAAAT

AATTACTTTAAATAAAGAAACCCACTAATATCCATTAGATAACAAAATATAATTATTATTTAATTAGAACAGCGATAATC

TAGTCTCAGCCCTAGTAAACTAGTGCTATTAATATTATTATTCTAAACACTAACAGGTACAAACAGAAAAAACATAATAA

TTATCATTAATTAAAAAGTATATATAAACATAATTATTTTGATATTTTATTATTATAAAATCCCGGGATCAATAAACATT

ATGAATGTCATGTGCAAATAAAATAGTGATATTACTTCCAAGTAACAAATTGTTAGTATTTTTTTGTCTGTAAATTCG

>contig_5856

TTTTTTTTTTATCCATAGAATATTTAAAATGTATTTAGACTTGGTAACGCACAAATTGATGTATAATAAGTAATAACTGT

ATTTTGTAAATATTAATACTACAAAAAATAGCTTACATATGTATACATATTTCAAGGGCAGTAATTATAGAGTGCTTATT

GTGAATAAAAAAAATACAAGACCCTTATTAATTATTCAGTTATTAGGATCAAACCATGGGTGTTTAAGTATGCATATTGC

ACAATTAACTTTATAAATAATTACATAAGCACACATTTATTTTAGTAAATAATTTAAATATAAATCTACATCATATACAT

CACAGATTTATAAATAGTAAAATATCTTTTGTGGCACTTATGACTTGAAAGATATTCTTCTCCTCAGTATCCATCTAGAC

CGTTGTATAATTTATTGATCGAGTTTTCACCTAATATTCTTTTGAGATCTTCTTTATCTTGTTCAGAGAAGTCAGTTTTA

GGAAACTGGACGTCAATAGTGATGATGAGATAACCGTGTTGGTTATTATTTTCATAGTTAGGCATACCTTCACCCTTTTT

ACGAATTTTTGCTCCAGGCCATGTGACAGTATTTCTTTCTATAAGAATTTTTCGACCATCCAATTGGGTTATCTCTGTTT

GGAAGCCAATTAATGCGTCCTGTAAAGAAATAGTTAGATTAGTATATAAATCATCGCCTCTCCTTTCAAAAACTGAATGA

GGTGTTGTTTTTATTTTAAAAACAAGATCGCCTGGTTCTCCATCAATATGTGGTTCTCCTTCTGCTGTAAACTTAGTTTC

TTGGCCATCTTTCATACCAGGTTCTATTTCTATTTCTAACATACTTTCTTCGGTAACCATTTTTACATTGGGACATTCAT

CACAAACATTTTGTTGCATCATTTGAAACCTTCCAGGCCCTAATTGTTTAGTGATCATCTCTTGGCGACAATTACATTGT

CTTGTACCATGAGCTGGTTTTATAACAGGTTTGTTTCTTGTTACCTGCACAAAGTTACCATTGTATAATTCTTCTAAAGA

AACATGTAAGTCAAGAACTATAGTTCCACCTTTAGGAATTTCTTTTTGTCCAGGTGATTCATCAAAATGAAAACCAAAGT

CTCCAAAGAAACTGGCAAACGGATCATGACCAGCAGCCATGCTGTCCTTTTTGACACACTGTTCACCACACCTATCGTAG

TCTTTACGCATTCTTTCATCAGATAATACTTCATAGGCATTTCGTAACTTTGAAAATTTTTCTGAAGCTTCTGGGTCATC

TCGATTTTTATCTGGATGAAGTTGTTTAGCCATTTTCCTAAATGCTGACTTGATATGATTCTGCTTGGCTGAACGTGGTA

CATTTAATATATCATAGTAGTCATTTGAATGGCAATATACCAAAATGGAAAACGACAGTAAATTGTAAACGACGAATTGA

ACGTAATTCAAAATCATTTTATATAATTAATTTTTGTGCAACAATAAAACCAATAATATCACGAGTAAGATTTACCAGTG

ACTAGAAACTAAAAAAAAAATCGTCAAACAGGTATTTTACTTTTTCCCTAACGAATCAACAACTTAAATTTGATCATACA

TAAATCCATTTTAACAACTTCACGTTTCACAAACAGACGACAAGACACTTTTGACTTTTCGCGACAAATAAATTGATTTA

ATTTCACTAAACTCTGGCTAGTTTTCAAATAATATACGCGTTGTGTCAACCTCCGGTAGATATTGTAATCATAAGTAATG

TGGGCGATTCTGATTGGCTGGT

>contig_5872

CTCTGACCGAACACCAGCGTTATTCGTCGTTCAGTGTTATATCAGTTTCGTGGCGTGCAAATTTGTATAGGACAGCTGAG

ATTCAAATTCGATTTGCTTTCATATTCTATTCCAGCGACAACGTAATGTAATAAAGAAGCCATTATATTATAGTGTCTAC

TCGTACTTACGACGCTGACGACGGCGCACGCACCAACATAACACTAAATTTCGCCGTGGCCGTGGTCGCCTTAGTCACGC

CGACTCAACGATTCCTAAACGGCCGTCACCACTACAACACGGACACTGTGATTAATGTTCTCTACAGCACCATGATGATA

AAATTATATTGTTTGTATGCTATATTATGGCTATCTGTTACGATTGTTTGGCGACCAGTACTATCTGCTGGAACAAAAAA

GTATTCTGAAATTGAAGCTTCCAAATATTTGGACAACGCCAATTACGCATTGACTGAATGGACAAACCGAGTCATACATG

CCAATTGGAATTGGTTAACAAACTTAACTAATGAAAATGCTGAAAAAAAGATCGCCATTAATTTGGAATTTAGCAAGTTC

TTAAAGAGTATGTGGGTAGAAACTGTGAAATATCCATGGTCAACATACAAAGACCCGGATATCAAAAGACAGTTTAAATT

AATGTCAGTATTAGGAACAGATGCTTTACCCGAAGACAAATTGAAAAAACTAGATGAAATCGTTTCGGCCATGGAAAGTT

TGTACGGCAGAGCAACCATTCCTGAGTATGGCGATAACAATTTAAATCGAACTTTGAGTTTAGAACCAGATATAAACGAT

ATATTGGATAAAAGTACGGATGTAAATGAACTGAAACATGTATGGGTACAATGGAGAGAAGCCACAGGAAAAAAAATTCG

ACCTATGTATGCGGAATATGTAAAATTATCTAACGAAGCGGCGAGATTAAACAATTACACAGATAATGCAGAATTTTGGA

TTCGAGGATACGATGTCGACGATTTTCGGCCGCGAATGGAACATTTATGGAATCAAATCAAACCTCTATACTTGCAGATA

CACGCCTACGTGCGTAGAAAGCTATGGGAGCTATACGGTAGCTCAGTGATCACAAGAAGAGGACCTATACCAGCACATCT

GCTCGGTGATATGTGGGCGCAGTCGTGGGAACGTTTGGATGACTTTACTCGACCTTACCCAACTATCGATGATGTAAATC

CCACTTCAGCCATGATAAATCAGAATTACACTCCGAAAAAGATGTTTAAAGTAGCTGAAGAATTTTTCACATCGTTAAAC

TTGAGCGCAATGCCCCAAACTTTCTGGGAAAAATCTATTTTGGAGAAGCCAAATGGTCGCGATCTGGTATGCCATGCATC

CGCTTGGGACTTTTATGATTCTAATGACTTTAGAATTAAACAATGTACATCAGTAAATTTTATGGATTTTATAACTGCCC

ATCATGAAATGGGACACATCCAATATTTCTTACAATATAAAGACCTGCCGTTCATATACCGCGATGGAGCTAACGAAGGT

TTCCACGAAGCGATCGGAGATACAATCGCTTTGTCTGTATCAACACCAAAGCACTTACATAAGATTGGTTTGTTACCTAA

AACGAGTCGCACATATGAAGCCGATATAAATTATTTGTACAAGATCGGATTGGACAAAGTAGTTTTTTTACCTTTTGGGT

ATTTAATGGATCTATGGAGGTGGAACGTATTCAAGGGACTCACGACGGAAGATCAATACAATTGTGATTGGTGGAAACTC

AAATATTCTTATCAGGGAATCGAGCCACCGGTGACCAGAACTGAGAACGATTTTGACCCGGGATCCAAATACCATATCGT

TGGGAACGTGCCATATATTAGATACTTTGTGAGTTACATCGTACAATTTCAATTCCATCAAGCATTATGCGAGAAAGCTG

ACCAGTTTGATCCGAAAAATCCAACAAGTAAACCATTACATGAATGCGACATTTATCAAAGCAAAAATGCCGGAAACGCT

TTCAAAGACATGTTAAAATTAGGATCTT

>contig_6123

TTTCGTTTTCGTTTTGGTTTTCTCGCTCTCGCGCGTGAACTCGCGCGCCGGCGTCTCGGGCGACGCGCGATTGCCAAGGT

CGCGAAGTCGCGCGCTTACGCGTACGGGCTGATTCTGCAGCAGTTGAAATGGCCGGTCTTCTGGTGGCAGCCCAACACTC

TGTTGCCGAAAGCCGTCGATGCCGCAGTGTCGTGTGTGCTCAGGTGCCCTTATAGATCGTAATAATATAATATTATAATT

CTTTTTTTTTTTTTCGAATTATATTTTTATTAGGCGATATTATACGCGTACACTGCAGTAGCACCACACTCGACGGCGGC

AATTCGTCTTCCGGTGTTTACGGGGTTGTTTCCGATCCGCAACCTGCAGTCCCGTGGAGTATAGTCTTGTTTTCCCGCTT

CGATTCCGGGATAGTAAGTCTTTCGCGTGGATCGTCGTCCGATCGTTTCCGCGTCCGTATTACACTTCACTCTTATAAAT

AGCATATTATTGTACCTAATATCGTCACGATCGCCATCATGCACTCGAAGACTATCAAGTCCGTGATGACAGCCGTAGTG

CTGGCGGCCATCGTCTGCGCGGCTGCGGCAAAGCCCGCGAAAACCAGCAGACTGGCTAAGTACGCGACGTTATGCAAGCT

TTCAGACCCAAAATTAAGCTTATGTTTGACTACCCTAATGAAGGATATTTTAAAGCACTCTAAAGCAGGTATACCTGAAC

TTAACATACCCACGTTGGAACCATTTATCATTCCGGAAATTGATCTAAAAATATTCCAAGGATTAGGTGCTACTATTTTC

GGTGGCACGGTTCAAAGATCCGATAAATCCAAAGCATTTGCACGTAATTTAATTATTCATCATTCATCTGAATTCGATAT

TCACGACCTTAAGGTAGATATCAATAAGAACGAATTCTTCATCGACCTCAGTTTCCCAAAACTTGAAATGGAAGGAGAGT

ATGATGTAAATTTAATTATGTTTAACATGCCAATTAAAAGCACAGGACCTGTATTCATAAATGCCACTGACATTACAGTC

AAAGCTACATTGAATGGAAAAACTGTTAAAAGGAAGTCCGAGACTCTTTTATTGTTCGACAGCATTGACATCAAAGTTGA

CTTTAAAGATTACTCAATATCGATTCAAAATTTGTTCAAGAAAGATCAAAACCTAAACAGAGCATTGAACGATTTGATAA

AAAGCCAAAAAGCTGAGTTAAGAAAACTAACGATGCCAATGATCGAAGAACTAGCCGGTAAAATGGTGCTCTCTATGATA

AATCAAATTTTGACCGGTTTACCTATTGAGGAAATAT

>contig_6230

TTTTTTATTCAATTTGTTTGGTTTTGTTAAATAACTTCAGCCCTTTATTTTATACCTTAAATAAATTATGGAACACAATA

CAAAAAACAATATTGTATCAAAAAAAATTATCAAATTTATACAATGGGATAATAAATTTAGTTATTTATAACTCATCTTT

GACATAAGAGGGTTTTAATTCTTCAACTCTTTTGATATAATCTGTCTTTTCTAAGCAACCATCACAACTTTCTCCCCAGT

TATCCAATATTTTCTTCAAATCTTTCACTTTCATTTTCTTTAAATTGACTGTCTTCCAATCGATTTCTTTGTCATATTTA

ATATCGCACACTTGAGCATCCATTTTCTTCAAACGTTCACAGATTTTAAGTGCTGGCATGGACCATGATAAAGGTTTGGA

CATTTCACTTAATATACCAGTTGCAGAATCTTCTAATCCACCCAAGTAATAACACAGTCTTTTCTCCTTATCAATTTTAG

TAGACAAACAATATTTTTTGAATTGTTCTTCGATATTTTTAGGATTTTTTTCACCTTCGAGTGTTTTTGAAAACTTATCT

ATTGTACTGACACAAACTGGACAATCTTCTTCAGTAAATGTTCGGGATTGTGCTTGAAATACATTAACAATGAAAAACAC

ACATACGAACAATATGTGTTTATCCATTGTTAAATAGGATTTTGTTATACAATATTCAGAATTATGTTAAGTTGCTTATT

TTTATAAATAAATTAAATAGATAAATAGATAGTCACGGGGTTCGAATTATTTGATTGTAGTTTTTTAGTACTTTAAAGAA

AAATCGTAAACGACAAATACTTTTGATTTTTATTGAAGCGGTAACACATTTAAAAGACACATCACAATTTCACAAATATT

ATATTTTAAACTTATCAAATACATATATAATAGTATCCTACTGTTTTAGCATATTATCCAAACACGACTTGGCGAAATCT

CATTGGATAACGTTCCGGTATGATTGAAGTATGAAACGTGCACAATGCACATGATTGTATTACCAATATTCC

>contig_6277

GCATAAGAATATATAATTGAAATTAATTATGGTTTACAGCAATGTTATAGTATAAAATAAAATATGAAAGTAAAAAAAAA

AAAAAAAAATTTGTATAATATTTATAATACGATTATATTTGAAAATCTTAATGATGGTTTATAGTCGGAATAAGATATTT

TTGAGTCAATAAAGTTTTGTTATTAAATAGTTCACTTATAATATTATACATTTGGATAAAAGATGATATTTTAATGTTAA

AAGTTACAACACGAAATATTGTGCCATCAAGTCTCGGTTTAATGATACATTCCTTAAATGGATTATAATATAGTACAATG

AATTTAATAGTTTTAACTCGCTTGTTTTCAAGTCTTTCGTTTTGATAATAGGATTTTAATATAGATTAATTTATACCAAC

AATGCAAATATAGTCAAGCACGTTTTGACCCTAAAGCAAGCCACGAGCAGCAGATTTTTTTCATGGTAAATCGACATTTC

AAAATTACACAACTAATCATAATTAAAAATTGATCTATGCACATGCACATACAAACACATACATGCTCACAATAAATGAT

ATTACCAGTGTTTGTGTGTACAGGCTATTATCTTAATTAAATTCCGGATCGGTATTATAAATGTGTTACAAGGTGATCGG

TAAGCGTTTTTGAAATTGGCGAATCGTTGAATTTAATAACAGGTTCATATTTATATCATCGAAAAAAAATTACCACTCCT

ATTTGCAAAAAAGTGTAACGGATTTTAACGCCATCAATATTGTGTGCTGTTTAATAAGATTGAATGAGCTTCCGAGTAAA

CATAAAACAGTATTGGGCAAGGACTTATCAATATTCAAGGTGACTTTTGAACGACTAAAACATGTGTAAAAATAAAAGTA

TTGTAAGGTCTTGTAAACGTATTTAAGAATAATATACAATTATCATATTAACAAGTATATAATAGATAATAGGTAGGTAT

AATAGAAATCTGTGTGCGAAGTGTGCTTGTCTGGAGAAAAAAAGGTAAACGTCGAAGCGGGACCGGTTTCGATATCGACT

AAAGTGATTGTTTTACCACCGGAAGTCGAAAATTAATAATTAAAAGATAATTCGTCCGTGGGCGAAAATGCCTCGTCCAT

TTTTTCTAATTTTATCAAGTCGTCTTCGAAGGAAACCTTAGAATCGAGCTTAACAATTGAAGAATTATTATTCAAAGCCA

AGACAGCGGCTCCTTTAGGCGGTATGGCCTCGTACAGGCTGTTCTCCACGATGACTGTCGCGTTGTCCGTGATTTTGGAT

CTACATGGCACCGCCTCGGAGTTAATGTTACCCGGGAATCGCACTCTGTTAGACGTTAGGTTGGTTCTGTTGAAAGTCGT

AATCCAGTTACTCTGACTCATGTTTTCGATATCGTCTTTCAGCGGCGTTGTAGACGGACTGGAAATACATGACAAATAAC

GGCCGTTCTTGACATATCCAAAATATAGCAGACCCACGGCTACCATGGACAGCATCAAGAAAAACATCGCTGCGTACACT

AAGGACATTACGGTACTCGTTTCATTGTCAGAATTCGTATTGTAAAATTGAGTAACACCCAAAACTTTAGCGTTGTGGTC

TAGATTCTTTTCCAATTCCGATGCCGATACCACCGAACCATCCGTGTTGTTCACTCCGTACACGGCCAACACCCTCTCGG

TGCGCCACTCCCCGTCCAAATACGTGTTGATTACGTCGTTCGTGAGTGACATTACTCTGACGCCCGATATTTCGGACAGC

TGTGATTCTACAGCTGTGTGATCTTCGGAGGAGCTAAATTGTACACTCACCCGGACTATGTCCGAGAACGTCAGGAACTT

CACGGTAATAGTCATCGTAGATTCTGATCCATCGGATTTTCCGTCGTTGTCTTTGGCCACCACTTCAAATGTTTTATTCG

CAGTGCTATCGTCCACCAGACTCGTACAATATATTATTCCGGTCTCCGAGTTAATGATAAAGTCGTTAGCGTCGTCTCCT

CTTAGCGAGTATTGTATTTTTCCGTGTTTTCCTGCGTCCTTGTCTGTCGCCTTGACCTGCAGGAGGTGTTTGGGAATAAT

GGTGTTGGCCACGTCTATCACGGGATATCCGACGACTATGTGCTTTTCGGGGAACACCGGCACGTTGTCGTTGATGTCCA

TGATGACCAACTGCACCAACGTGGTAGTCGGATCGTACACGATGTTATTCACTTCGGACAGGTGTAAAGGCTGAGTGGTC

GGCAGTTTTTCACCCGGTGAACACTGGACTTTTAGTCTCAGGTACGCGGACGGTTCTTCCATGTCTTCGAATGGGTATGC

CTCCCGGTCGATGGGACTTTTCAAGTGCACGACACCTGTTAGTTTGTTGATGAAGAAGAAGTCCGCGTTTGTTCCAAATG

GATATTGATGTGTTATTGCAAATCTGCATTGACCGCTCGACTCAGTACCCGGAACAGCTGTTATTAATATAGTCTCTTCG

GTTATATAGTGTTTACTGAAAGCTCTGATAAATTTAAATGTGTCTGAACTACATACCGCTATATTATCAATAAACATATT

AACTTTGGATACAGCTTTTAAAACGGTAGTAGAATTAGGACCAAAATCCACTGCTACGATGTCAAAATTGTAATGTCCTT

CATCGATATTTCCTAAAACCTCCAATTCACCGTCATCACTAACCCTCAGTTTTGTTAAAAGTTGTTCAGGTACGTATTCC

AAACTGTAATTGCCCTTGAAGGAGTATTCATTTTTCGAAAAATATTTATCATTTACTGGTTGTTTTTCAAATTCCAAATC

CGTGTTTTTTTCTTTTGCTTTGTGTGTGGTTTTGATGGTCTTAAATTTGTAAACTTTACCTTTGTGACCATAAGCCAACT

TTAAAGAATAACTAGATTCTTCAAACGCTAGTACAGTGTCAGGCATGGGTAGAAATTCAACAATTCTGATGAGTACAGTC

GTAGAACCACCAACCGTAGTTAAATCAAAATTTTTTATTGCCTTAAGTTTATATACAATGGTCGAGTCATTGGTTGATAT

ATCTCTCAAATCGTCGTCGATGTTGATGTACACCTTGTCTTTCGATCCGAATGCAGAAAATTTTTTAATAATACTCTTAT

CTACATGATGACAAAAATGAACGATTGGTTCCAACACCAAACTGTAGTTTTCGGGTGTTACATAATCAATTGGCGGTTCT

AATCGCATATCTTTAAGCAACTCATTAGATTGTATTTTATACGAATAGTATGCTTTTTCAAATGGATATTCTTCTGTTTC

TTCGTCGTTAACTGCGTGACCCACAACATTCGCTGTACGTCCGAATTCGTGGGCATTTTTTTGTAGTTGATTTTCAACGT

TGCTTAACACCTTTGAACCGCTGATTGAAAAAATCAGGATCGCACAGAGCAGATAAAATAACGATGAACGAGTACCGACC

ATAATTATTACTTTTATTGTGATTCGAACTGATCGTAAAGAACGCTTATTGCTGACTGCAGTACGAAAAATAAAAAAATG

TTTGACGAAGATCAAAAATTGTTTACTGTTCTTATCTATAAATGACTGCACACCACACACGCACTTGTACACTTGTACAC

AGTATTATTACTTATTAGTGTCGAGTGCCACATTAAGGAAAATAATAATAATAATAATAATAATAATAAACGTTAAAATT

ACACAGTGCCTGTCCCAGATAACAGTCGCCTTCTTATCTGCTCGCAATTAAACCGTACGATCAATTAAAAACAAAAGAAA

CAATAATACGCGTCCGCACACGCACACAGCGAACAATATAATATAATAATATACATATAATATACTCGAACGTGGATTGT

CAAGAAAAAGCCATTAAATCAATCGTGGTTTCTTACGCGCAGTACATTATAATACTATATATAAATATATATGTATATAT

TATAATGATAACGATATAATATTATATGCCTCCTTCTCCGCCGCCGTTTATCGAATGGGTACGCGAGGTGTAAGCTTCAC

CATGCTCGCCCATTGGCCGTCGCACAAGTCTTCAAAGGTCATGTCTTCGACGGGCTTGCCGCCCAGGTCCCTGGGCCCTC

GGCAAGTCGGGTTCGAGTCCCACACTTTTTGCCTGTTCGCCCGCAGCCATCTAGCCAAGTACAATATTGTGCAATCACAA

TGCCACGGGTTCTTAGACAGTTTCAATGATTCCAACTTTGGTAGGTGATTGAAACAGTCGTTTGGTAGAAATTGGAATTT

ATTGCTGTCAATGTGTAATTTTCTGAGATTCATTAACGGTTCGAAAAGCCTGTCGTCGATAATTTTTAGCACATTGTTGT

TAAGAAACAGCGACGAAAGATTTTTCAAACCATAAAAGCTGTTGACGTCCAACTGTTGGATGTTGTTCGAAAAGAGGTAA

AGTTTTTCGAGAGACGCCAAATTCTTGAATGTCCGTTCCGGTATGAATTCCAAGTAATTTTGACCGAGATGTAGTTCGTT

CAAGGCTACCAAGTCAGTGAACTCATTGCCGTTAAGTAAGTTAATCCAGTTTTCGGTTAGGTCTAGATACACCAATCTTC

GCATATTGTTGAACAGCCCAGGACGAATAAACGATATGCGATTGTTCCTCAAGGACAAAACTTCCAAGTACTCGACGTCG

TCAAATACCCCCAAGGGTAGTTGTTGCAATTGATTTTCTTGCAATTTCAACCTTTTGAGCGACGACACTCCTCGAAATAT

GTCAGACGATATCTCTAACAACGAGTTAGCCGACAAATCGAGGTCTTCTAACTTGTTGAGATCTTTGAATATCGACTTCG

GAAAGACCTCTATTTTGTTTCCATATAAATGTAACAGCACCAAATTTTTCGTCGATTGGAACAACGTTTCCGGTAGTTGT

TTTATATGATTCATTGACATGTCTAATATTTGTATTTTTGTGTTTCTTTTGAACAATAATGGTACTTCGATCAAGTGATT

CCTGTGAAACCGCAGTTTTGTAAGGTTTTCCAGCGTGTTGAAAATGGCTGTAGGTACATAGTCGATATCGTTATCCGTAA

GGTCCAGCTCAGTCAAGTTGGTCAGACCTTTAAATGTGTCTTTATGTAGGGAAACTATACTGTTAGTACTCAGGTCAATT

GCGCTAACGTCGTACGGCAGTATTTGGCCCGGTGGAATTTCTTCAAAGTCCTTTCTCCAACAGCTCACCGAATGTGTTTC

AGTTCTGCACTTGCACGGTCCGTAATTACAACGCTTCCATCGAGAGACTCCGGACGTGATTGTCCAAACGGGCTGTTCGA

CGGATCGCAGTCGCCTGTCCAACCGCTGTAGCCTGGCCTCGAGCAGCGCGCCCCTGTCGCCGGCCACGCCTCCCCGTCCA

CCGTCTTCGCCGCGCAGCTTGTCGTTGATCGGCCGCACCGCATTGGCCAGG

>contig_6314

TTACTGAGATAATGCATATTTATTATTCATAAATGTAAGTATAGATATTATTGGTATACAGTTGCCAGTTTGTATGCCAA

AAACTTTTAATCTGCTTAAATTTTTTCACTTTACACCTCTATAATTATTATTAAAAACTTAACACACTGGCAACAACTCA

GACACTGACATAATACTATGATAGGAAAAGGTCTGACCATAAACACTGGTTGGGACCATCAGAATGCAATGTATTGTCTA

ATGTACAATTATCATACTTGATTTACAGTGACCAGTTTTTTTATGCATATAATACAAAGATGCATTGAACAATAAAAAAC

AAATTATACAGAAAGTAAAGGTATTACATGATAATTAGCAATTACAATGTTATTGGAAAACAATTTTTCATCAATGTTAT

TATATCATTTTATTTTTTACATTTGATAACTTTGTACCGAAAATAATATCTATATTGTAAAACAATAAAATTGTATTTTA

CAAAGATATAAAATATTAATATTCTGTTAAAAATTAAATAAATAATTTTACAATAATTATATTATTAAACAGTGGTTATA

AGTCTTCACTATATGTGTGTATTTATTTAAGAAATGTTTAGGCATTACACCAAGCACAAACGCTCAATGATTCACCTTAC

ATACATTTATAAATATATATTAATGATTATCAAAATAGAGATGTGATGCATGCCGCTACATGTATATTGTACAATATATA

ATAACTTCAGCATTCAATTATCTTATATGTATAAATATATATGTGTGTGTGTGTGTGTGTGTGTAAGTATGTCTAGAATA

CAGTATAATAATTATTACAAGTACTAATAGTTAATTATAATTTCCTAGACTGGAAAATGAACAAATGGACTTGGTTTTGA

CCACAGTTTATTAATCAAATCTAAAGTTTTATCAGTTCTGCCAAATTTATACACTTTTGTTATACCAAACCAGTATTATG

TAAAAATTATTTTAACATAATTTTGTTTCTCGGTATACAATACATAATACATAATATTATAATACAAAATGAGTATTCAA

AGTCATAAATTAAAGCGTTCAGAGATTTCAGAAAAAATACTTTAAAATAAAGTACAGGCTAAAATCTAATTTAGTATAAC

AGTGTTATTCTCTTAATTGCTAATTGTGCATAGGCATTGACATGGCAAAATAACAGATATCTTTAAAATAGAATGACATT

TAAATAGTTAATAAATATTAAATTATGTACACATAATAATATAATTTGAATGGGTACCAGCACCTTATAATGATAATCAA

AATATTACAATAATATGATAAAAATGGAACAATCTGAGATAAGGACAAAAAAATTGGAGGGAAAACAATAATAATAAAGT

TAATGGTAAATTTTAAAATATGAACAAAAATAAAATAAAATGATTCACATGGGAAATAAACTCAATAACTTAATAGTAAA

AACTATGTAGGGCAGTTATAATTCGATATGCCACATAGTATCGTTAAGCTATAATTCTTCTTTTAATAATGTGTATCGAT

TCTTAGATGGTGCCAGTTTTTTAAAAGTAGATTCGGGTGGCATCGTCTGTTCAGCAGGTTGGTTATTACCATGAACTATT

TCTATGGCAGAAATTTCCTGAGGACCTAAATGGTACTCTCTATGTAATTTACCTGAGTACAGATCATCAATGAATGCTTT

CAAATGCTCGTGATTTCTGTAATCACTTTTTGCAGGAAACTGATACATGTGCTTGAAACTGTCAATAACGACTACAGGTA

AGTCAGTATGTCGTTTCCCCAGGTGCTGTAATGGATGAGCAAACTTAACACCATCAGCTGTTAAAAAGTTTACCCGTTGC

TTCTCTTGTACTAAATGAGTGCCAACAATTTCTTTAAACAGTTTAATTGACTCCAAATCATCAGGATGATAGAAAAATAG

TAAGAATGGTAATCCTTCTTCTGTGATTTCTTCAGCATTCTCAAATGTTATTTCACGTACTAATGGTAAGCAATTGTCTG

TCGCCCATATATTTAATGAATCATAATCGGCTGAATTTCCTGTGAAAGTATGATGTTCAGATTTGGGAGATGCTCGATCA

GGTCTAAATTCTATTATTGTTTCATTGGGCGGATGCATAGCACGGAATGTTTCACCAATTCCAGCATAAAAAAGACAATC

ATCTTTGAGATTTGTTGCCACTTTTCTAAATATCTGGTATTCTTGAGATTCCTTGTTTTCAAAGTATCCGATTACAACTC

TTGACTTCACATCAGCTGCACTAAATAAGTTTGCCACTTCTTGTATGGGATCTTCAAGTTGTTTTTGTACAAAAGACACA

AAAGCTTCAACTGATCGCTTACCTCTATATTCTCTTTTAGCTGGTTGTCCATTTATTAATACTTTTATAGTAGGATATTT

AGTAATTTGAAATCTAGATGCAATGGAAGTTTCACTATCACAGTCGACTTTTCCCAATACAACTTTACCAGGCTCTGGAT

AAAGTTCAGAAACTTTTGCAGCAGCTTCATCAAAGATTGGAGTAAGAATACCACTAAACCGACACCAATCGGCATAGAAA

TTTATTATAACTAACTCATTGGATGCCAATGTCATATCAACATTTTGGCTGGTGAGTTTAACAGCATTGCTGGTTACTGG

ATTGTAGAACAGATAAGCGGTGAAAAAAAACAGCAGGAACAATTTATTAGCGAATATTTTCCAATAATTTTGATTCATGG

TATTTTGAATACTATGCTTGAGGTAACATCAGGGTATGGACTAATTCTTTATTTCTTTTGCAAGAGAATAAACGCAATTC

TTTGTGGAGTTTGGACAGTGGAAATAACAATCACAGAATTCTACTCGATGATGGATTACGACCAACAGACCATCACACAA

TTTTAGAGAATATGTTAAATAATTTTACCTTTAGGAATAAAATTTGAAGATATCACGAAAACCATTAGACGTCATCCGGC

GAATGTTCGTTGATTACGACACGTCGATTTGTTCCATGAAACTGATAATGGTAACGTGGAGAAAAAAATACGACAATCAG

AGGGACTAGTATAACAAAGACTATTTTTTAATTTTTATATAACATTAAATAGTAAAATCACGGTCCACGAATAATTAAGA

TATTATAATA

>contig_6513

CAGAGCCATGTTAAGATTTAATTGATTAACCAAAATTAACACATAAAAGAAATTTTCTTATTTATTTGTATTACCATTGA

CAATGCTTATAATTTACAACACAATATAATGATAACTAATTATTTTATCTACTACAATATTTTCTAATAGATCGAAAAAA

AAATAATAATAATAAATAAATAATTTTCAGTTAACTAAACAAACTGGATTACGTAAAATCCTCTGTACAATAATAATTGT

TTTAAGTACACCAAATACACATTAATACTCACCAAAACTATTATCTAATAAATTACTTAGAAGCCATTAGGTAAAAACAA

TTTTCTGTCAATACTGTTGTTTGCAATAATATGTTAGTTGAATAACAAAAAATAAACAAGTCTAACCCATAGAGTATAAT

GTACATAATATATACATTATAATAAAATTTTGGTTATGGTTTAGAGAAAACAAATATGTCAAAGCTAACAAAATTATTTA

TTTTCATTATAATTCTTATTATTTAACCTGTCAAATCATTATGAGATTATAACTAATATTTACAAATATGATAAATTAGG

CAGACCAATTATGTCTCCTTTGTCATGATAGTAATAAATCAATAATAATCAATGAATTGAATAAAAAAATTTGATTATAA

AAAAAAAAAACATTGTGAAAATGTAAAAAAATAATAATACATAAACTATGTAGTATATTCAGTTGTATAATATTAATTAT

TTTTGAATAATTATTACATAAACTTATGTCTATACATAGAACAATAAGCGAGGGTTTGGAGAAATTATGAATGATAAAGT

AAATAATACATTTTTACTTCACGTGATGTTATATTACTTTAACACTAGAAAAAAACCCATTAAATAAAGAACTCACAATT

ATAGAAAAAATTATACTGATTACTACGAAATGAATAAAAAATGAATAATAATGTTATCATGAACACTATTTTTTTTTTTT

GTTAATTAATATTTAAATAATAATAATAATTAATGAATTAAAAAGTTTTAATTAATGACTAAAAGGAATTGATCAGTCAA

ATTGATGTAAAAGACCAATTTAAAAACTAATTTAGAATCAGACTTGTTAAGGAGGAACGTAGGGTGGGGGTTTGCGATAT

GATGGAGTTGATGATGGTTTTGATCTATGATTAGAGCCAACATTTGAGAAAGGAGGTGGTGGAGGCTGATATGGTGAATC

TTCTGTATCGGCAAGTAAAGCTGTGGTGGCCATTGGTGGTGATCTTTGATATTCAGGTGGTGGTAAAGGTGGCTTTTCTT

CTCTCATAATTATTGGTGTTTTATTTGTAGGCTCAAGTCTTTCATCCAACTCATCTTGGAATATAACTGGAATACCACGA

CTTCGGAAACTTTGTCTTTCATCATCACCAATTCTTAACTTTGATGATGGCCTTCGTCGATACAATAAACAAGCCAGTAA

TCCAGCAAATAGCATCATAGCTAAAATTATGATTGCTGGAATAATAAATGTTGAAAAATACTGGTCTGATGTGTCAAATA

AAAGCAAAATATTGTCATCGCGAGGTGTAACATTGTTGGGAGTTTTTGGTGGTCGAACTTCGGTTAATCCACCTTCACAC

ATACCAGAAGGTATAACATGTGCTCCAACTACACTAAATGATGGTTTCATATATTTTGTTACATTTTCAGATAACTTTTC

ATCTTCATTTAATAAGATTTTTCGCAGTTGTAGAACTTTTTCTTCATCACACCGATTATTTTCTAATGTTTTATTGTACC

ATGTCACCATTGTCATACCATTTACAGAGCTAATTCCAAGAATAACAATGTTAGCTGTATCTTTATCTCTAAAAAGATTA

GCTATACGTTCAACAAATAATTTTTTTAGTTTCGCAGAATTATTGAATTCTTTGAACTCTGGAGTTTTGAATATCATATC

AAATTCAGCAGATGGTAAAGGCTTAGTAGTATTGGGTATAGATACAACTAAACTATCGTGATTTGATCTACCTTCTCTAT

CAGTACAAATAAGTTGATATTCAGAAAGGCTTTTGTCACCCCATAATGGAATTCCATAAAATTCTTGGTTTTTAGTGTCA

AATTGTAACCAATTATTTTCTGGAACTTGTGTGCGATCAATAGTCATTAATGATAATTTCAATCTCCGTGAAGTACCATC

TTCGGGATCATAAAATGTATCTTCAGGTACGACATAAGATAATAATTGTCCATAAGTGGCATTAATGAGATCAACTGGAT

TGCGTAACACAGGTTGATAGTTTTGACTTGAAATAAATGATGTTGAAGCACAATTACCTAATCCTTGCCAACTAACATCT

TCCAAAACAACTGAAGAACCAAATGGTTTTCCAGATTTCATTTTATTACCCTTTTCTGGTTTTAAAATTCCTAAAATTTG

AATTATTTGTTCATTGGGACAGACTTCACGTGGCAATGTATCATTGGTCCAGGTAAACTGAATGCTATGTTTTTCTTTAT

AGGCAGCTAAAATAGAGATTCGTGGCCAGTCTAAGACTGTGATGTCCTTAACATCAGTTGAGTAAATTGTACTGATTTTG

TCAATGATATCTAAGCTCCAGTTAACTGGATGAGTCCATACTAATGAGTCTTGTTTTAAACGCATTTGAAGAGTTAACTG

ATGATTAATGGCGCGTAAGTGAGGTAGCTGTTGGACAACCAGTTCTAATGTATTGTTAACGGTCAGATTCCCAGAATCTT

TTGCTTCAATTATTACTTCCCATTTTGATACATGATCTTCCAACGGTAAAGCAAGTAAAGTTTGTTTTTCAGCATCAAAC

TCAACCCATGACAATAGTGTTGGATCTGTGTTTAATAGTGCCAACTTTAAGTGTTTTGCATCTCCATCTTCAGCATCATA

GAATATATTGTTGGGAATTGGATACTTAAATGGTTTTCCAGCTGTAATAGCAAGTTTGGGTAACTTATGTTTAATTATTG

GTGGTGTATTAGGATTTTCCCTATTTGTAGAAGTAGAAGTAGTAGTAGTACTAACAGGATTCTCATTATTCTCATTAGCT

AAATCCTTTATTTTGTCTATCACTAAATTAGGAGTTTCGGTAGATATGCTTTCATCTGGTATGATTGTTGTATCATACGA

ATCACCCATACCCACAGATTCTGATAATTGTTCAGTAGTAGGAGTGGCAGATTTTGACTCGTAGAATGAAGGCGTTGGCT

GAATTACAAAAGAATCAATAGCTCCACTTAAATCAAGAGTTGAAGATAATTTCATATTTTGAACAGGTGTAGAGTTAATA

ATGCCTTTTGGAATGTTCAATACAGATTCATCTCTACGGTGATGTCTATGATAATGGTCGGTATCATATTGATGGTTTAA

ATAAGATGGAGATGGTTCAATAATAAGCTGTCTAGTATCAGGAATAATTTCTTCTTCTTCATCATCGTCATCATTGTAAG

ATTGAGTATTAACATATTTGGTATCATCATAGTCATCTAATTTGACACTTCCTGAAATTGTGTCTTGTTTGGTACCTGTC

ACTTGACGTCGGCCTCGTAAAGGATTCAATGAATTTACTAATCTCCAACTGGTAACTGGTAACTGCAATACCTCAGACAA

TGTTCCATCAACAGTATTACTCTGCATTTGACGTACATGATCAATATGTTCTGGCAAAATATCACCTCCACAGCCAACCT

CCCAAAGGACAAAAGATGTATCATTGAATTGATGTTTTTTATGAGACTGGCGTTCAGATACTAAATAGTCAATTAACCAA

GAAGATACACTTTCTTGAGGCACTAATAACACAGCATCTTGAGGAAGACTTAAAAATCCTGCCAAATTTTTCAGTGCGAT

AAGTCTTTGTAAAGGTTGTATGCTCTTAAAATTCATACCAATCACAAGGCATAGCAAAATAGAATCTTCAGAATTTTTAC

ATCGTTTATTATTTCGAACATTTTTCCATTTTAGGGGCGCAATTCTAATAGAAGCTACATCAGAAACTATTCGACGACTT

TTATCTCCTGCTCTTACCAACAAATGATCACGAACATCTTCAGGTCCAGGTATTCCTTCAAGTTCACCCTTACCTTGAAC

TAACCAACTTGGAAGACCACCACCATCTTGACCCAAAAAGTCGATGGGACCAGTGTTGTCGACCACTGAATCATGAGGTA

TTTTGAAGTAGAACAACTTGCCTACAGTGGCCACGGGTTCAGGGATGCTCCACCATTTGTGTTTGACGTCGCCGGACTTG

TGTGCGGACGCCAGACACAAAAGAAACGCAACGGCTGCGAGACCCTTCATTTGGACATGTCTGGGTGCGATGTTATTTGA

ACGCGAAACGGTGTGCTGCTGCTAAATTACTATAATGATATGCACGTCCAGTAGGGCTAATGCGTGGCGGCTGACGTCAG

ACACGGGTGATCTTTTCGTGGCAGTCACGACGCCAATAACATAATAACGACAACGATGAAAATAATATTATGACGACGAA

GGGAAAATAATTATTAAACTTGTACGTGCGACGTGTGTGCGGTCACTGCAGCGAAGTACCAACACGATTACGATTTACGA

CTTTTAAGTAGGCGGCAGTCAGCTCGGCAGAGCTTGTGTGGGAAGACGGATGAACATTCGAGAGGGGAAGGTTCAAACAC

TGATATTACCATAATATTGTATCATCTCCAAAGTCTAAGACGATGATAATATTAATATTACCACCTCCATGACAACAATA

TTTTCCAATGATCGTCACAGCATAAA

>contig_6531

CCCGTTCTCGTCGAAACAGCATCGCAGTTTTGCTACGTATCTCGCAAACACAACTTTGTTCCGCAAAAATATAATATATT

TGTATTATATCGGTATTAAGCATAATATGAAGTCTGTCGTCCGCGTTTCGTTGGCCACCGTCTTCGGACTTTGCGTGTTG

GCCATCACCGTTTCCGGTCAACTGCTACCGGAAAACCCCATATCGAAATCCACGGAAATCCTGAGTGATATCCTACTGAA

GGCCACAACTGAAGACCAATCTAATTATGTGGTGTCGCCGTTTGCGAGTTCTGTGATTTTGGCGCTAGCGGCCGAGGGAG

CTGACACGGAAACTAAGAGCCAACTCGTAGCAACCATGGGTGGTGAGCTACCAGACAAGAGTTCTTACAAAGAAGTTCTC

ACAACTATCAAAGGTTATTCGCTAGATGGATTTGCGCAGAACAAAGTCGTTCTAAAGAATTTTTTGTACGTTTACAAAAA

CTACAGTGTACATGAATCGTTCGCCCAACTCGCCCGGGACTATTACTTAACTGACGTGAGGAGCGTCGAAAGACCAGACC

TGGCAACAAAACGAGATGCGACCAACAATATCTCCTTAGATGACGATAACAGCTCCGATTTTAAAGAGCACGCCTTGTTG

ATCTTCAACGGTTTATCATTGGAAATGAATTGGCCGACGAGCACGTGGCACAAAACATCATTATCCTGGAACGGCAACGT

GGTCAAAGCGTTCGGTGCAGCCGGAAATTTCGCCATCGGTCAGATACCTTCACTTGATTGTACAGCGTTTAAATTGCCCT

ATAAGAACACTGACTATGCCTTGTTAGTGCTTTTACCGAAAAACAAAGATGTGTCACTGAACGAAGTACTGAAAAAACTA

AATCCAGAACACGGTATCGAACAACTAACGAAAGCGATGACGATGAAACCCGGTTTTGTGACTATGCCATGTTTCCAAAG

CAGTAACATTACCCATCTGAAAACGGTTTTACAACAGGGAACTCAAACGAATACCGTATTCACCGAATCGGCGGACCTGT

CAAAACTATCATCGGACAAGTTGTACTTGGACGACGTGGTCCAGCAGGCCGGAATCCGCGTGTGCGTCGAAGGCTCCTCT

TCTTATTCGCTGACTAGTTCCGCGTTTACGTCGCAAAGGGTCATCAAGGAGTCGGTGGTCGTGGACCGACCGTTCGCCTA

TGCGCTTTACAACGTAGCCAATGGAATCGTTTACGCCGCTGGCAAGTTGGAACGGCCGATCTGGGAAGAGACTGACGATG

GCAGCGATGGTTCTGACATCGAGACAATCAAAATTTAGACAGGACCGAAGCGAAGATGTATATTATTATAACTGGAGTTC

GGATTACGTTTTAACTCTAAAAAGAAATTTTCTTTAAAAGTACTATAAATTCCTAAAACTATAACTATATAATTAATTAA

CGTACAACATACACGTTTTTAGAATATACAAATAATAAATCTGCCAAATTTCTGAACTAGAACCAGTGACCGTGGTAGAA

TGAAATATTTTTATTTTTGAAATTTTTTCCGACAAATTAAATAACCAAATAATCCTAAATTTAAAATTTATAAATAAAAA

CTACCAATAGGAAAATAAAATAAATATTCTATTCTACGTCATCCGAACTTCATGGTTATAAATTATAATATAATGAGTCG

TATAGCCGACGCTTATAATTTGGTTGGCGGATATAATAATCATAATAAGTTATTAATAATAACAACGGTCGACGAAGATC

GTCGGCGATTTGACGACGATTATTTTAATAATATAATTTTTTTATGATGGTCGAACATTTTAAATAATATTATATTATA

>contig_6659

CTTCCTATTGTCATGTACGGCCTTTGTGGTATGTAAAACATGCAACAGTTTGGCGGCCTGATTAGCGTGCCGGCATAGAC

AGGCCACAGTCCACTTAGGACCCTGAACAGAGACGATTTACCGCATCCGTTGGGGCCAGTGATCAGCAGGTGTTCGCCAG

GTTTCATCGTCATCGTTAGACTGGACACGACAACGTCGCTGTTAGGCGTCACTATGGGAACGTCGAACAGTGATATGCTG

CCGTCCGGACTGTTTTTCACGACCCCTCTGATCACGAGTTGCCCGTCTTTGTTGTAGTGCAATTTTGGTAACGTCTTGTG

CACTTTTCCGTTGGTCAATCCATTTCGACGGTATTTACACTTGCTGACGTCGTTGAACACATCAAGCATAGCGCCTACCC

TGTACGTGTAACCAGCGAGCTCGACAATTTCCTTATACGAAGTCATTAATCTCTCTACGGCATCGGCTCCTGAAGCAAGC

AGATTTCTTGCCGTGGTCAAGTATTGAGTTCGCTCGCTCACTCCTCCGTCTGGAGAATCGTTGAGCTCTGCTACTCTTGA

GCTAGTTATAATCGGTAACGACACGACTACCATTCCGGTTCCGCTCCATACGTATTTCATGAGGAACTGTTCTAGGACGA

CGTACCACAGTCTTTGTGAGAAAATTGCGTTCATATGTCGGACCAACGACTGATAAGCGTTTTGTAAATGTATCATTTCC

ACCTTGTGACCACCATAGAACGCGATCTCTTCGGCATTCGTGATAACCCTGGAATGGATGTGACGGAGGTAAGCCTTCCT

GTCTGCCTCCACTGCAACTAAGGAACCGAATTTTGGTGACAGCATTCGCAGTATCTGTCCGGTGATGCTGATGACGACGA

TGGCCAATAAGGGTCCTGGCACGACGGCGGCGCCCATTTGCTTGCTGCTCCTGGCCAAGGTGAGCGCGATCAGAGCACAG

TCGAACAGCGGCTTGGTCAGGTGCGAGTACAGGTGAGCCACGGACGACGTGAACGCCGTGATGTCGTCCGTTAGCCGGTG

GTCTGCATTCTCAATCCGGCCGTCCATGTTGCTTACCCTATAGTAAGTTTGGTCCTTGAAGTACATACCGTACGCGTGGT

TGACAAGTCGAGTCCTGAAAGCCAGAGCCAGTTTGCATTCCAAATACCGGATCATGGAGTTGAGGAACGTGGCGGGTAGG

GCACACAACAGCCACTTAAACAGCATCCACCCAAAGTTGGGTACGTCCCGGCGGACGATGAATTTGACCATTCTGCCTTC

CATGGTGGCCACGAATATGGACATGAACGTGCGGCATATCAGGGCTATCGTGTGCATGGACAGCAAGCCCACCTCGGGCG

TCCAAAGGCCGGGCACCATGAGCCGGAGCAGTTTCTTGAGCTGGACCAGGAACTCGCGGTTGATGCCCGGTCCGGCGGCT

CTCGTTCTCTTTCCGGTCGCCGATCTCTTTCCACCGCCGATTACGGCCACGGCGGTGATCGCCGTTGACCCCTTTGTGTT

GTTGTTGTCGTTGGACTCGCCCGACGGTCGCCGCGATCCGCCATCGGGTCCTTTTTCGTCCTTACCACCATCGGCGCCGC

CACTGCCACCGCACACCTTCAACACGATGGGGTAACCTATCTTACAGCCGTACAGCAGTACGGCCGCTGCCAGCACTCCT

TTGCTTACGTTGTCCCGTCGGATGATTCGTCCGTCCAGGTATTTTGATATTACGGCGGGCATGATGATTATGGTCACGGC

GGTGGTGGTAACTTAGCCGGGCGACAGTTGTCGCGTCTAGTTGACAAAGTGATGAGTATGTGTAGATACTCGCACGACAG

TGGTCTTCTTTCACGACGTTCTGTTATTGTGTCAAAAGGGTATCGACGACTCGTGCGTGGCAATAGTAGTCATCTGTGCG

CGCCGATACGACTGACAATAATATAGTTACGAGCGTGTTATAAAGATGTATGCGATAACGTCAACGGCCGACCGGGCCGG

ATCAGTCCTCTGTTCGTTCGGTAATGATATCACTGATAACGACTATCGAAAAATAGTGTAATATTATATTAAACGACTGA

TAACACAACGATACGACGAACGCGCGACAATACGTGCGAAAAAAAATAAACGGCAAACGCGCAACCGACCAGACTGCGCG

CCGGAGTCGACGTTGCTACTGAACGATTCGGCGCGTACCCG

>contig_6719

GTTGGTATGCAGGATGACAAACCATCTCCTGAACCACCAGTGGAAAATAATACAACAGTGTTAAAAAACTCCATATCAAT

AAAACATTTTGTACCGAAAGTTGGATTATTGCATCATAGGGAATCTCTGCAACTGATACTATGTAAGCCCAAACTAATGC

CATTAAAATCTATGACATTGGAAAAATTACAAAAGATGCAATCCGAAGCAGAAAAAAAATTGTCAAATAGATGATTATTT

AAGTTATAAAATGACTGTGGGAATATTCAGCGCTGAAGTTATTTTTGCTTTTATTTTAACTACGGTGATACTCAATCGTT

ATGGCAATTGGAAAACTCAAAATATTGTTGTTACAACAGCCGTACTCATCGCTTGGTATTTTTCTCTTCTAATTATCTTC

GTTCTTCCCATTGATATATCATTGGCAGCTTATCGTAAATGTGTTCAAGATGGGCATAACCATTACAATAATATTACAAA

TTCTAGTCAAGATTCATCAGCCTGTAAGAAACCCTGGAGTTATGTGCCAGAATCTACGTTACCAAACATGTGGAGAGTTG

TTTATTGGACTTCTCAATTTCTAACATGGTTTATTATGCCAGTTATGCAATACTATGTGAAGTCTGGAGAATTTACATTG

AAGGACAAATTAAAAAATGCTATTAAAAGCAATACACTCTATTATAGTACATTATTGTTAATTGTTACAATACTTATAAT

TTACATTGCTCTTAAACCAGGAGTGCATTTAGATTGGCAAAAGTTAAAAGCTATCGCATCATCTGCTAGCAATACTTGGG

GATTATTTCTATTAATATTATTGTTAGGTATTGCATTAGTGGATATTCCTAGAGAATTATGGCGTTCTAGTCAAATTGAT

TATACATTGAGAAAAGTATACTTCAAACTGTCAAAACTGAATACAGAAAAGTTAGAATCCGAAGGAGCTTTAGAAGATGT

TCTTGAATCAATAAAAAGTGTTAGTATCAGTATAAGTCCAAATGATGCATTGTATGATTGTTTTCAAATAATTTTGAAAA

AAGTTCCCGAGGATCAACAAGATTTTTTAAAAAATGTGCGGTCAAATTCAAGGAATCATACAACACCTCCTTCAATTGGC

ATGCTAACTCGTCTTCATAAACAATTGATTGTTGCTGTGCATATGTATCATCGTACAGAGACACAGGGTAATTTAATGCT

AGAAAAAGCCATTTTCTTGGAAGACATTAATTCAAACATGACATCTAGAGAACGAAAGTTTAAAAAAATGTTTAATAAAC

CTTCAAAATTGGATAGTTATGCAGATACATCAACAATAGAATGGTATTGGTGGTGTCGCTTACATCCTATGATGCTTCGA

GCTTTATCAGTGATTACTGGCGTGCTATCAGTTATTATTGTGTGGTCTGAAATGACATTTTTCAAAAAGCAACCAGTGCT

CTCTATTTTTGCTCTCATGGTGAATATGGCAAAACAAAACAATGATTATGTTATGATACAGGTATTATCCACATTATCTA

TTGCTTATTTGGCATATTGTACGTATTCAACAATATTTAAGATAAAATTGCTTAACATATACTACCTAGCACCAAACCAT

CAAACTACTGAAAGTAGTTTAATTTTTTTTGGACTGCTGTTGAGTCGTCTCACATCACCAATGTGTTTAAATTTCCTTGG

ACTTATTCACATGGATAGTCATGTGATAAAAAGTCGTATTCTTGAAACTGATTACACCCAGATAATGGGACATATGGATG

TTATTACAATAATCAGTGATGGTTTCAATGTATACTTTCCAACCCTCATACTTGCTGTATGTCTAGCGACTTATTTTAAC

GTTGGAACTAGAATATTGTCAGTATTAGGATTTCCTCAATTTTTAGAAGATGATGATTTGGTGATCGATTACATTCAAGA

AGGGCGTTTGCTTGTTGTTAGAGAAAAAGAAAGGCGTGAGCGTAAAATACGAGGTAATGAAATGCGTCGTAAATATAGAG

AAAGGTCATTACCAAACACAAATGAAGATATTGAAGAAAGAGTTCCACCTTCTCGGAAAGAAAACATGAAATCATATTTA

TTGGATAATGCTGATCCCATTGATCGATCTTATCATAGCTACAGTACGAATAATACATCTGAAGTAGAGAGACAGTTGCC

CAGTGTGAGTACAAGACCAAACAGTACGTGGGAGCCTCCGAGGAATATATTTAGTGATTTATAATTTTGGTCGAAATCAA

TTATTTTATGTTTCTATTATATGAAATGCATTGCCATAAATATTTTCTATATGTTGAATTTTAATGTGTTAAGATTAAAA

TAATTTCTACTTTAAAATATGTATTATTATTTTTTTTTTTTTATAGACCACAAAATATTATAATATTATATTATTACCCA

AAATATATGCGCGGAAATATATTGCCGACAGTTTCTACTCAAATGATGATAACATAACAAGTATAACGATATAATGATTG

TACGATATAATATGAATAGCCGAAACGACAACAATAATTTTCATCGGTGAACCGTGTACGAATAATTATGATAAAAATAA

TAATTATAGTAATATCATTTTAAAAATTATTATTTAAGGACTAAAGATACTACCTACGGTTCAGTGGTGTAGTCAACCGT

TTTGTTTTAATTAAGGACCAAATGTTGAAAATCAACGATACCCACTATACCTATACTCACACTAATATACTTTACTCACG

TAATTTCTAATTTTCGATCGACTATAAAAACGCCGATGTCAATAAACTATTTTTTTAGCTCTTTCACTACATTACAACTG

CAGCGTATTAAGTACAATCGAAAAAAAACGACTTGTCATCGTAGTTACCGTTCATAGAGGACCGTATTTTGGTTTTAATA

TTACATTGAAACATCACATACGCATTACGCACGCATATAGAAACGCTAAGCTCGAGGAAATAGGTGTAATATATAATATT

ATATTGACATGCGAAATCGATCGCGAGTTGGTCTAGAAACGTAGCAACTTTGTAATATTATTATAATACCATAGACGATA

TCTACACAAATAAATACATTCGAGGATTTTCTTTGACGTAACAGTGACTAGTGAACCGCACTCAACCCTAAAAATATTAG

ATTACCTCATAACTATCAGTTTATCGCTTCCATTCGGTAAGTCTTACGATATCTCACATTATTTTACACCACGCCTTATT

ATTTTAGTTATTTATAAGCAACCTATGCTATTTTTCTATACAAAAATCAATACACCCGTCAATGAAAAAAAATATATTGG

ATTGGCTCCGTGCAATACTGTTTGAATTTGTCTCACGATAACCTACTCCGTTCGCGTCAAAATCCCAAACGCAGTATTAT

CACGAAACGCAAAGATAGGTAAATAACTTAACTATATATATATTAATTGTAAAGATAACATAACCCTTAATCTTCCTATC

TCTGCAACTACTGATGCAATGTCATGATATTGTAGTTACATTTGAACGCGTAAATGAAACAAATCTTTGTTTCGTAATTA

TACTTTCATGTTTATTGAGTCTCGCGCATCACTACAAACAATCTATGTCCTTACACGATTTTGGCATTTCGCATAACATC

ATAATATATTAAATTATAATAACAAGCTATCAATAAATATATATATTATCATTGTTAATAAAAATAATGATCATTAATCG

TTTGTGGTCCATATACTTACACCATTCATCGTTTGGTACCAAAACATTACCTATATACTACTATTACACTTATTTATAAT

TCAATATTATTATTATTATGTAAAATAATGCGTGTGACTGAATGTATAATTTGTTGACAATATCTTCTGTATAAATACCA

TTTAATAATATTACTATGTTCGTCAAGAAAACTGTTGATGATTATTTATGTAACAATTTAAACTATACAGAATAAGGAAT

AATAATTATTATAATATTATATTGTATAACTATTAACTATAATACTATAAGAAAGTT

>contig_6786

CAAAACTATGCTTCATAAAGTATTCGCAGTCACGCACGGATGTTCTTAAAAACTCTTTCTATTTTAAAATAATAATATCG

AAAATTTATACACCTATTTGTACATTTCCGTTGTAATATGATTGAAATTTTTCCGAGTTTGATTATCGAACTGTTCAGTT

CCGTGTACGTATTGTATGTATTGTTGGTCTTTCTATCGATCGCTTACTACTTTTCAACGTATACTCTCGATAAATGGAGC

AAACTGAATGTACCCTACCCGCCGCCCTTACCACTGTTTGGCAACTCAATGAAAATGGTCTTAACCTTGGAGGAACCGAT

ACACTTTTTCAGTCGTATTTACAACCAATTTTCGGGTGAAAAGTTTTGTGGTTTCTACCAAATGACCACGCCGTTCCTGA

TGTTACGCGATCCAGAGTTAATCAACAGCATAATGGTCAAAGACTTCTCGTACTTCACAGACCATGGTGTAGATACTCAT

CCGTCTGTCAACATCTTAGCAAACAGCCTGTTCTTATTGAACGGCGATCGGTGGAGAACGATGCGACAGAAGCTCAGTCC

CGGGTTCACCTCGGGCAAACTTAAGGACACGCATGAACAGATCAAAGGGTGCATTGAGCAACTTATGAACGTCATCGACG

ATAAGTTAAAAACCTGTGATCACTTCGAACTGCGAGAGATAATTGCAAACTTCTCGACGGACGTCATCGGGGCATCGGCT

TTCGGTCTGAAATTGGACACGATAAAAAACGGAAATTCAGACTTCCGCAAGTTCGGCAAGAAATTATTTCAGGGAAACTT

AAAACAGCTTTTTATCCAGGCTCTTTTGCTAGTATGTCCAAAATTGGTGTTTGCTCTAAAGCTAAAACAGTTCCCGGAGG

ACGTGTCTGAATTTTATGGATCTATGTTCAAAGAAGTCCTCGAATACAGGAGCCGGAACAACGTTATCAGGAACGACGTC

ACGCAAACCCTGATACAAGCTAGGAATGATTTGGTGTTAAATAACGACTCAACGTCCGAAAACAAATGGGCCGAAATAGA

CATAATCGCAAACGCGGTACTGATGTTTGTCGCTGGGTCCGAAACGGTTTCTACCTTGACATGTTTTTGTTTGTACGAGC

TGGCGTTGAACAAAGAAGTCCAAGATAGGCTGCGCGAAGAGATCGTGACGACAAAAGCGAAATACGGAGGAAATTTGAAC

AACAACTTTTTGACTGATCTCCGTTACATGAACATGGTTTTAGAAGAAGCATCGCGTAAATACTCCATCACTTTAATCAT

ACTCAGACAAGCAACAAAGAATTACAAGGTACCCGGTCAGTCGTTAGTCATTGAAAAGGGACAAAAGATCGTCATACCAA

TACTCGCCATACATAACGATCCAAAATATTATCCCAACCCCAACACTTTTGATCCTGAAAGATTTTCCGCGGAAGAAAAA

TCTAAACGACTCAATGGCACTTACATACCGTTTGGCGACGGACCGAGATTGTGCATAGGTAAACGGTTGGCGGAATTAGA

AATGAAATTTGTTTTAACGGAAATATTATCGAAATATGAAATATTGCCGTGCGAAAAAACAGAAATACCTCTAATCATAG

GAAGTCCTGGAAATATCGTCAGTCCGAAAAACGGCATTCATTTAAAATTTAAACCGATCGTTGTAAATTAACATTATTTA

GCATAATATACCAACAATATCCATATTGATATGTCCATTTTTTCCAATATTATATATAATGCTATTTTTAAAATTATAAA

ATATAATACTATTCAATACTACAAAAAAAA

>contig_6872

TAATAATATAATCCACTTTAATCTTCCACTTATTAACTTTACCTAATAAACTATATAGTTTATTAGGTAAAGTTAATAAG

TGGAAGATTAAAATGGATTATATTATTAAAAAAAAACAAAAAAAAATTAAATAAAATACCTACATATATTTGAGTTCTTC

ATTCTACATCTATTATGCTATTGATACAGACGATATTGAAATATAGTCAGTATAGACCTAAAAAGTATTAACATCATTCA

CAACTACTATCAAAAATAAAAAAAAGAAATTGAACATAATTATATGGTTTTGAATGGAGATAAATATAATTTAATTCAAA

CATGTGTTTTATTATTTTAATAGTCATTCTAGTTATTTAATTTTTGGCCAAACGATTTGTTCAATCCATGATATGTATTT

CGATACCCGAGTATAAACAGCAGGCGAATCCTTTTCTCCACAAGATGCTCCCGCAAATGATGTTATTCCTATTTGTGAAT

ACATACACATGTATTTATTGTGTTTTATTTGAATCGGACCACCTGAATCGCCTGTACAAGTATCATTTCCACCTTCTGCA

TTGCCAGCACATACCATAAGGTCATCAACTATTCCTTGTGATAATTTTTTATTTCTATTTGTCAGGTATAAGTAACTTTT

ATCGCATTCTTTAGCCGGAATAGTGTCTATACGTACTTTTAATAACTGTGAGCTAGGAAGTGAAGCTACTTCAGTTCTTC

CCCATCCTGTAGCTATTATTGCTGAGGGTCTTAAAGAATGATCTGTATTGAGACAAATAGGTCGTACAAATGCCGAGAAT

TCTACATCTCTTTCTAGACGAAATAATGCTATATCATTGTACAAAGATGGTGGTTTGTAATTTGGATGTACTATGCGTTC

TACTATTTTATAGTCTTTAGGTCTAGCCTCGTCAGTTTCCGAGATATAATCTAAATCACCTAAGCGGACCCATTGTGCGT

ATGTCTGTGTAAGTTTACTGCCAAATTTTTCACAGTGGGCTGCTGTTAGTACAAAATTCTTACTTATCAACGTTCCTCCA

CATAACCACGAATAGATGTCTGGTTTTTCACTATAACCCAACAAAGCCATGTGAGGAAATTCTTTTGGTTTAGCTTCTGT

GCCGTTGGTAATTAAAGTAATGGCATCATAGCAATCTTTAACTTTGAGGTAATTATTCTGATTTGATATTAATATTGGGT

CTTTAATCGTATAATAAATTAATTCTGAATACTCAGTACACTTTTCAGTGGCTGAGTATGCTTGCATCACACTTGGTTTT

TTGGTTGTAGAAGTAGATGTAGATGGATTAACGGTAACGGTTTTATTTTCTGGTGGACAACACACTATTGGAACTGATCC

ACTGAATGAGCATAATTGTGGTATAATGTTTTTCTTAAGTCCTTCCTTTGCTACCGGACAGTTTTCAATGTTCCGACAAA

TGGAGTTGGAAGATTTTCCATCACCTTTGCGGCAAATATCTCCTTCATATAATTCCAATATTTGTGCATTGTATCCCAAA

TTCATAAGACATAATATTAACACCGTCAAAAAATATTGTAAGGTTGTATAACTTCTCATCATTAATAAAATAGTTTTAAT

ATTTTATTCTAACAAAGAGTTTATTAATATTTTTGTTTACAATAATCAACTAAATAATCTTACAG

>contig_6922

AAAAAGTGACATTATATTTTATTATCCCTCTACATAAATTAAAAAAATTTACACACGATAACCAACATAATAATATTCAG

CAAAAATTTAAAAGTTAAATAATTAATAACATTCGAATAATTTTTATTAGTAGGTATTAAAGGTCTATAAACTAAAATAT

AAGTTAATATTGTTAAATTCAGTACAAATTTAAAAGCATTAAAGTTCATTTTTTTCTGATCGTTGAGCTTTATTATTCAA

TCCAGTTAACAATGGCAAATTATCCCAAAATTCATATTCGTCATTATACGGTCTTTGTTTAATTACAGGGGTACTTGTAT

TAATAAAAACATATTCATATTTTTTGGTGGTGAACTTCGGCCATTTTTCTCCTTTTTTCGAACCATCAATTGTAATGGTG

TTGCTGGAGGCGAAATTTGTCCATATGTTCACCATCAGATTTGATACACTGAGGTCCTCTCCCGCCAGATCTCCACGTTC

CGCGGTATAAAATAAACTGGTTATTTCATCAGCATGCGTCACGCCTAATTTTTTAGGAAATGGATATGGTCCATATACTG

AATTAAATGATATATAATTCAAATGATCATATAAGTAATAGTACACCGGCCGCGATAAATTAATTGCCATCTTAAAGATA

CCGTGTAAAAAAAGACCACCAGAAATCATTTTAACAGCATTCAACGGATCATTCAAAGTCCCATCAGGAAAGTATCGTTC

GAATACCTTGTCACCAATTACTGAAATATCTGAATACTTTGTCGTATACCTGTATTCTAAAAACGACGAAACATAATGGT

CGAAATCTTCCTTCAACTCAGTATACACAAGATCAGTTGCGTTGTACATTCGAGAGGCAAAAAGACCACCCTCTCCAGTG

TTCATTCCCATCAAAACTGGAACTTTCGATATTTGTTTAAAATCAAGCAAAGGATATTTGCATAAAAACGATTCTTTTTT

TTTACCACAATTTTCAGCAACAGGCATGAAATTAATGATTGGATAATTTCTCCACTCAAAAAAACTGTTATAAATGTTCA

CCAAAACCTTTGCAGGCAATTCTTTTAAACATTGAAGCATTTGTTTTGGATCTTCGGGGCATCCAGCAATAGTTGAAACA

GCTGAAGCTCTTCGTTTAGCATATCCCGGTGGAGTTACTCCCCAAAGATTTAATGGCGTTGCACTTTCCATTATTGCTTT

TTGAAACAATCCTTTGCTCATTGGAGATAATAAATGTAATCCAACAGAAGAACTTCCAGCACTTTCTCCAAATATCGTAA

CTTTTTTTGGAGAACCGCCAAATTTATTAATATTTTTTTGTACCCATTTTAAAGATAAGACTTGGTCTTTCATACCATAA

TTTCCTGGCAAATCATCGTTTTCGGTACTTAAAAATCCAAGAATTCCGAGTCTGTAATTCATAGTAACTAGAACTACATC

TTTATCTAACAAATATTGTGGACCGTATAAACTTTGACCACCTGAACCCGCAAAATAACCACCACCAGCAATCCACACCA

TAACTGGAAGCAATTCTCTATCACCTTTTTTTGGTATTTTTGGAGTGTACACATTGATATACAGGCAGTCCTCTGAACCG

ACAAGAATATCCTCTTGTTGATAATAAAACAAATTCCTCTGAATGCATAGTTGTGGCTCAGTAGTAGCATTTAATACACC

TATCCACGGATCGGCTGGTAATGGATCCTTAAACCTAAGATCTCCAGTTGGTGATTTGGCATAAGGTATTCCCTGAAATG

CTTTGAATTCACGTCCATTTCTTGTCTTCATACTACTACCAATCAACTTTCCTTGGTCTAATTGTATAACTTCATCCGCA

CCAAATACGAATCCGAAAAGTAAAGTAAAAATAAAAAATACTTCCATGTTTCT

>contig_7140

TGATAATAATAATAATAATACCGTAAAACCTCCAACGTGTAACCGTCGGACCGCCGAAATCGGTAGTAGTAATAATAATA

TCGATGCCAACGGTACAGTTTAGCCGATGACACCGTTGTGATAGACACGCGCGAACAACAGTTTCGAATCTCGCCAGTTT

GAGAGCGGCACGCGTACTTACAGACAATCGAGAGCCGTCGTGAGCAGGTCGTACCGCAGACTCGTCGTCCGCCCGAACGG

TCCAGCTGTTCGGCATCAACATGTCGTCGCAGTCTCGTCTGCCGCTGGTGGTGGTGTCCGCCGTGTTCGTTCTGCTGTCG

GGCCTTGCCGATTTGGGCAGCTGCAACCAGGTCGTGTCCCGGTTCGAGTACAAGTACAGTTTCAAACCGCCGTATTTGGC

GCAAAAGGACGGGTCCGTGCCGTTCTGGGAGTATGGCGGAAATGCTATTGCCAGTGCAGATAATGTTCGAATTGCTCCAT

CATTAAAAAGTCAAAAAGGAGCAATTTGGACAAAAAGTCAAACAATGTTTGATTGGTGGACAGTTGAAATAAATTTTAGA

GTCAGTGGAAGAGGTAGAATTGGTGCTGATGGCCTGGCATTTTGGTACACAACTTCTAAAGGCGATTATAATGGTCCAGT

ATTTGGTAGTTCTGACAAATGGGTTGGATTAGGAATATTTTTTGATTCTTTCGACAATGATGGCAAACACAATAATCCAT

ATATTATGGGTGTTGTAAATGATGGGACACAAGTTTTTGATCATGCTAATGATGGTTCTACTCAACAGTTATCTGGATGT

TTGAGAGATTTTAGAAATAAACCTTATGCGACAAGAGCCAAAATTGAATATTATATGAACACATTAACAGTATTTTTCCA

TAGTGGTAATACTAATAACGAAAAAGACTTTGAAATTTGCTTCCGAAGTGAAAATGTCTTCTTACCTAAGAATGGTCATT

TTGGTGTATCTGCTGCTACTGGTGGTCTTGCAGATGACCATGATGTAAATCATTTTTTGACATATAGTATTTACCCACCG

GGAACAACATTCAAACCAGCTGGTACATCTCAGCAGCCATCAGATGAAGAAAACAAGCTAAACAGAGAGTATGCAGATTA

CCAACGAAAATTATATCAAGAAAAAGAAGATTATCATAAGCAACATCCAGAAACTAAAACAAAAGAAGATAACGAGTTTG

AAGATTGGTATGAAACAGATAGTCAAAGAGAATTAAATCAAATATTCTCTGGTCAAAGTCAAATTGTAGATTCTGTAAAA

GTATTAAGCCAAAAACTTGATGAAGTAGTCGGTCGTCAGGAACGAACACTTAGTTTGTTATCTAATGTTCAATCTACTAT

GGCAGTTATTGGATCATTAGGTGGTGTACAAGGACAACCTAACGTACAAATTCCACAAGTAAATGCAGGAATGGGAAGAC

AAGATGTTGATGTATTGATAAACAATCAAAATGGTATTATCAACTCTGTGAGAGAAGTCAGGAACTTGGTGGCAGAAGTA

CATTCAAAGACTGAAAACATAATTAAAAACCAAGCTCATCAACCAACAGCTCAAGTTCAACCATTAGGCTATGATCAGGC

ATCAATTATACATGAAATCAGGGATACCCTGAATACAATCAAACGGGAATCTACACAGAAGGCTGGGCATTCACAACAAT

TATCATGTCCTACGTGTGCAACCAATACAATTGTTTTGGTTGCTGCGGTGGGCCAAACATTATTATTTATTCTCTACGCT

GTCTATAAGAACAGTAAAGAATCACAAGCTAAGAAATTCTATTAAATCTTGGAAATATCAAATAAGAGCAGAACTAAATA

AATAATACTATGTATATAATTAGGAGATTTCATTAATTATTACAACTGTGTTTAAATATAATTGATACCTCTTATGAATA

AGATAATAGTAAAACTTTATTTATTTTTTTGGTACAAGTGATTAATCTGCATGATAAATCCACCAAAATAAATATTTTTT

ACTATGTAATTAATGACTTATTATTAAATTAAGATAATTTTTTTTAAGGTTGTTTTACTTTTCGTTACAATAAGAATGTG

TTGTGATTTATAAAATAAAAATAATAAAATA

>contig_7195

ATAATAAAATTGAAAAATACGATGAGTTAATACTAGCAGTAGATGCTACATATGGATGTGCGCCTCCGACGAATGGCCAC

CACTTGCCTAAAGTTGATATCATATTCGTACGGCTTTCGATATCTATGTCCAAATCGTCACTCTGACTGGATTCATTTTT

TTTCACGTACACCGAGGCCAAATCCATGTTTTCTGAATTCAGTATTGACACCAATCCTTTACCGTTATAGTTTCCACATT

TCGGAAAACCAGGAGGGGATGGTGGCATGTCCCAGTCTTCACCGATCTTGAATACAGTGGCCATGCCCACTTCAACATGG

AACTCAATGTGACAGTGAAATAACCAATAACCAGGATTATCGGCTAAGAAACGTAATATTGTAAATCCACCATCTGGTAC

TGTCACTGTGTCTTTAAGAGGCGCAGTCCGCAGATTTCTTACAATGCGCCCGTCTCTGTCCATTTGTTCGATCTCTTCTA

CAGTAGTATGGTTTCCAACTCTTTCCATCGCAACTACTCGAAATGGATGTCCGTGCAAATGGAATGGATGATTTGCGTTA

TATGTAACACCTTTATCAATGAGAAATAGTTCAACGACTGACCCCAAAGGAACTCTTATAATATTTGTACATTCACAGAA

TTCGTTTGAGCAATCTTTCTTTATATGATCACAACTCATCCAAGGTTCTATCATTTCTCTTTGAGATAATAATGGAAATG

ATGGAAGCTTGAAAGACATTTTGTTAATTTGTGGTGTGTAAACTCGTTCAGACTTATGCGATACTTGTTTGAATCCATAT

AACATAGGTTTATGGAAATGTGGATTATCCAAAGAATAAAAATCATAAGACATAAATAACGTTACGTCAGGCTTTTTCTC

TAAACGAAGATCGTATTTTGGAGGTGTTGCGTCTTCTAATTCTGATACAGTAGCAGTGTCCATTAATCCTGATCCTTTAT

TCAGTGCATTCAATTG

>contig_7196

CGAACCTTCTCGATCGTTCGCTCGGATTATATAAACGCTTACAGCACGCTTGCTGGCGTCAGTTATATTTCGAGAGCGGT

AATAGTGCGATTCGTAATATTCATAACTTGTGCGACTTTCAGTGTTACACTCGTTTTATTTTCCATTGCGAAATTTAAAA

CACAGTCGAATAAGTGATTCTAAATCGTTATTGAAATCAAATTGCGGTGTTAAAGTATACACTTAAAACACATTTACTTT

TTAATTTCGTATTAATTGTGCGTAAATAAATTAACGGAAATGAGTGGAAAAACAGCGATCGGTATCGATCTGGGCACCAC

TTATTCTTGTGTGGGTGTCTGGCAACATGGAAAAGTGGAGATTATCGCCAACGATCAAGGTAACAGGACCACTCCAAGTT

ATGTGGCGTTTACGGACACCGAACGGTTAATCGGTGATGGAGCCAAAAACCAGGTGGCGATGAATCCCGTCAATACGGTA

TTCGATGCCAAACGTTTGATCGGCCGTCGTTATGACGATGATAAGACACAAACGGACATTAAGCATTGGCCGTTCAAAGT

GATTAACGACGGTGGAAAGCCCAAGATCCAAGTGGAATTCAAAGGTGAGCGTAAAGTGTTCGCGCCGGAGGAGATCAGTT

CGATGGTTTTGACGAAAATGAAGGAAATCGCGGAAGCTTACTTAGGCCATAACGTGACGGACGCTGTTATCACGGTGCCG

GCGTACTTCAACGATTCGCAGAGACAAGCGACAAAGGACGCGGGTGCCATAGCCGGGTTGAACGTAATGCGGATTATCAA

CGAACCGACAGCCGCAGCTCTGGCATACGGTCTAGATAAGAACCTGAAAGGCGAGAGGAACGTGTTGATCTTCGATCTGG

GTGGCGGCACCTTCGACGTTTCAGTCTTACAGATTGACGAAGGTTCGATATTCGAAGTTAAGTCCACGGCTGGCGACACA

CACTTGGGCGGCGAAGACTTCGACAACCGCCTGGTGTCACATTTGGCCGACGAGTTCAAGAGGAAAACCAAAAAGGACGT

GCGTGCCAATCCGAAAGCTTTAAGACGGTTGAGGACGGCTGCCGAACGAGCCAAGAGAACTTTGTCGTCCAGCTCAGAGG

CGACCTTGGAGATTGACGCTTTGGTAGACGGTATCGATTTCTATACACGAGTGTCCCGGGCACGTTTCGAGGAGCTGTGT

GCCGATCTATTCCGGTCGACCTTGCAACCAGTGGAGAAAGCATTGGCGGATGCCAAGTTAGACAAAGGAGACATAAATGA

TGTGGTACTCGTGGGTGGTTCGACGAGAATTCCGAAGATTCAGAATCTCTTGCAAAACTTCTTCTGTGGTAAGCCACTCA

ACCTGTCCATCAACCCCGACGAGGCAGTAGCATATGGCGCTGCGGTGCAGGCGGCTATCCTTAGCGGTGACAAGAGTTCT

GCAATTCAAGATGTATTACTCGTGGACGTCACACCGTTGTCGCTAGGCATCGAGACCGCAGGCGGTGTTATGACCAAAAT

CATCGAACGCAACTCTACCATTCCATGCAAACAAACCCAAACTTTCACCACTTACGCGGATAACCAACCGGCCGTCACCA

TCCAGGTGTTCGAAGGAGAAAGGGCTTTGACAAAGGACAACAATCTACTAGGAACGTTTGATCTAACAGGTATACCTCCA

GCGCCCAGAGGAGTACCCAAAATCGAGGTGACTTTCGATATGGATGCCAATGGAATTTTGAACGTTTCGGCCAAAGACAA

TAGCTCCGGGCGCTCAAAGAATATCGTCATCAAAAATGACAAAGGCCGTTTGTCTCAAGCCGAAATTGATCGTATGCTCA

GCGAGGCCGAAAAGTATAAAGAAGAGGACGAACGACAGAAAGCAAAAATCGCTGCCAGAAACCAATTGGAGAGTTATGTA

TTTGGAGTTAAGCAGGCATTGGATGAGGCTGGCGACAAGTTGACCGAATCTGAGAAAAATAC

>contig_7197

CACTGGACGACCGCGTCGCATTCCTGTTTGCCGGTGTTCTTCTCGGATTCGGTCAACTTGTCGCCGGCCTCTTCCAACGC

TTGTTTAACACTGAACACGTAGCTCTCCAGCTGGTTCTTTGCCGCGATCTTGGCCTTTTGTCGTTCGTCCTCTTCTTTGT

ACCGTTCGGCCTCGCTGAGCATACGATCGATTTCGGCTTGAGACAGGCGACCCTTGTCGTTCTTGATGACGATATTCTTG

GAGCGGCCGGAGCTGTTGTCCTTGGCCGACACGTTCAAAATGCCGTTGGCGTCCATGTCAAAAGTAACCTCGATCTTGGG

TACGCCCCGGGGCGCCGGAGGTATGCCCGTCAGGTCGAACGTTCCTAACAGATTGTTGTCCTTAGTCATGGCCCTCTCGC

CTTCGAACACCTGAATGGTGACGGCCGGTTGGTTGTCCGCATACGTCGTGAACGTTTGGGTCTGCTTGCACGGGATAGTG

GAATTGCGCTCGACGATCTTGGTCATCACTCCGCCCGCGGTCTCGATACCCAGCGAAAGGGGCGTGACGTCCACGAGTAA

CACGTCTTGAATCGCAGAACTCGTGTCGCCGCTGAGGATGGCCGCCTGCACTGCGGCGCCGTAGGCCACCGCTTCGTCCG

GGTTGATGGACAGGTTCAGCGGTTTGCCGCAGAAAAAGTTTTGCAGCAGACTCTGAATCTTCGGGATCCTCGTCGAACCG

CCCACGAGCACCACGTCGTGTATGTCTCCCTTGTCCAACTTGGCGTCCGCCAACGCCTTCTCCACCGGTTGCAGAGTCGA

TCTAAACAGATCTGCGCACAACTCCTCGAAACGGGCGCGGGAAACTCGCGTGTAGAAATCGATACCTTCCATCAGAGCGT

CGATCTCTATGGTGGCCTCCGAGCTGGACGACAACGTTCTCTTGGCCCGTTCGGCGGCCGTCCTCAACCGTCTCAACGCC

CTCGGATTGGAATGTACGTCTTTTTTGCATTTCCTCTTGAACTCGTCGGCCAGATGAGCCACCAGCCGGTTGTCGAAGTC

TTCGCCGCCCAAGTGCGTGTCGCCCGCCGTCGACTTCACTTCGAATATCGAACCCTCGTCGATCTGCAGAACGGACACGT

CGAACGTTCCGCCGCCCAGATCGAATATCAACACGTTCCTCTCTCCTTTCAGGTTCTTGTCCAGACCGTACGCCAAAGCC

GCAGCTGTCGGTTCGTTGATTATTCGCATCACGTTCAGGCCGGCTATGACGCCCGCGTCCTTTGTCGCCTGTCTCTGCGA

GTCGTTAAAGTACGCCGGCACCGTGATCACGGCGTCCGTCACTTC

>contig_7254

ATATTGCCGCTAAGAGTCATTGGAATTTTATGAGTGCAGTTCTCCAATCTTTGTCGGATAATGGACATAATGTTACCGTA

TACACTCCATTCCTCGCTGGAAATCGGGCTAACTACACAGAAGTCTATTTAGAATTGCCATCAAGAGTCGGATTGGAGGC

CTTAGAATCAATAAATAGTCTTGGAAAAGCTACAGTGATAATGCCTTTGGGCATGAATATGACTCGTTATTATTGTAATA

TTATTCATGAACAAACGGATATACGAGAAATATTAAGAAGCGGTAAATCAAATTATGATATTATTATCACTGAACTTTTG

TCCTCGGAATGTTCATCTTACGTCGCTTTCAAGTTAAATTTGCCACTTATATACGTAATTCCTTCGTCAATGATCACATA

TATGGAACATGCTGTATTAGGGGATGTATCTAATCCAGCAACAGTCTCACATCTGTTAGCTCATCACGCGGTACCTAGAA

CATTTGTTCAACGATTTACAAACGCCATCCTTTTGGGTTTTAGTTTGATTGCATTATTGTATAAAGAAATGGAGCTGAAA

AAAATTGATGGTCAACCATATGATCTAGTGAAACCTATAAAACCGTCGTTGATATTTATGAATTCAGATTTCATTACTGA

AGGGCCAAGACCTATGCTTCCAAATGTCATACAAATCGGAGGAATACATCTGAAAACACCAAGTAACATACCAAATGATA

TACTGGGGTTTATTGAAAACTCACCACATGGAGTGATTTATTTTACGTTTGGTTCAGTCATTTCAATGTCAACATTACCA

AATCATATTCAAAATGCGTTTAAAGAAACTTTTGCACAAATACCTCAGAGGATATTATGGAAGTACGAAGGAGAAATGAA

AGATAAACCGATTAATGTAATGACGAGTAAATGGTTTCCTCAGCGTGATATACTTCTGCATCCCAATGTAAAATTATTTA

TTAGTCACGGAGGTATATCTGGTGTATTTGAAGCTATAGATGCAGGTGTTCCTGTTCTCGGATTTCCCTTATTCTACGAT

CAACCTAGAAACATAGAAAACTTAGTTGATGCAGGAATGGGGATTTCAATGGATCTGTTAACAATTGAAAAGGATGAATT

GTTAAAAAATATTTTAGATCTCACTAATAATGAAAAATATATGAGAAACGCTAAAATCAATTCTGAAAGATTTAAAGACC

GACCGATGTCTCCAGCAGAATCAGTTGTTTACTGGGCAGAGTATGTAGTTCGTCATAAGGGTGCGCCACATTTAAGATCT

CATGCGTTTAATTTGACGTGGTATCAATACTTTTTATTGGATGTCATTGTTGTTTTATTATTATTTATTTATTTTGTTAT

TTTTATTACTTGCAAAGCTTTCAAATTTATTTATTATTACTTTTTAAAATATTCTCAAACTGTCAAACCGAAGTTAGAAT

AATTTTAGTACTATTATGGTATTCAATTTTTTAATTAGTTTTGTAATAAAATAAATTTATCACATCTCTAGCTGTTAAAA

TC

>contig_732

GATACGCAAATTATTAAATCGGTTAACGAGAAATTCGACTGTAATTTTATTGTCTTATAATTCGTATTACAGTCGTGTGA

TGGTGTGGATATTAAATTTTATGTACAATAATTTATTGGTTACAAAGAATGGAGCAAACGTGGTTTACAATGATTATTTG

TTTTATATAAACAAAAAACATTTTGGAATACAATTTTTTTTTAAGTTATTTTTAATTTTACTTGTATGCTTATATGTTCT

CGCTTCGTTATTTTTTTTTTAAATTTATTATATTTTTTCGGTGCAGCGTGGACACGTACAACTCTATTGCAATTATTGTT

GACCATTTCTGGAGTTTGAACGTCTCATCGTTCGGCTTTTGGTAAGGAAACCAGTTGCAGATTTGAATGAGAAGTTTTTG

CGGAAGTTGATCACCTTGGATATATTATTGATCACCGTTTCAAATTCGGTGTCGAATTTGGGATGTGCCAACAATAAATT

AAAGTTATTTAATACCACGTCTGTTATCAACTTGGTGAACGGGAGTTTATCAAAACACATGTTTACGCCCTTCATTGACT

CGTGCCAAACGACACGGAACTTAGGGTCCTTCATGATCTTCCTGGCCCAGTAGAATACATTTCCCAGAGTTTGCGAGACA

TTGGTGTTCGTCGTCACGTCGGTGATCGTGTCGGCTAGGAAAACGATTGAATTGTGGGCTTTCGGGTTCAAGCAAATATC

CTTTAAGTTCTGTCGCAATTCATTGATTGCTTCTATGCCTACGTCGTTCTCGAGGATCGAAGCCAAATTTTCGTGCAAGT

GATACTTTACGTCACAACTGTCTTGTCCGAAGAACGAATCGTGTGCAGACGTGTTGGCCGCAAAAAAGGCCAAAACGATT

GACACGGTGATTACGGAATAGCTCTTCATTTTCCTGGAAATCAAATTCTATAAAAGATAAATAAGTGTATTTTTCGTTAA

AAAAAATGCATTGATTCAGAAAACGACTATAATGCTTGGGAGATCTAGAAATTTATACTCGACGCGCGCCCCC

>contig_7391

CGTCACCAGATCACCGTCAAACGGTCAAACCGAATCATGCAGTCGCTCAGGATGGAGTTTGTGTCCTTTAAAATGTTTAT

TATTGCCTTGTCCCTGATGGGTTCGGTTGTTAATTCGAAAGAAACTTCCGAATTTAAATTACCAACTAATTTTAAGCCCG

TCAGCTATCGACTGGATGTGACCACACACTTGGATGATAAATTCATGTTTGAAGGAGTAGTTGACATTAAAATGACCTGC

ATAGCAGCTACTGACACCATAGTACTCCATTCGAACAGTTTGGACATCGATACTAAAAGTGTCGTAGTGGCTAACAGTGG

TGAAAATGTCGTCCCAGTGGGTAGTGTTACGTTTGATCCAAAGAAAGAACTTATGTACGTCAAGTCAACAGAAAACTTTA

AGCCTGGTGACGAGTACGTGCTGACTATACCATTCACAGGAAATATCACCGACGATTTAGTAGGTTACTACAAAAGTAGC

TACGTGGACAAGGAAAACAATCAAACGAGATGGTTAGCGGTAACACAATTCGAACCAGCAGACGCCAGACGAGCTTTCCC

CTGTTTTGACGAGCCTGCGTATAAAGCAACATTCAAGATAAGATTGGGTCATAAAAAAGAATATACTTCGATTAGTAATA

TGAAGATGATGAAACAAACGCCCATCCCTTCACAACCAGATTATGTTTTCGATGAATTTGAAGAATCTGTACCGATGTCC

ACTTATTTAGTAGCATACATGGTATCTGATTTCGCATACATTGAATCTGACAGTCGGGACGACGAAGTGAAGTTCCGTAT

AATAGCCAGGAAAGACGCAGCCGATCAAACAGAGCTTGCTAAAAACGCTGGACCATTGGTATTGAAGTACTATGAAGATT

ATTTCGATGAAAAATTTCCGCTGTCCAAACAAGACATGGTGGCCATACCAGATTTTTCTGCCGGCGCAATGGAGAATTGG

GGTCTTGTCACTTACAGAGAGACTGCATTGTTAATTGATCCGGACGTAGCTACGATTGATAATGTACACAGAGTAGCCGA

AGTTATCGCTCACGAACTTGCTCATCAATGGTTTGGTAATCTTGTTACCATGAAATGGTGGACCGACCTTTGGTTAAATG

AAGGATTTGCCACATACGTAGCAGCACGTGGAGTTGATTTCTTGTACCCCGAATGGAACTCTTTCCAAATCGAAACGGTT

CAAAATTTTTTACGCGTTTTGGATCTTGATTCGCTTCAATCGTCCCACCCTGTGTCGGTAGCCGTCGGACATCCTGATGA

AATAGCACAAATTTTTGATACAATATCATACACTAAGGGTTCATTTTTATTACACATGATGAACACTTTCTTAGGCGAGG

ATACGTTCAAACAAGGCATTAGAAATTACATAAACAAGCACAAGTTTTCCAACGCTGAACAAGATGATTTGTGGAATTCT

TTGACTGAGGAAGCTCACAGACAAGGAACTTTGGACAAAAATCTAACAGTGAAATTGATAATGGATACATGGACGTTGCA

AACCGGTTACCCCGTGTTGAAAGTCGTCAGGGATTATTCTGCGGACACGGTTACATTGTCACAGGAACGATTCCTAACTA

TCAAATCAAACGGCACGGATAATAAAAGTTGTTGGTGGATACCACTCACTATGACGACTTCAATGGAGGCCGACTTTAAC

CAAACAAAAGCAAAATCTTGGTTGAATTGTGAAAACAATAACCTCACTATTCCTTTGGCTAAAGACAACGAATGGGTCAT

ATATAATATGCAGATGGCCGGTTTATTCAGGGTTTTGTACGATACGCGAAACTGGATGGGTATCGTTTCTACGCTGAACG

ATCCAAACGAATATGATACTATACACACGTTAAATCGAGTACAGTTGATCGACGACTCATTTAGTTTTTCTCAGATTGGC

GATTTAGACTATGGAATTACGTTTCAATTATTGAATTATCTAAAACACGAAAAAGAGTATGCACCGTGGTTGGCTGCTTT

GGGTGGTTTGGGTCCCATAAATAGTTTATTGAAAAGGACTCCGAACCAAGGATTGTTCCAAAACTATATGCAACGTATGC

TGTCGCCTGTGTACAGCAGATTCCGAGACATGACCGTCGAACTTAATAATTTTGAAGAAATACGTTTTAAAAATCTTGTG

ATCGCCGAAGCCTGCCGTCACCGAATTAAGGATTGTACCGAACAAGCTCTCGATTTGTTTAGCAGGTGGATGAAAACTAC

AGACCCAGACAAAAATAATATATTGCCTAGAGAACTGAAACCTGTAATTTACTGTCAAGCGATAAAATACGGAGGTGTAG

ATGAATGGGACTTTTTATGGGAACGTTATCAACGTTCTAATGTGGGATCTGAAAAAGCGAAATTACTCTCAGCATTAGGA

TGTAGCTCGGAAACTTGGTTACTTAACAGATATTTGAATTGGTCTCTTGATAATTTTATTATTCGAAAACAAGACGCAAT

CTCAGTTTTCTATTCTGTGGCGAGAAGTGACATTGGATTTTATGTAGCAAAAGACTTTTTGTACCGCAAAATTGCTGATA

TATCTGAATACTTCCAGCCCCGAGGCGACCGTGTAGGCAGTTATGTAAAAGCTATTGGATCTCAAATGAAAACTAAAGAA

GAACTAGATGAGATTCAATCATTTATTAATAGATCATCAAATTATCTAAAAGGAGCTGATTTAACTACCAAGCAGACAAT

TGAGACTGTTAAAATAAACACCGAATGGACTACGAAATTTGATCATAAAATTATAAACCACTTGGTATAATATTAATATT

TATGTTACTAGTAAGTCTGAACAGTTTGATTTGCATTATAAAACTGTACATTTTTCCACTGACTAAAAAGCTATACTAAC

TATATATTATGTACTCCTATAAAAGATAAATTAAATTATACACATTTTTATCCATATCATGTTTCATAATATGACGTAGT

TACTACTTTTATCTTGAGCTGTGTTACAAGATGAAAGTTGCAGATCACGATAATGTGTTATAAATCTGAACTGAAATTAA

CTCGTCATATTTAAATATTCAATCGTCGTCAAATATTTTAATTTATTTTATAAAACTTAACATAATTCTATTTTTCTATG

TACATAATTATATAATTGTGAAATGTTATTACTTTTTTGTTTTATAATAAATACCTAATAACATGAAAAAAAA

>contig_7508

TACAATTAATTCTTAATAAGTATTTTAATTTTAATATTTAAGTGTTTTATAAAGAAAGGATGGGCAACTGGCGGACAATA

ATAACTTTTTTATAAAGAAAGGATGGGCAACTGGCGGACAATAATAACTTTTTTCCTGGCTCATGATACATTTTCAAATT

ATTTTATAACATTTTACAGTTTATATATATTTTATTAAGTTTTTTAGTATTAAAACATTGTAAATATAAAAAGTAATATT

ATAATATAATATAATGTCCATTGTAAATTTTACTTTCTTTCAATGTAACTTTAAGTTGCCCATCACTATTATAAAGGTAC

CTACTTTAAATTATAAATATAAATAAAATTAATTATTCAAATTTTGATGTGCATCATCTCTAGTATCAAAATCATTTAAA

TCTTCATCGTCTTCATCTTCTTTGTTATGTTCTTGTTCATACTCATATTTATAGGAATTATTTTTTTGTTTTGATAATTT

TTCTTCTTCATTACTATACTTATTTAATGGATCTTTTTTAACAGTCTCTTCTTGGGGTGGAGCAGGCATAGCTTTCATGG

ATTCATTTCCACCAACTTGAGATTCTTGAGTTGAACTAGTTTCATTTAGTAAGACCAAAACATTATTAGATACTTTATCA

GAACTGATGTCTAAAACAATAGGTTGATTAATTGGATTGGTTTCATCGTTTTCAATAGGTAATGATTTGTTTGTATTAAT

CACTTTTGAACTTATCATTGGTGTTGAACTCTTAGCAGTAGCCCCATCAATTGCTTGTGGATTAATGGCAGGAGTTACAG

AACCACTTTTTAATTTTGTAACATTCATAACATCAATTGATTTCTGAATAAATTTTATGTTTGAAAATTCATTTGATTTT

TGTAAGTTATTAATTTGGTCATTTTCTAAGTTTTCTTTTCCACTTTCTAATTTCACGTTTTCATTTTTTAAATTTTGTAT

TTCGTTTTGTGCATCATTCAATTCTTGAGTAAGTTGTTTATTTTTCTTTTCTTCTTCCAAATATTGTAATTTCAATTTTT

CCAATTCCTCTCTTAAATTTTTTTCTTCTTCCAGTAATACTTCATGATTTCGTTCTAACTCGTTATATCGATTCATAGAG

TC

>contig_7518

GTCATCTTCAAAAATGACAATGGGTCATTTGCTTCGTTCTTTGATTATCATCGGCTTGGCTATTCAGCTTTGCAATGGAA

CAAGTTACTGGAGTAAACCAAAACATAAAAAAGTATGCATCGCCTCATGTTCAACTAGTAGTTCTTCCTCGACTACTGTT

AGAACTTCGTCAAAATTCAATGTTTTAAATTCAAAGAATAAAATTGAAGACCTTGAAAAAGGTCTTAAAAAAATAAGCAC

AAACTCTCAAGAAGACAGCAGTGAGTACGATGATGAAAGTTGTGAGGACTTAGATTACAGAGATTCACAGTCATCACGAA

AATTACAAACCTTTGTACACAGTCGTGCTCTTTCAAAAAAAATGAAGAAAACACTAAAAAAAACGTCTAAAAAGGTCTTA

AGAAAAAAGATTACCATTCCTTTAATCGCGAACAAAATTATTGGGTCTGCTATAACAAGATCCACTAGTAATGGCTTACA

ACAATCGTTACAGCAAAATCAGCAAATTGGTAGTACAAGTAGTACAACAATTACGTCTAGAACAAACACCGTGGGTGTCA

ACGGAAACATATTTCTGTCAATACCAAAAGGATATGTAGCAAACACAATGTCAATGACAACAACTAGTTTATTAACTGAA

CAAGAAGCTATTCGATTAGGACTCTTGAGTAACACTCTAATTAGTGGAAGTCAAACGTATACATCTTATCAAAATGGTGG

ACAATTTAGTTTATCTGATAGCGCCGTTAGTAGGTCTATTAATAGCCAGATGTCCCAAATGCAAAACCAAGGAAATAGAT

ATTATTTAAATCGTAATTTACAGAATACCGCAACATCAGGTAATTTAAATAGCATTAATGAAAATTCACAACAGTTTAGT

AGATACACATCAGTAAACGATAATATGAGTAATAATCATCAACAAATCAATGGTCAAAAAACTATGGGTAAATTGTATGG

ATTAAATAATCAACAAAACAATGGCCAACAAACTGTAGGTAGATTATATCAGTTTAAAAAAACTAGTTCATTGCACCGTA

AAATTAATCAACTTCAAACATCAAATAATGTTGTAAATAAAGATGAAAATGGTAGTGAAGATAATGAATTGAATGATGAC

AATAACCAAAAAGTAGGTATCACAAATGTTAAAACTGCCGGACAATACTCATTTAAATCATATAATAAAAATCAATTAAA

CAAGAATATTAATAGTCATCAAATAAATAACTTAAGTAAAACAATCAAGTCAAGACATATTCAAAAATACAGCATTTCTA

ATAAAGGTAATAGTGATAAATCAAATGAATCAAAATCTTCCGATGACCAAAATGATGACGAAAACGATAAGTTAAAATCA

ATTACTAAAAAGTCCGTAGAGAATAACGATGATGAAAATAATAAATCTGAGGATAATGAAGACGGGGATGACAAATCTAA

CGATAAAGATGATGAAAATACTAAAATAGAAAATAATGAAGATAATAAATGTAATGATGATAAATCTAAGGATAAAGATG

ATAAAAATAATGAAATAGATGATAATGAAGATAAAGATGATAAATTGGAAGATAATGATGATGAAAATGATGACATTGAT

AATGATAATAGCGAAGAACAAGAAACATCAGAAGACGAAGTATCAGTATATGAATATTTGGATACATTTAGGAAATTCAG

ATTTGATAGTGGTCTAAATGCAGTTGGTATAATAGGAGAAGTATTAAGTCAATTAGATTATCAACGTTTTATACTTATGG

ATGATATTATATATCCACAAGACTCCACGCGTTATGTTATAAACAACGAGGAGTTCGATTTTATCATAGTCGGTGGTGGT

AATGCAGGGTGCGTTCTAGCAAACAAGCTATCTGAAAATGAGAAATGGAAAGTATTATTAATTGAAGCAGGTGGAGATCC

CTTCCCTGTAACTCAAATTCCTAATTTATGGGATAGAACATTAAATAGTGTTGCAGACTGGCAGTATAAAATACAACCGG

ATAGTACAACTGGATTTGGAATTGAAGGTAATATGAAACTACACAAAGGAAAATGTCTTGGAGGAAGTAGCACTACAAGT

CCTCAAATTTATGTTAGAGGGAGTGAAAAACTCTATAATTCACTTGTTAAAAAGGGTTTGAAGAATTGGTCGTACAATTC

AGCGGAGACTATCTTTAAAAAGGTTGAAAGAATCCGTTCAATCACAAAAACTGAAACAAACACTACTATTTATGGAAAAT

GTGGACTTATTCCTGTAAGCAAATTCCGAAAAAATGAAATAAGCGTTTTAGAAAAAGTTGTATGTTCTGGGTTTGAACAT

ATCGGTTGTAAAAAAGAAAGTGATATCAACGAAAAAGATATTGAAGTTGGATTTGTTTCAATGCAAGGAATAATTAAGAA

TGGTAGATCTATGAACACTGCTAAAGCATATCTAAGCCCAGTTTTCGGCCGCGAAAACTTAAAAGTGATGAAATATACTA

GAGTTACAAAGATTTTAATTGACGAAACAGAAATGAAGGCAACCGGAGTTGAAGTACAGACAAAATACGGTCAAACTCTT

ACACTAAAAGCCAACTTGGAAACCTTACTTTGTGCTGGGGCTGTTGGTTCTGCTCAGATATTGTTGGCTTCAGGTATTGG

GCCTAAAAAGCATTTGTCCGAAATGGAAGTACCGGTGGTTAAAGATTTAAATGTAGGGCAGAAATTCTTAATTACTCCAG

TGTTTGCTGGTTTTGTTATGTCCTACGACAAATCAATTGTTTGCAATCAGACTGATGAAGAAATTGCATTCAAATACTTG

GCTAGACATTCAGGAGTTTTAAGTAGACCTAACGGTATGAGCTTTGGTGGATTTTTAAATACTGGTATGTCTGGATCTTC

ATTCGCTGATATTGAAGTCTTCCAATATTACATTCCCAAAAACACTTATTCAAGGTTATGCCAACTAAAATCAATATTTG

GATTTAGTGATACTTTGTTATCAGTGTATGCTAAATTGAATTATGAACGTGCTATTTCTATTTTTACAATAGCTTTGATA

AACACAAAAAGTACCGGTAAAATTCTTTTAAGAAGTACGAACCCATTGGATAGTCCGGTTATTGTTGGCGATATGTTGAC

CGATAAAAGTGATATAAAAAGTTTCCTTGAAGCCATCAAATTACTGTCCAAAATTGAAAAATCAGATGGAATGAGGTTAG

TAAACGCTAAGATAGAAGATATTGTTCTTGACGGATGTGCAAAATATAATCCAAAGACCAATGAGCACTGGGAGTGCTTA

CTAAAATACATGGTATCTACAACATCAAGTACTGCCGGATCGTGTCGTATGGGAATTGAAACAGACACAGATGCTGTTGT

TGATAGTGAACTGAACGTGATTGGTATTTCTAGTTTACGAATAATAGGTCGAACTATTATGCCATTAATTACTAGCGCCT

ACAGTAATGTTCCTTGTATTATGATCGCTGAAAGGGCATATGATATGATAAAAAGTAAATACGTCTAAAAACTAAACTAC

TCGATTAAATATTGTTTTTAAGAATTTAAAGCACTGTGTTTACATTATAACTATATTCATTAAAAATCAGAAGGAATCAA

GTTTTCTTTACGAAAAAAAACAAATAAAATTCTAAGCACCTATACATGTTTTGTTTTTGTTTTATAAATATCACGGTAAC

CTTAATGAAAAATTGGTTTTATTATATACCTAATTATTTCCTCGTTATTTATACAATCGCTTTAGTAAAATACTTTGTTT

GAAAGAATTTT

>contig_774

TTTTTTTATCAGCGGTGGACAACGCCGCACGACGCACATTTTCTCGTCACTATTCGCACAGTCAAAGCAGAAGTGGTCGT

CGTAGTTTCAATAGTGAAATACACCCGTTAGGTTTTTTTTTTTCAGTTTCACAAATCGTTTTTAGGGTTTTCGAGTTTTA

CGTATCCACATCGAATCGTCACTATAATCCCAAACCATCTCCGAATCAAATCAACATGATCCTTCACGTAGCGTTTGCAG

CGTTCCTGTTGGTAAATTCGGCGATCGCCGATGCCGGTGAACAGGAACAGGTGGCTGTTACATCTGACGAGGGCGTGCTT

GTGTTAACAAAAGACAACTTCCAAAATATTATCTCGTCGTCTGAATATCTGCTTGTCAAATTTTATGCACCATGGTGTGG

TCATTGTAAACAATTAGCACCAGAATATGCGAGTGCAGCCCAGCATTTAGCTCAAAATGAATTATCAGTTAAATTGGGCA

AAATAGATGCTACTATTGAGAGCGACTTAGCTGAACAGTTTGGTATCCGAGGATATCCAACTTTGAAGTTCTTTAAAAAT

GGAAAACCAATTGACTATACAGGTGGTCGTACCAAGGATGAAATTATTCAATGGGTACTAAAGAAGTCTGGCCCAGCTGC

AAAAGTTTTGCAATCAGAAGAAGAATTCAAATCATTCATTGAGGGAAAACATGTTGCTATTGTTGGCTTTTTCGAAAATA

TAGAATCAGATGCTGCAAAATTATTTTCAGAACTGGCTGACTCTGTTGATGATCATCCATTTGGTCTTGTATCAGATTAT

TCTAAATTTTCTGATTTAGAACACAAGGATACATTTGTTTTATACAAAGATTTTGACGAAAAGAAAGTTGCATTTGATCA

AGAGTTTTCTAATGTTGAAGACATCAAAAGATTTATTTTTGTTCATTCGTTGCCTCCCGTTATAGAATTCAACCAAGAGA

CTGCCCAAAAAATATTTGGTGGTCAGATTAAGAGTCATTTATTACTATTCTTATCTAAAAAAGAAGGACACTTTGACAAA

TTTATTGATGACATCAAGCCTGTTGCTCTTGAATTCAGAGGAAAAATTGTATTTGTTACTATTGATGCTGATGAAGAAGA

ACATCAAAGGATTTTGGAGTTCTTTGGAATGAAAAAGAATGAAGTACCAAGCATGAGAGCTATCAAACTTGAAGACGATA

TGACCAAATTTAAACCAGAATCTCCTGAATTAACTGGAGAAAATGTAAAGAAATTCGTCTCAGATTTTATTGAAGGAAAA

GTTAAGCAACATTTACTTTCTGAAGAATTACCTGAAGATTGGAACAAAACCCCAGTCTGGACTTTGACTGCTACTAACTT

TGATAGTGTAGCTTTGGATTCAACAAAGAATGTTCTTGTGGAATTTTATGCCCCATGGTGTGGACATTGTAAACAGCTTG

CTCCAATTTTCGATAAAGTTGGAGAACATTTTGCCGATAAAGATGATATTATCATTGCAAAAATGGATGCTACAGTTAAT

GAACTAGAGCATACCAAAATCTCCAGTTTCCCCACACTAACATACTACCCAAAGGGAGATAATCCAAAAGCCATTGAATA

TAATGGAGACAGAACATTAGAAGCAATCATTAAGTTTATTGAAGCTGATGGAAAACAAGAAAGTCCTTCTAGCGCATCGA

GTGAAGAAGAAGAAGATGAAGAAGAACGTGAGAAGCCTAAAGATGAATTATAGATACTAATTGTAATTAAATGTTTAAAA

TAATAATTATTTTTTGTCATCTTACTTGTGAAAAACTTCAGCACATTAGAGTTCAACAATTGGACTAATACCTGATTGTG

TATATATAGCTAATTTTGGTTGTATTTGCATTATTTAAACTCTAAAAAATTGGTATTATTACGATTGTAAGAAAAACAAT

TTTAATTTAATTCTCATAATGTACCCTTTTTCAAGACTGTCATAGATGGTTCTGAAATGGACTTCTTAAGAACCATATTA

TTTTAAATCAATGTCTATGATGTTTCCTTGATAAGTAAAATAAAAATAATGAAATTAATATTTATATTTTTAATTAATTC

ATGAATCATTATTTTAAGTTATAATTAAAAACATTTATTTATTAAATCACAATTACTCAAATTCTTGAGTAATTTAATTT

ATAGATCTTTTTACTAATTTTATATACTAATATTAACATTGATTTCTGAAATTAATTGTAATGGTAAAGTTTAGCCATAG

ATATTTATTTATACTTTGATACCAATCATCATATATATATTTATATTTTTTTTTTACATTCGTTATGTATTAAAAACAAG

CGTGGGTGTATTTTTATTTTAACATCACAAAAATTCATATTTTTACTTCATTTGGTTTGTTAAAATGTTATCTTTTTGTA

GTTACTTTAAAATAAATGCTTTATGATTTAATATAACTTTAATTTATTATGGTGGTTTAAAGGACACATTTTTAAAGATG

GTTGTATTACAATATTCAAAAAATGTGATAGTTAATGGAATGTGAGTTTAATTTAGTTATTTTTAATCAGTGTCAGTGTA

TAATTGCTACACATGAGACGATGTTTTACCAAATAATTGTTTTTTTTGATCAGTGTATAATGCTAAGTGCTAAGTTGTAC

TTATAATTTTTGATTCCCAGAAATGTTAATATAAAAATATAATATATCACGAAAACACTTAAAATATAAGATTTAATTAT

ATAAATTATAGTTATAACCTTATAGTTAGGTATTTCAAAGTATATGTTATTATCGATTATATTTTGTTAAGAAATGTTCC

AAATATATTATGTAATCCTACATTTTAATGTTTATATTCTATAAATTATGAATTTAACAGATAATACTAAATAATATATA

TTTTCT

>contig_7803

CCGCGAATGAACGATTTCATATTATTCGAGTAAGCGGTTGGTGAAACACGACATATAATTTAAATTTAAATAACATATTT

TAACACCTATTTATAATATAATAATAGTAAAATAGCATTTTGTATTCTCGCTGCGAGTGTGCAGGTGGTCGTTGTTTCAT

CGGGTCCGCGAATGAACGATTTCATATTATTCGAGTTTTTTTTTTGTTAATTGTTCGAACTGCGGCAAATTTATATTAAT

GTATTAAAGTAGTTATTGGTGGTCCAGAGGAAAACGTTAAGAGTGACGCATTGCCAATTGGTGGATTTTACCGAGCCAAA

ACCGTCTGAAGAGGACTTCTCGTGTACATAACAATACTATAGCTGAAATCTTTTACCGGCTATTTCATTCGGTATTTATC

GCATCATGATACCTGTAAGACTACAATGGGGACGGCTTTTGTGCCGAGGTAATTTGCGACAGACGTGCGTTCTATTGACC

CATAAAGATCTAAGTCAACCGGCTCGACATCGCACCGAGTCTAAAGTAGTTCGTTCGCCGTTTTCGGACGTTCAGATACC

GGAAAATTTAGTAGATGAGTACGTGTGGAGTGGGCTGGAAAGATGGCCGGACAAAGACGCACTGGTTTGCGGAATAACTA

ATAGGAAATTCACGTACCATGAGACGCGGCTGGCTTGCAAAAGGTTTGCGGCTAGTTTGAAGAAGAGAGGTGCAACACCT

GGCCAAGTGTTCGCTGTATTGTTGCCCAACATTCCAGAGTTCGCTGTAGCCTCGCTGGGTGCCGTAGAAGCCGGTCTGGT

CGTGACGACGATCAATCCAATCTACACGCCATTTGAACTAGCTCACCAGTTTAAAGACTCCGGTGCACAAGGTGTGGTGA

CGATTCCTGAGTTGTTGCCCAAAGTTTTTGAAGCCCAACAGCTGATGAAGGGGCCCGGCGTGAAACCACTGTACATAATA

TCTGTCAACGGCAAAGGTTCAAGACCTGATGGCGCGTGGGACTTTAATGAAATGCTTGATCCAATGGTGGACACTTCTAT

TCTGAAAAAGAGCAGATCAAACAGTGACGTGGCATTCATGCCGTACTCGAGTGGTACAACTGGTCTGTCTAAAGGCGTGT

CACTGTCGCACCGGAACCTCTTGGCCAACATCGAGCAGACCAGCCATCCAGATATAAAACACTTTCTTGATACGACAGAA

AATTATCAAGATGTGTTGCCGTCTATTTTACCCTTTTACCATATTTATGGTTTGACAATGCTTCTTCTGAGGGGGCTAAG

CTCTGGATGTAAATTGGTGACATTGCCCAAACTTGAGTCGGAATCATTTTTAAACATACTTAAAAACCATAAGGCGACTT

TATTGTACGTAGTACCACCAATAGTGCTGTTGTTGGGTCAAAATAAAAACGTTACACCTGAACACTTTCAAAGTGTGAGA

ATTATTTGTAATGGTGCTGGTCCTGTGAAAGAAGCAGACGCTGAAAAAGTACTTGCCAGGACCAACAATAAAAATGTACG

TTTTTGTCAAGCGTATGGCATGACAGAATCATCACCAGCCGTGTTTGTATCCAGGAATTCTTCATTGTTCGATTATTTGA

CTGTTGGTCCTCCCGTATCTAACACTCTAGCTAGAGTCGTCGATCCCACAGACAACTCAGTTGAATACGGTCCCGGTGAA

ACTGGAGAAATTCAGGTCAAAGGGCCACAGGTGATGATCGGCTACCACAACAACCCCAAAGCCACGGCTAACACTATAAG

TCCGGAAGGATGGCTGAGTACTGGGGACATTGGTTACTATAACGATAAAGATGAATTCTTCATAGTTGATCGCATTAAGG

AGCTGATCAAAGTCCAAGGCTATCAGGTTCCACCGGCTGAGCTGGAAGGAGTGCTGAGGACTCACCCGGCCGTGTTGGAC

GCAGCTGTCATCGGCGTGCCGGACGATCGAACAGGCGAGGCACCGTTGGCGTACATTGTGCTAGACCAGGACAAACCGGC

CGCCAGCGAGGCAGACATAAAGGCGTTCGTAGCGGAACGAGTGGCACCGTACAAGCAGATCTCAGCTGGTGTGCGGTTCG

TTGAATCTTTGCCAAAAAGCGCAGCTGGCAAAATATTGAGGAGGTTACTTAAGGACGAGTACGAAAAGACCAAAGGCACC

AAGTGATCACCGGAAGTCACGAGAATGTCACGATTTCAGCGATTAATAACAATATACATAATATATTATTGTCATAAATA

TTTGAGTGCAAAAATTAGGTTAAAAAATATGAAAAAATACTCGTCGTTTTACTTAACTATCACGTCGTGTATATTAATGG

TAACGAATTGCTCATACGAATTTATTATTACTGTATTATTGATTTCTGATTTATAAGACAACAAGTGATATTTATTATTG

TTTTTATTTATTTTATCGATTGTTAAAACATAATTATTTATCGTCACATCATATTATTATGATAATTTCCATATAAATAT

TATATTATAT

>contig_7807

GAAATCCGGTGCGGTCAAAGTGACAATTACACCATGTCAGCCCAAGACCCTGAGTCATCTTCCCAAGAGGCCGCCCGTGG

ACATCGCCTTTTCCGACCTCATGTACACCGTGACCGAAGGCACCAAAAACACTTCCAAGAATATTTTAAAATCTGTAAGC

GGCCGTTTGAGATCAGGTGAATTGACGGCCATCATGGGACCGTCTGGTGCCGGGAAATCCACACTGCTGAACATACTCAC

GGGATATAAATGTTCCGGTGTTAAAGGTTCTATCACGATTAACGGACATGAAAGAAATTTGAGTCAATTCAGAAAACTGT

CATGTTATATTATGCAGGACAATCAGCTTCATGCCAATTTATCTGTAGAAGAAGCTATGCACGTGGCTACTTCGTTAAAG

TTAGGATCAGACATATCGAAGGACAGCAAATATCAAGTG

>contig_7941

CGCGGACGTCGTTTTCTACTTTCGTTCACCGACTGATCTAAATAAATCTGATAGCTAAATTCTGGGCACAGGACGTATCA

TACCACGGCAGTTGTTACTTTGTTAGTTTGTTTAGTTTGTTACTTGTTGTTCCTTTCATTAGTGCACCACGAAAACAACT

GTATTTTAATAATAATAATTTAACCGTACTATAATATCGCGTTGATTGCGTAGTACGTGTTTTTGTTTATTTATTTTATT

GTGCGTGTCAAGATATTATTTTTCAATAACGATTAACGACGAGTTGGTCGTTTATATTTTAATTGTTCAGATGAGTTGAA

ACGCTCAACCAACGCTCACAATGTTTATACTAAAGATAATCGTCATCACGTCAGTGATACTTCACATTGGTTCACCAGCG

GCGCAATGCGGCTCCGACCCTGTATTTGAGTTTCAGTGTCTAACTCCTGAGTGTGACAGTATACTGGTAAATGATAAAAT

CTCTGTGGGCCGACGTCTTATTAGTCCAAAACAAAATGTCACTATTATTGGTCAAAATGATGACATGTTTTTGATAAAAA

GCATGAAGGGTAAGATGTCTAAAATCTATAGAAAAGATATTAAAATATTACATTTGGCCAAATTTGAACATTGGAAGTTT

AAAGTAAATTTTTTTAAAAATAATTTGGAAATATGCGAATCAAATAATCAAAGTAACGAAGAAAACAACAGTGATTCCAG

TATCTCTCAAATTAGTACAAATACAGATGAAATAAATGTTAAGATAAATGATGGAATTGATACATCAGAAGTTATCCAAA

ATGTAAATAAATTAGATACTGAACAATACGATGACAACTTAAGTAATGAAACAGATTCAATTTCTTCAGAATTTGATGAT

GAGATGGATGAAAGTATAGAAATCGAGAGTGAAGATGAATCAGGTCTAGATGATATTTTAGTTCAAATACCATCAATTGA

TAGTACTGATAGCAGTATAACTAAAAGTGCAGTTAATAATATTTTATCTACAACGAGTAATCAGATTTTAAATTCTGGAC

CTGTTAATTCTGAAAAAAATTATAAATCTAATATTATTGACCAACAAAATATCCATACATTAGCTAATGATCAACAATTG

GAAAATGATTCTTCAATAGATTGTTCTTCAGGTTCTTGTGTAAAAGAAGTAAATGATAACATTGTTACTGACAATTCAAG

AAGTAATATTTCTCCGGAAAGTACAGATGATTTTTCTCAAGAAATAACACAGCAATCACAAAATTTAAATAAAATAGATA

ACTCAATAAAAGAATCAGCTAATAATATGCTGCCAACAAATATTAATTCAGAAGTAAACTTACAAGATGACAATAATGAT

AACAGTACTGAAGAATTTATTCTAAACACTTCATATCAACAAAAAATTGAAAATGAAAAAATTATTCAAAGATCATCACA

TTTAGATAATACAGATAATTCGATTAAAAAATCAGTTAATATAGTATCACAAATCTCTGATATAGATATTAACTTACAAC

ATAATAATAATGGCAATGAAGTGCTTAATATGAACAGGTCTTATCAAGAAAGTAAACCTATACAGACTACTCAACAATTA

CCAAGTGAAGAGGAAAAACTAGATGTCTCAATAAAAGATTCAGTTAATAGTGATATACCCATACAAGACTCCAATACAGT

AGTTAGTTTAAATGAAATTATTAATGATTCTAAAGAGTTGAATATGAACAGTTCTTATCAACCAAAAAGTGTAGAAGCAA

TAACAAATGTTTTAATAAATGAATCAGTTAGTAATATAGAGTCACCAAACTCTAATACAGTTGAAGTTAAAAATGATGTT

ACAGAACAGCTTAATAAATCCCAGCAAGAATTTAAAGATAGTGATTCTTTACCAGCTATCACAGAAGTGGACAATCAACA

GGTTAACACTAATAATTACAAACAATCATCTTTATTAGAAAAAAAAATAGTAGAAATAAATAATGAAAATGGAATTTCAA

ACGATAATACTATGTTTATTGGGGAGAATTTAAAAAATGAAGTTATAAAAGAGCCAGTCATTAGTGAAAAAACTATTATT

GATATTCCTGAATTATCAAACGATCACATAGAATCTAAATCTCATAACATTCACAAGAATAATTTACCTAATGTTGAATC

TGTTATATCCATTAATGATTCAACTGATGGTAATATGGCAAATTTGTCCTCATCGCAGGAATTAATTAATGCAACAGAAA

TTGTTCCTTCTAATGATTACCATACAACAGTTGTTAATGAACAAGATGTAACAGAAACATTAAACAAAGAATCAATTGAT

CAGTGTACATCAGCTGGGTGTTTGCAAACAATAGACAGTACTGAAAAGTATGGTTATGAAGGTATTCAACATCAGTATAA

ATCTGAAAACAATGAATTACCTTCCGAAAGTATTAAAAAAACTATGGAATTTGATAATAATGAACAGTTGAAAGAATTTG

TACCTAAAAGTTTTGTGGCATCTTTTGGTCACCCAGAGGCTTGCAGTGGAGTTAGCTGTCTTAATTTTAAAAGAAATTTA

GATAAAACAGTGAAGTCAACACAGCCTTTAAATCCAGTTCCCAGAACTTTAAACCCTGATGAAATTTCGCATTCAACTAA

TATTGACAATGGAAAAGAAAAAGAAAATATAATTAATGAAGTAACTGAGCAATCTCAAGTGGAATTGAGTTTCTTAGATC

AACTTCAAAATTGGTTATCGTCTCCTTCTCTAATTACAATTGATTTTCTTTGGAACATTTTTGATGATAATACTAGTGAA

TACAATGATGTATATCCTGATGCTGTTGAAAATCAAAAAATTGTAGAACATAAGAATATCAAGCATTCAGAATTGTACTT

CATCGTTGTGGCTTTAGTCACATTACTATTTACACTAATTTATTATAAATATCAAAATGGATCATATGAAAATCATTTGT

TAACATTACTAGCAGCTAAAGATCAAAGTTTGCTAATCGTGGAAAAAGAACTATTTTTATTAAAAGAACAAAACTCTGGA

AAAACACAATCTACAACAGAAAGGAATTCTGAAATTGAATCTTTGAGTGATCATTTGACTAAAGCAGAGGATATTATATC

ATCTCAAGCCATACGAATTGAAAATCTTGAACAAGAAGTTGTAGAATTAACAGAAAATGGTATGGAAATGCATACACTAT

TATCTTCTGCATTAGAATCTAGTACAAAAAATAAAGAACAATTAAATACTTTGAAAGTAAAATTGGTTGATTCAGAAAAT

TTGATAACAGCTCTTACACAAAAAAATTTTGAAAAATCTACAGAATTAGAAAAGAAAATTGATGCTGGGAAAGCTCTTTT

AATAACTGTAGAAGAGTTAACTAATAAAAGTAATAGCTTGGCTGAAGACAATGCTAAACTATCTTTACAATTAAAAGAAG

TTATTAATAGAAATAAGGCAAAAGAATCAAAACTACTTGCTGAAATAGATAGTTTGCAATTTGAACTGGACAAGCATACC

TCTGCTTTAGTGACAGCAGAAAATGAAGTTTCTTTATCAAAGGAAGCTTTGCAAGAAGTTATGTCACAGAAATTTAATCC

TGCAGATAGCAAGCAATTTTTATCTTCAATTAAATTATCTGCTGAACTCAAATCAGCAAAACAAATATCTGAAGGATACA

AACATAAATTAAATGACGCTTTAAAATCTAATCAACAATTCCTGGATAACATTGAAGTTTTGAAAAGTAATATTGTGGAC

TTAGAAAATAGCTGTTCAAAATTAGAAACAATGAATGAAGAGTCTGTGAACAAATTGAATATTCTAACAAAGTTTTTTAA

AGAACAAGAACAAGAGTACCTTAAACAAATCAATGAGAAAGCAATGTTGTGTGACAATAAAGAAGGTGAATCTAGCGATC

TTAATGAACGTATTAAATCTCTAAACCAAGAAATTGTCAATTACAAATCAGAAATTCAATCATTGAAAAAGGAAATAACT

GATCAAGAAACTAGTTTCAAAATTCAATTATCTGCAGCAGATAAAAAAGCCAGTGATTATTGGATATCATTCAGACAAGC

TGAGCGGAAACTCAAGGAAATGGAACTCGAAACTGCTCAACTAAGAAATAAATTGACCATGGTTGATAAAAAACTTGAAA

ATGGTGCAAAAAGCATAGATTCGTCTAAAGACTTTGAAGATGAACTATTGGACATACCATTGCCAACTGTGTCTCCCGAT

TTCACATTATTATCATTACTTGATTTTGAGCCACCTCCTCCAATTTTAACTAATCAACGGTTACCTCCACTTGGTGCTTT

GGGTCGAGCTCCAAGTCCACCAGCTCCTTTACCAAGGAGGCACAATCGATCACTATCACCTGGTTCTCCGCCACCTTTAC

AAAGGTCTTCCGCATTTCGGCCATTGACTCAAAGATATAACTATATGAATCGTGAAAATTCATTGGGTCATTCGGTTGAA

TCCCTTGACAAATAATTATATTATTTTGCACTGTTACTATTATGATATGCAAATCTCTGAGATTATTTTCAGAAATTTAG

TTTTTGTTAAGATTAATTATTTTTGGTGTATATCATTTGATTTAAGTATTTTAATTTTTTACAAATCCTTTTTTGCTTAT

TTTTTTGGTTTTTTGCTTACATATTTCTAACAATCGTTATATTTATTTTTGTTTTCACTATTAAACCTGTAAGTAATATC

ACTGTGATTTAATGCATTTTTAAACTGTCCATATAACTACAATTAAATGTGTATAAAAAAAATTTTATTTGAACCTATTG

TTAGAAAATAAGTCGTTTAAAGATGGATCCCTATTGAAGAATTTCTGTACATTAAACTAACTGATGGTAATTCAACCACC

GTATATATATATAATATGGTAGAATTGTATCAATATATTACAATTCTCTGTAATGTTGTAAACAGAAATGTGCGCGTGTA

AACACTTATGAGTGCTTCATTCATTTTCAACTTTGACTGATGATTAAAGAAATATATTTTTGTAGTATAAGGATTAATAA

TAATGTTTTTAAATGTATTAAATACTTTATTGCTACTGTAATATATTATGTAAAATATTAATATGAATTTGGAATATCAA

TACTGTTTAAAATATTTTATAAATTATAAGTTATGTTAATAGAATTATAGAATTAAACTCTATTTGCCATTCCAACTCAA

TGTAAAAAAAAAAACTTATTTAGTTATAATATTTGTTTAACATATTTTATAGTTATTTTAAGATTTATGTGTTAATTTAA

ACCCATTGTGCTATCCATGCTAACACTTGGTGAAGTGGTGAATTTTCTGGTACACAATATTTTATTTTCATTATTATTAT

TATTATTATTACTAAATATTATTGTATAATTTTTTTTTTTTTTTTAGACCAATTTATGAGTATTTGTTTACCTACTTTTT

TTGGCATATGTATTAACTCAAAATTATGCATTGCCTATAAGTTAGTCAATTTTCAATTATGGAAAAAATTATTCTAGGGT

ATATGGGATATTGTTTAAAAAAAAGTTTAAAACAATAACTTGACATTAATTTCAGCCTAATATTTTGACTAATTAAAAAT

AATTAAATATTATTCAACTTGTTATTTATTTTAATCACAAATAGAGTTATGAAAAAATTTTAAACAAAAAAAAGTTTGTT

T

>contig_7990

TGTGTGTGTGTGTGTGTGTGTGTGTGTGTGTGTGTGTGTGTGTGTGTGTGTGTGTGTGTGTGTGTGTGTAGATGGTGCCG

CGATGTTGTGTTTATAAAATTTCGATGACATCTATTTATGTCACACCGTCATATAAATTATGAATTATATAAATATATAT

AGCTAAGTATATACCGTTTGTAAAATGTACCTATTAAAAAATTGTATGTACGTGAACGATATTATATCATTTTATTATTA

CGCATAACTACGTTGACGTTTCAACTGCTTGCCGTTCTGATTGATTCCGCTCATGGACTTCATCATGGACACTATGTTGT

TGAAATACTTATTCAGTTTCTCGTTGATGTTCTTCTCGTTCACGATTTTCCTGATCTCGTCCCAGAACGTCTTGACGAAC

AACGGGGCGTTGGGTTTGTTCGCGGCCGTGATGGCCAGCCCGGCAGTCTTGTTGACGACCTCGGGCATCACGTCCGACTT

CAACAGCGAGTACGTCAACGCGATGATGGTGGCTAGCCCGGGGCGCATCTTCTGCATGCCGGACCTGGCCAGCAGCACGT

GCGCGGTCGTGGCCAGCATGTTGACCATGTCCGCGCCTTCGGCGGACGTGAAGATCTTGTTGATGTTCTGGCCGACCGTG

CCGATCACCCGGGACGCTTCCGGACCCTCGAGCATCAGCTGCCACTCGTGTTTGATGTTGGCGAGTCCTGTCTTGGCCAC

GTCCGTGTTCATCGCGGTCGTGTACATGTCGGTAAAGGCCTCCTTCACCTTACCGTCCGGGATGACCGCCTGAGCACTAC

TCGGCAGGTTTTGCTCCATCTCAGCCAGCCGCTGGCTGGGCTTGTACTGAAATTCGTCCGTTTGGGGTTCTTCCTCATCC

TGGTAGTCGGCTTGGTTTTGAGCTTCCTGTTCTTCCTCTTCCGACAATGCCCCGTTCATACTGTTTTCACCGTCGTCGTA

CTCTTCATCGTATTCTTCGTAGTCTCCTTCTGCCTTAATGGCAATTATAGCAGAAAAAAATAAACATATAAAAAGTGATG

TGTTCAATTTCATCTTGCTTGTCCGAGGTAGATTTGTAATTTATATTTTTACTCACTAATACAAAAACTCTTGATACAAT

ATTTTCATTTCATCAACAACACATACTTTTATATGGTACCCCATATATGAAGATAATTGTATAGAAAT

>contig_8043

CGACGCCGTCACGCCGACAACAAATGCCAGATAGTAGTATCGTTACTACTAATTTAATAACTTATGTACTAAAGTAAATA

TTTAATAATTTGTATTATGTTATGATTTATGTTATTTTGTAGTCACTATTAAATATTACTATGAATGTAATGGAAAACTT

TTATTTATTTATCACTGTTTTTTGTGGTATATTCACACTAATCAATTGTACAAGTATAGTGAAACCTAGGGGTATCCCAT

TTGAAAGAGCTTCGCTTTATGTACCCGACAAAGATTTTTCATGTTTCGATGGAAGTTACATAATTCCTTTTTCATTTGTA

AATGATGATTATTGTGACTGTCCAGATGCTAGTGATGAACCTGGTACCTCAGCTTGTCCTAATGGCACATTCCATTGTAC

CAATGCTGGTCACACATCACTTGTCATACCATCATCTCGAGTAAATGATGGAATTTGTGATTGTTGTGATGGATCCGATG

AATGGGCAAATATGTTAAGGAAAGGTACATGTGAGAATACTTGTGAAGAACTTGGCCGAGCAGCAAGAGAAGAAGCAGAA

AGAGTTCAAAAAATATTTGTAGCCGGCCATGAAATCCGTGCTCAATTAATTGCTAAAGGAAAAGAGCTCAGACTGGAAAA

ACAAAATAGAATTACTGAACTTTTTGAAATACAAAGATCTGCTGAACTTACTAAAAATAATACATTGCATGCCAAAGAAA

CAGCAGAGGAAATTGAAAAGTCTGCATTAGAAAAATATAAAATTATGAATGATGAAAAAAAAAGACAAGAAAATGAAAAA

GAAAAACTGAAAGACCAGAATGAAGCTTTCGAAATATTTAATCAAATAGATACAAATAAAAATAACAAAATTGAAGAAGA

AGAAGTAGAAGCATATGCTACTTTTGATCAGAATAATGATGGTACTGTTTCACAAGATGAAAAAGATTATTTTATGGAAA

ATAAAAAAGAAATTAGTTTAGAAGATTTTGTTTTAAATGGCTGGATTCGTTTGAAGCAATTAATGCTAGTGGAAAACATG

GATGCAAAAGATACTGAACTCCCAGTTGATGTTGATAATGATAGACAAGTTGAAGAAACAGAGGATCAAGATGAAAATGA

AGATGACAGTGACCATGTAGAAGATGACGTAAAATATACAGAATCTGAAGAATTTGATGAATCACAGTATGATGAAGAAA

CAAAATTAATAATTGAAGAAGCCAAACAAGCACGTAATGCATTTGAAGAAGCTGATAGGAAATTTAGAGACCTACAACGT

GAAGTAACTCATTTACAAGAATCACTGAATAAAGATTTTGGACCAGAAGATGAATTTGCAGCTCTAGATGGAGAATGTTA

TGAACTTTCTGACCGTGAATATGTTTATAAATTATGTCTTTTTGATCAAATAACTCAACGGTCCAAAAATGGTGGATCTG

AAGTAAGATTAGGTACTTGGAACAGTTGGATTGGTGAACCAAAATACCGTACTATGTTATATGATAGAGGACAACACTGT

TGGAATGGTCCTCAAAGGTCTACTCACGTACGTTTAAGTTGTGGCCTGGAACCAGCATTGCTCTCAGCTACAGAACCAAA

TCGTTGTGAATACGCTATGGACTTTGTTGTACCAGCTGTTTGTGTACAGCACAGCGAGAGACCTTCAGCACCTGTAATAG

ATGCTGAACATGATGAATTATAATTTTAATTGACTAATTTTAAATCATTGAATTTGTGTCAACTGTTTTTGGGGTATTTA

ATACAAAAATATATTTGTTCATTATTATTTAAAACCAATGTACTATTATTATATTTTATTTATATACTTATTTTTTTTTT

TAAATATAAAATTGTAAAAGTAATATTAAACATGTTTCTATTGAAATAAAATACAATTGTGATGGATAGTTTAAACCCAA

GTAGTCATAAAATGAACATCAAACTTAAAAATTGTAAAGTTCCATGTGGACTATATG

>contig_8089

TGTTCCAGAGCTACACCTCTACGCCGTCGCTGACGCCCGACGACGATGTGTTCAAACATTTGGCTCTGACCTACTCCAGA

AACCACCCGACGATGAACCAAGGAGTGGCATGCAAAGCGGGAACGCCGACGTTCAATAACGGCATCACCAACGGAGCCGC

GTGGTATCCGCTCACAGGAGGTATGCAAGATTTCAATTACGTTTGGTACGGTTGCATGGAAGTGACGTTGGAATTATCCT

GTTGTAAATATCCGCCAACTTCCGAGCTGCCCAAACTCTGGGAAGAAAATCGATTGTCACTCGTCAAATTCTTAGCCGAA

GCTCACCGGGGTGTCCACGGATTCGTGATGGACGAACACGGTAACCCGATCGAAAAAGCGTCTCTTAAGATCAAGGGTCG

TGACGTAGGATTCCAAACGACCAAGTACGGTGAATTCTGGAGAATATTGTTACCCGGCGTTTACAAGCTTGAGATCTACG

GAGACGGATACATACCGAAGGAAATGGACTTCATGGTCGTGGAACAACACCCCACGCTGCTGAACGTAACGCTGCACACG

TCCAAGGTAGGTCGGCCACCGAACAGGCCTCGGCTAAAATCCTGGCAACCGCCGTGGATGACCGGCGCCCAGGACGAGAC

GATATCCGTCCGGGGCGCGTCTCCGAACTTCAGCAGGAACTGGCGGGACGGGCTGCGGGCTTACTGCAACGACGCGGGTC

CGACGTCGCGCAGCTGGCACGCGAGAACCGTGACCGTGGTGGCGGTCGTGCTTTTCACCGGGCATCGCAAATAACTGCTT

TTTCGCGATTTATTTTAATATATAATATTATATTGTACAACGTAATGACGAGATGTCGTAGTGGTCTAGCCAACTGAGTG

GTCAGCAGTTACGATTTTGGATTGTTGGGTACCTTGTTAGCTTTATAACGTTAACCGCAGTGAATATAATTGACCTTTTA

TGAAATTGAACATTTCTTTGGTTTTTTTTCGTTACGTTTTAACGATACCTAAAATATTTAAATTTAAAAATGTGTATAAC

TTGCTTAGAAATTAAAACAAATATCGTATAGCCATATAGGCGATATAATATAGTAATAATAAAACAAAAGAGAATCAGAC

GGTCGATGTCCTTTAAGTTTCATAAAAGGTCAATTCACTCTTTTATTTTACAAACATAAACGCATGGCACAAAATTGGAA

CTGCTGAACGTATATTTTGGGATGTTATCTTTACGGTTAGTAAGACGTCCGTGTACGACGTTTTACTATCAATGCTGTCC

AAGCCGAGCAGATAATTTTGAGTAGGTATTGGCGATTTATTATACGAGGAATGTCTATGCCTGTGTATAGGTACCTGTAT

AGTATATATTAATGAACGTTAGTGTGACAGAACTTAAGTCACAAAAAAAAAAAATCAAAATATTTTAAATGCTTCTTAAA

ATTACACCTTAAACGGCTTTGACTTAATAATTTACTGGAGGAAACGTGTAAAAGAGTTTAAACATTTTTTTTCTAGTACT

ATCAGTGGTCCGATTTAAATATTTTTCAAAAATTTGTATTGTTGAACATTTTTACTGCAGGGTTATTAAGGAAAAATATT

AAATCTTGTTATGATTTATAGAACGGGGAAAAAATATATTACTATTTATTTTGCTTATCGAACACAACGTAAAAGTAAAC

AAATATTATAAGGATAACATTAATTAATTTTGTATCCATTTTCCGTTTCATAAGACAACAGTATCTATATCAAATTCTAA

GAAACTGAAAAAAAATGTAATATGTTTAATAAAAATAATAAATTAACCAAACATTTTTACTACTTTAATAATTATTAGAG

AGAAATGAATAATTATGGTAGGTATCTTCTTTAAATACATTTTGTTTTAATGGTTGATACTTGTGGATTTATGGAGTAGA

ATAAATTAGAAGAATGACTTTATAACATCTTATTTTTGAGTAGGTACTTTACTAATATTTATTATGTTGTCTTTGTCATA

ATCATCAGTTATTAATGTGTTTAATCTATGCATATCCAAAAATAATATCAGTATTGTGTTATTTAAATTGTTTAATATTT

TTATCAATTTATAAGGTAAACTATAAAAGCAGTTAAAGAATTAAAGTTAGATTAAAGGTGATAAAAATAAATACATTTAT

GACTAAGAAAATATAAGGTATTTAAATTTGTAAATTACATTTTTGAACATGTATTAAGCTAGAAAAACAAAGAATTTTGC

ATTTAAAGTGTCTGAAATACCTATTATAACAATTGAAAATGATATAAATACAATCCAAATATTTTTTCTTGAGTGTATTT

GTATAATTATCATAGTAAAATTATTAATATATTTGTG

>contig_8225

TTTTTGTTTTTCAATTTTTTTTTTTTTATTTCCAATAAATTACTATAAGGTATACCAAATACCTGTTGTACATGCAAAAA

GGTGTATAACGATAAATTACAAGTATTTCAACTATTAAAAATTGTTGCTTCTTTTTTTTAAAAATAATATCAACATATAA

CAACAATTCCTTACAATAATTAAAATAGGTGTAAAATTATTTTATATAATGTATAAATGAAATAATACACGTTTTGTATC

CACAAACATACATTATTTCTCATTGCATTGTTTTTGTGTGTATAAAAAATTAATAATTTTAGGTACGCATTGTCTTTTAC

TAAAGAAAATCATCTAAAAATAATATATTTTAGATTATAAAAATTATCAAGTTGTAATAAATTAATACTGAGTTTCTTCT

TTGAATTTATCAGCGTTATTCAGCTGTTTGAAGCATGGTAAATTTAATTATGTACCTAAATTTCCGGTGCACTGGCGTAT

AATGAGATTATCCCTGACTTCTCAATGTCCAAAAACATTAATTGTTGTCTGATCCTATTGAAATCACACAAGTATTTATA

GCTGCTATTACATCCATTTGGCCCGTTTGAAACCGTATCGCCCCATCGATTTAAGAATTCAAACACAACACCATTTGTCC

AACCAAAACCTGTCTGCGGAGTATATTCACCACCGCCACCGGTTTCTCCTGAAGCTAGAACGTCGTATTTTTCGAACATC

ATTGATTTCTCTGCGAATCCTTTATAATTTTGAGCGTAACCAAACTTCGGCTAATTTTGTGCCACCTGTTGTGCAAGTTT

TTGTTGTGTTTTGTCTAGACCTTGAATGATGAAGCCTGTAGAGGAGGCCATGCATTTGGAAAGTCCCATTGCTGTGAGGA

TG

>contig_8346

GGACTCGCAAGTAAATGGTCACCGTCGCAGCCACTTCTATAAATAGTACTTGCAACTGTGCGGAGACGTTGACTATCGCT

TGGACATTCCCTGGTCCTGTTTCGTCTCTATACCTAGCGTTCTTGCCTGTGCAAGTAGTTTCATCTTCCAAATCATCTAA

TTATTAAGATGACAGGAGCTGTGTCTTTTGGATTTTTTGCCTTCGCGCTGACAGTGACAAGCTGTTTGGCGGCCATCAGT

GAGGTTAAAACCAACAACCCCGTTACGCAGAAGCCTGGTCGTTTCCTCAGCTTGCCTGTCGCTCAGAAATGTAGTCAAAG

ACCGAAAGAATTCACGTACAAAGGACGCAATTACTTCTACAGTGGACACACACAACAATACAAAACATCCAAAGTAGATT

GGTTAGAAGCAAGAAATATCTGCCGAGAATACTGCATGGACCTCGTATCGATTGAAACTCAAGAAGAAAACAATCTCATT

TTCCGACTGATCCAACAAAATGATGCTCCGTACATCTGGACCAGTGGTCGGCTTTGCGACTTCAAAGGCTGCGAGAACCG

TTCGGACCTGGAGCCGAAGAACGTGAACGGATGGTTCTGGTCGGCCACCCGAGGCAAGATACCGGCCACCAATCAGACAG

CGGAAGGCTGGACTTACAGGCCGTGGAGCAAGAGCGGTCACAAGAAGGTACCGCAACCGGACAACGCAGAGTTCGACATC

AACGGCACGGTGGAGTCGTGCCTGAGCGTGCTGAACAACGTGTACGGCGACGGTATCGCGTGGCACGACATCGGTTGCTA

TCACGAGAAGCCGTTCGTGTGCGAGGACAGCGACGAGCTTCTCAACTACGTGGCCGCCACCAACCCGGGACTCCAACTCT

GAGATCGCCACCCAAACACCGCCAACAAAAATCACCGCCTCCCTATCACCCAGTCATTCCAATTCCCATCTTCCACCCAC

ACACCTACACGCACGACATGACAGACGTCCCGCGCGATCTATAATATTATAACATTTTCCTATACACCAAACGGCGGCGG

TCGTGTGCCCCCGATAACGGTAATTATATATTGTCGACCCTGCGAATTCAAATCGCGGATATCCGTGGTTAGAGGGCACA

GGCGACCGTATATTATTATAGTGTTGTAGTTTATTATCGGTGGTCATTCGGTCTTCGACTAATTATTATTGCAGACTATC

CATCAGCGATGTTAGAAAAGTAGTCAAGATTTCGTATCATCAGAGAGTCTGAGGTCCGTCGTGTACGATCGTTCAACGAA

ACGAAATGTTACTCTAGTTTTCTTATTTCTTTCCGCCGATATTTTAAGAAATTAAACTATTTTAGCACACATATTATAAA

TCTTACTGCGTTTATCCGGAGTTCTCTATATTTAAACAATGCGGTTATAGGACGCAGACGAATCCGCAGACGTCTGTCCG

TCATAATAGCGTATATAATATTATAGTAGTCGTGTGTCGAGTCGTTCAAGTCTCCGTCGACCTGTAGGGCGCATTAACAA

TGTAATCGAACTCGTTCCGTTCTGTCCCTAAAACATACAATCGTATACGACGCGCTTCTTTTCGCGTTTCACTCCTCTCC

CCCCTCCTCCGGCCATCTATCCATAATCGTTGTTCATCGTCTCATAATTTTCCATATTTTTATAATACCATATTTATATT

ATCATACGCGCATATTATTATATGTCTTAAGTTTAATTATTATTATTATTTTTTTATTTTACTTTTTATAACTGATGTGT

TTCGGTTCACTAGAATTTATGCGCGACCACAATCGCATTTAAGTATCATTCGTTTTTATAATATTTAACTACTGTTTTTC

ATATGAATAAACGTTCGGATGATACACATTTTTGTA

>contig_8514

CGTGAAGCTATATCAGTGCGAACGAGACGTATTAACTTGTGCAGTAGTGCTGTTCTGTATTATTCACCATTTATCCAGTG

TAATTTTAAACACAAAGTGACTTTTAAAAAATAAATCGTCATCGAATTGTGCGTTATTACGTATACGTTTGTTTTTATAT

ACACAATTTAATATCTATTTGTATTAATTGTACGTGAATATAATCGAGAAAATTACTGGCGAAGAAATGGTTGGAAAGAC

GGCAATCGGTATCGATTTGGGCACCACCTATTCTTGTGTGGGTGTCTGGCAACACGGAAAAGTAGAGGTCATCGCTAACG

ATCAAGGTAACAGGACCACCCCGAGTTATGTGGCATTCACTGACACCGAACGGTTGATCGGCGATGGGGCCAAAAACCAG

GTGGCAATGAACCCCGTGAATACGGTGTTCGACGCCAAACGTTTGATCGGACGTCGTTTCGACGACGAGAAGACGCAAGC

GGACATTAAACATTGGCCGTTCAAAGTGGTGAACGACTGTGGGAAGCCCAAAATCCAAGTTGAATTCAAAGGTGAACAGA

AAGTGTTTGCGCCGGAAGAAATTAGTTCGATGGTGCTGATGAAAATGAAAGAGACAGCGGAAGCGTACTTGGGACGTGAC

GTGACCGACGCCGTCATCACGGTGCCGGCGTACTTCAACGATTCGCAGAGACAGGCGACCAAAGACGCGGGCATCATAGC

CGGCCTAAACGTGATGAGAATAATCAACGAACCGACAGCTGCAGCTTTGGCGTACGGTCTAGACAAGAACCTGAAAGGAG

AGAGGAATGTGTTGATATTCGATTTGGGCGGTGGCACGTTCGATGTTTCTGTTCTGCAGATCGACGAGGGTTCGATATTC

GAAGTGAAGTCAACGGCGGGCGACACGCACTTGGGCGGCGAAGACTTCGACAACCGGCTGGTGAGTCACTTGGCCGAAGA

GTTCAAGAGGAAATTCAAAAAGGACGTACGTAGCAACCCGAGAGCGTTGAGACGATTGAGAACGGCTGCCGAAAGAGCTA

AGAGAACTCTGTCGTCTAGCTCGGAGGCCACCATTGAGATTGATGCTTTGATGGAAGGTATCGATTTCTACACACGAGTT

TCTAGAGCGCGTTTCGAGGAGTTGTGCGCAGATCTGTTTAGATCTACTTTGCAGCCAGTGGAAAAGGCGTTGGCAGATGC

CAAGTTGGACAAGGGAGATATACACGACGTGGTGCTCGTGGGTGGCTCGACGAGGATTCCAAAGATTCAGAGTCTGCTAC

AAAACTTTTTTTGCGGCAAACCACTTAACCTGTCCATCAACCCCGACGAAGCGGTAGCCTACGGTGCCGCGGTACAGGCG

GCCATTCTCAGCGGTGACACGAGTTCTGCGATTCAAGACGTGTTGCTCGTGGACGTCACTCCTCTGTCGCTGGGCATCGA

GACCGCAGGCGGCGTGATGACCAAAATCGTCGAGCGCAATTCCACTATTCCGTGTAAACAAACCCAAACGTTCACTACAT

ACGCAGACAACCAACCAGCCGTCACCATCCAGGTGTTCGAAGGAGAAAGGGCCATGACCAAGGACAACAATCTGCTAGGA

ACGTTTGACCTGACCGGCATACCTCCGGCCCCTAGGGGCGTACCCAAGGTCGAGGTGACTTTCGATATGGACGCAAACGG

TATTTTGAATGTATCTGCCAAAGAAAATAGTTCTGGGCGGTCCAAGAACATTGTCATCAAGAACGACAAAGGTCGCCTGT

CTCAAGCCGAAATCGATCGTATGCTCAGCGAAGCGGAACGGTACAAAGACGAGGACGAACGACAAAAGGCCAAGATCGCG

GCCAAAAATCAGTTGGAAAGCTATGTGTTTGGCGTTAAACAAGCGTTGGACGAGGCCGGCGATAAGTTGACCGAATCTGA

GAAAAATACCGGCAAACAGGAATGCGATGCAGTGGTTCAGTGGTTAGACAATAATCAATTGGCAGACAAAGAAGAATACG

AGTACAAACTTAAAGAAATCCAAAAGAGCTGTTCTGCTTTAATGATGAAGATACACGGTGCAGGACAACCCGGTGGCGCG

CCACCTGGTGCTCATGGTTTCCCAGGATCTAATGGACCAACCGTCGAAGAAGTAGATTAATTATTTTAACAGTTCCATCC

CTAGTGAATACTATTGTTAACTATATCCTAGATATACATTTTATTACTATTATTTTTTTTATTATTATTATTATTATTTT

TATTTTGTAAATAAAATAATCATAAATTATATCTTAGTTACAAATGAAACACACATATTATATTATAGGTACAAAATGTT

AGGTATAATATTAAAAATATA

>contig_8994

GATCGGTGTTCCAAAATTGATCGACGATTTTTTATTTTTCCAATGATAAAACAGTTTTCCAATCAATTCCGTAAACGGTT

CTTCAACTACCAAATACAAGATGATGGAAATCAAATAACACATGAATACATCGCTGAGCCAAGAGTCAAACAAATATTTT

GATGTCAGATGAATGGGTAACCTTTGTGAATTCTGAGATAATATAAAAACAGTCAAGTTCACCAACGAAACGATATACGA

TAGTTTACCCATAATCGTAAAAATTCTATTGTTAAATACATTATTAAGTATACCATATCCAGACGTGAAATAACTCATGG

TTATCCAAATACCCGCTACCGGCCACGTACTATGAGAGATAACCGAGTACAAGGCGTGTTCCAATGGATAATAAGGTCTG

TTTCTTTCATAGAATACGGCGCCGTAAAACTGAGCCCAAAAAGTCAATGTGCAAATAGCAATTAATCCGCTGTGTACTAC

TATTTGTGAAAATTTAACTTTTTTTTCTTTCAGTTTTTCGTTTACAACGCTCATAGCCAAACCTACAAAGAATGGCGTTG

CCCGCATGTAACTTGGTCGGTACGACTTGTTTAACCATAAAACATTTCTTAAGTAAATTCGAGAATCTGTTGAAATTATA

TCTATTCCTTTTCGTCCTGTCCAAAGCGTGACTATGAACGGCACGAATACTGACAAACCAACTACAGTGCCAAGCAATGC

GATTCCTTTTTTGGGTTTTTTTATTAAAAAATAAACGATAATTACTCCGATTATAAAAAACTGCATATCACTTGACAAGT

GCCAACTCATAATTAAACACTGATGTTTGATATCAACAAAATTGCTTATGAACAACAGGTTCGTCCACCAATAATTTTTA

CATATTTCGGCTTCCTCCCAGGAATTACTTGGCCAAAGAGGGCCATGTCCAAGGTGGGGTATTATATATGCCGTGATCGC

CATCACCGCGCAATAAGTCGGTAAAATTCTGAGAATTCGCTGGAAGATTGGTAATAGGAGAGTTTTCCAAACCGATTCTT

CCTTTGGTTTCCGAAATTCATGAGCAAGATTAATGTACATAAGGTATCCGCTCATGAAGAAAAATGGATCAACGATGTTA

AAACAAGTCAACATTATAGCTGGTCCGTCAAGATATAACGTTTTCATTTGTATTTTTGTGGTATAATATTCTTACAAAAT

GATACAACGTGACACAGCAACAAATCAGAATGAGCAGTAAAAATATCCACACTGTAATATAATACGGTGTGTCGTAGGGA

AACTTATCCTCGCGAACCGTGCACATTATCGGGTCTACTTTGACGATGGTTTTGAATTGTTCGGGGGGAAACACCTCATC

AAATTTGCTTTGTAGTGATGTCTGTAAGTCTAGTGCCGAACAAGCGTCCGGCATGCATATTCCCAACTTCAAAATGTTTC

TGTGCATTTGGTCCTGATAACCAGCCCACCCGAGTATGGTTTTCCAAGCATGATTGTTACCAAAATCATCTAAATCTTCT

GTTCTCTCAAAGCTGTAATTTCTGTCAGCTGGTGGAATTAATTTTATTTCGGACAAACAGTACTGTCCTTTCATTGGATA

ACGTAAGTCTACACATTCATCGTAGTCACCCATTTGATATATGTTACCCGTGAGTAAGCCGACTGGGTATCGGTTCCAGG

ATTCTTGCATTCTGACGGCCCAACTGGTGTGATTGCGCAAATCCCTCTCATACACGGCGGTTTGACGACGGCATTCTGTG

CCAGCCGCTCGTCGCAACGTGAAGTTGGCCAACGCCCGGTAAAACAGATCGGATACCCAAAGATCCGGTATCCGGGTGTC

GCGCTGCATGACATGATCGTCGACGTCGGTTCTCGACGTACCGTCGGCCGACCGTAGCAGAACGATCTGCAATGCCAACG

CCAAGAAAGCGATCGTCGGCATCGACAGCCGTGACATATTATTGTACAATATTGTTTACTCACGTATAATAATTTCAATA

CCAATAAATTATCATGAATTAGTATTCGTCGAAGACGTTTATTTATTTGTACGGAAAAACAAAACACTCGACCATACGAC

GATCTCGGAAAAAAAAATCTATTTCATATTATTAGGTATACACACAATATTATGATTATGTGCCATCGTGCACGTTTTTC

AAACGTTTTATCTTTATTGAGAGTGTAACATAAAAAGTGGCCAATGAATAACACCACTCACAGGATTGAAATGCTCGATA

TGAGTATAGGTACTACGTATAATATATATATATATATATATATCATCACAATCGCGATCATCCAGTTGTAAAACAATGCA

AATTGTAAGGCCATCAATGGGTTACGAAAGAACTTCGTCAATTCTTTGTATTTTTATTTTTTTACAATAGTACGTATACA

TAATTATGACACTAATTTAAATTTTACAAAAAATGATTTGTAGACGCATCTATTATATTATTATTGATGTTATTGTTAGG

TTTTATTACGTTGGACATGAATCACAACAATAGCAGTGCAATATAGATATTATTTATTTTACACATTACAGTATAGATTA

GAGATGGGCTCAAATGGCGACCCTATGCCATGTATGTCACGGAGTCATCACAGATAGGTTGGATTGCTTAGAAGTTACTA

CTTATGAGTTTATCACTAAAAATATTTAAAAAAATTTATGTTCGATTTTTTATTTTTTTATTTTGCGTTTGAAGTAAATA

ATCTTCCCCATCACCGCTTGGCGCTTATTTCTTCCATTACACAGGTTTTTCTGTTGGAGTGGACGGTGTACAAAGACTGT

AAAGTAATGACGCACTAGATGACTACTACAGCCGCGGTGACCAAGTATTTAGTGATTTGCTTTCATTGGTAACAATGTAA

CATGAATTATTTTTCTATTTCTAATGTCGCGTCATTGTAACATGATAATTATTTTCTTTCTTTTTCCTCGAGAACTTTTT

AACGTTGTTTTTGAAAATTCAATTTTTAAGAAATCTTAAAATAATAATCTTCTTAATGAAAACTTTACCAAGTAATATGT

CAATAATACAGATAAAATACAGGTTTAACATCTTCTTAAAAATTTATTCAATCCACCTGCACCTATTAATTAAAATAAGT

TACTTTAATCATTTTTAACAACCTTACTGCTGTTGATAGTTGGTTTTCTGATCGGTGTTCCAAAATTGATCGACGAGTTT

TTATGTGTCCAAATATTTTGATGTAAGATGAATGGGTAACCTTTGTGAACTCTGAGATAATAGTAAAACAGTCATGTTCA

CCAACGAAACAGTGTACGATATTTACCCATAATCGTAAAAATTCTATTGTTAAATACATTATTAAGTATACCATATCCAG

ACGTGAAATA

>contig_9097

ATATATATATATATATAAATATCAATATAACCTAAATAATTGAGTATAAAAATCATATACTTTTATTATCTGTGTAAATA

GTTGGTTGTGTAACTAAATTACACAGTAGTTAACAATTAACTTAAGATAACGGAGTAAAAAAAGTTACTTAAAAATAATT

GCCAATTTCAGATTTTTTTGTTAACAGCCATAATATATAGCTTATACTATATACTTTTCGTTTTAAACTAAAAGGGTAAA

ATTTAATGGCACCCACAACTTTTAACTATCATATTTTTATACTTTTTTAATATAACATTGTTATCTTCCAAAAAAAATAG

AACTGCTTGTTGAGCCATTTTTGTGGGAGCACAACATGGTTTGGGGACTTGTAGAGGACTCATCAAATGCACCAATGTCT

GAACAATTGCGTGGTTAGTTGCATTCATGTGAGCATTGAGCGGGAAATTACACTCACCTGTGCAATAAAATGCACCATAA

CCATCTGGTGCTATTATCCAATCCTGCCATTCGAGATCTCTAAAGCTCACATATAAGGTTTGTATTTGACAACTTCTGGA

TTGAAATCCATCTGCTATATTTTGAAATGGGTTAGAGTTGTAGCTACTTTCAATAGTTTTCCTTCTTCTTCGTCTATAAT

GTTTTTGAAGAGAAGGACCACCAACTATATTACCTCCATTTCTAGACTTCAAATAAACAGCCATAAAAGGTTTTTTTGCT

TCAGATAATTGATGTTTATCAGCCCAAGTCACCATACCAATTTCTTCAGGTTTGATTTCATGATCTTCATTATCATGAGA

ATGTACAGAAACATATAATCCTCTATTAGGGTATAAGTAATGGGTCCATGAAATTAGAGCACCAGTTGCATTAAACCGAA

GCCATCCATCATAATCTGTTGTTGTATTTTGGGTATCCACAAATTCTAATTCTTTTTCTCCATTTTTTGTGATTAACACC

TGATACAATGTTACCGAATACTTTTTTATACTACTTAAAGTCCCAGATTGGTACAAATGAACTTCAGCTGAAGTAATCAT

ATGAGTTTCTTCAGGTGGTACATCATTTAATTCAAATAAAATACGTTTTCCATGTTCATGCCGAACATTACTAACTGGAG

AATGATTTGATAGGTATAGTGTGACAATAACATCACTATCTTGTACTGTGCGATCATCGTACTGACCAACTTCGGAAACA

CTTCTTGTTTTTCTGTCATGTAAACCATCTTGTTGTATGGATCTATAGACGTCAAACAAAAACTGTGGTGCCGATCCGTC

TAGATTCTTTGGAGATTTTCGTGGGATTTTCGGCACACCAAACATGGTCAGTATCTCTTGTTCAACTTCATTTCGTTCTT

CGACCGACATAAGATCTTGCTTAACTGTCTGAACACCGTTGTCCATGTACACACCAGTAGGTGATCCAGCAGCAACCATA

CCGTACGCTGATATGTGGAACAGTACGGCAAACATAAACAGACATTTTTTTTCGTACATTTTTGTTGTTATTGTTATTAG

TGAACTATTATTTGGTCATAAAACGAGGGGATTCTGTAGTACGAGACGCGAACGCTGGAAAAATACAACACTACAACAGT

CAACAACGATCCGAACTCAATCCGATTAGCCCACGG

>contig_9124

AGTGTCTCTTCAGTGTTAGTTGGACTTGGAATTGTTGTAACTACCGGTTTTGCTATATCAGCATTTGTAGCCAATTTCCA

AAAGAAAAGCCAGAAAAAAAGAACTTTAGTGGATTCAAATACAAAGATTCCATTGCCACTTATTCAAAAACATATTATTA

GTCATGACACACGAAGATTTAGATTTGAATTGCCATCCAAAAATCATATTCTTGGTTTACCTATTGGGCAACACATACAT

TTATCAGCAAGAATAAACGAGGAATTAGTTGCTCGTGCTTACACCCCAGTCAGTAGTGACAATGATGTCGGATATATGGA

TTTGGTTATTAAGGTTTATTTTAGAGATCAAAATCCCAAATTCCCTGATGGTGGTAAATTAACACAATATCTTGAGAAAA

TGGAAATCGGAGACACAATTGATGTCCGAGGACCTTCAGGTCGTCTCATTTATCATGGTCGTGGAGATTTTGAAATTAAG

GCTGTTAAAAGAATAGATCCTTCTCATAACCTTTATGCTAAAAAATTATCAATGATTGCTGGTGGTACTGGTATTACGCC

AATGTTGCAGTTAATTAGACAAGTCACTAGAGATCCTAAAGATGAAACCAAATTGTCATTACTTTTTGCTAATCAGACTG

AAGAAGATATATTATTAAGAGATGAATTAGAAGAAGCTGTTAAAAATTATCCAGATAGGATCAAAGTTTGGTATACAGTT

GATAGACCTACTGATGGGTGGAAATACAGTGTTGGATTTATATCATCTGACATGATATCAGAACATCTATATCCACCAGC

TCAAGATACATTGGTATTAATGTGTGGACCTCCTCCAATGATAAACTTTGCATGTATTCCAAATCTTGATAAACTTGGAT

ATGATGCTAAGTTACGGTTTTCTTATTAAAAACAAAAAATAAAAAATGTTTTTCATTTATATTAGACCGTCCGTAACAGT

TTTTGAGGTGTATTTGTTGATTCACATTTTGTAATAGATTTTGATCATTGAACGGGTATTTGGTATTAAAAAACATTAAA

AACCCTTTTATAATGTTGCTAATAAATTGGTGTTAGCATTTAATTTATTTACAAATTGTTTAATCTAGCTAAGCATAATA

CAATATATTTATATAATATTTCTTCAATATATCTATTTTAGAATGTACTTCAAAACATAAATATTTTGAAATTCCTGTTT

TGGCTTACACCAAAAATGTATATATTCTTATATTATATATTGTATGTGATGTGTTTTAATAAAATGTTTCATTTTAAGAA

ATTAACTGTTAATTGATACATTGTGAGAAAATAAAACAATGTGAATGAATTCTAAATGATTACTGAG

>contig_943

CGTACTCATTATTGACCTTACCATAATTTTTCACCAATCATTTCACCCGTACACCATATTTACTATAACTCCCATCATCG

TTTTCGTGAGATCTCGATTGATTTTTCGATTATTAGTTCAAGGCACAGGCGAAAAATTAACTTGTAACCATGTTATCCAG

ATTAGCTCTTCGTTCAGAACTGTTCAGAGTAGCACCACTGTGTGCTAATGCCGTACGTACCACACAAACTAAACCGGCAG

TCGCTACCACTAATGAATCAATCATTGTACCAGGGTTCCCTAAACCCAATGGTCAACCTCAACCGAATCCTTATGACGGA

CCCGAACGTGATCTTGTAAATTTTCCCAGAATGGTGAGGTTAGAAGAACCTGCTAAAACTAGATATTTGTTCGTGCCCGA

AGAGTGGTTTGAAGTATTTTACAAAAAGACTGGAGTCACAGGTCCATATGTTCTTGCTGCTGGTGTCACAACTTATTTAT

TAAGTAAAGAAATTTGGGTAGTTGAACATGAGTTCCCTTATGTATTGGCTACTATTGGTTTGTTTTATGTTGGATGGAAA

AAGTTCGGAACATCTTTAGCTGCTTTCCTTGATAAAGAAATTGATGAATACGAAGCATCTTGTAATGCTGGTCGTAAAAG

TGAAATTGATGGCTTAAAAGAAACCATTGAACATCAAAAAACAGAAATATGGAGAACTGAAGCTCAAAAACATGTAATTC

AAGCAAAGCGAGAGAATGTAGCTCTTCAGTTAGAAGCTATTTATCGCGAACGTGCTCTTCAAGCCTACAACCAAGTAAAA

AGACGTTTAGATTATCAATTGGATTTGGCTAACTTGACACGTACTGTGCAGCAGAGACATATGGTTAACTGGATCATTGA

AAATGTTCTCAAATCTTTAACAAATGAACAAGAAAAGCAAAGTTTTAAAAAATGCATGACAGATTTACAAGCTTTAGCTG

CAAAAGCATAAATGTAACAAGAGTTAGTGCCATTTTTTAGAATATTGTTTAATGGTAAATAAACTTAATTACATTCATTA

ATATACTCCTTTCACAGTAGATTTCATGTTATTTATATGAGATAAGAAAAAATTATTATTTGGAGTATGATATTATTAAT

ATCTTACACAATATTCAAACTAATAATGTTTTATGTACTCTAATTATTACATTCAACACAAAATAATGTTATACTGTTAT

TTAAAAAAAAAATAAATTCAATTTTACCATTATAGATTTAATTTATTCTTGATATTTGTACATGTGGATGATATATATAG

TCCTAAAGTAAATGAATCATTTTACTCAGTGA

>contig_9694

CATAATATTATAATATATTATATTATATTTTATATATCGATCCGATATATACGTATATATAAATATTTTAATATTTTTAC

CGTCCGTCGTACAAACGCTGCCCAATACACTATATCTCTTCGCCCGTTCGACCGTCTTGTTCACACCCGGTCCATCGGCT

CTCCGCGCGGCGGAGGCGGCGCGAGATGACGGTCGAGATCATGACCGAAGGCGGCGGATACAGCCGCGAACTGTGGCTGA

ACGCGGTCGCAGCGGCGCTGGGTCTGACCTACTCGGCGTACCAGCAGCTGCGGGCCGCCAGGACGCTGCCACCCGGGCCG

TGGGGCGTACCGTTCCTGGGGTACGCGCCGTTTTTGTCCAACCACTGCACGTACCTCAAGTACAATGAGCTTGCCCGCCG

GTACGGGCCCATATGCTCGTTCACGCAGCGCGGAAACACCGTCATACTGCTCAGCGACCACAAGCTCATCAAGACCGCGT

TCGACATGAAGCAGATCACCGGTCGTCCCAACGACGGGTACATGGACATCATCGGAGGATACGGAGCGGTAAATAGCACT

GGGAAGTTATGGGAGTCGCAAAGGAAGTTTTTGCATTTAGTACTTCGACATATGGGGATGACGTTTACGGGCCACAACAG

GTTGAACATGGAAAACAGAATAATGATCGAAGTATCAACGTTAACGGAAACATTTCACAAGGCCTGTGGTAAACCAATCG

ATCTAAATGCCGGTTCGTTATGCCTGGCCATCACCAACGTGATTAGTTCATTGACGATGAGTGTTCGATTCGAACCGAAC

GACCCGCGCTTCGAGCGGTACATGCACATGGTCGACGAAGGTTTCAAGCTGTTCGGCATGTTGAGGCCGGTGAGTTTGTT

TTTGCCTAGACGTCATATTAACGACGAACGAAACATACAGGAAAAAATTAAGAATAACCATCGAGAAATCGCCGGATACT

TTCAAAATATTATCGAAGAACACAGGAGTACATTCGATCCTAATTGCATTCGAGACCTGGTCGACGCTTATCTGCTGGAG

ATAAATCGCTCACAAGAAGCCGGCACGATGGACCAATTATTTCAGGGTTTAGATCCGAACAGGCAGGTTCAGCAAATTCT

CGGTGACTTGTTCTCGGCCGGCATGGAGACTATTAAGAATACGATTTTATGGGCCATGGTGTACATGCTCCATTATCCGG

ATGTGATGACCAAGGTACAAGAAGAGATCGATTCGGTTGTGGGTCAATATAAATCGCCGGTGTTGGACGATTACCCCAAT

TTACCATATACACAAGCCACCTTATATGAAGTACTGCGGAAATCGAGTATCACGCCTTTGGGCACGACTCACGCAACCAC

CAGTGATGTAACACTCAATGGTTATCATATACCGACTGGCGCTCAAATCATACCTTTACAACACTTTGTACACAACGATC

CAAACTTATGGGACGAACCGGAGGCGTTCAAGCCGGAGAGATTTATAAATGCCGAAGGCAAAGTGAAAAAACCCGATTGT

TTTTTACCTTTCGGAGTTGGTCGGAGAAAATGTCTGGGTGAAACGTTAGCTCAAATGGAACTATATTTGTTCTTTTCAAC

CTTGTTACATGAATTCGATGTATGTCTACCAGATGGTGACGAATTACCTAGCATGGACGGCCAAGTCGGTATCACACTAA

CTCCACAATCGTTCAAGGTCGTTATGAAAGCACGCAACAAATAGATTTTATCTAAATTTTTTATTCTCAGATCCTCTTAT

TTAGGTTGTTTATGTATTTTTAATGGAATATTATGTATTTATTAGAGTAATATTCCATAGTAATTGTTTAGTTTAAAAAT

GGTTTATTATTTATTTACCAATTGTACGTGGTTCCCTATAAATTATATACACATTTTCTAATATTTAATTTAAAACCATA

CTATTTATTGATTAGCTAGTTTAGTTTAATTTATACTGTTATGTAAAATAACAATTTTATTTGTTTATTTAAAGTAGTTA

GAAGTGCATGTTTTCCTATTGATATGTTTATGTACTTAGTGCCATATACCATTTTTTGTAAAAATGATTTGACGTAATGC

TTTATTTTGTATTAAAGTATAATTGTTATTTTTTTTATATATTACCAATGTAACAATCAACCTATT

>contig_9851

AACAGTTACGAACACTTGTATATTATAAATAAATAAAAAATATTACAAATATAGAATTTTAAAAATTTAAAAAATTTAAA

TGGTAAATACTAAATATTATAACAAGTTAAATATGAACAGTTTATTTTCACCAAATAAGAATTCAATAATGTTTTAACAG

TTATTATTTTCAATTTAAATTTAACTATAAACATTATGATTTACACGCTATTAACAATATTGTTTTTAAATTATATTTAT

TTTATATTTTTACACAAAAATCTACAACTTAATATTTTACAATTATTTCAATAAAAATTATAAAAATTAAAATAACTTAT

ATTATATTACAATTTACCCTTGTTATCATTGTTTAATAACATTTCCTTTAAACTTTAATAATACATTGGTTCTATAGATC

CTATATTGCTTATACCGATTTGTAATAAGTTATATTGTATATTTTATAGATTATAAATTTAATATTAGCTTATGTATACA

AATGCAAAGTCTTTTATTTGGTAGAGTATGTTTACAAAGAAATATTTAAAATGGTTCTGTGAATATAATATATAATGTTA

ACATAAGAATTATGCAATAAAATAATATAAAAAGGTATTTCCAAGTAAATCTTAGAATAGTTACATTAATTATATGTGAT

ATTGTAATAAACACTTAAAATAATGTATAAGTAGTGTATTGTATCTTATTGTATGTACCTTTGGTTTTTAATTTTTATTA

TTTACATATTTATTTCAATAATTTTTATTGCCAATTTGTGAATTTGCAATATTAACGAATATAATAATATTTAGATACCT

ACAATATTAAACGCTTTGGTAATGATTTTAATATAGATAGGTAGAGCATTAAAAGAAAAAAGAAATCATTCGATATATGT

AGGTAGTAGGTACATACTACTGTCCCAGAGTTATTACATAAGTAATGATTTATGTACTCCAAGCATAATATAAGGAACTT

TATATTATGACATATCACTAAAAACTATTGCTACCTAACAATTAATTTGTCTGTAAACCATTTTTTGCAAAAAAAAAAAT

CTAAGATTTATAAAAGTTATCCTTTTTTGAATTTAATCAACAGTACACTAGATGTAAGGAATACCGAAATCCATATTAAT

GTGGAACAAAAACCAATGTAAACAGAAGGTTCGGTGATTGGCCAACCTCTTGCCAACATTGATCTCATCGACTCCGTAGA

CTGCGTTAGTGGCAAAATAAAACTTATTGATTTTGTTATTGCGTGCATTCCTTCCACTGGCCAAATTATGCCACAGAGCA

TGACAATAGGTAGAAAGCTGCCCATAGCCAAATATGTTGCATTCCTCTCACTGTCAGTTACACAAGAAACTACAAACCCA

AAACACATGCCACACAATCCAGTCAATATTGTTAATAAGCATATCCAAAACACATCTCCTTCGTTGGTAACATCGAAAAC

AACCAATGCAAATGCAATGACCATCAGTGATTGGAACACCATAATTAAAAGTTGACATACTACATGTCCTAAAAGTACTT

CAATCCCCGTAATACCTGAAACTAAACTTCTTTCTAATATGCCTTCATTTCTTTCAATCAACATAGCACCAGACGTCAAA

GCTACGGCTAAGAAAAATATAATCGTGAGTATGACCCCCGGAGCAGCAAAATCTGTAAAATTTGGTACGTATGGACCGTA

TATAGGTTGATTGAACTGTATAGGATTAGCTACAACTTTCGTACTGAAGTTACAAGTTACCGCCAATGTTTTAGCAAAAT

CTTGGAAAGCAAACAAAAGATCTCGTCGAAGCAGCATGGATATTTGTTGATTTGATTCATCGAGCCAAATTGAGACAACA

CTATCGTCTAGTACCCAATCCGTTGCAGACTGTCCATCATCCATACGTTTTTTTAGAGATGTTGAATAGTTTGAAGTGAA

AGATAAAGCACCCCAAGCTTTGCCTCTTTTAACTGCTCGAAAAGCGGATTCTTCGTCATCGAAAAAGTCTAATACTTGCC

GACGTAGTTCCATTTGATGTAGGAACTGGCAACTGAGTTGAGTTAATTCGCAAGTTCCGGTCGGTGGACAAGTTTTGTTA

TTAGTTTCATGGTTCACGATGGCCAAATGCAAGCCATATGGATCTTTTCCAACAGACAGACAGAACAACACGATTTGAGC

AACTGGAAGTCCCACAATAAACAACATTACCGCCACATTTCTCCACATCCACAGAAAGTTTTTCCATATCAATGCTCGCA

TATGGTGCGATTGAAATAGTTTGCATCGGTCGAAGAGTGTCATTTTTGTATTTGCTTCTGGTGGAAGTTCCATTTCCGCA

TTGTTGACTTGCATTGAACATACAGACATTCGCTTACTAAGTGGAACAGGACTAATACTGTCTCCAAATTCTCCGCTCAT

TTCTGACGAATTGTCACATATCGAATTCAACGCTTCGACAGCAGGTACAGGAGAAGTTTCCGGAACAGCAATGGCAGCTG

TTATGTCATGCAAAATGCTACTACGCCTTCGTTTACCACGATTTTGAATGACACTAAGCTTTAGAAAAACGTCTTCTAGT

GATTGGCAATTGTATTTTTGTAACAGCGATGTGGGTGATTCTTCAGCCAAAAATTTACCACCTCTCATTAAACCAATCAT

ATGAGCTTGTCTAGTCTCGTCGATGTAATGTGTGGTAACAATAATGGTTGTTCCTCCATCCTTAGTTAAATGGACTAAAT

GATCCCATATTCCTTGACGAAGAACTGGGTCAACACCAACAGTAGGTTCGTCCAAAATCAGTAACTCGGGATTGTGCAAC

AGTGCCGCAGCAAAAGAAACTCTTCGTTGCTGTCCACCACTCAAATTTTTTACTGGCCTACTGGCGGACGGTAACTGTAA

AAATTTTAGTAAAAATTCTATTTTGTCATCTACTTGTTTTGGTGTCATGCCGTTTACCATACCAAAAAATTGCATAGTTT

CTCGAATTGTAAACTCTCCATAAAGCGCAATTTCCTGAGGCATATATCCAATACGAGGTCCCGGAACACCGCTACCGGCA

GATCCCGGTTTACCACCTAGTACCCATATTTCACCAGCATTCAGTCTTCTACGACCAACTATACAACTTAACAATGTAGT

TTTTCCACATCCACTAGCTCCCAAAAGACCATATATGCAGCCTTTTGGGACAGTCATGTTAAGCCCATCAAGTATGACGT

GTGGATTTGAGCTCTGTCCATATTTCTTATAAGCTCGTCTTACACATACAGCTTGTTGGCGTCTCGTCCATACCGTTGAT

TGTTGGGTTTCCAGTGGAGGCCGTCGTTCTGTCAAACTAATTGGCAATTCTGGACGCATCGTGGACTTGTGTTACGAATC

CTCCGTGCGTTTTATATAGGTAAATATATTGTTATAATATTGTATTTTATGTATCGATATACTGTTCTAGAGCTGAATAT

CAATGAACGAATTGGCTCGTGAAATCCGTCCGTTTGCGAAGCGTATCGATAACGGAATGTAACTAAACTCGCGATGACAA

CGGCTATGGAGTCATTTGATCGCGACGACGGGGTGGTTCGTGTGCAGCACTCGCGGCGGTCGAACAGTTCGCGGTCGGTA

CATCAGAGACGGACGAGAAAATTGTTTCAATAAAATTTCGCGTATATCTTCGTGTCGTCGTCGTCGGTCGCTCAGCGTCG

TGCGAGTGACGGTAACGTTTACTTGCAGCGGACCGCGCAGAGTGGTCTGTAACGTCTCGCGCGATACGAGTAAGTGGCGT

GCGCGCCGGCACACCGTATAGTGGCGTGTGCGTAAAAACCCACAGGAGGTGGTGA
