# Supplementary material for: RNA-Seq reveals a xenobiotic stress response in the soybean aphid, Aphis glycines, when fed aphid-resistant soybean
Source: BMC Genomics. 2014 Nov 16;15(1):972. doi: 10.1186/1471-2164-15-972 (PMC4289043; doi:10.1186/1471-2164-15-972)
Supplement: Supplementary file 12 — Additional file 12: Gene names and primer sequences used in this study. (DOCX 35 KB) [file 12864_2014_6855_MOESM12_ESM.docx]

**Primer sequences used in this study**

| **Transcript ID^1^** | **Top hit description** | **Primer sequences** |
| --- | --- | --- |
| *For quantitative real time PCR* | | |
| contig_2224 | hypothetical protein LOC100162252 | CTTCTTAAGCGATGTCTACTAT |
|  |  | CTGAACGGATCTTGATGAAT |
| contig_4515 | serine protease | TATTCGTCTACTCGCTGAA |
|  |  | TATGTGGGTTGGTGTGTA |
| contig_7286 | l-ascorbate oxidase-like | CGTTCAGATACCAATTCG |
|  |  | ACAATACTACCATAGATACCA |
| contig_5265 | facilitated trehalose transporter tret1-like | ACGGTATTGGAAAGTTAGG |
|  |  | GTCCAGCCGAGTATTATG |
| contig_12531 | cg17600 cg17600-pa | TATAATGCGTCTTGTCTT |
|  |  | TATCTTGCTCTGTGTTTA |
| contig_4476 | fatty acyl- reductase 1 | GATTCTTCATTTGGGATGCTAA |
|  |  | ATTGTGCGGCTACGATTA |
| contig_8994 | nose resistant to fluoxetine protein 6-like | GCATTTGGTCCTGATAAC |
|  |  | TGAGAGAACAGAAGATTTAGA |
| contig_5850 | glucose dehydrogenase | AGTTGAAGATTGGAGTGTA |
|  |  | ATGCTGTATTCTGAGGTT |
| contig_559 | protein disulfide-isomerase a3-like | CGTGGTTGTTGTTGGATT |
|  |  | CTGATTGTGAAGTGGTATGAC |
| contig_16027 | cytochrome p450 4v2-like | TTACGAGATGCTTGAACC |
|  |  | CGAGAGTGATTATTGAGTGA |
| contig_9694 | cytochrome p450 18a1-like | ACGGAAACATTTCACAAG |
|  |  | ATCGTCAATGAACTAATCAC |
| contig_3571 | glutathione s transferase d1 | TTGCTAATGCTGCTAATGGTTAT |
|  |  | GAGCCACAATACTCACGATAA |
| contig_45413 | uncharacterized protein LOC100160919 precursor | GGATGAAGATTATATGAGGTGGTA |
|  |  | TGTATGGTTAGGCTCATTAGAC |
| contig_14185 | probable cytochrome p450 6a13-like | CTCGTTCGTGTCCCGTTC |
|  |  | ACGAGTTATGTCGGTAAGGTT |
| *For semi-quantitative PCR* | | |
| contig_6230 | Armet/Endopeptidase inhibitor | CTAAGCAACCATCACAACTTTCTC |
|  |  | GATGCTCAAGTGTGCGATATTAAA |
| contig_7391 | M1 zinc metalloprotease | CGTCGGACATCCTGATGAAATA |
|  |  | CGTATCCTCGCCTAAGAAAGTG |
| contig_351 | Calreticulin | TGTCTGCTGCTTGGTATA |
|  |  | CGTTATGTGCTATGGAATCA |
| contig_559 | Disulfide isomerase | CGTGGTTGTTGTTGGATT |
|  |  | CTGATTGTGAAGTGGTATGAC |
| contig_2545 | Disulfide isomerase | CACCACCAGATATTATTG |
|  |  | GTTCAGATACTCATTACG |
| contig_8225 | Trehalase | GGAGGCAGATTTAGAGAAC |
|  |  | CGACGACATATCACATAATAAC |

^1^ Nucleotide sequence for each contig is provided in Additional File 12.
